# Correction: A zebrafish embryo screen utilizing gastrulation identifies the HTR2C inhibitor pizotifen as a suppressor of EMT-mediated metastasis

**DOI:** 10.7554/eLife.101706

**Published:** 2024-07-31

**Authors:** Joji Nakayama, Lora Tan, Yan Li, Boon Cher Goh, Shu Wang, Hideki Makinoshima, Zhiyuan Gong

**Keywords:** Zebrafish

Nakayama J, Tan L, Li Y, Goh BC, Wang S, Makinoshima H, Gong Z. 2021. A zebrafish embryo screen utilizing gastrulation identifies the HTR2C inhibitor pizotifen as a suppressor of EMT-mediated metastasis. *eLife* **10**:e70151. doi: 10.7554/eLife.70151.

Published 17 December 2021

It was brought to our attention through PubPeer that the KRT14, KRT19, and GADPH gel bands of Figure 5D were duplicated from Figure 7G of an article we published previously in Molecular Cancer Research (MCR) 2020; 18(3):477–487. doi: https://doi.org/10.1158/1541-7786.mcr-19-0759. The duplicated bands in Figure 5D were not present during the first round of review and were introduced when the manuscript was revised. Specifically, when preparing Figure 5D for the revision, we reused the template (format and labels) of Figure 7G of the MCR article and inadvertently forgot to replace the KRT14, KRT19, and GADPH bands from the MCR article template with the correct KRT18, KRT19 and GADPH bands for the current study.

We are therefore correcting Figure 5D to include bands for KRT18 (instead of KRT14) and the correct KRT19 and GADPH bands. This now correctly aligns with the quantification data for KRT18 and KRT19 in Figure 5—figure supplement 2 and with the source data of the original gels we provided for KRT18, KRT19 and GAPDH.

We noticed a second error where the immunofluorescence images and corresponding figure legends in Figure 5E and Figure 5G were inadvertently switched. We have corrected this by swapping the panels from Figure 5E with those in Figure 5G. The original figure legends remain correct.

Lastly, the GAPDH band in Figure 4C (bottom left) and GAPDH in Figure 5—figure supplement 4 (bottom left) were obtained in the same experiment. In the original publication we inadvertently forgot to include a statement to explicitly state this in the legend of Figure 5—figure supplement 4.

Corrected images of Figure 5 is shown here:

**Figure 1:**
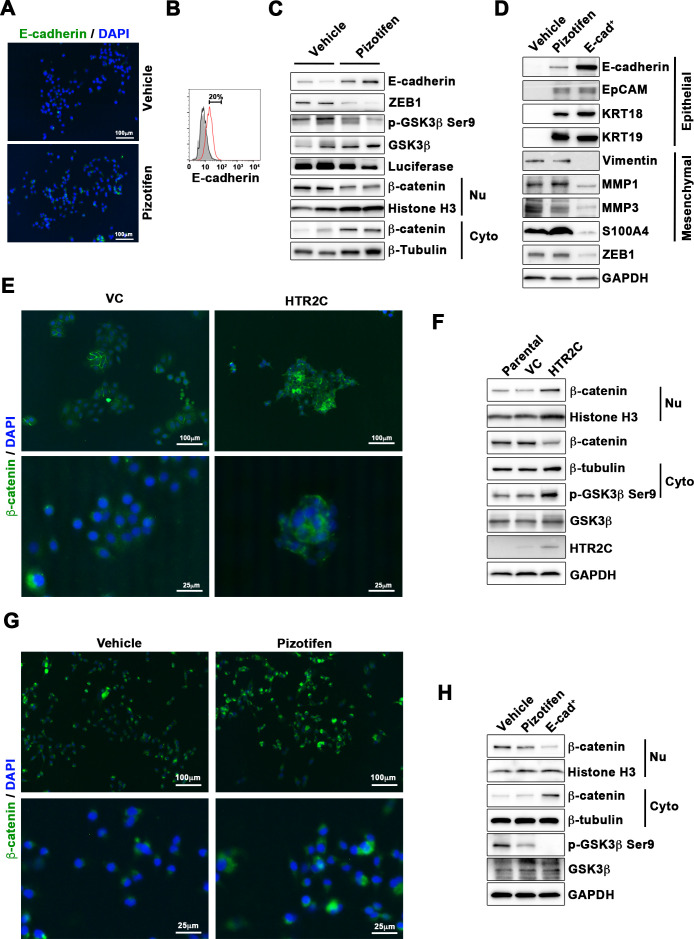


The originally published Figure 5 is shown for reference:

**Figure 2:**
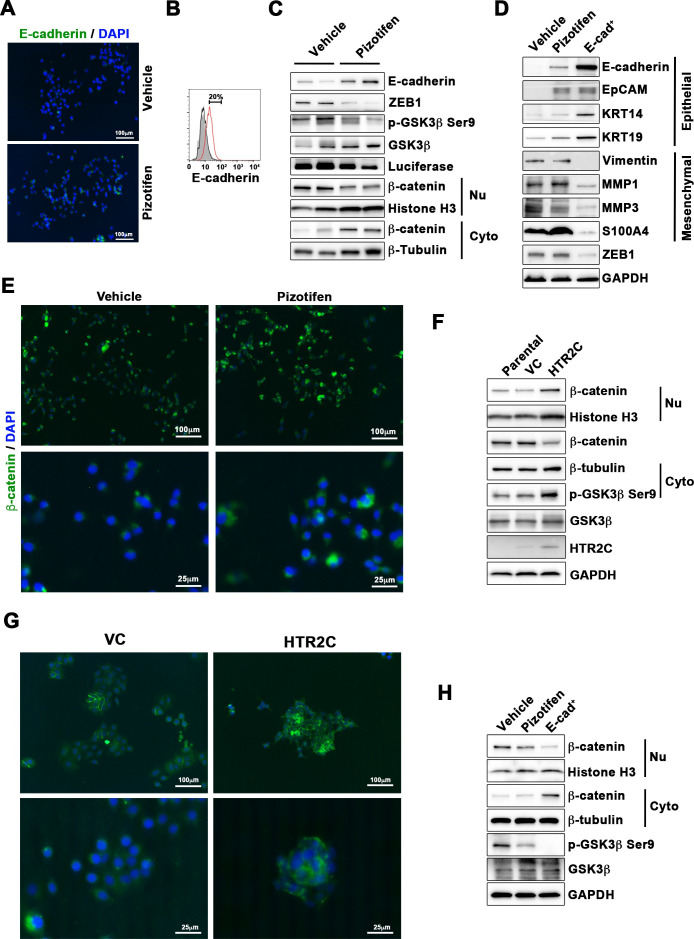


The corrected legend for Figure 5—figure supplement 4 is shown here:

Expression of Snail and Twist1 was examined by western blotting in the MCF7 cells (left); GAPDH loading control is shown (bottom). GAPDH band is the same as one from Figure 4C since GAPDH bands in Figure 4C (bottom left) and Figure 5—figure supplement 4 (bottom left) were obtained in the same experiment. Protein expression levels of Twist1 in either vehicle- or pizotifen-treated MDA-MB-231 cells are shown (middle): GAPDH loading control is shown (bottom). Protein expression levels of Snail and Twist1 of 4T1 primary tumors from either vehicle- or pizotifen-treated mice are shown (right); luciferase is used as loading control for whole cell. Luciferase control was obtained in the same experiment from Figure 5C.

The originally published legend for Figure 5—figure supplement 4 is shown here:

Expression of Snail and Twist1 was examined by western blotting in the MCF7 cells (left); GAPDH loading control is shown (bottom). Protein expression levels of Twist1 in either vehicle- or pizotifen-treated MDA-MB-231 cells are shown (middle): GAPDH loading control is shown (bottom). Protein expression levels of Snail and Twist1 of 4T1 primary tumors from either vehicle- or pizotifen-treated mice are shown (right); luciferase is used as loading control for whole cell. Luciferase control was obtained in the same experiment from Figure 5C.

The article has been corrected accordingly.

