# Supplementary figures and images for: A zebrafish embryo screen utilizing gastrulation identifies the HTR2C inhibitor pizotifen as a suppressor of EMT-mediated metastasis (part 2 of 2)
Source: eLife. 2021 Dec 17;10:e70151. doi: 10.7554/eLife.70151 (PMC8824480; doi:10.7554/eLife.70151)

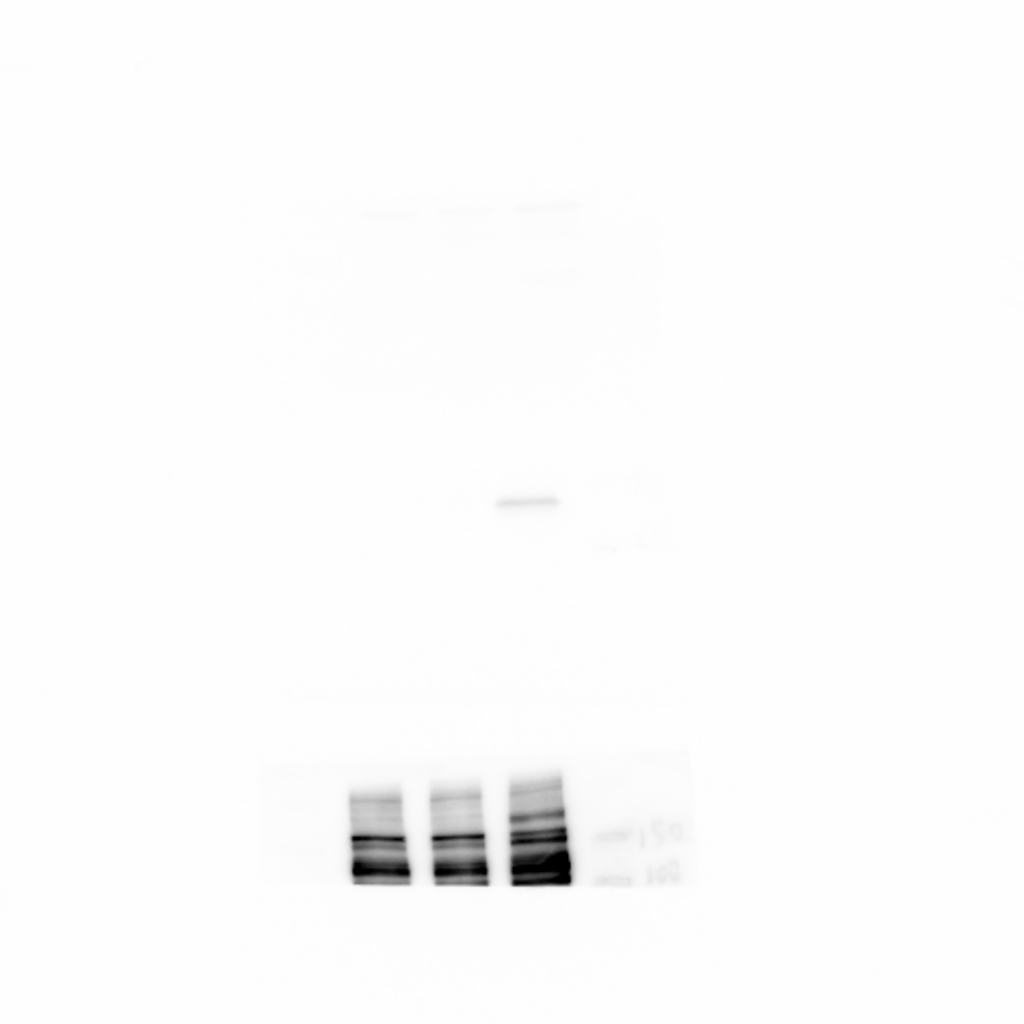

Supplement: Source data 3. [file elife-70151-data3.zip › Source data_v2/Figure 4C/MCF7/Figure 4C_ Zeb1 in MCF7_source data.jpg]

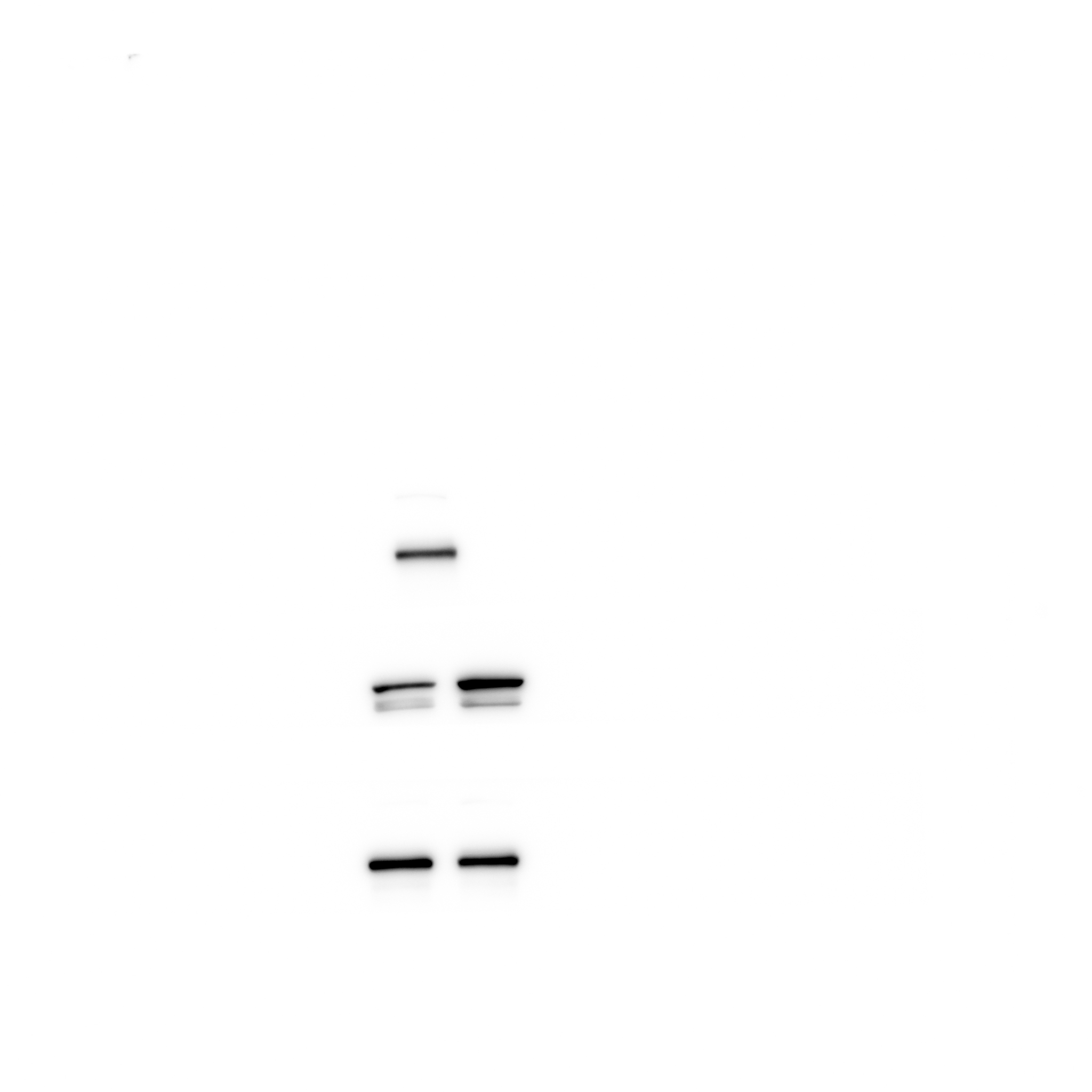

Supplement: Source data 3. [file elife-70151-data3.zip › Source data_v2/Figure 4C/MCF7/Figure 4C_N-cadherin in MCF7_source data.jpg]

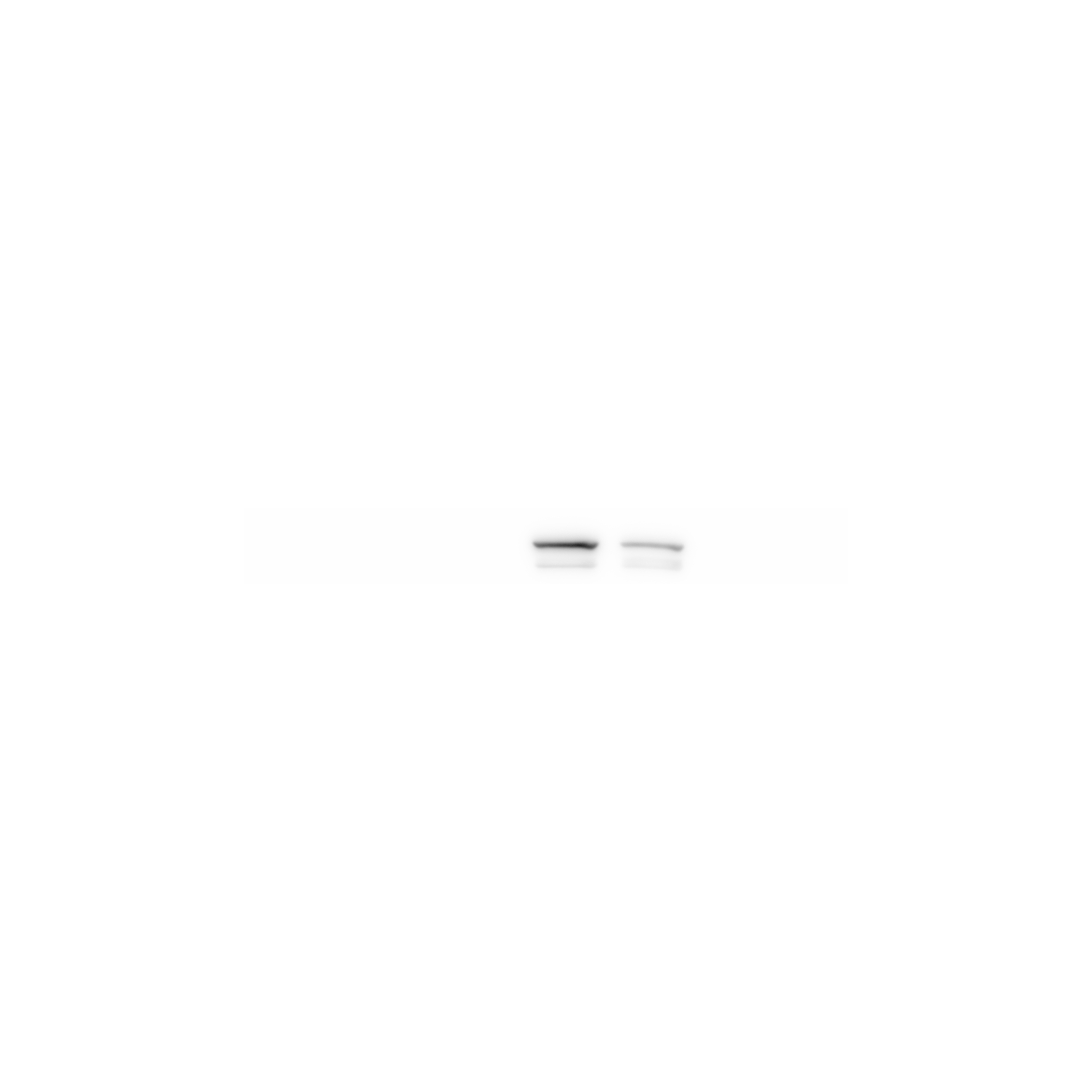

Supplement: Source data 3. [file elife-70151-data3.zip › Source data_v2/Figure 4C/MCF7/Figure 4C_Vimentin in MCF7_source data.jpg]

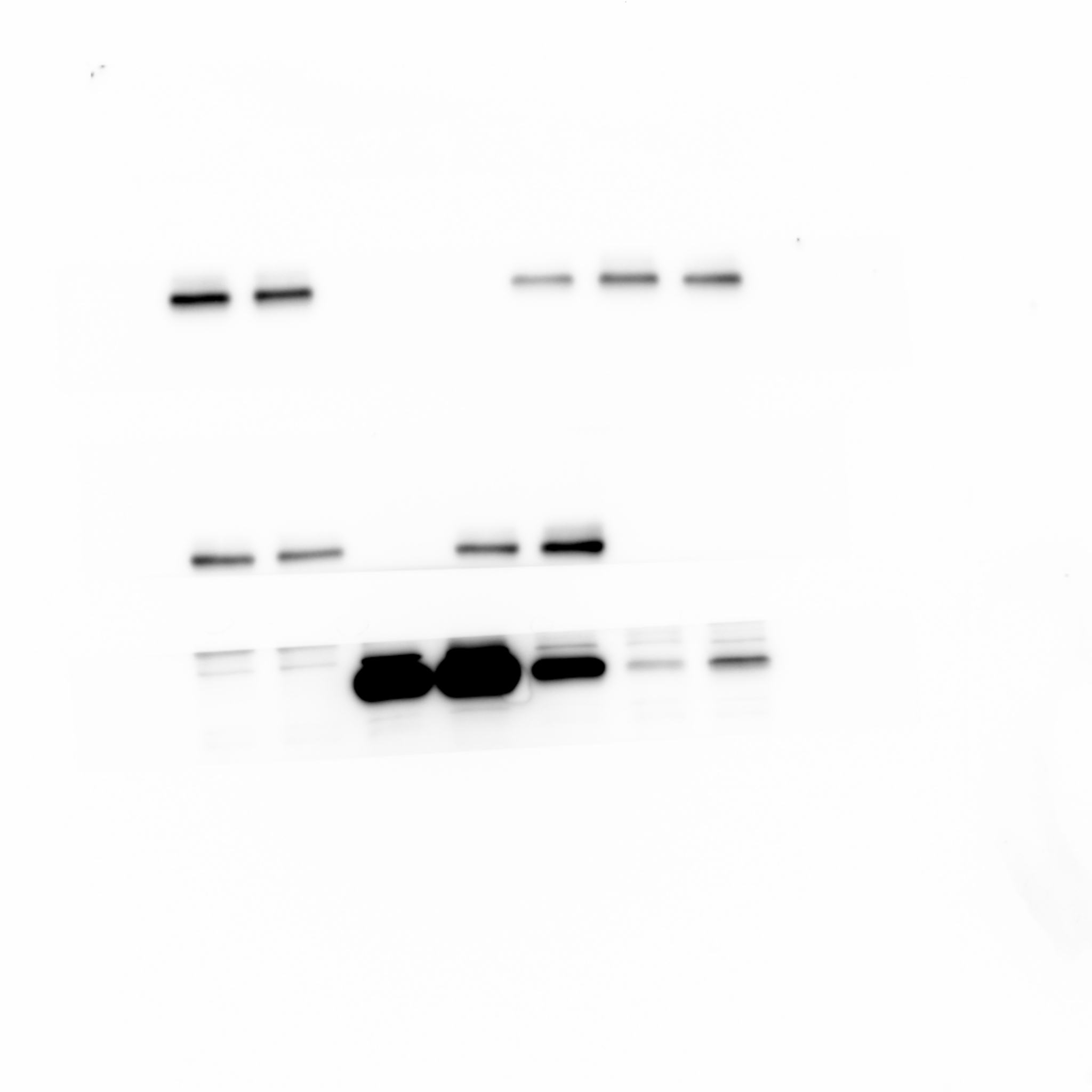

Supplement: Source data 3. [file elife-70151-data3.zip › Source data_v2/Figure 4C/MCF7/Figure 4C_EpCAM in MCF7_source data.jpg]

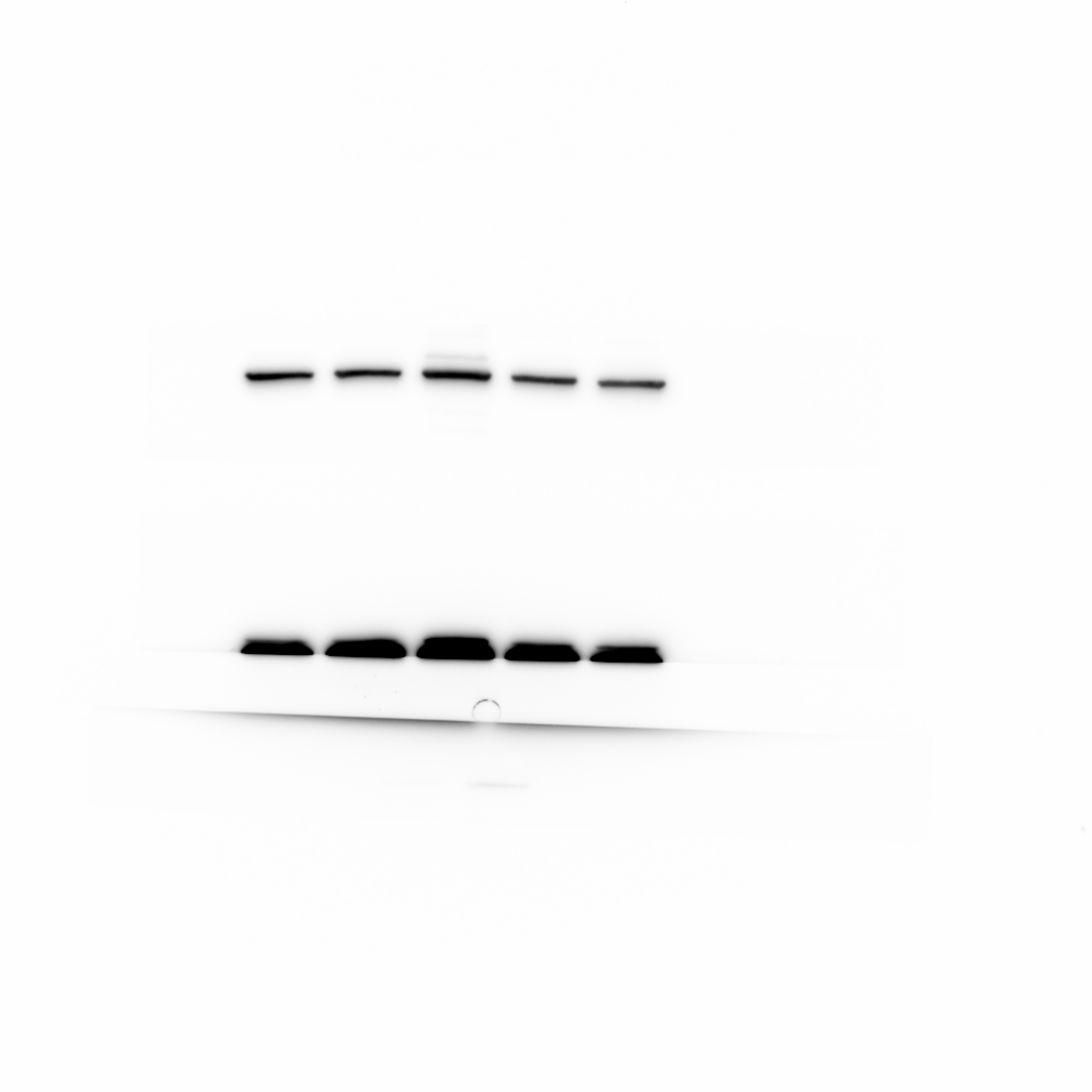

Supplement: Source data 3. [file elife-70151-data3.zip › Source data_v2/Figure 4C/MCF7/Figure 4C_GAPDH in MCF7_source data.jpg]

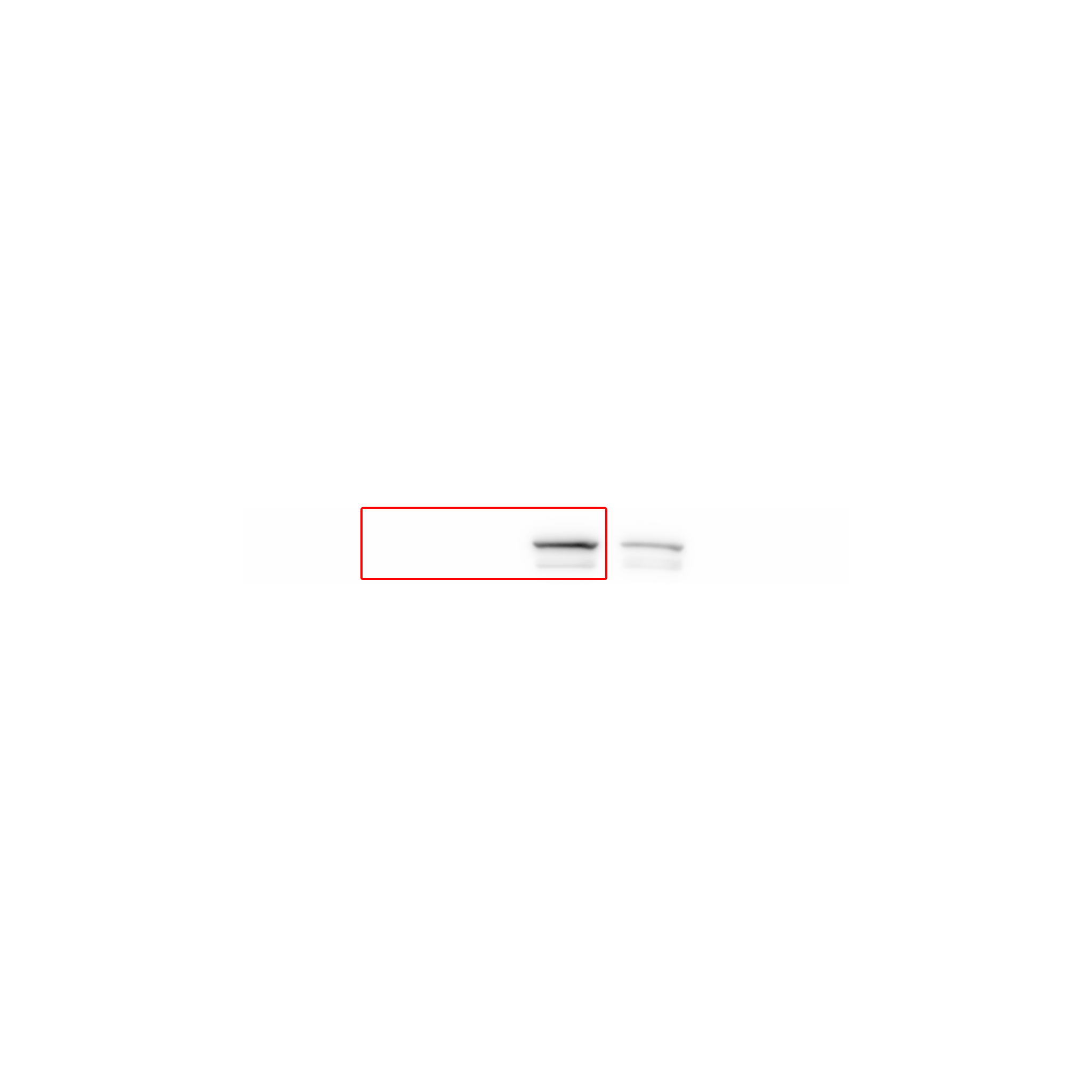

Supplement: Source data 3. [file elife-70151-data3.zip › Source data_v2/Figure 4C/MCF7/Figure 4C_Vimentin in MCF7_source data_labelled.jpg]

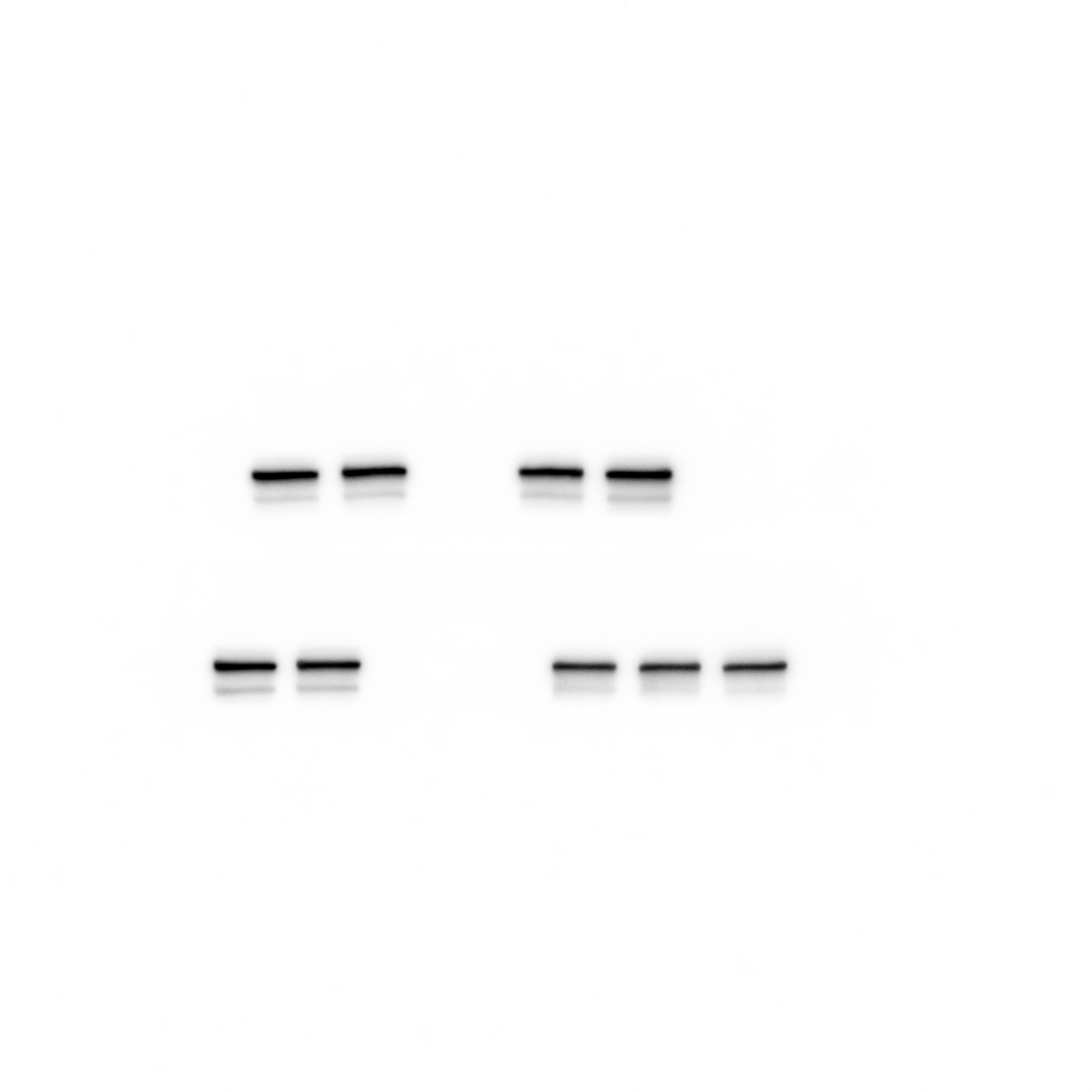

Supplement: Source data 3. [file elife-70151-data3.zip › Source data_v2/Figure 4C/MCF7/Figure 4C_E-cadherin in MCF7_source data.jpg]

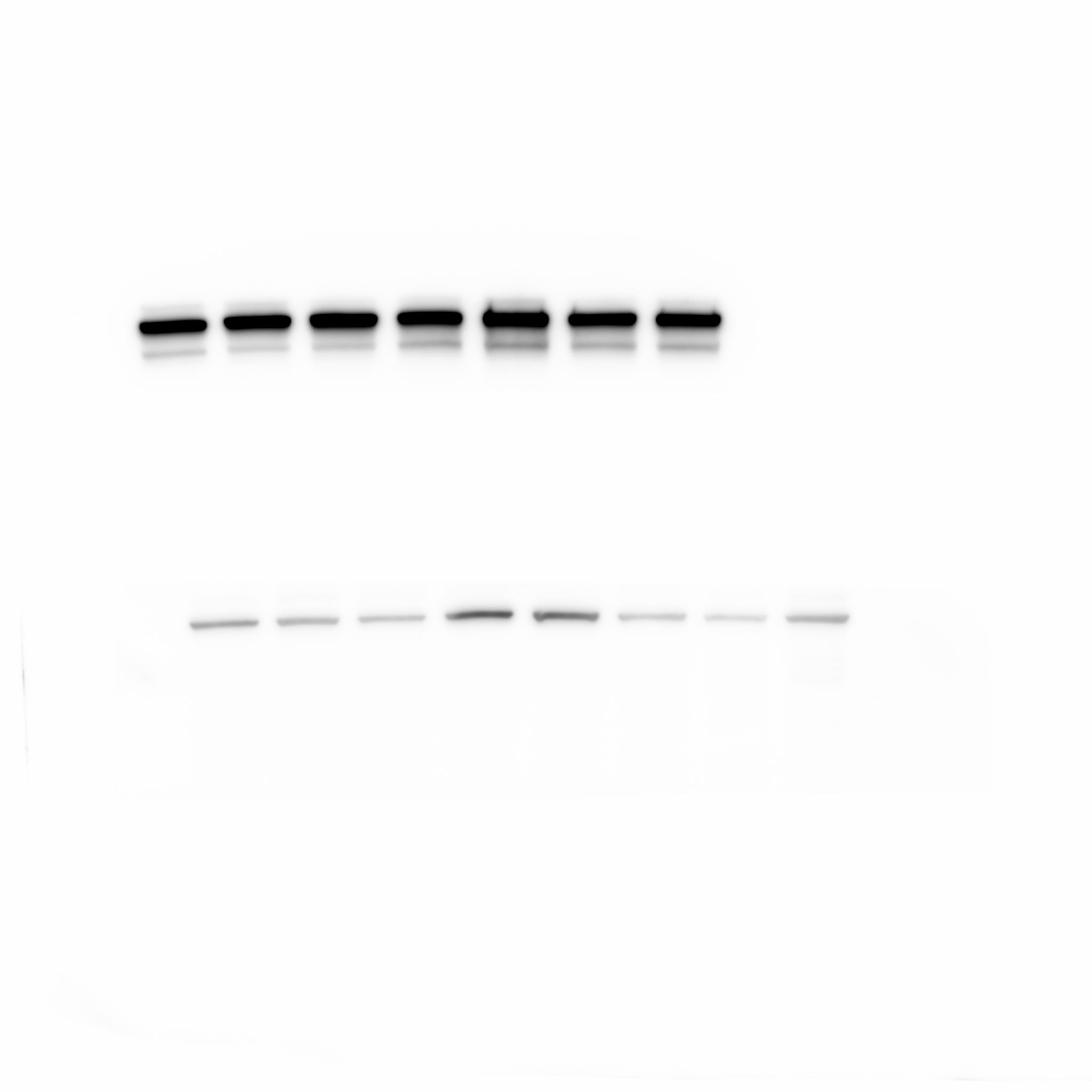

Supplement: Source data 3. [file elife-70151-data3.zip › Source data_v2/Figure 4C/MCF7/Figure 4C_HTR2C in MCF7_source data.jpg]

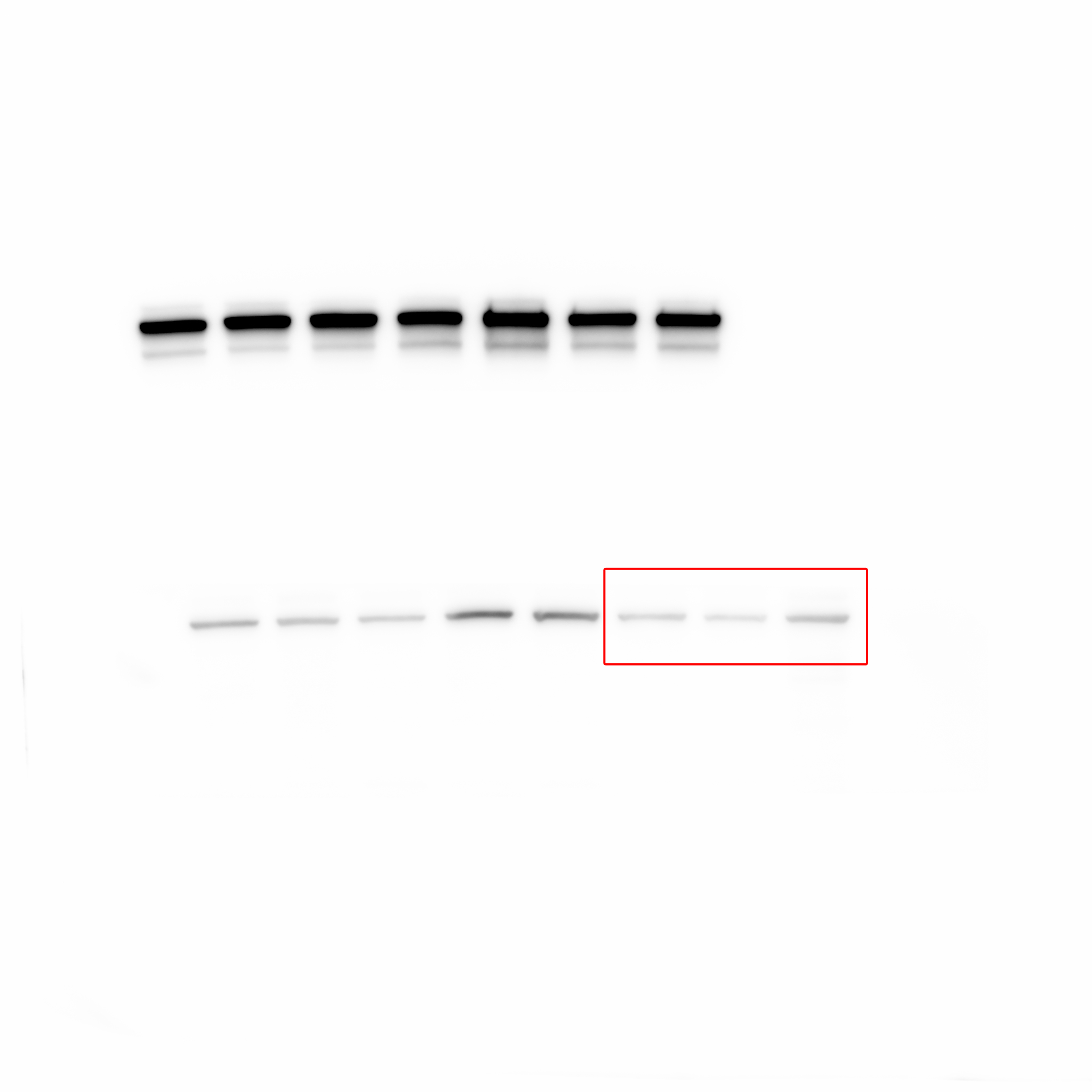

Supplement: Source data 3. [file elife-70151-data3.zip › Source data_v2/Figure 4C/MCF7/Figure 4C_HTR2C in MCF7_source data_labelled.jpg]

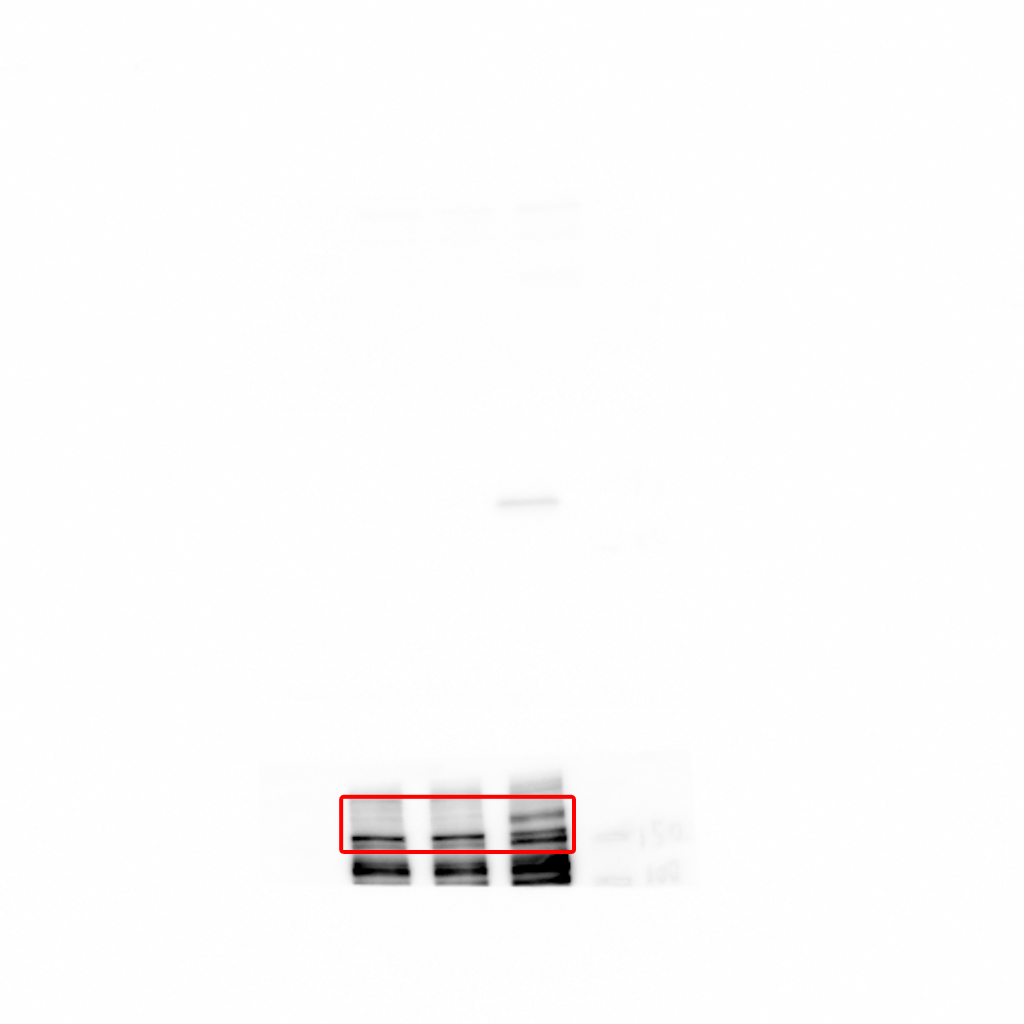

Supplement: Source data 3. [file elife-70151-data3.zip › Source data_v2/Figure 4C/MCF7/Figure 4C_ Zeb1 in MCF7_source data_labelled.jpg]

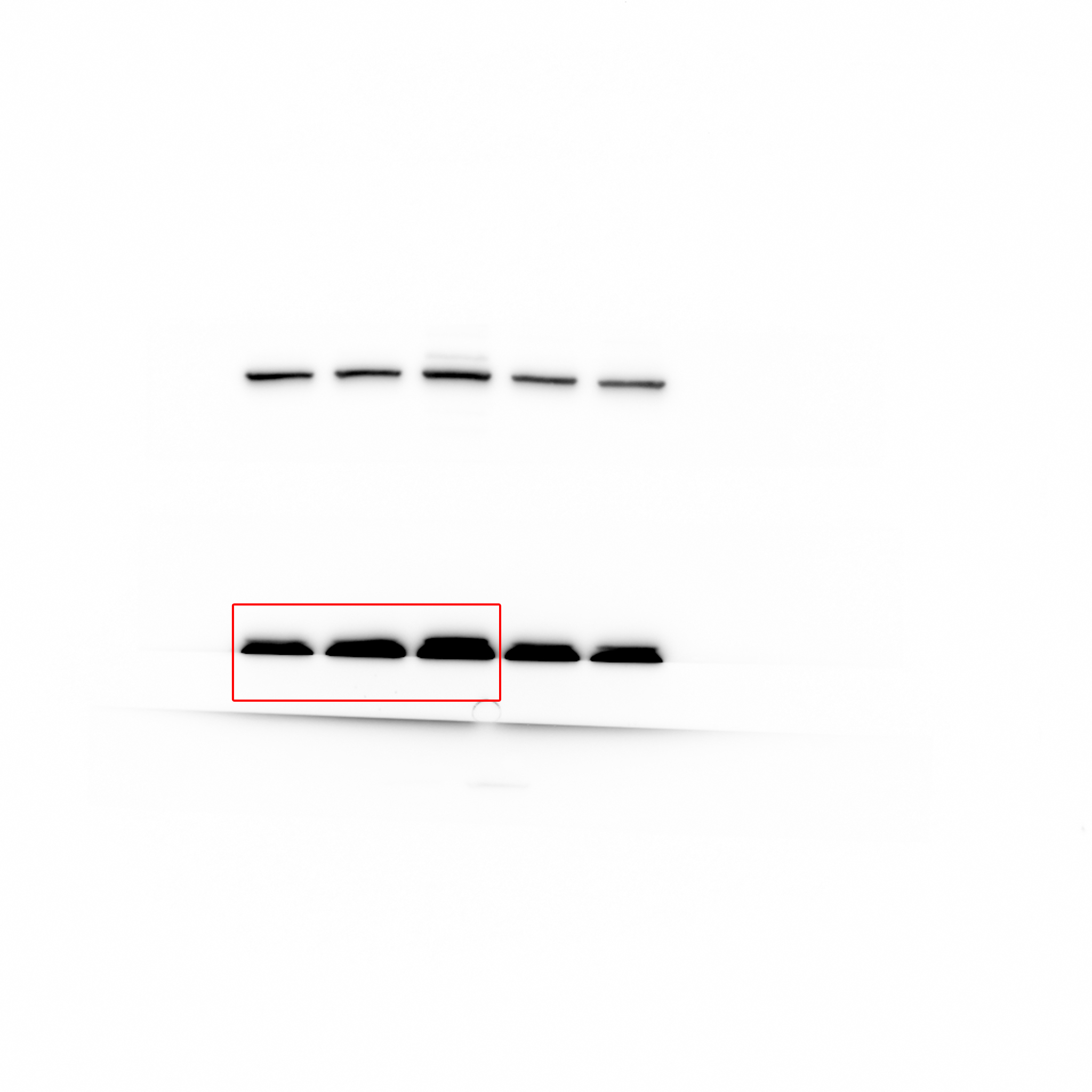

Supplement: Source data 3. [file elife-70151-data3.zip › Source data_v2/Figure 4C/MCF7/Figure 4C_GAPDH in MCF7_source data_labelled.jpg]

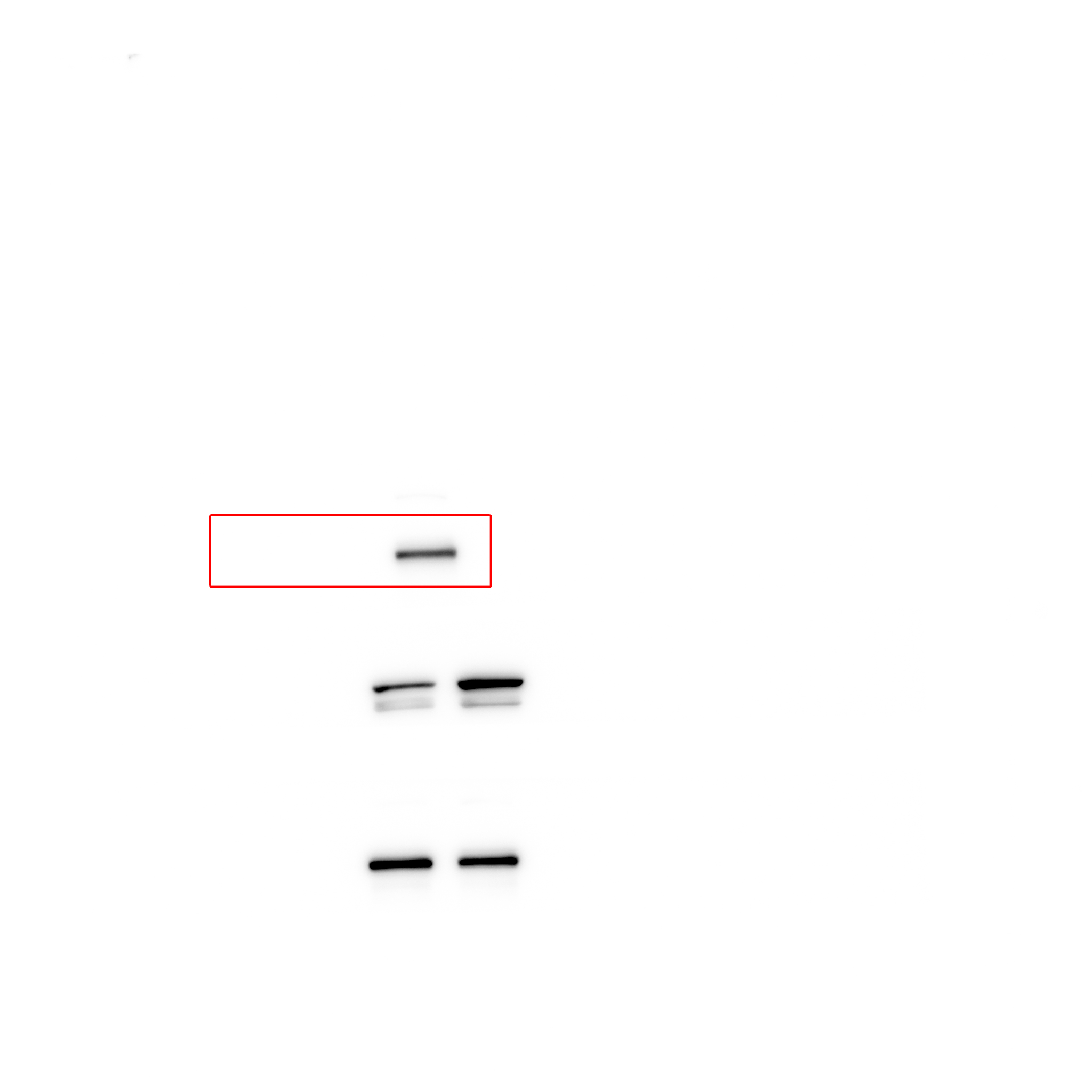

Supplement: Source data 3. [file elife-70151-data3.zip › Source data_v2/Figure 4C/MCF7/Figure 4C_N-cadherin in MCF7_source data.labelled]

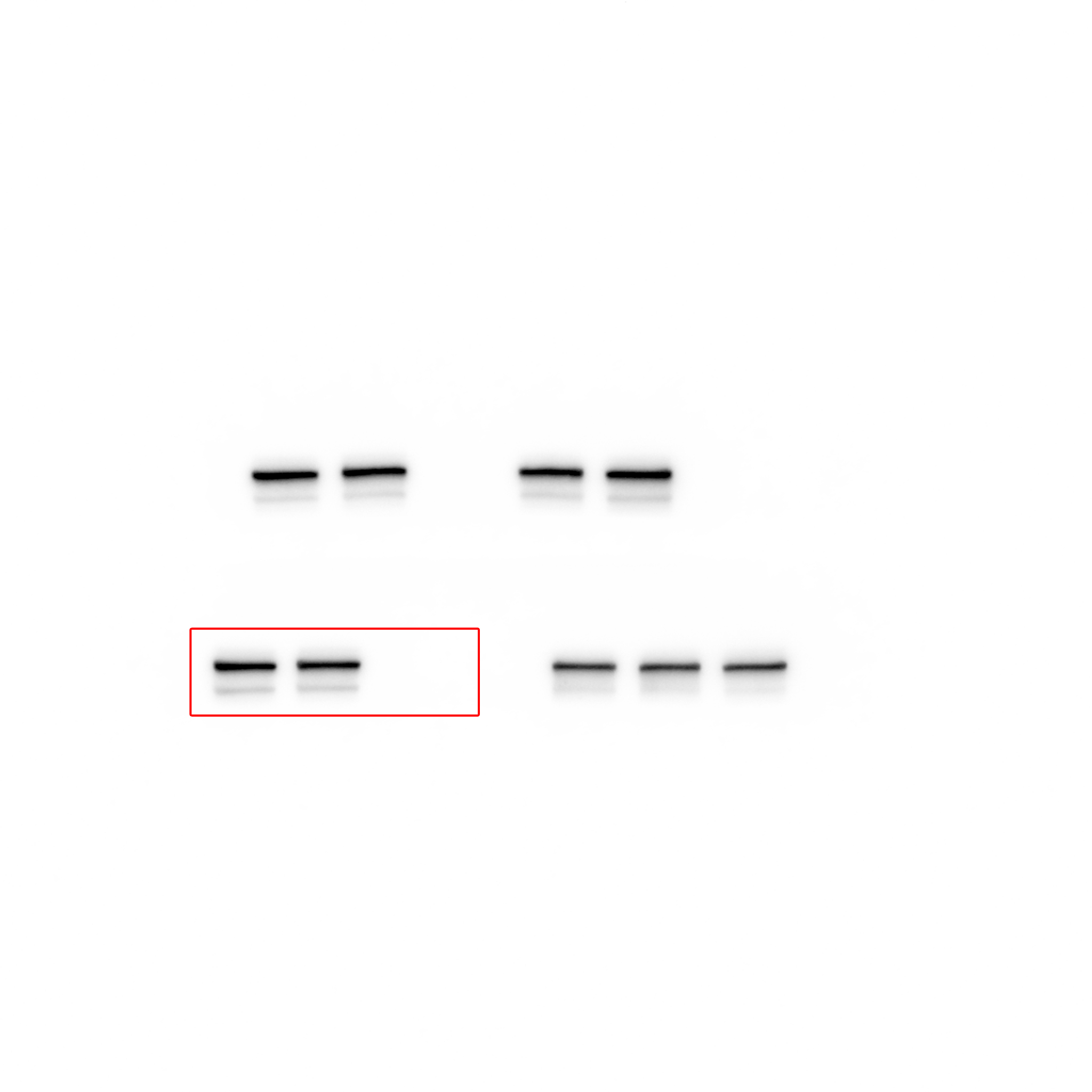

Supplement: Source data 3. [file elife-70151-data3.zip › Source data_v2/Figure 4C/MCF7/Figure 4C_E-cadherin in MCF7_source data_labelled.jpg]
