# Supplementary figures and images for: A zebrafish embryo screen utilizing gastrulation identifies the HTR2C inhibitor pizotifen as a suppressor of EMT-mediated metastasis (part 1 of 2)
Source: eLife. 2021 Dec 17;10:e70151. doi: 10.7554/eLife.70151 (PMC8824480; doi:10.7554/eLife.70151)

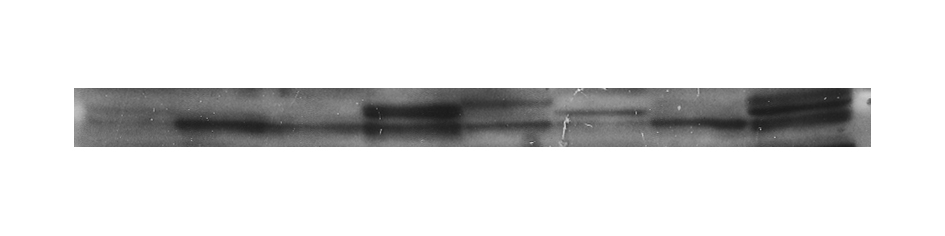

Supplement: Source data 3. [file elife-70151-data3.zip › Source data_v2/Figure 2-figure supplement 1/Figure S2A_DRD2_source data.jpg]

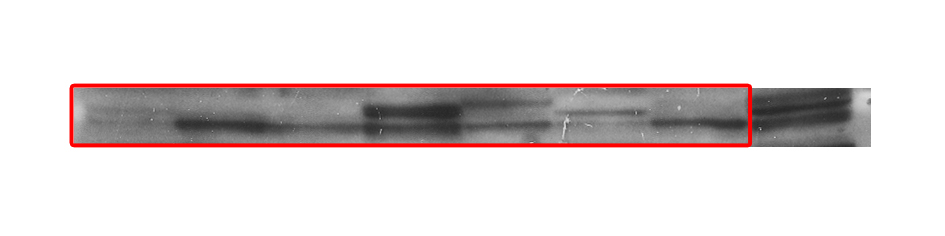

Supplement: Source data 3. [file elife-70151-data3.zip › Source data_v2/Figure 2-figure supplement 1/Figure S2A_DRD2_source data.labelled]

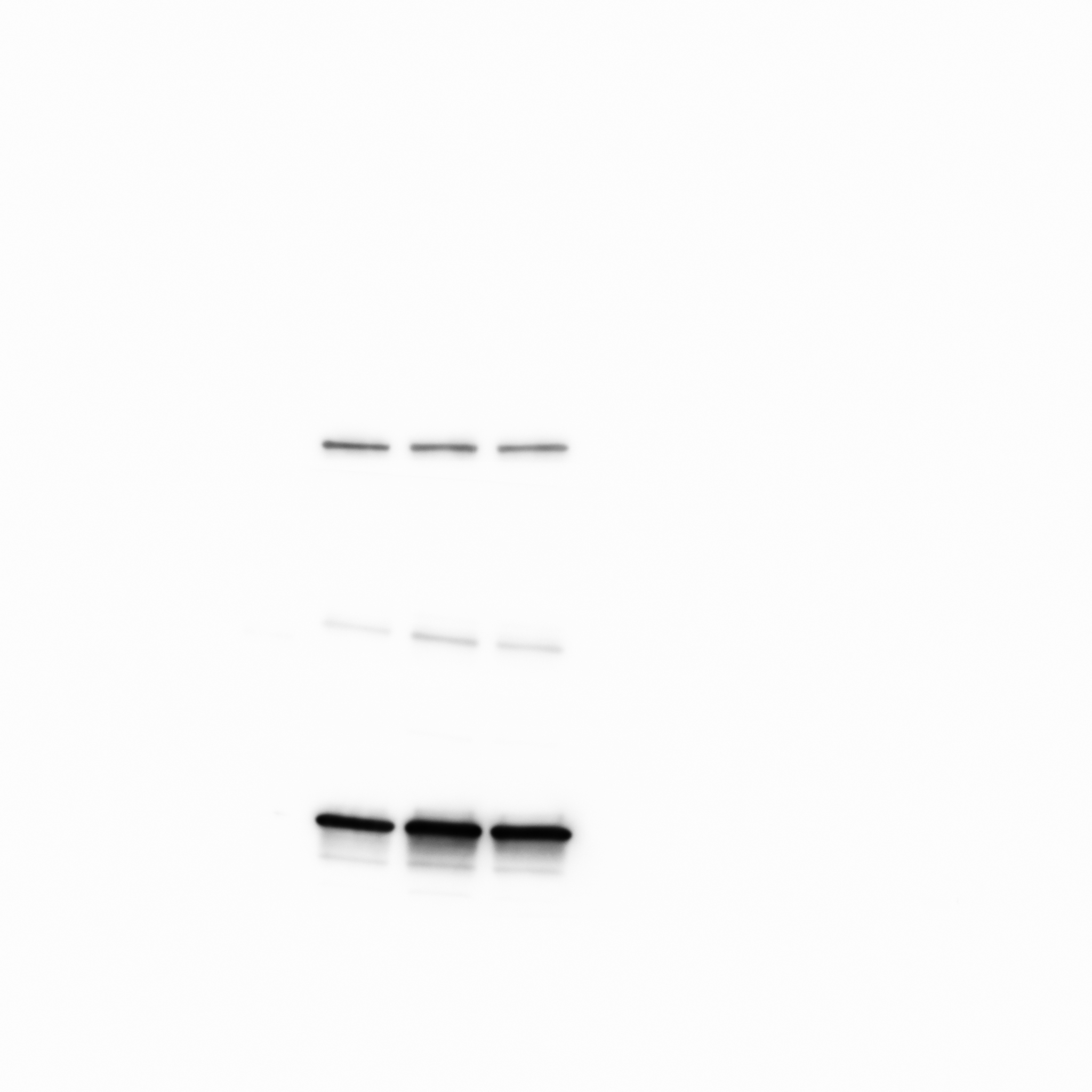

Supplement: Source data 3. [file elife-70151-data3.zip › Source data_v2/Figure 5F/Figure 5F_GAPDH_source data.jpg]

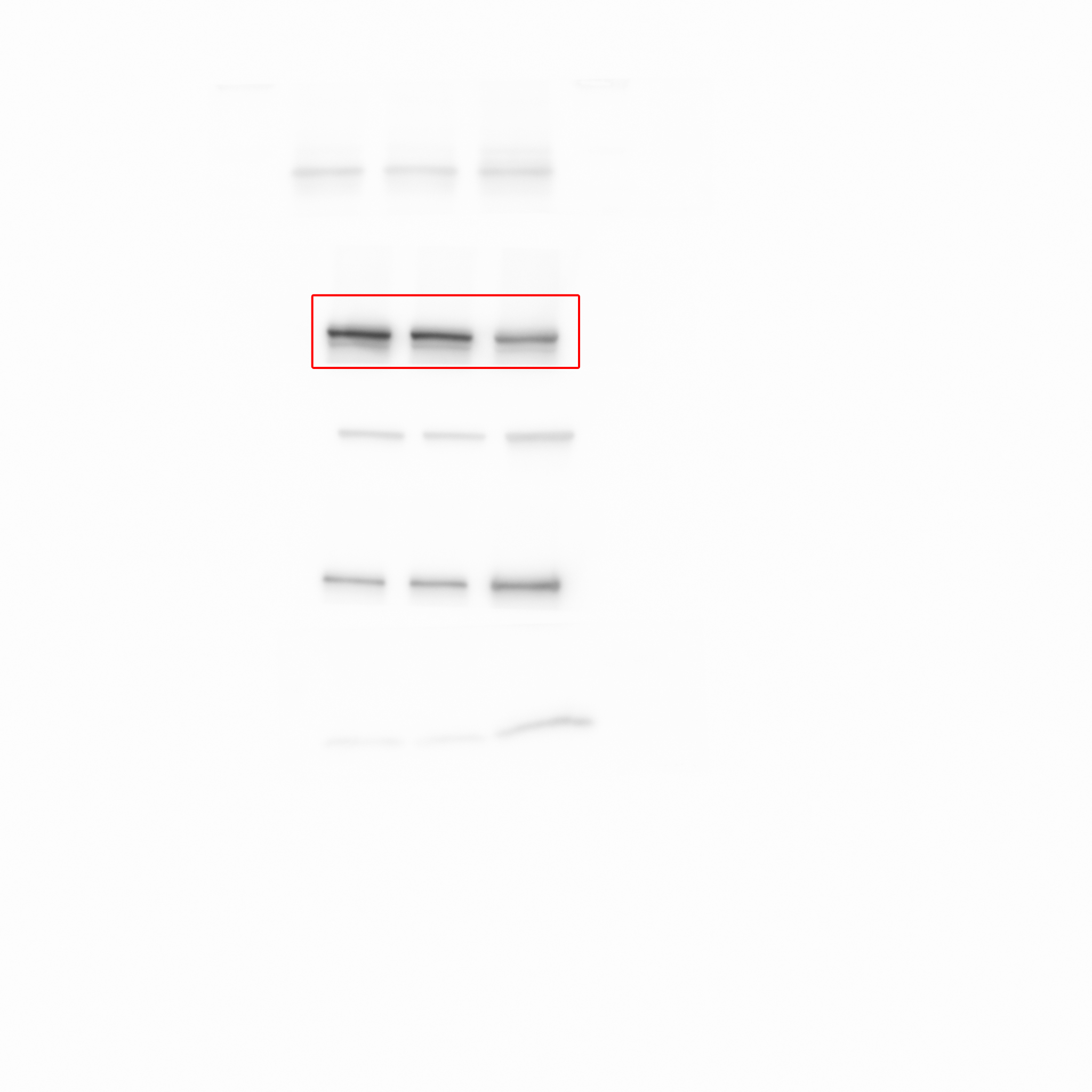

Supplement: Source data 3. [file elife-70151-data3.zip › Source data_v2/Figure 5F/Figure 5F_b-catenin_cytoplasm_source data_labelled.jpg]

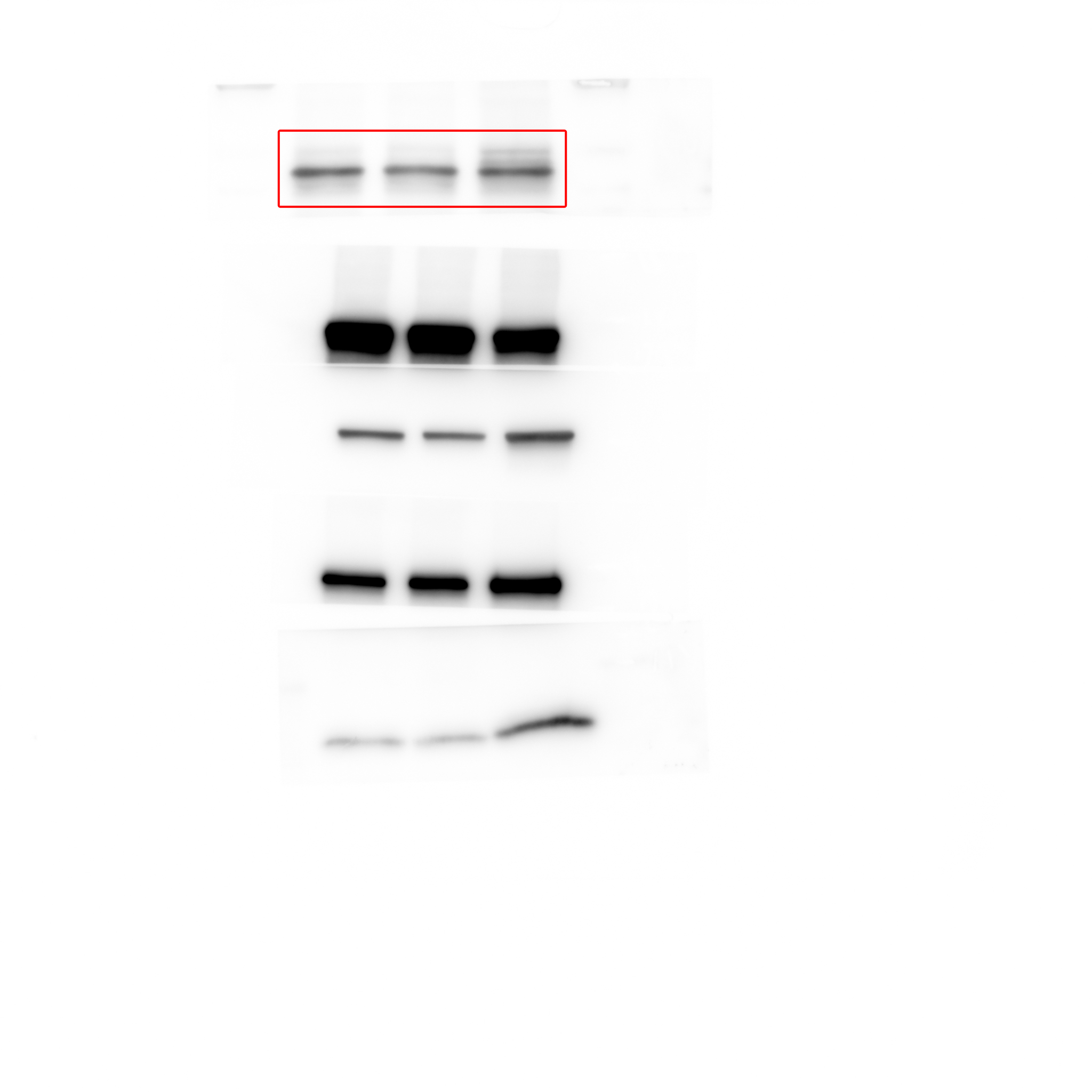

Supplement: Source data 3. [file elife-70151-data3.zip › Source data_v2/Figure 5F/Figure 5F_GSK3b_source data_labelled.jpg]

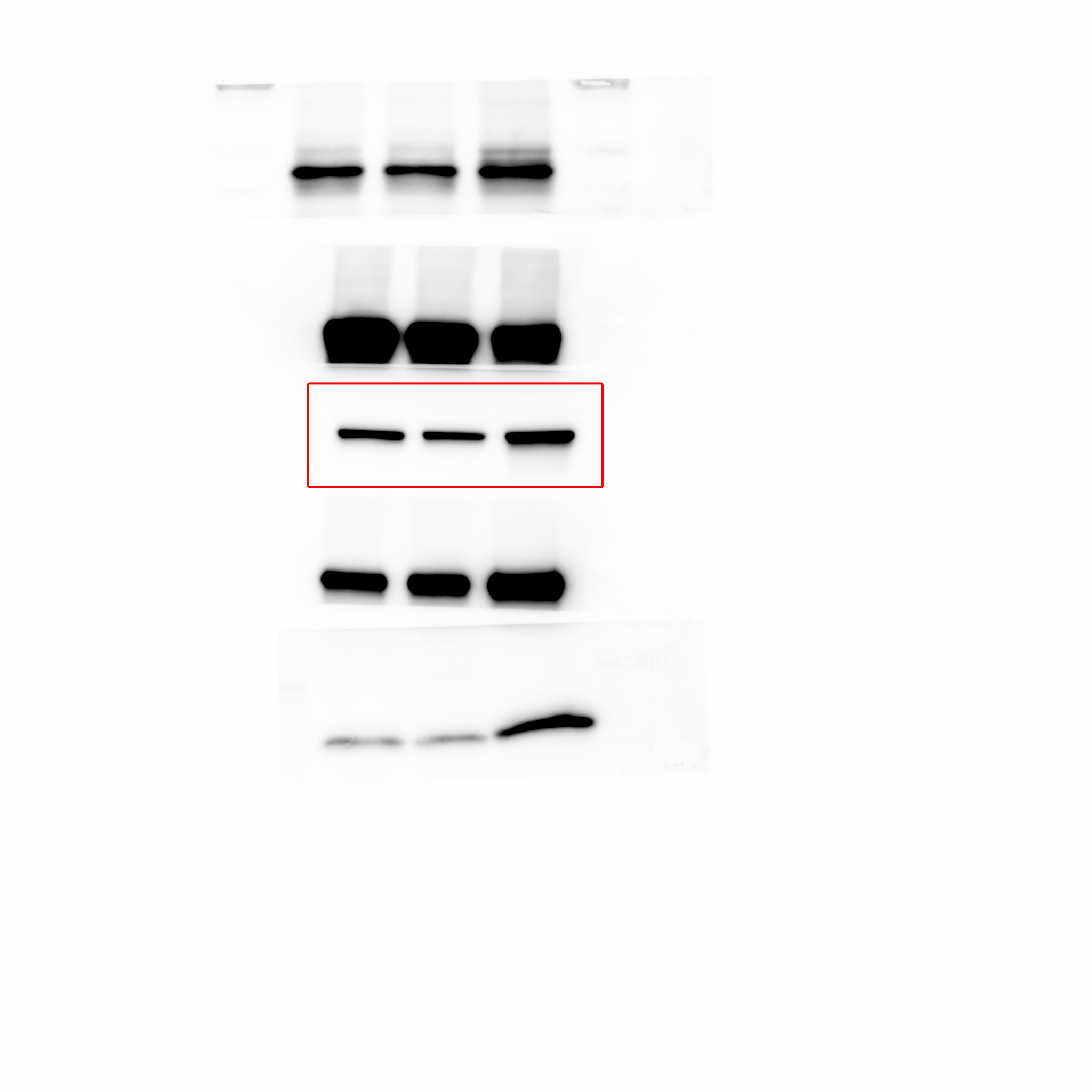

Supplement: Source data 3. [file elife-70151-data3.zip › Source data_v2/Figure 5F/Figure 5F_b-tubulin_source data_labelled.jpg]

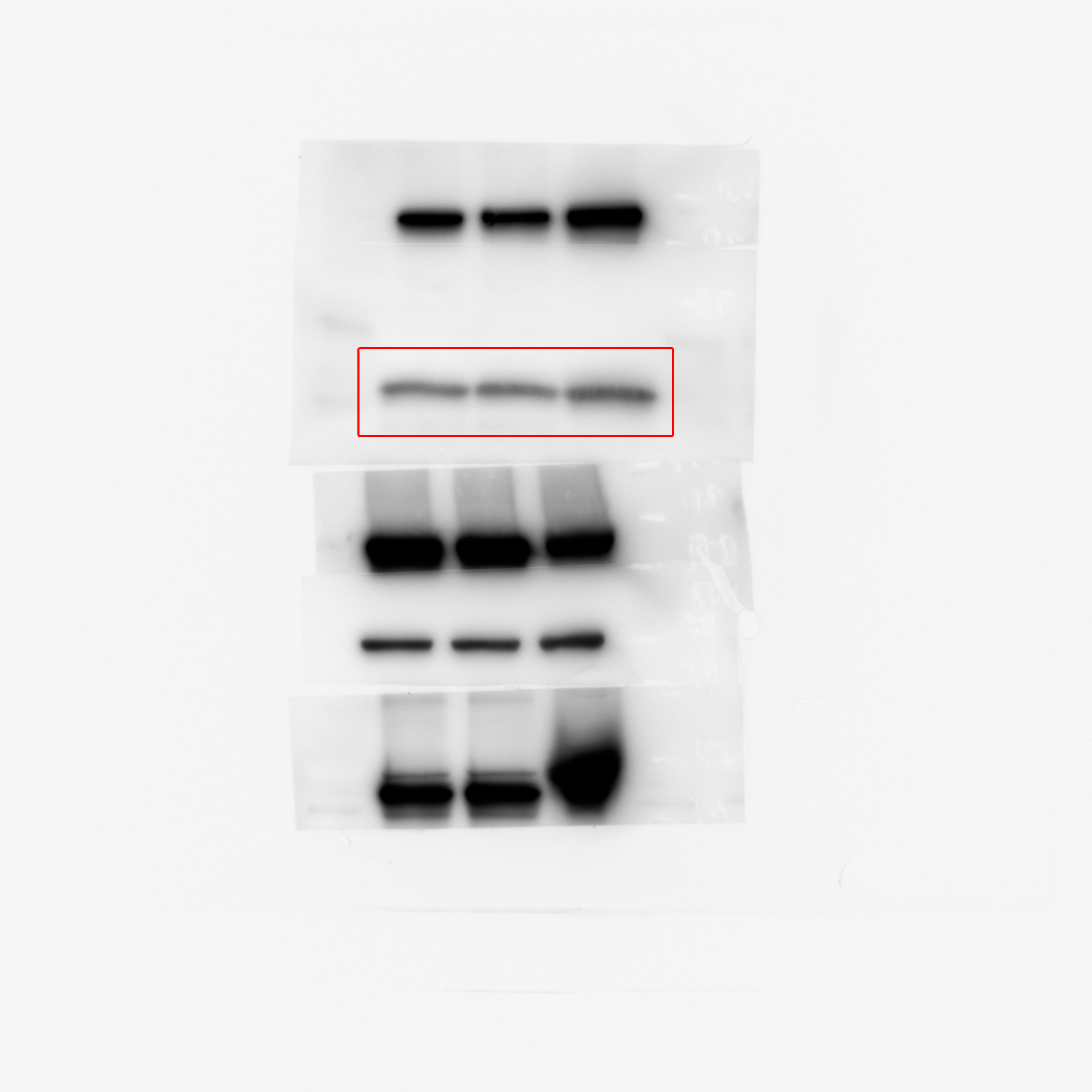

Supplement: Source data 3. [file elife-70151-data3.zip › Source data_v2/Figure 5F/Figure 5F_Histone H3_source data_labelled.jpg]

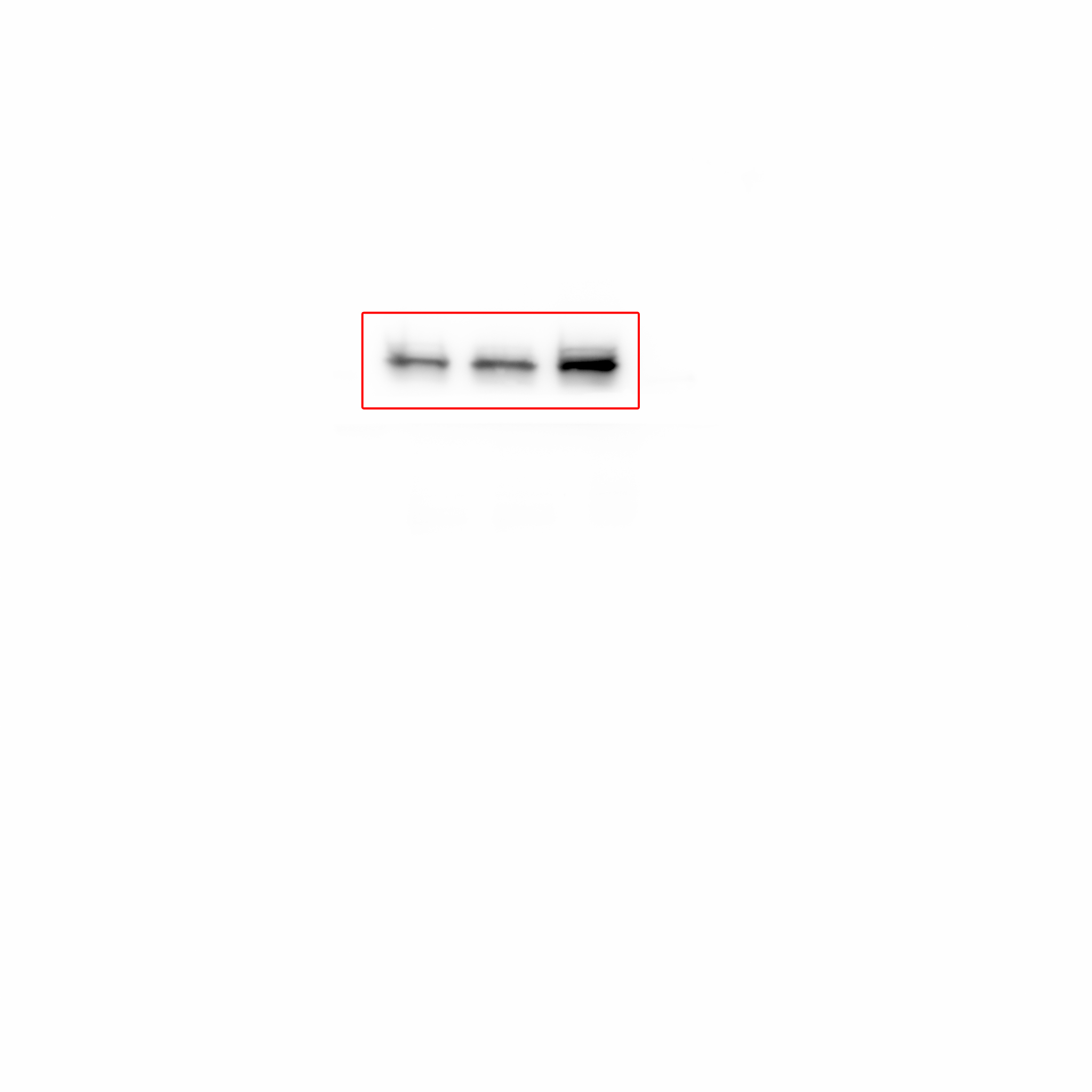

Supplement: Source data 3. [file elife-70151-data3.zip › Source data_v2/Figure 5F/Figure 5F_p-GSK3b_source data_labelled.jpg]

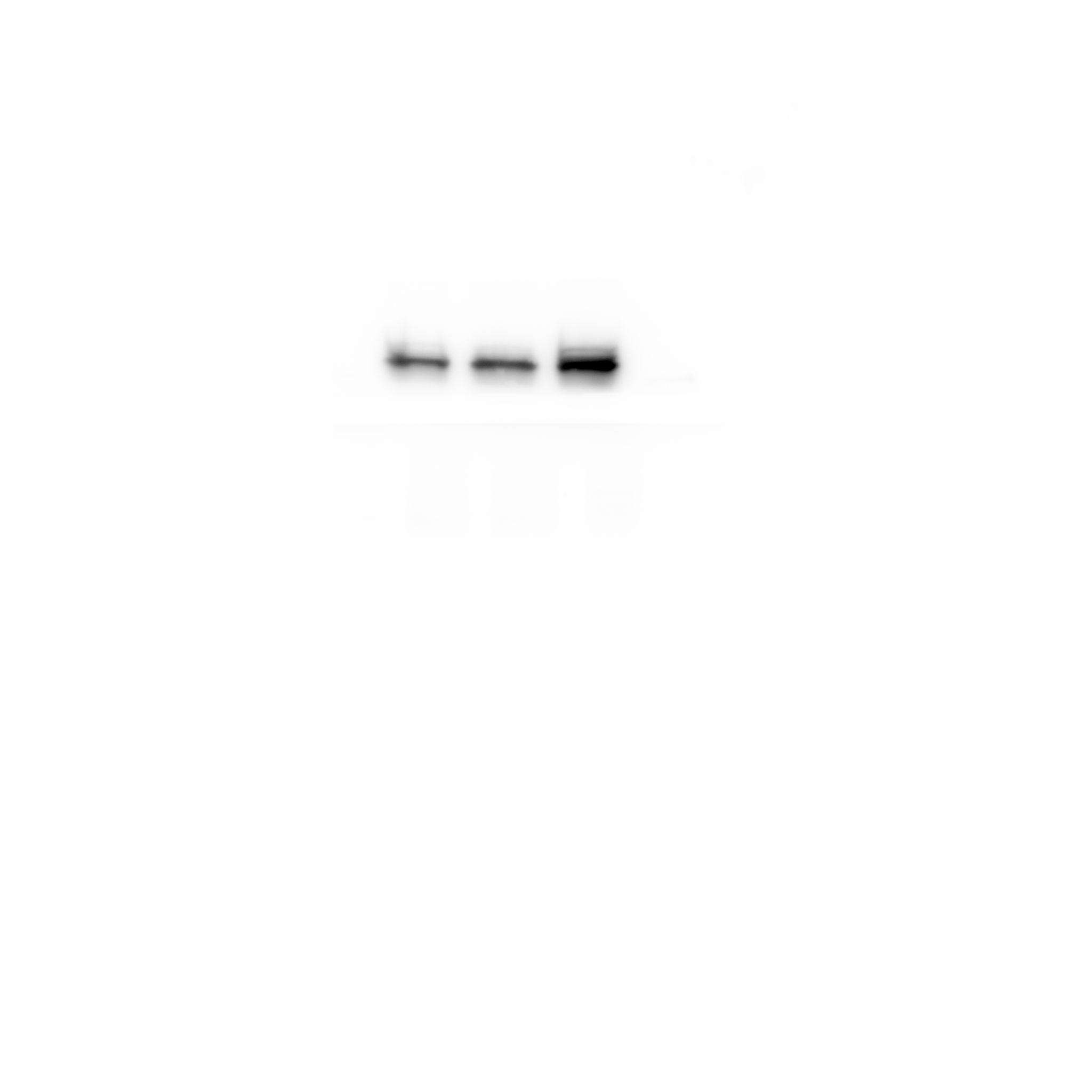

Supplement: Source data 3. [file elife-70151-data3.zip › Source data_v2/Figure 5F/Figure 5F_p-GSK3b_source data.jpg]

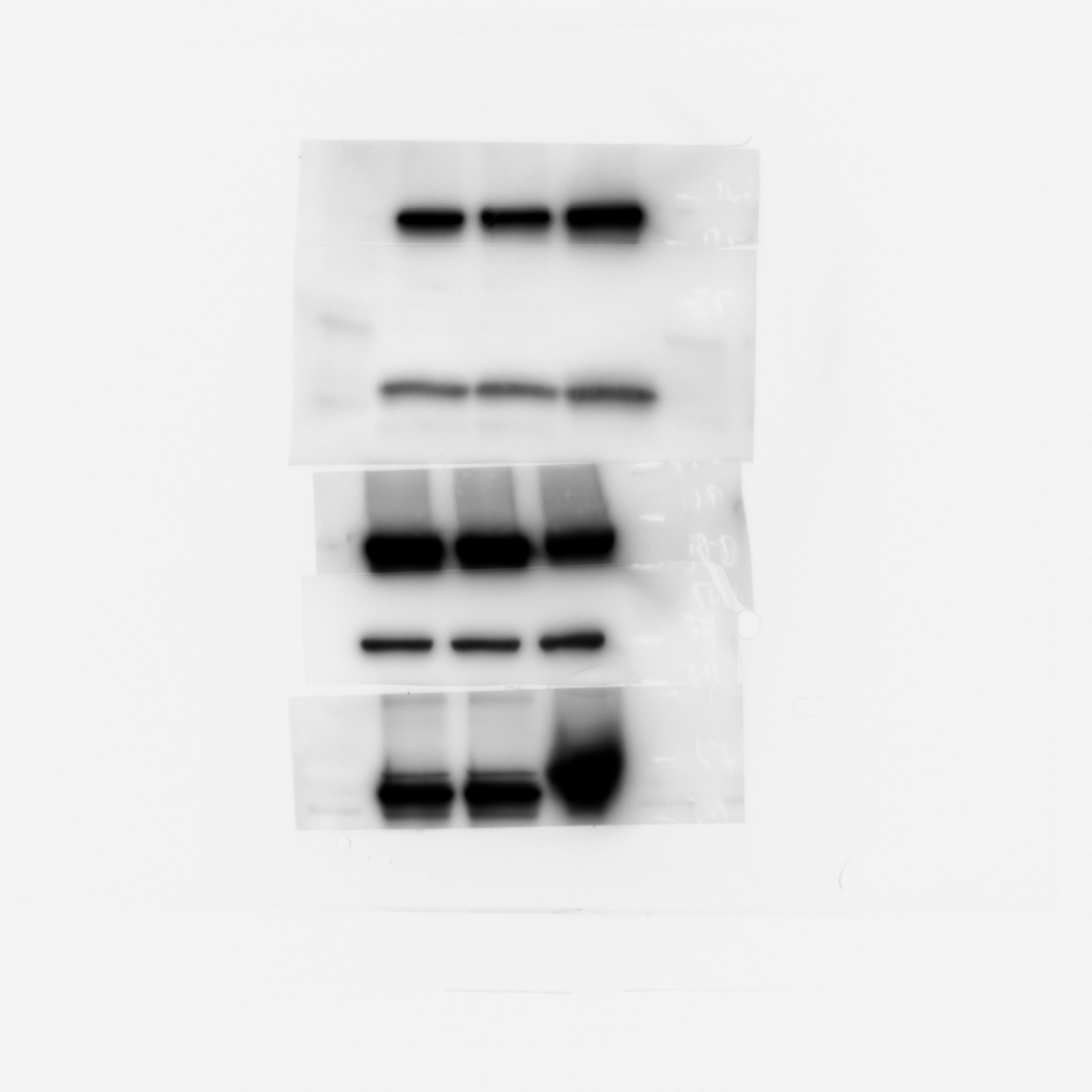

Supplement: Source data 3. [file elife-70151-data3.zip › Source data_v2/Figure 5F/Figure 5F_Histone H3_source data.jpg]

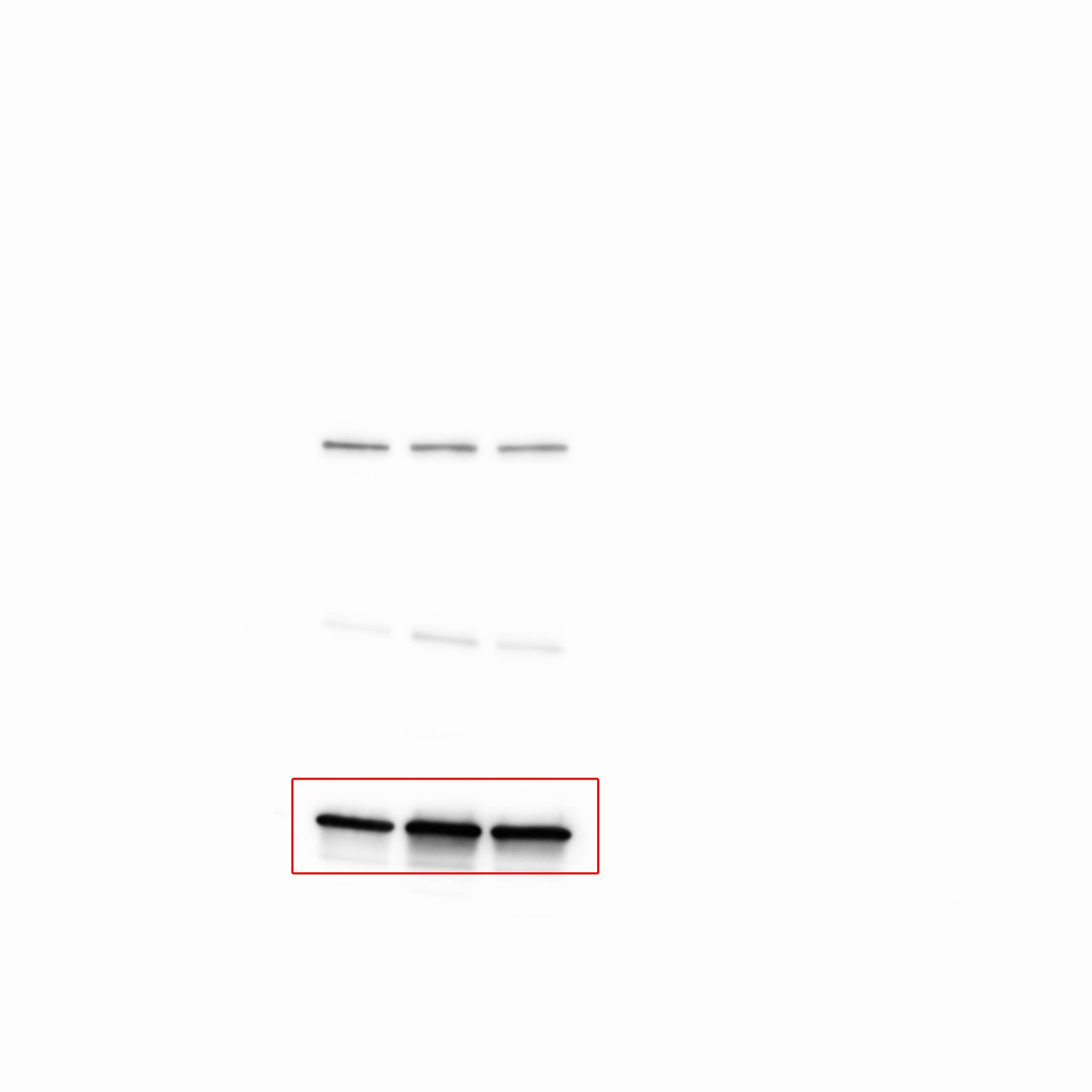

Supplement: Source data 3. [file elife-70151-data3.zip › Source data_v2/Figure 5F/Figure 5F_GAPDH_source data_labelled.jpg]

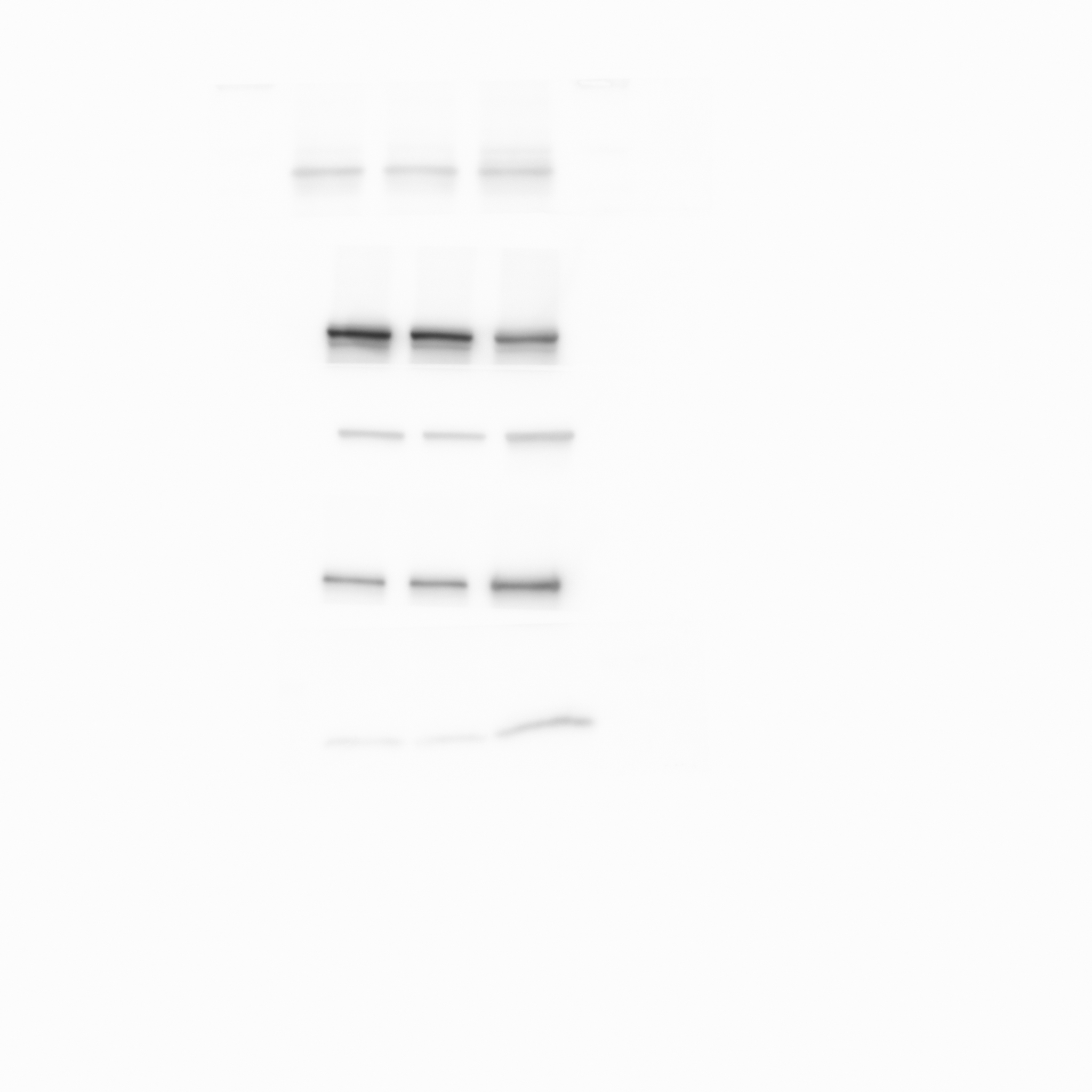

Supplement: Source data 3. [file elife-70151-data3.zip › Source data_v2/Figure 5F/Figure 5F_b-catenin in cytoplasm_source data.jpg]

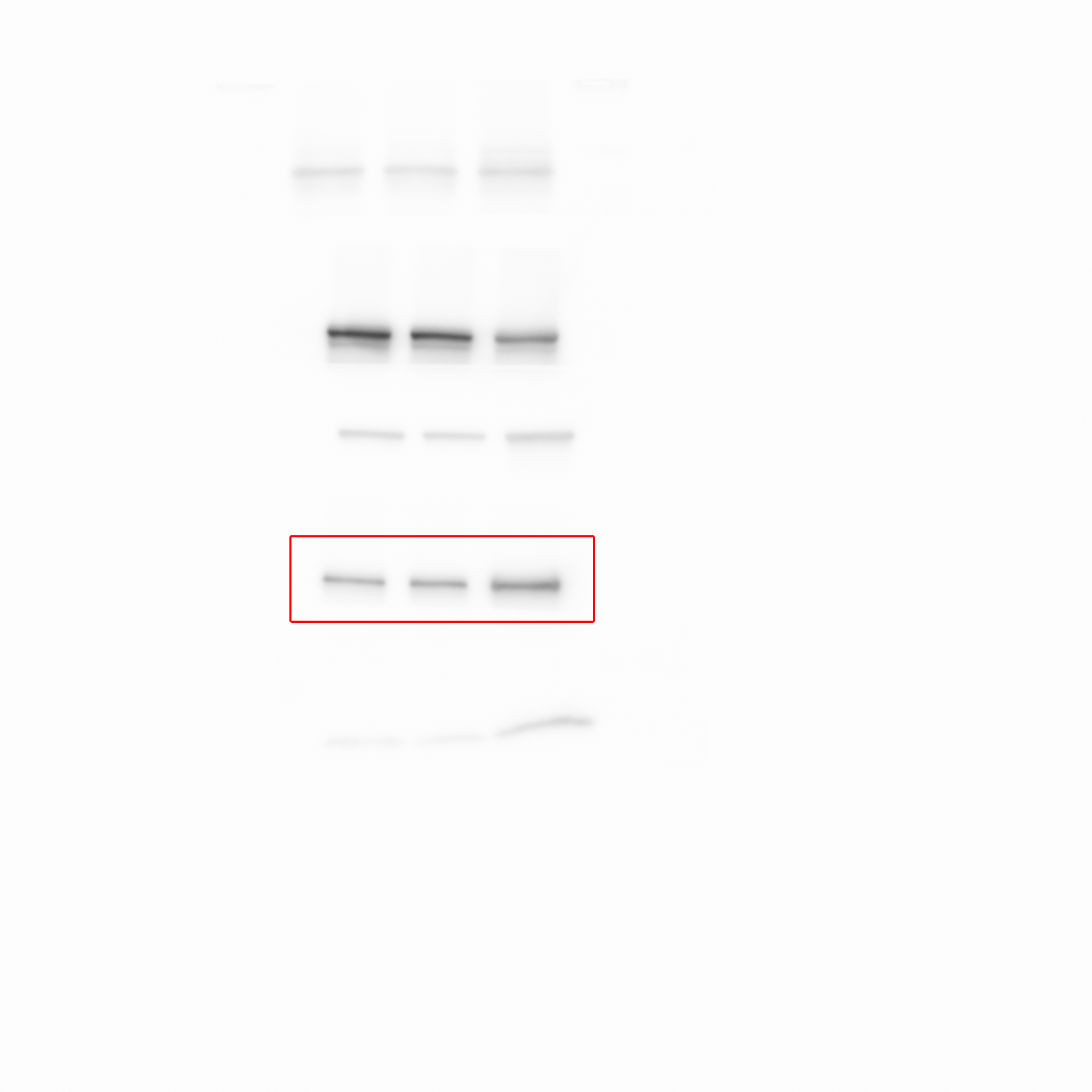

Supplement: Source data 3. [file elife-70151-data3.zip › Source data_v2/Figure 5F/Figure 5F_b-catenin_nucleus_source data_labelled.jpg]

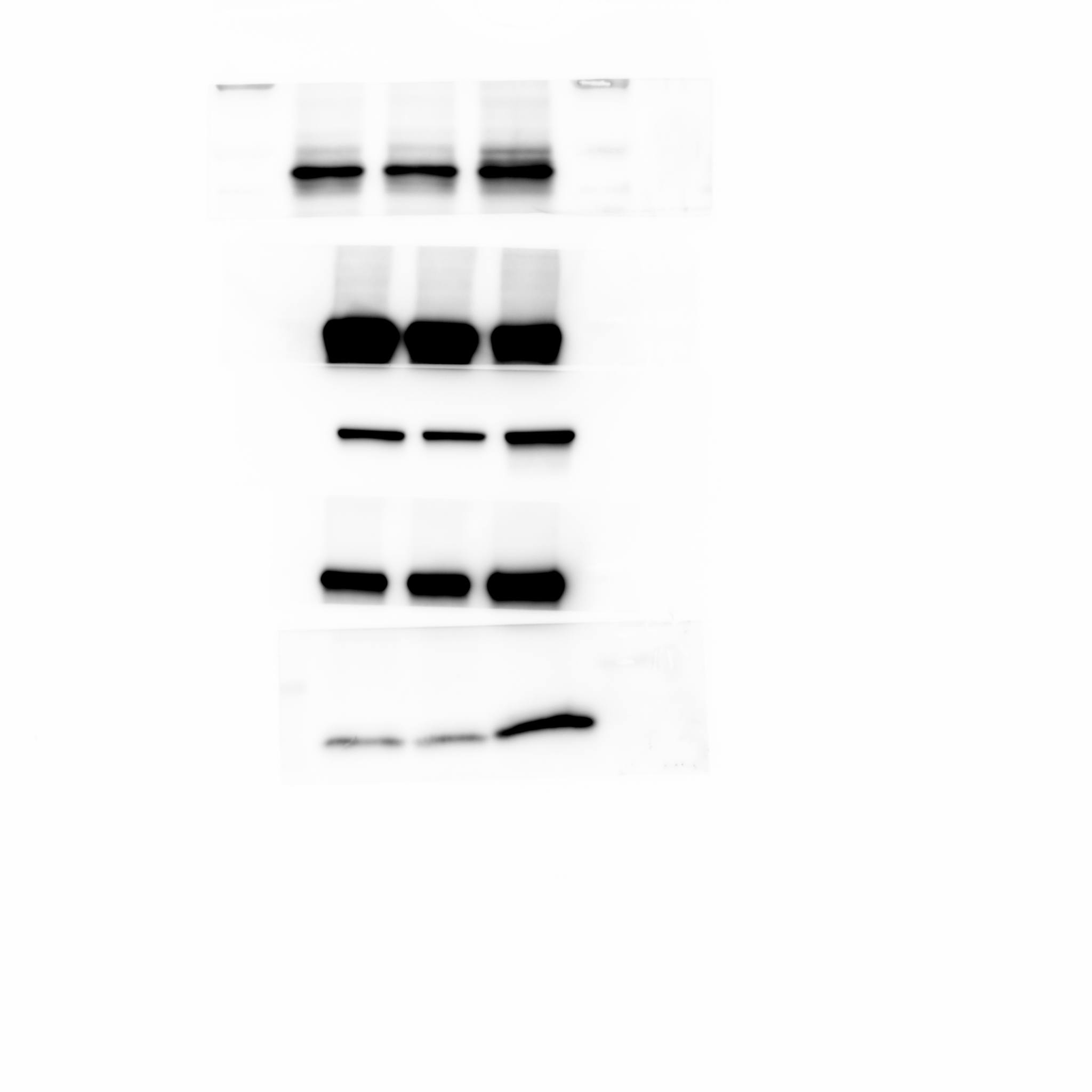

Supplement: Source data 3. [file elife-70151-data3.zip › Source data_v2/Figure 5F/Figure 5F_b-tubulin_source data.jpg]

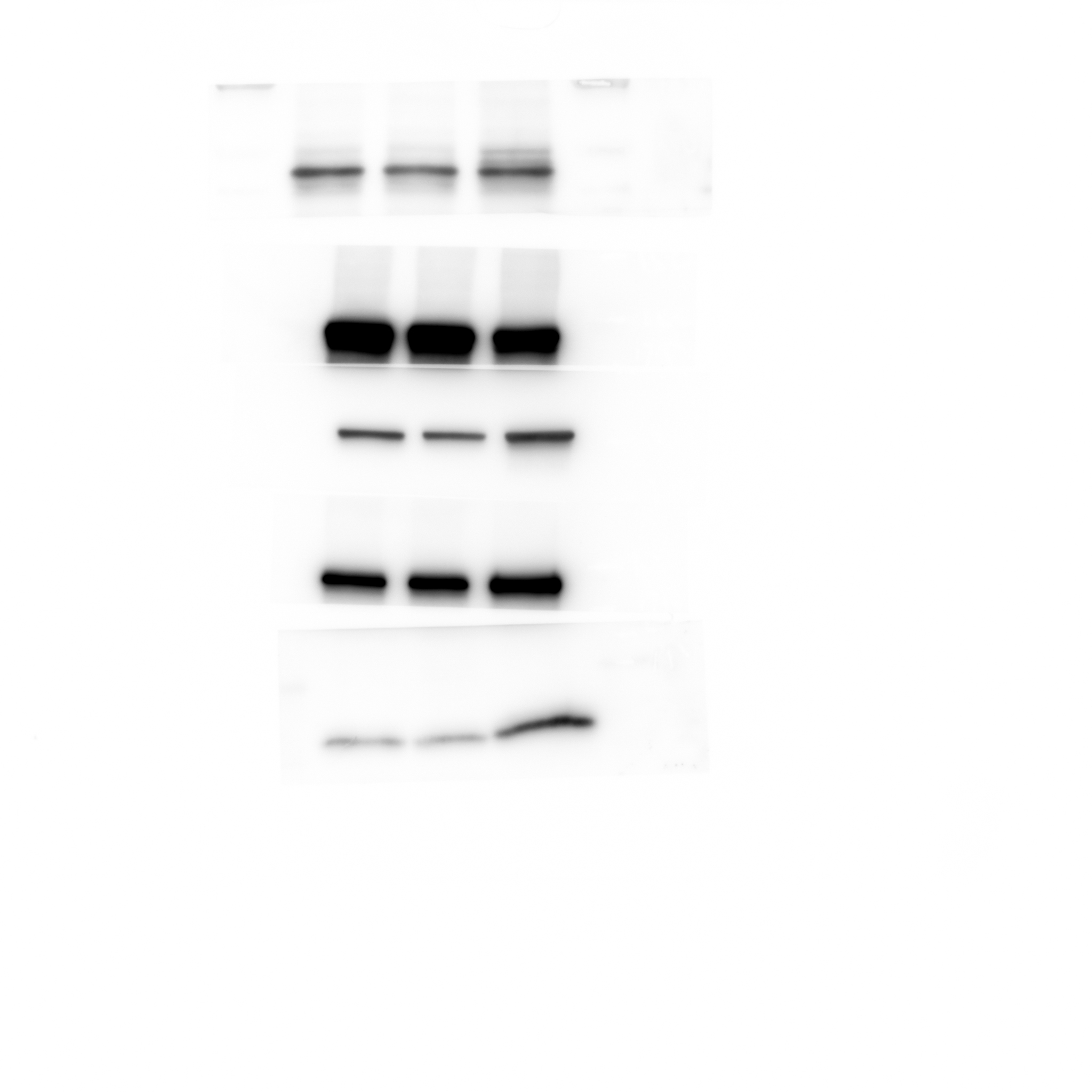

Supplement: Source data 3. [file elife-70151-data3.zip › Source data_v2/Figure 5F/Figure 5F_GSK3b_source data.jpg]

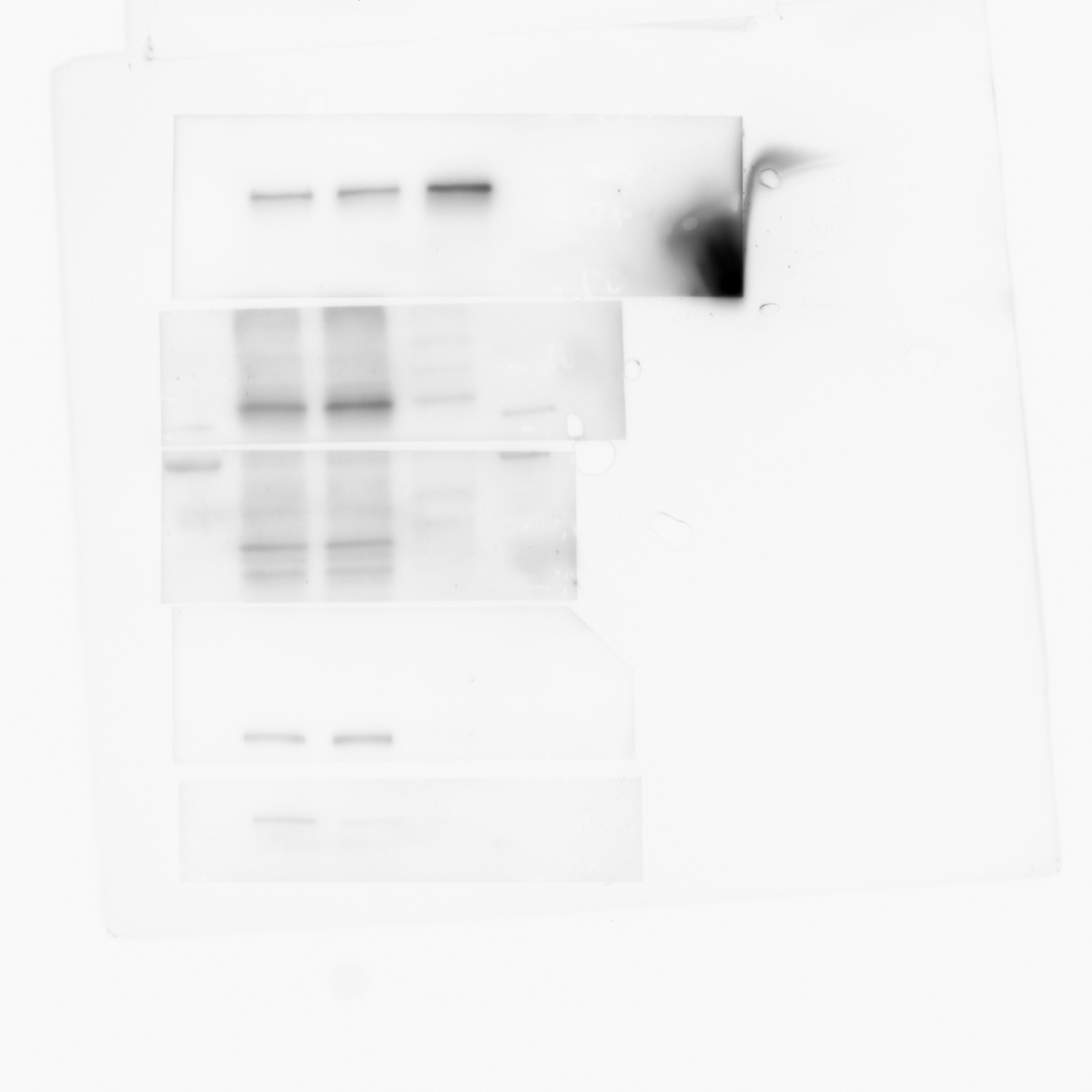

Supplement: Source data 3. [file elife-70151-data3.zip › Source data_v2/Figure 5H/Figure 5H_b-catenin_cytoplasm_source data.jpg]

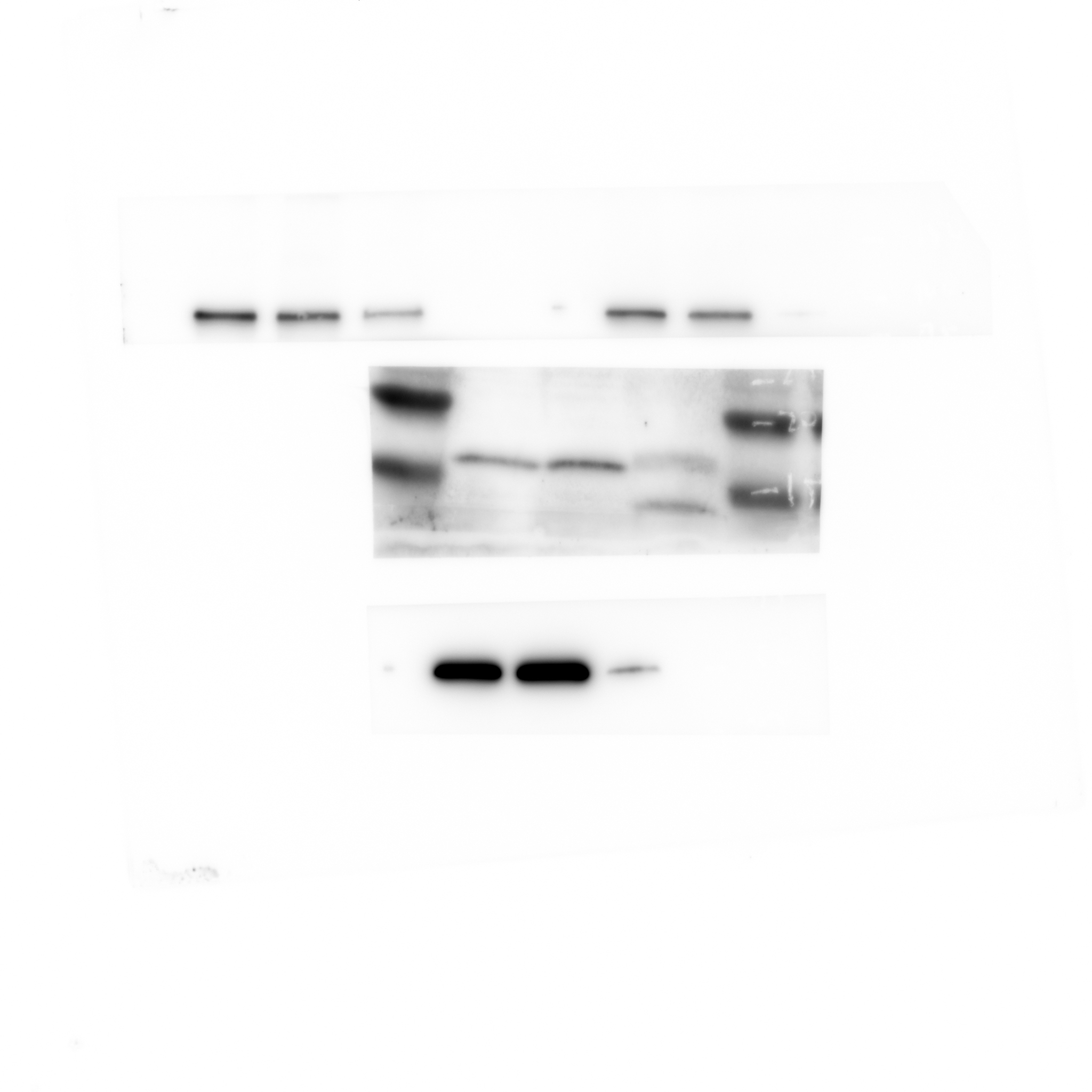

Supplement: Source data 3. [file elife-70151-data3.zip › Source data_v2/Figure 5H/Figure 5H_b-catenin_nuleus_source data.jpg]

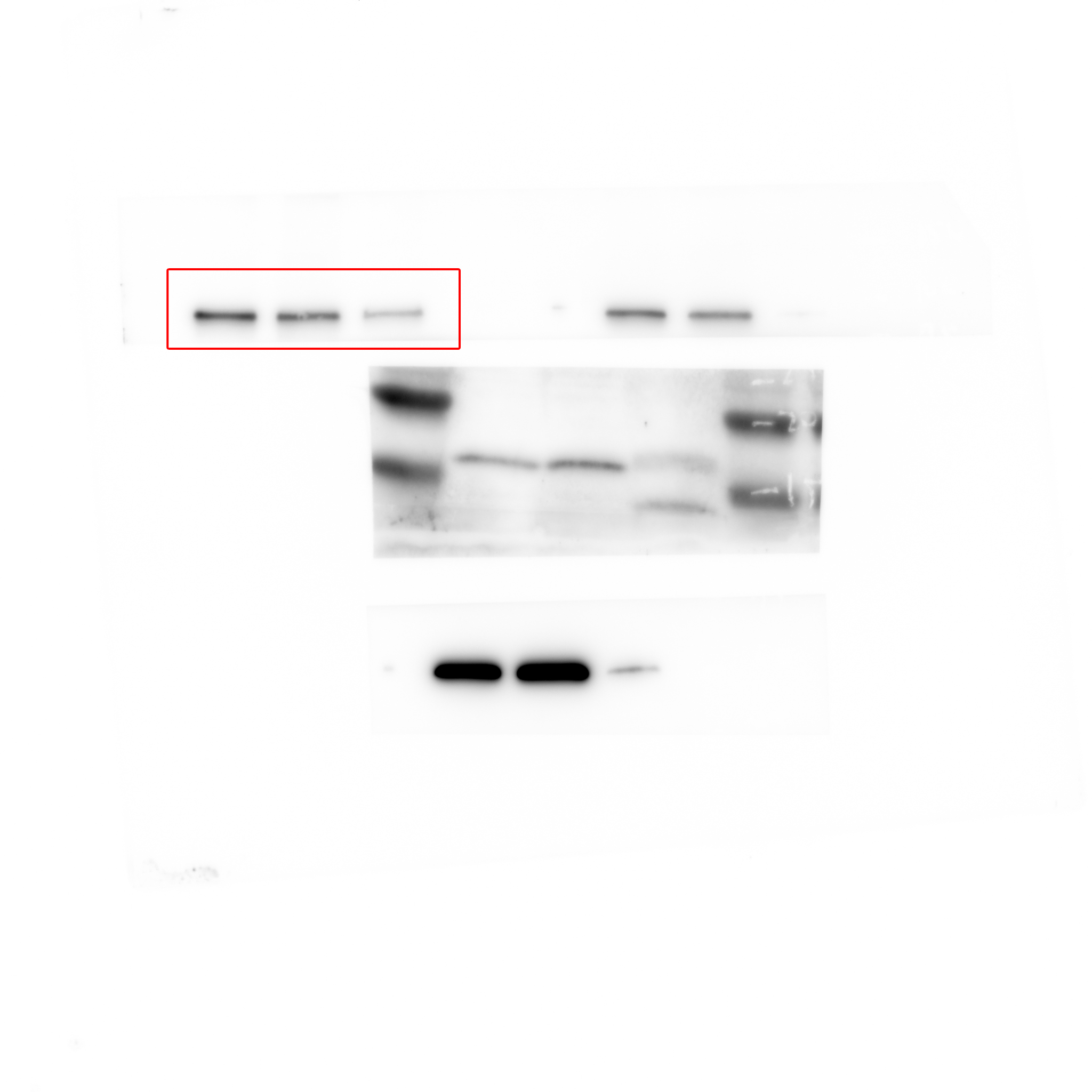

Supplement: Source data 3. [file elife-70151-data3.zip › Source data_v2/Figure 5H/Figure 5H_b-catenin_nuleus_source data_labelled.jpg]

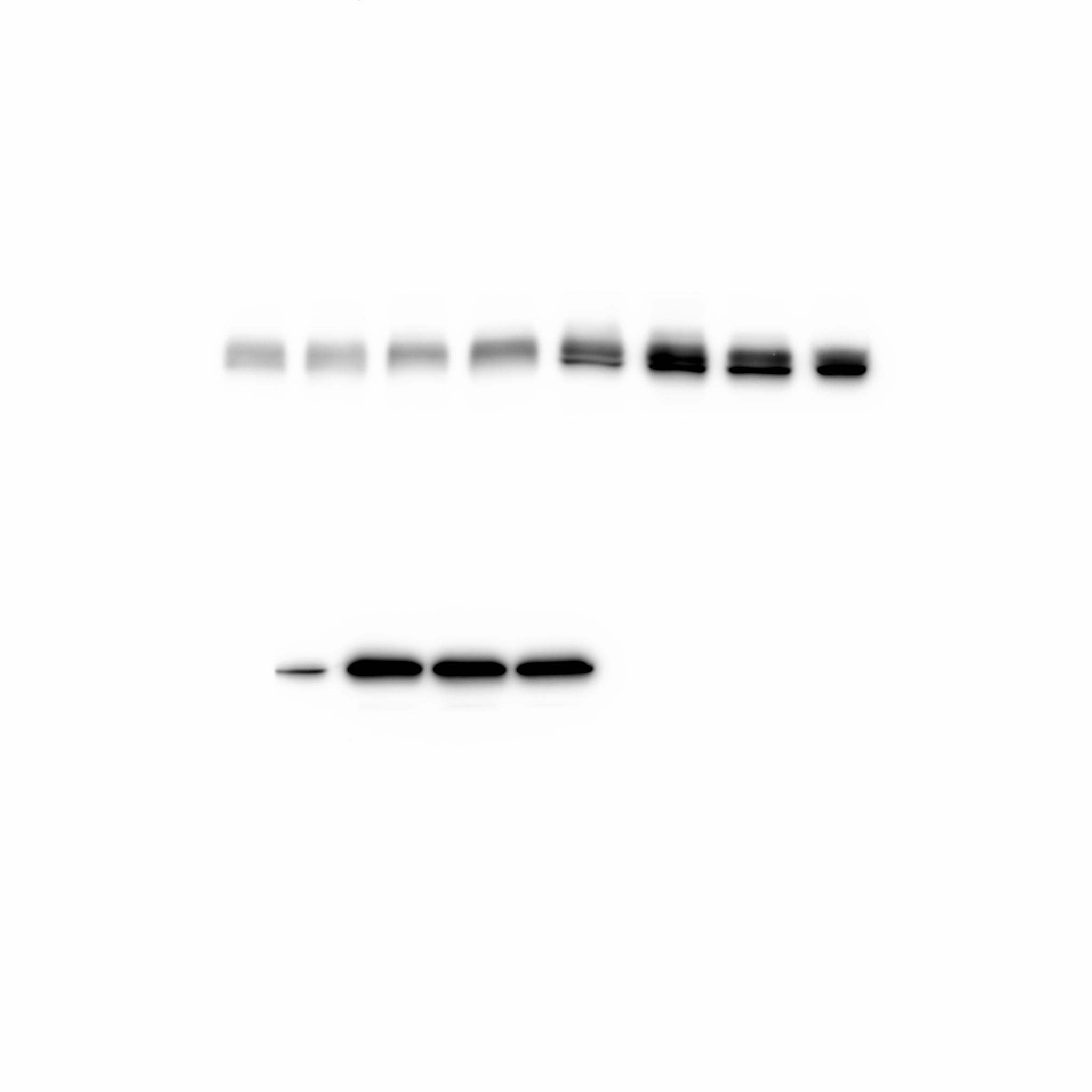

Supplement: Source data 3. [file elife-70151-data3.zip › Source data_v2/Figure 5H/Figure 5H_GAPDH_source data.jpg]

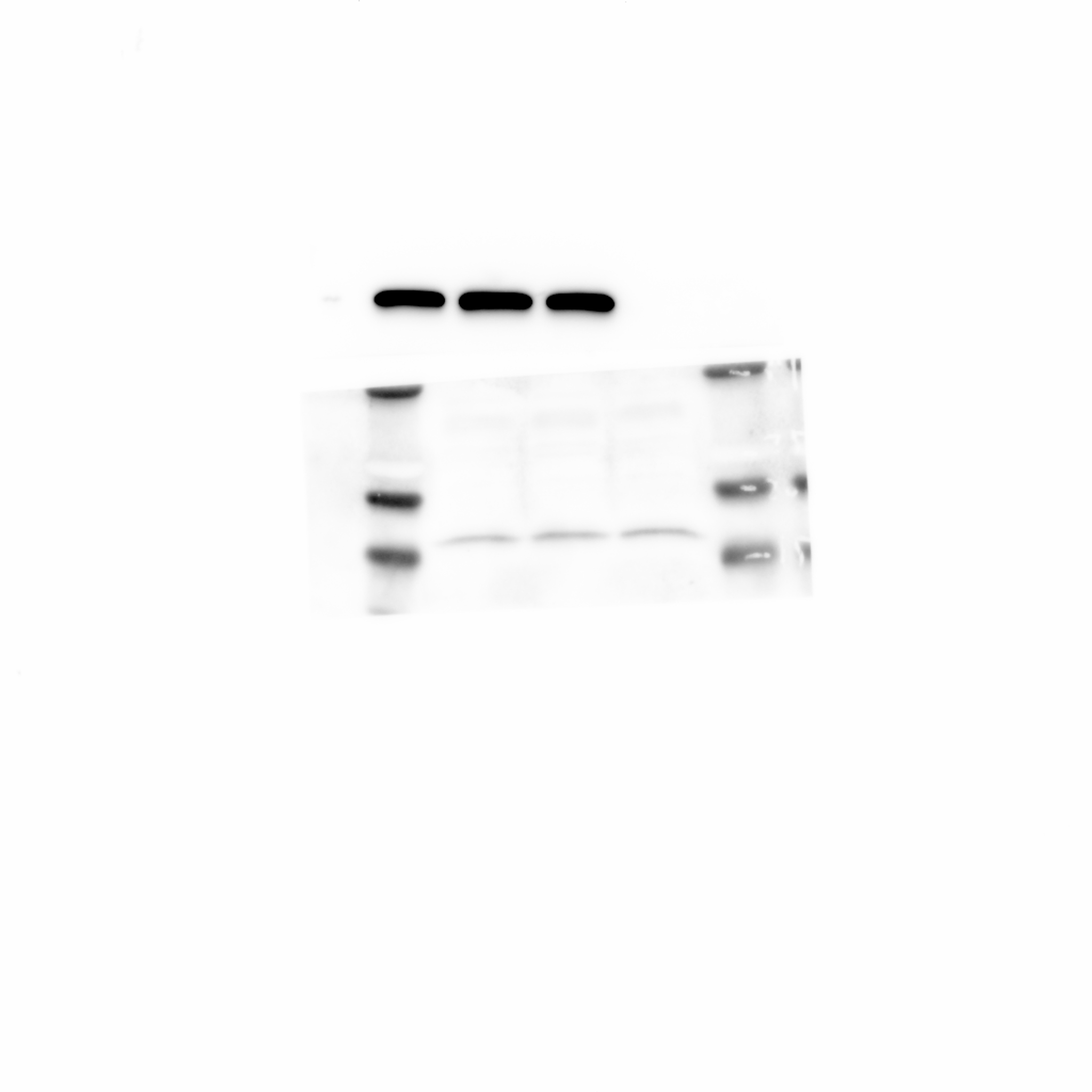

Supplement: Source data 3. [file elife-70151-data3.zip › Source data_v2/Figure 5H/Figure 5H_b-tubulin_source data.jpg]

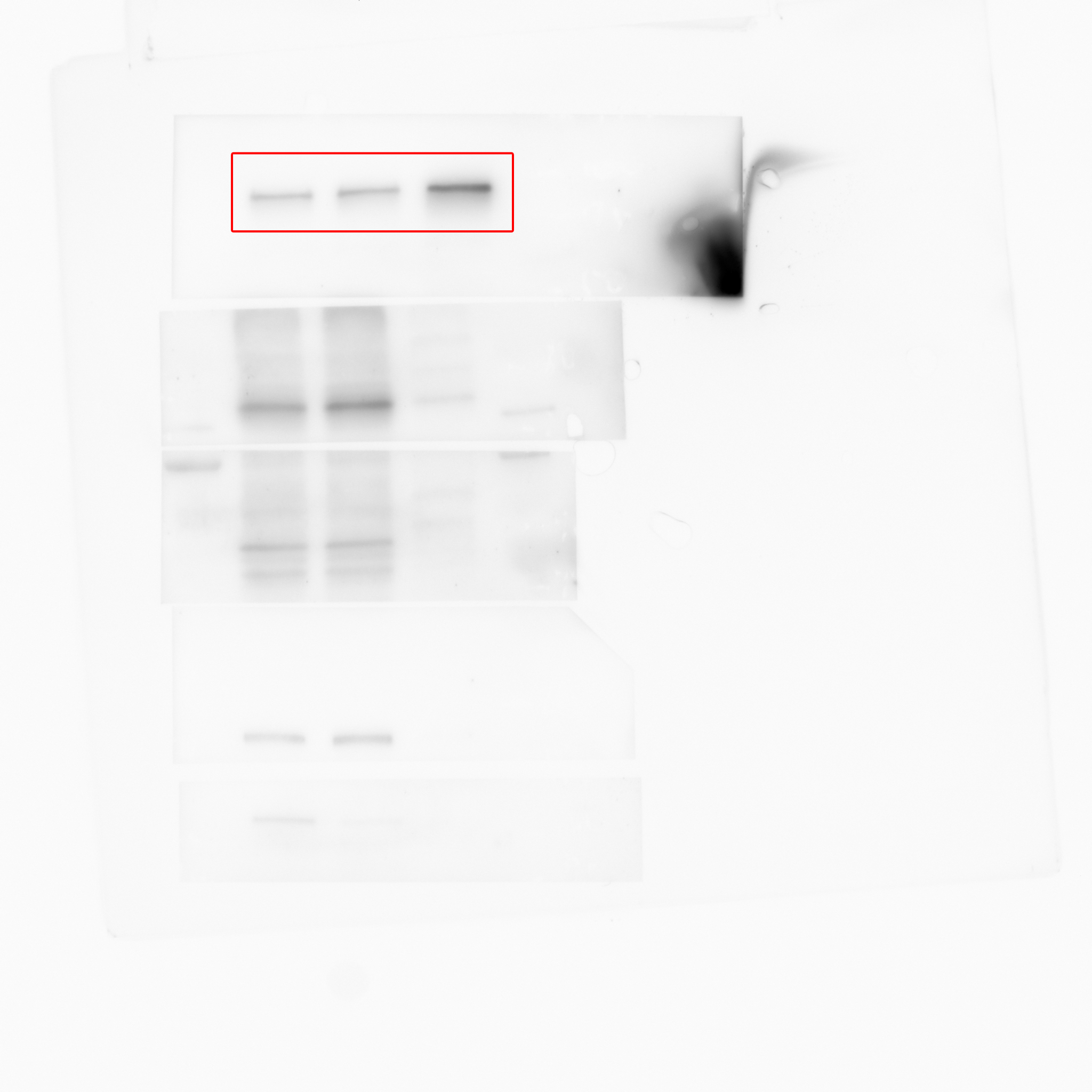

Supplement: Source data 3. [file elife-70151-data3.zip › Source data_v2/Figure 5H/Figure 5H_b-catenin_cytoplasm_source data_labelled.jpg]

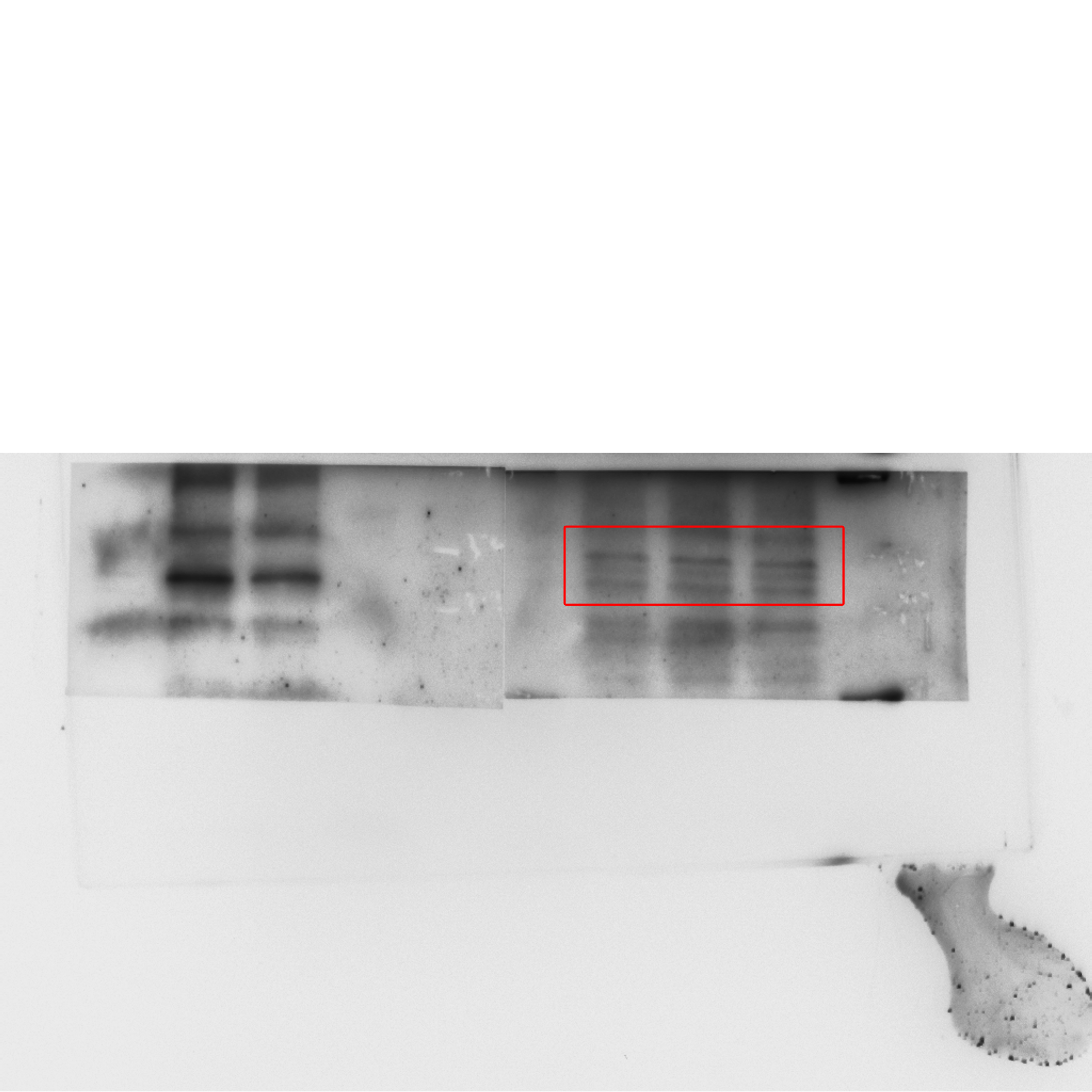

Supplement: Source data 3. [file elife-70151-data3.zip › Source data_v2/Figure 5H/Figure 5H_GSK3b_source data_labelled.jpg]

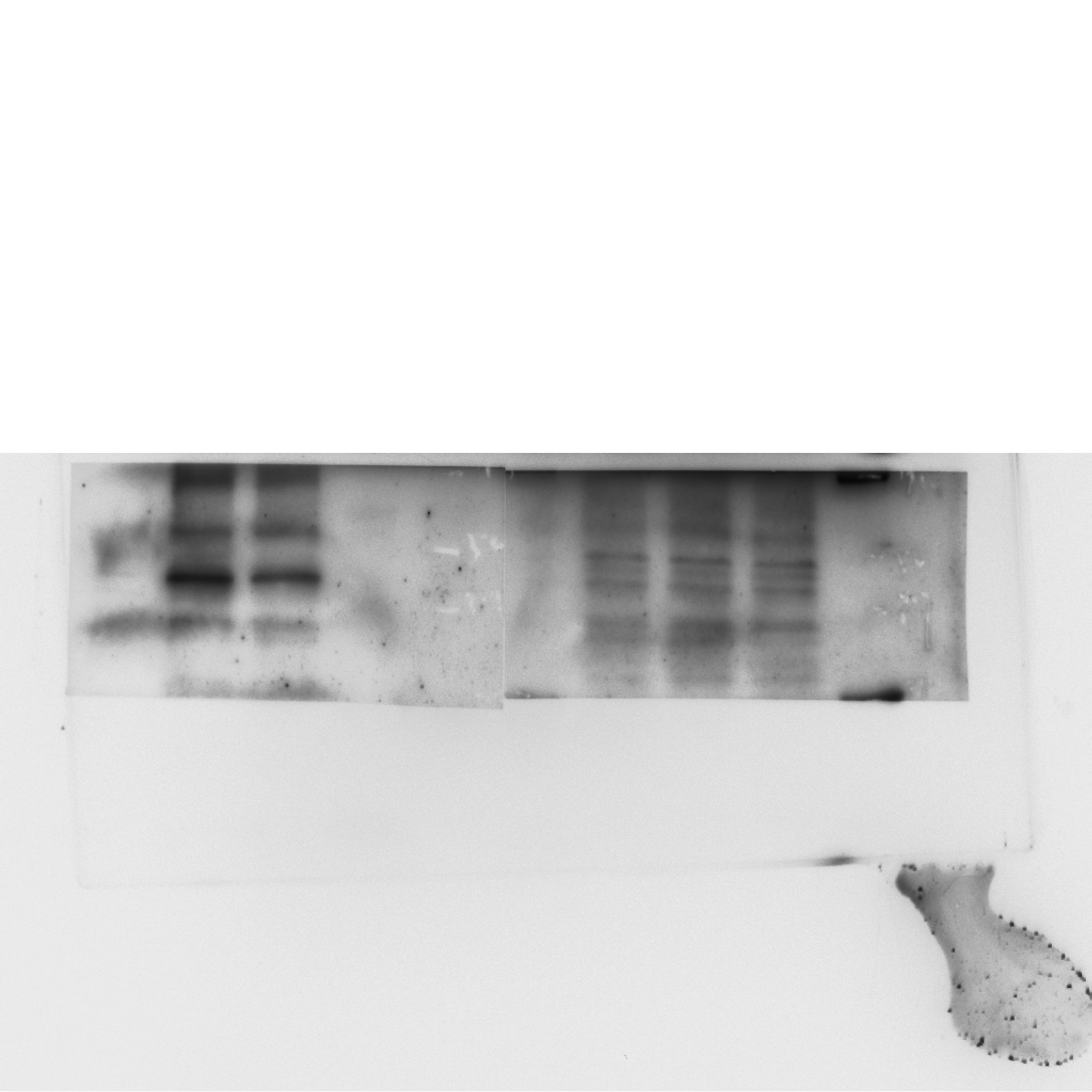

Supplement: Source data 3. [file elife-70151-data3.zip › Source data_v2/Figure 5H/Figure 5H_GSK3b_source data.jpg]

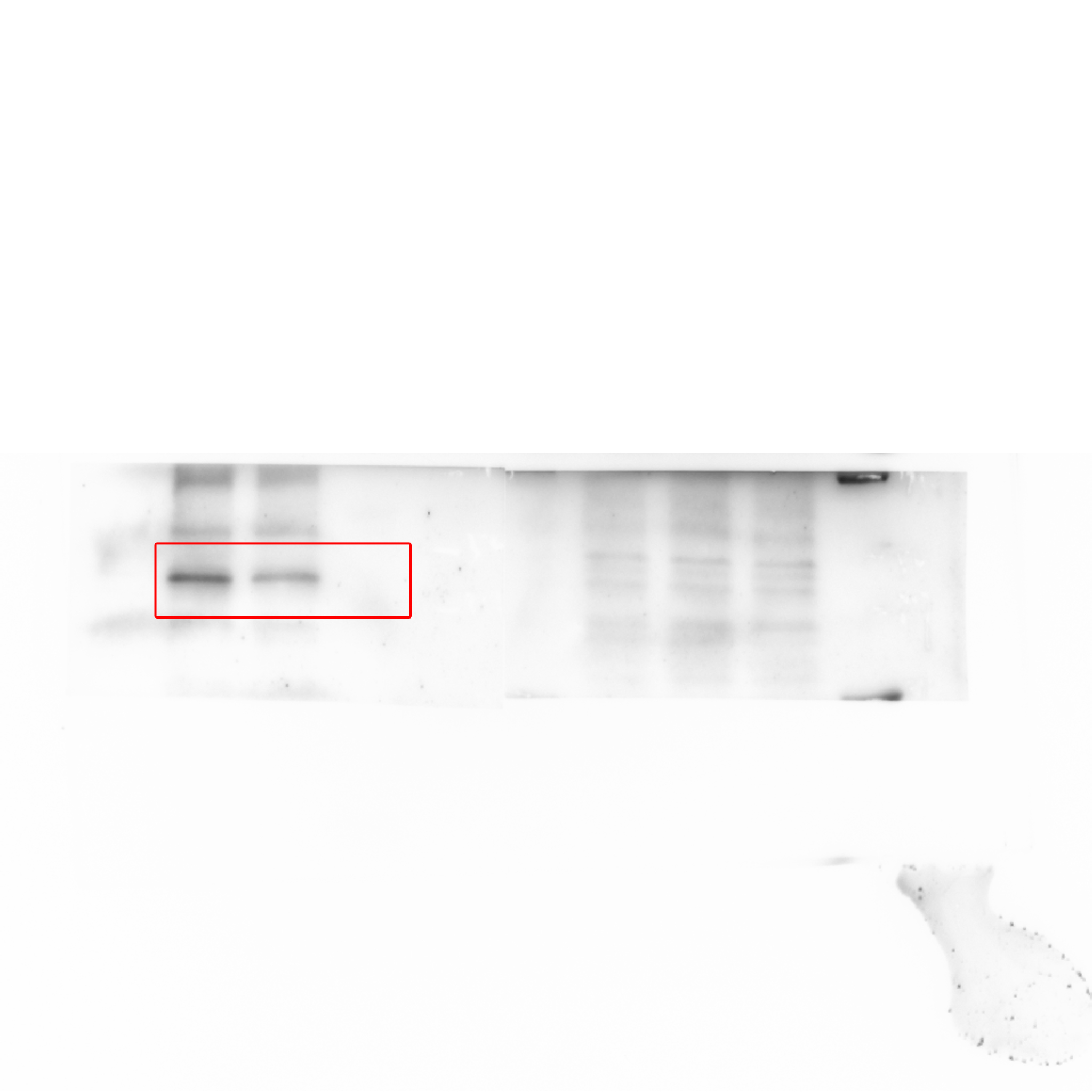

Supplement: Source data 3. [file elife-70151-data3.zip › Source data_v2/Figure 5H/Figure 5H_p-GSK3b_source data_labelled.jpg]

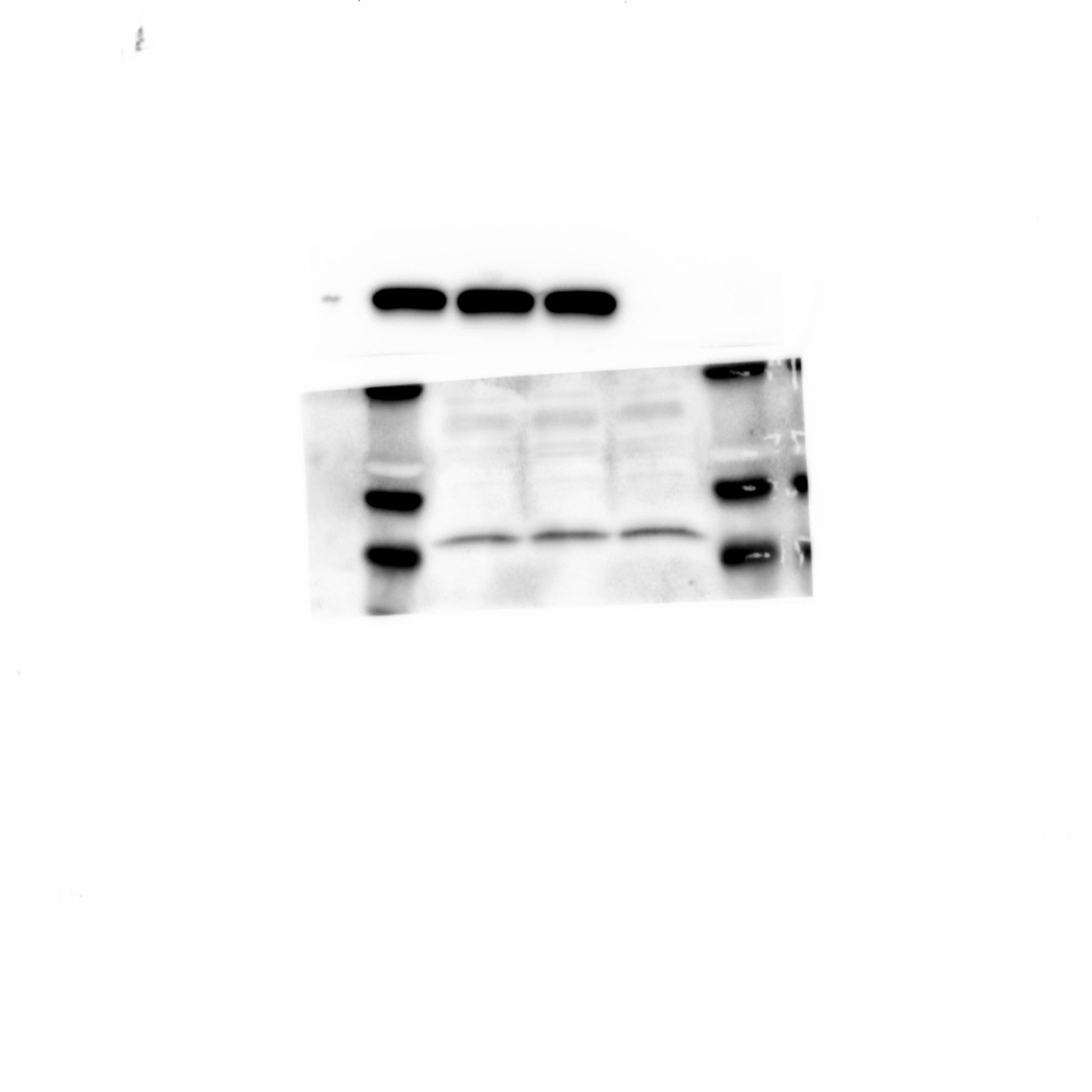

Supplement: Source data 3. [file elife-70151-data3.zip › Source data_v2/Figure 5H/Figure 5H_Histone H3_source data.jpg]

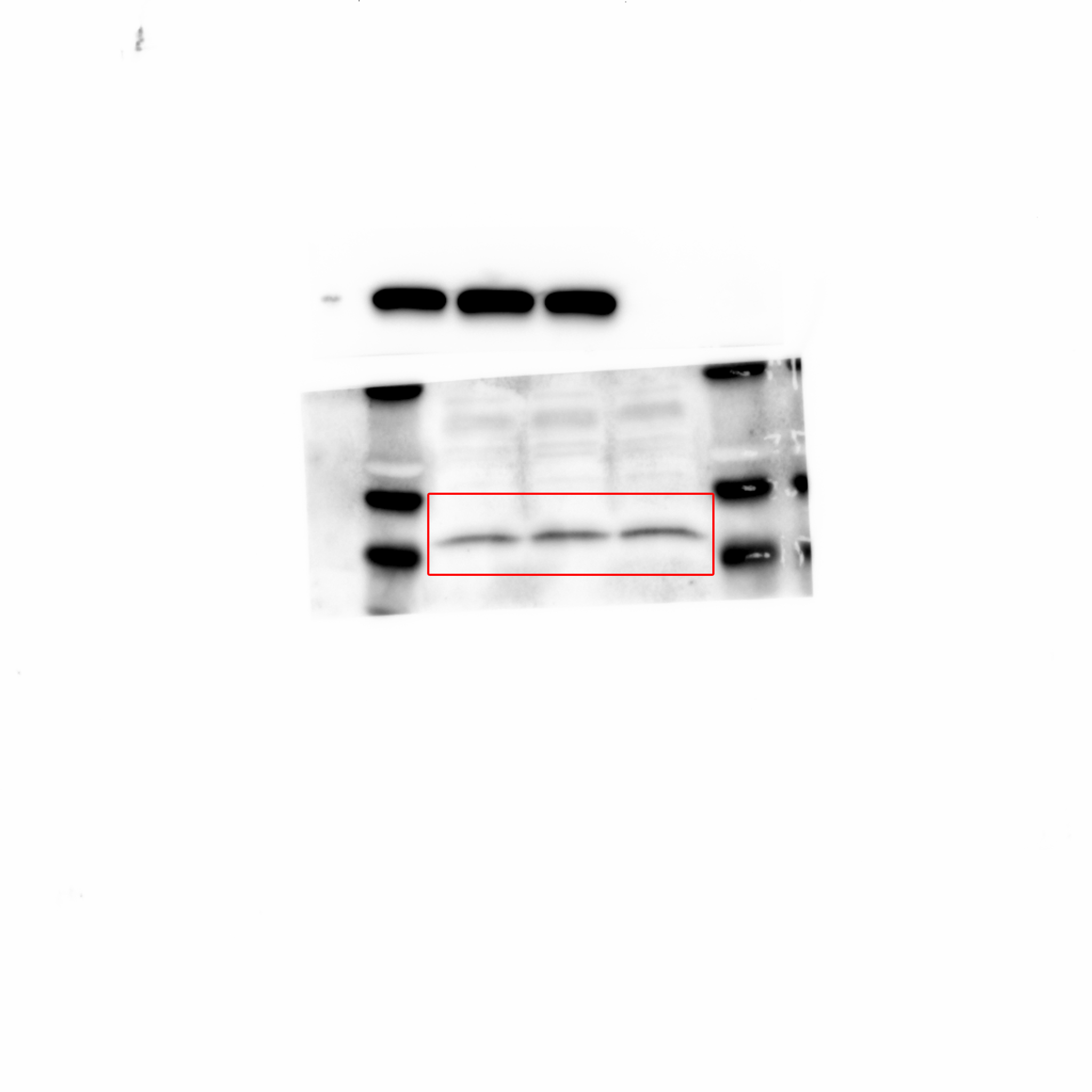

Supplement: Source data 3. [file elife-70151-data3.zip › Source data_v2/Figure 5H/Figure 5H_Histone H3_source data_labelled.jpg]

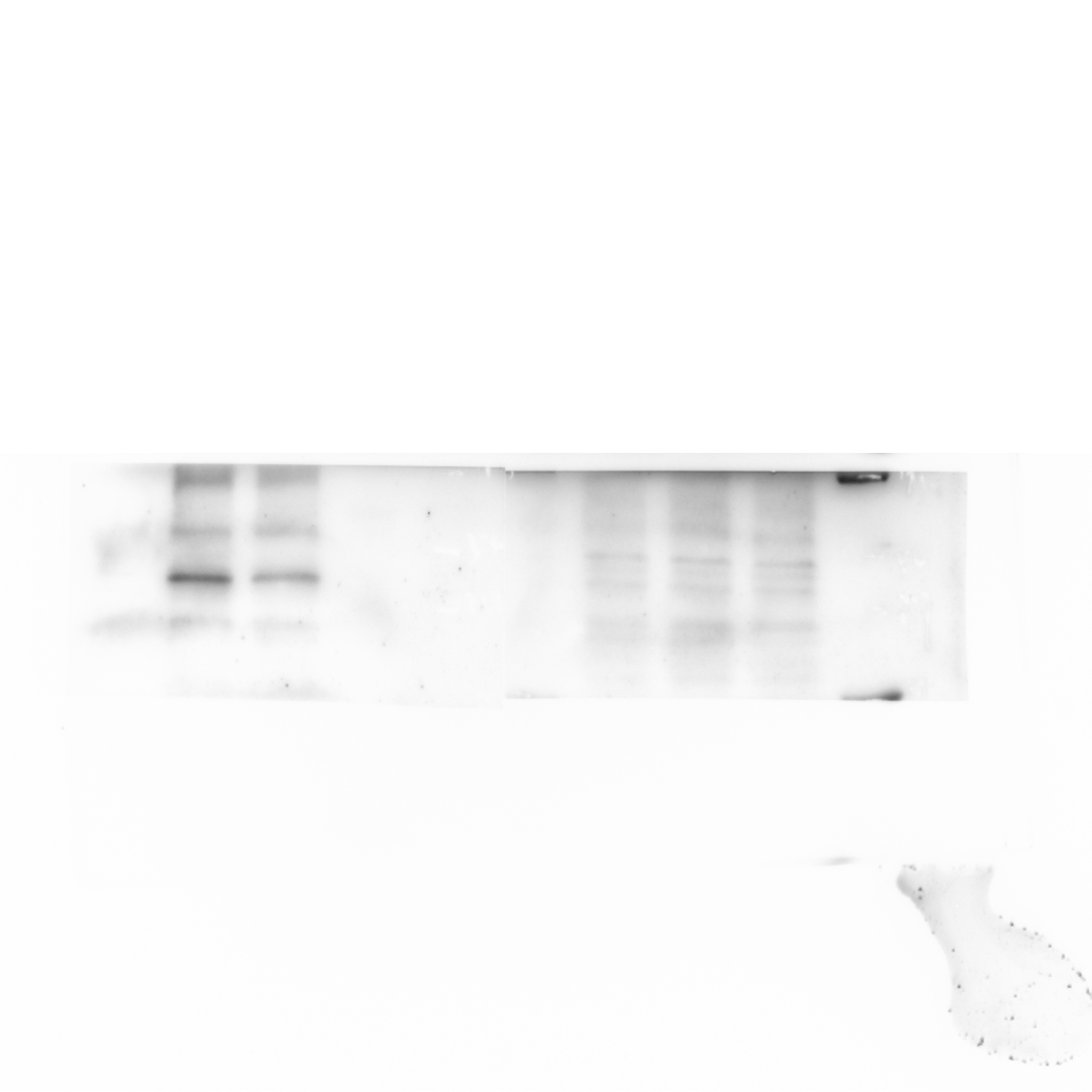

Supplement: Source data 3. [file elife-70151-data3.zip › Source data_v2/Figure 5H/Figure 5H_p-GSK3b_source data.jpg]

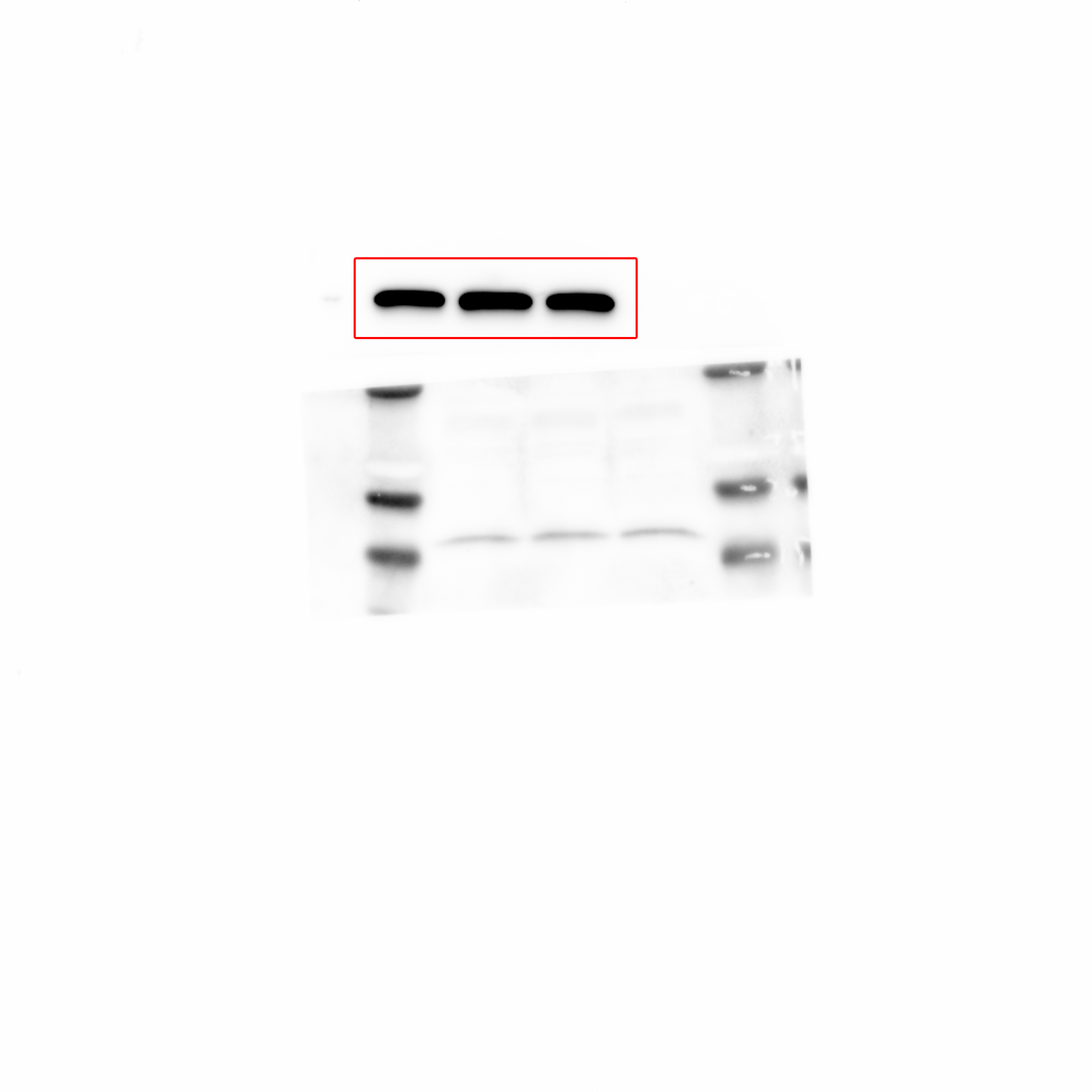

Supplement: Source data 3. [file elife-70151-data3.zip › Source data_v2/Figure 5H/Figure 5H_b-tubulin_source data_labelled.jpg]

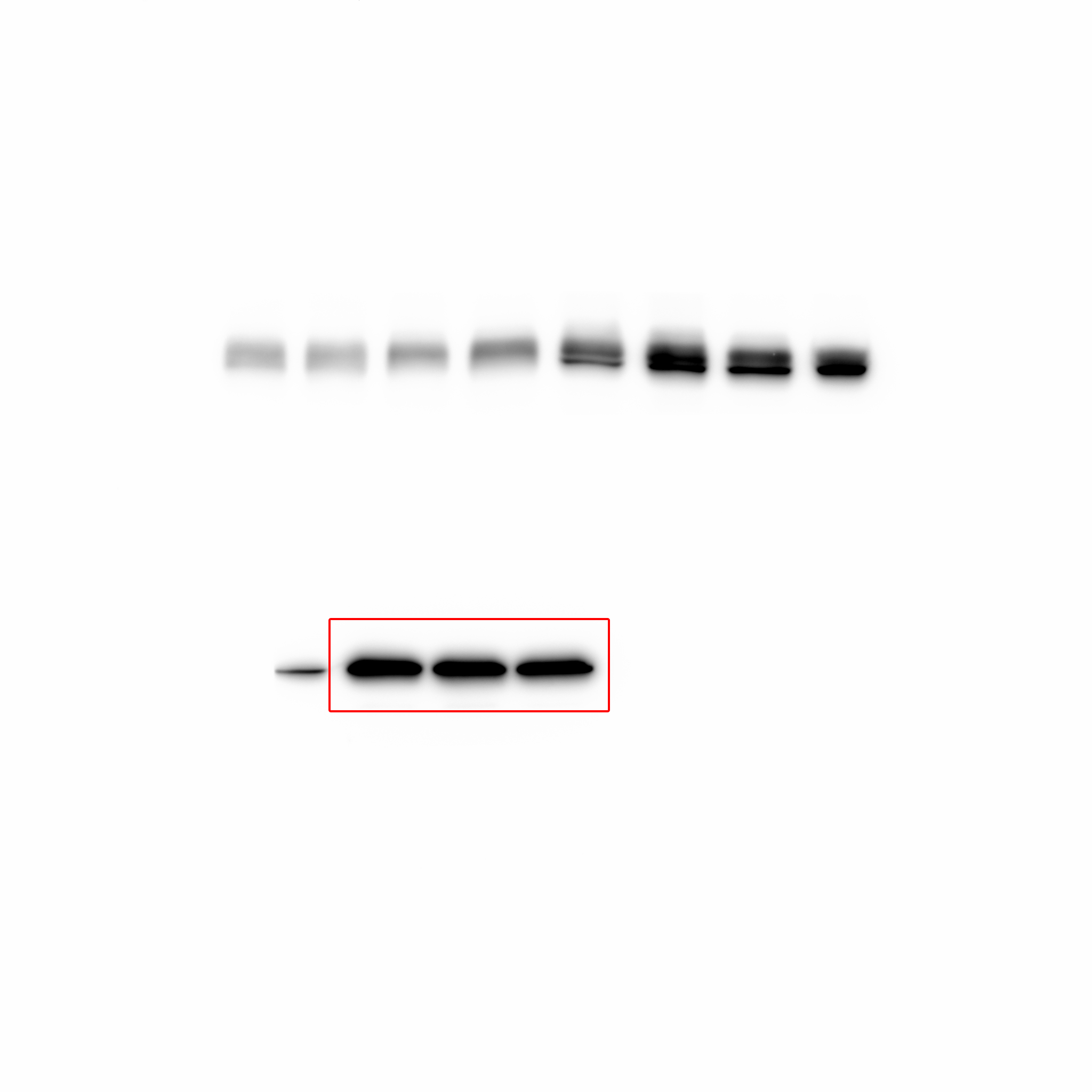

Supplement: Source data 3. [file elife-70151-data3.zip › Source data_v2/Figure 5H/Figure 5H_GAPDH_source data_labelled.jpg]

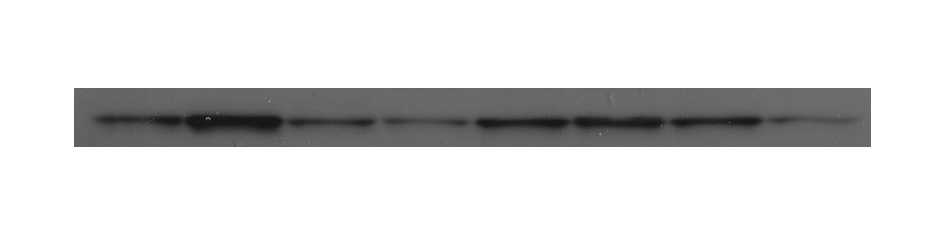

Supplement: Source data 3. [file elife-70151-data3.zip › Source data_v2/Figure 2B/Figure 2B_HTR2C_source data.jpg]

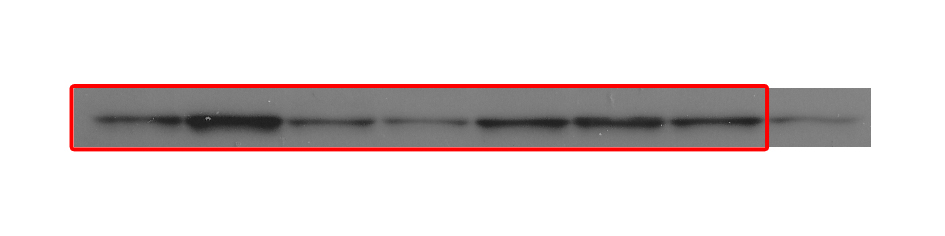

Supplement: Source data 3. [file elife-70151-data3.zip › Source data_v2/Figure 2B/Figure 2B_HTR2C_source data.labelled]

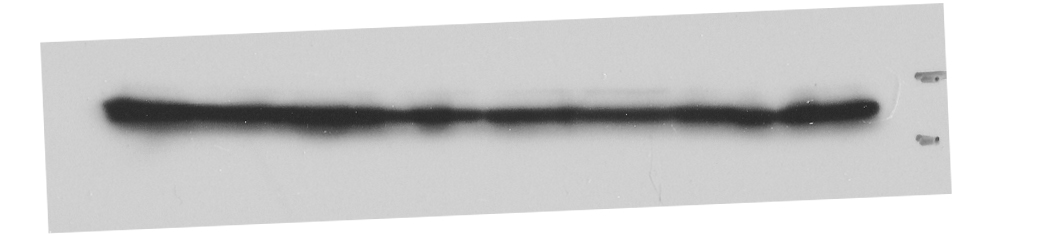

Supplement: Source data 3. [file elife-70151-data3.zip › Source data_v2/Figure 2B/Figure 2B_GAPDH_source data.jpg]

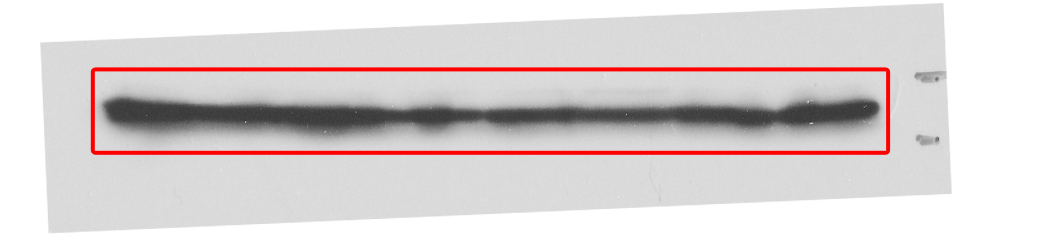

Supplement: Source data 3. [file elife-70151-data3.zip › Source data_v2/Figure 2B/Figure 2B_GAPDH_source data_labelled.jpg]

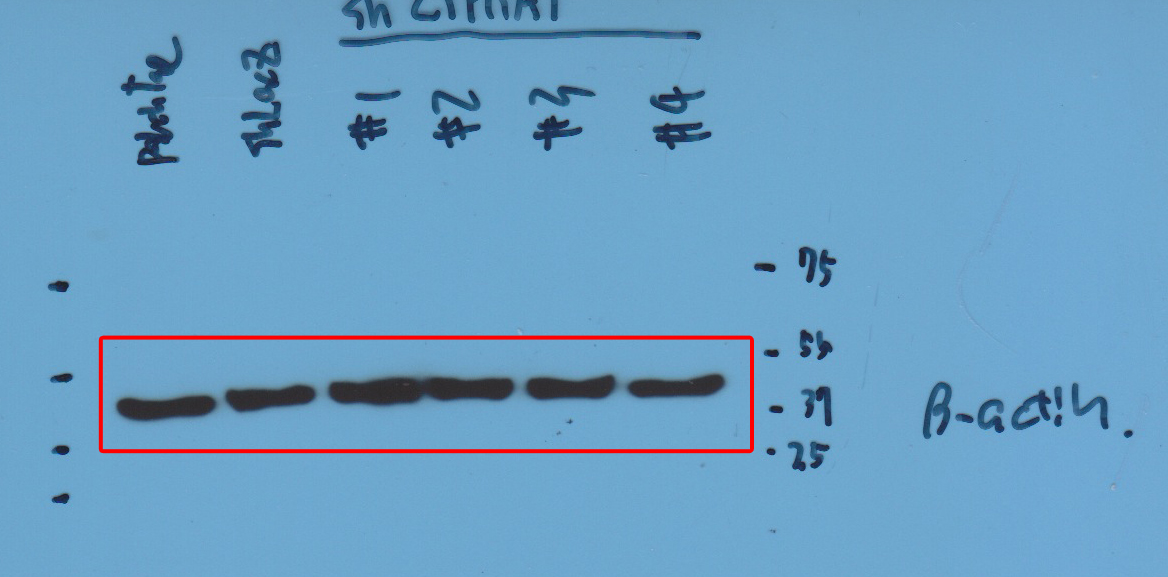

Supplement: Source data 3. [file elife-70151-data3.zip › Source data_v2/Figure 1-figure supplement 2/Figure S1b_sorce data_b-actin_lower_labelled.jpg]

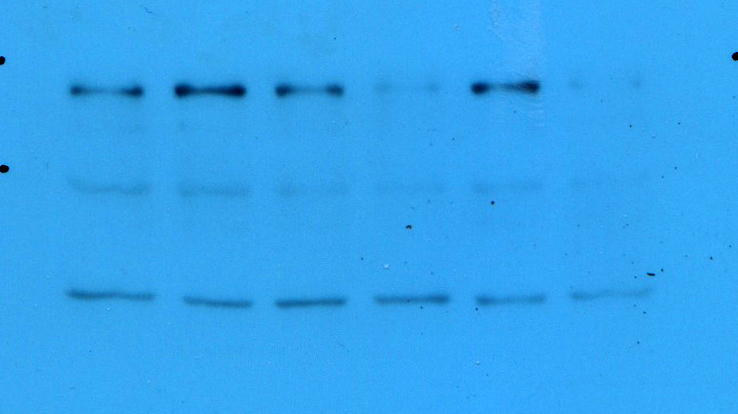

Supplement: Source data 3. [file elife-70151-data3.zip › Source data_v2/Figure 1-figure supplement 2/Figure S1b_sorce data_CYP11A1.jpg]

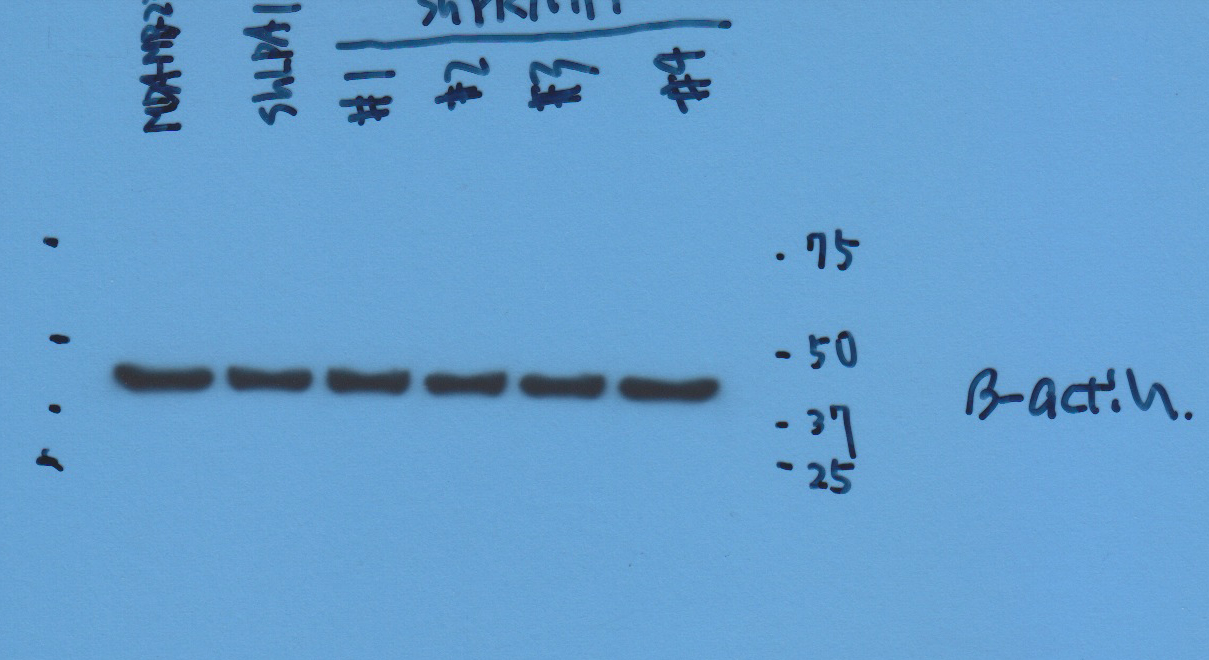

Supplement: Source data 3. [file elife-70151-data3.zip › Source data_v2/Figure 1-figure supplement 2/Figure S1b_sorce data_b-actin_upper.jpg]

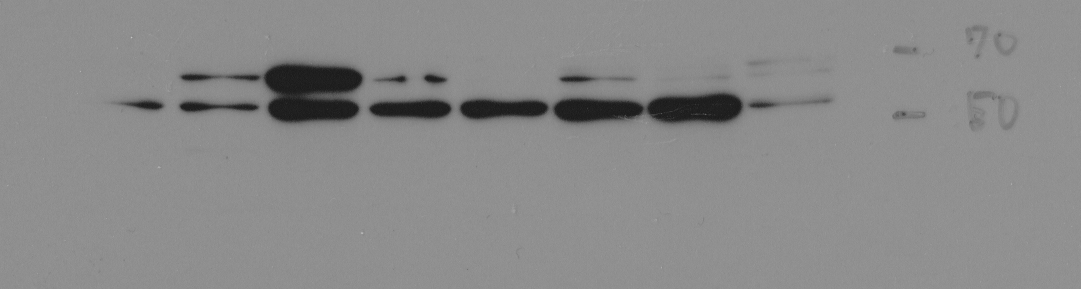

Supplement: Source data 3. [file elife-70151-data3.zip › Source data_v2/Figure 1-figure supplement 2/Figure S1a_Source data_CYP11A1.jpg]

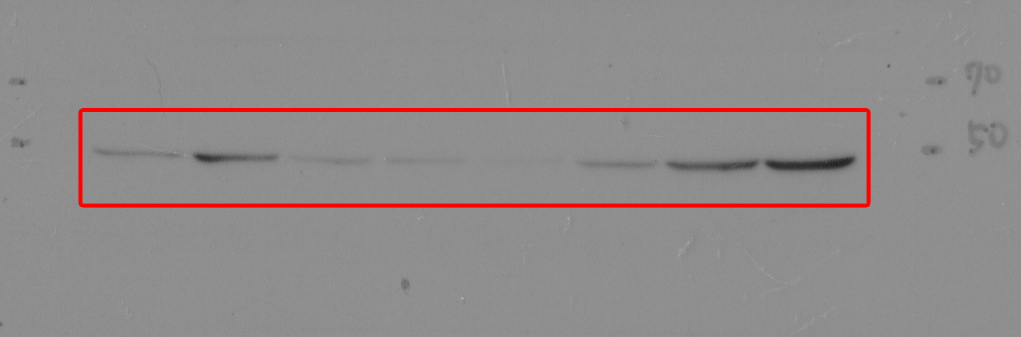

Supplement: Source data 3. [file elife-70151-data3.zip › Source data_v2/Figure 1-figure supplement 2/Figure S1a_Source data_PRMT1_labelled.jpg]

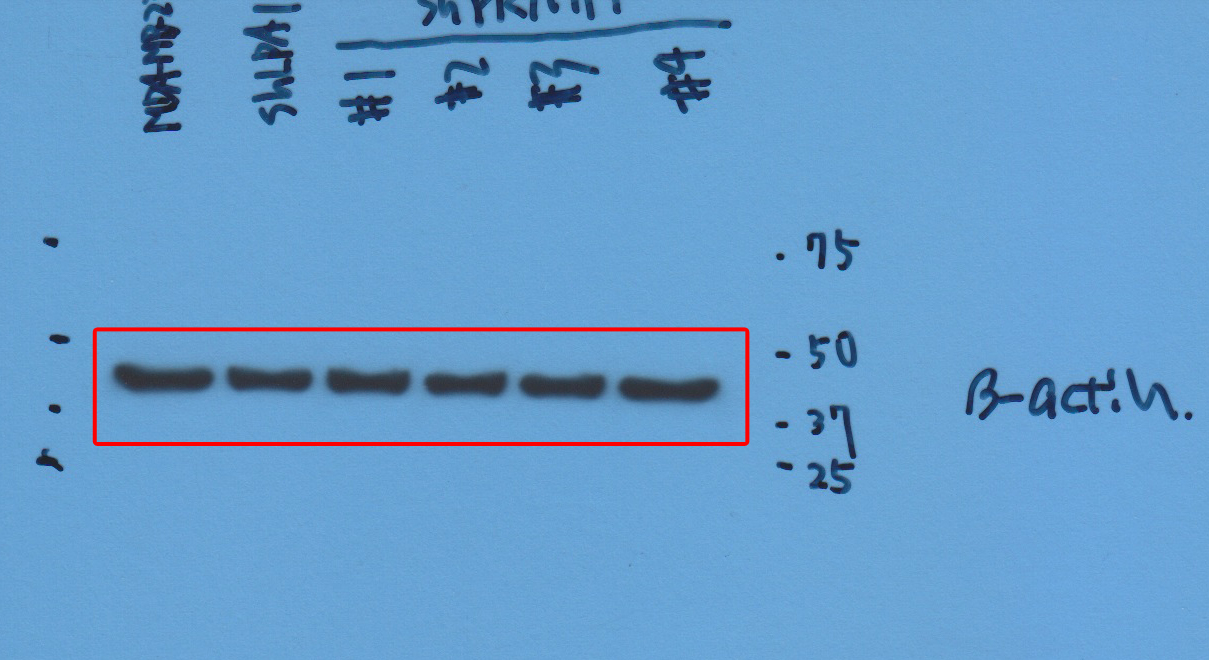

Supplement: Source data 3. [file elife-70151-data3.zip › Source data_v2/Figure 1-figure supplement 2/Figure S1b_sorce data_b-actin_upper_labelled.jpg]

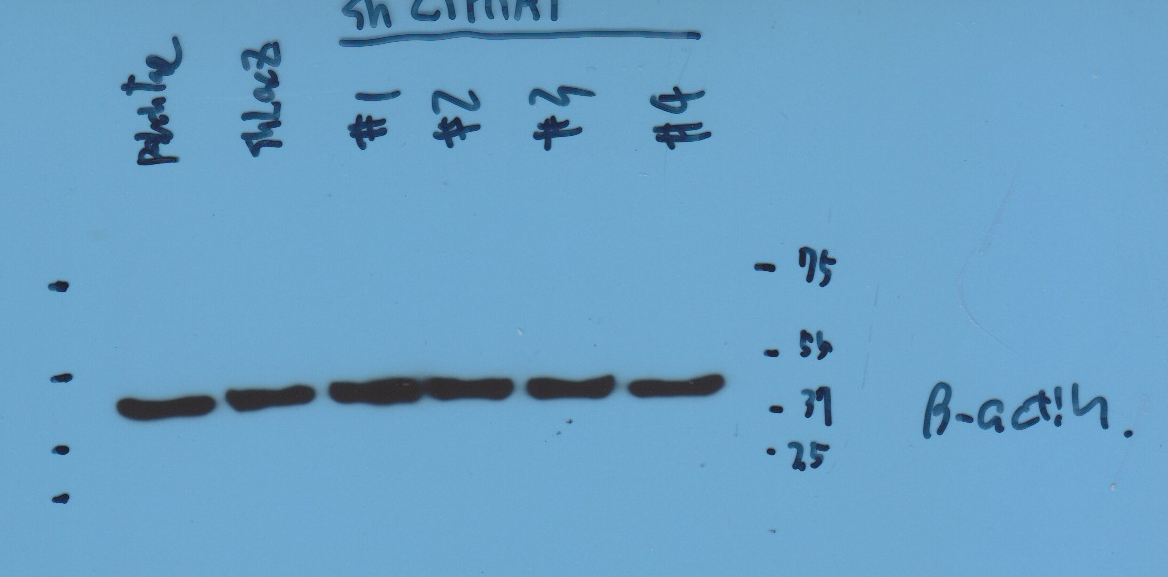

Supplement: Source data 3. [file elife-70151-data3.zip › Source data_v2/Figure 1-figure supplement 2/Figure S1b_sorce data_b-actin_lower.jpg]

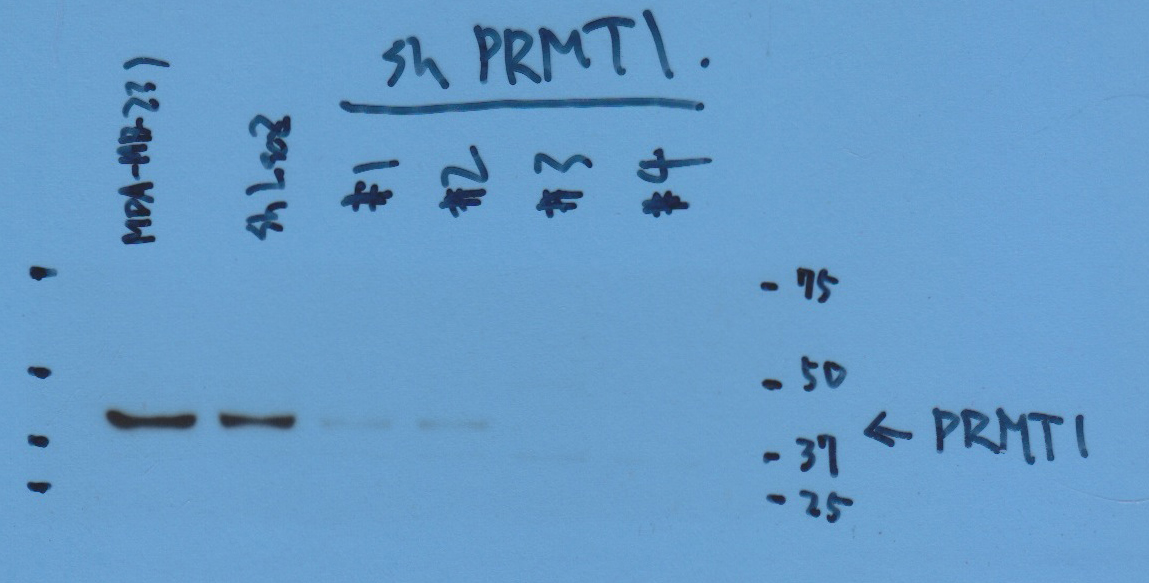

Supplement: Source data 3. [file elife-70151-data3.zip › Source data_v2/Figure 1-figure supplement 2/Figure S1b_sorce data_PRMT1.jpg]

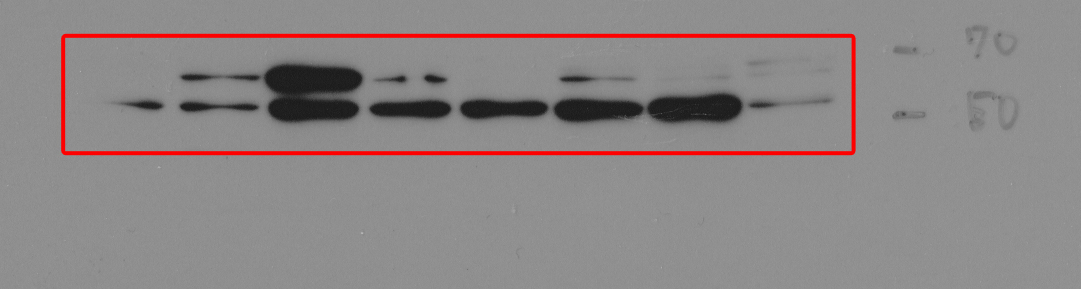

Supplement: Source data 3. [file elife-70151-data3.zip › Source data_v2/Figure 1-figure supplement 2/Figure S1a_CYP11A1_source data_labbeled]

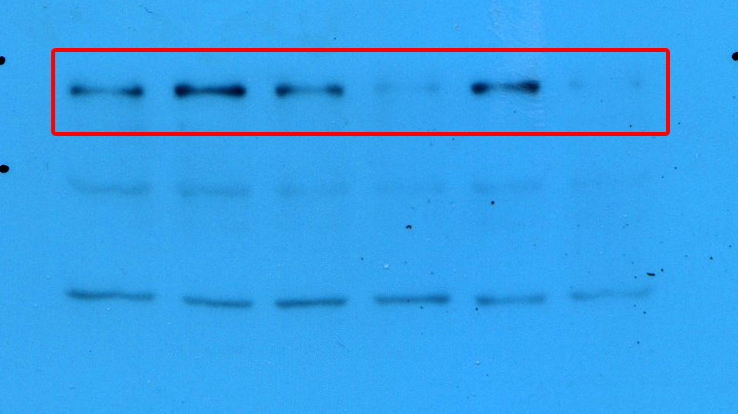

Supplement: Source data 3. [file elife-70151-data3.zip › Source data_v2/Figure 1-figure supplement 2/Figure S1b_sorce data_CYP11A1_labelled.jpg]

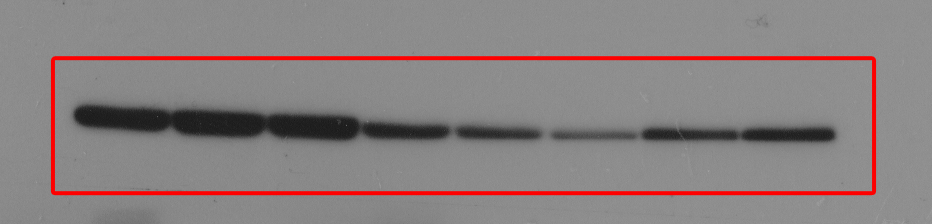

Supplement: Source data 3. [file elife-70151-data3.zip › Source data_v2/Figure 1-figure supplement 2/Figure.S1a_b-actin1_source data_labelled.jpg]

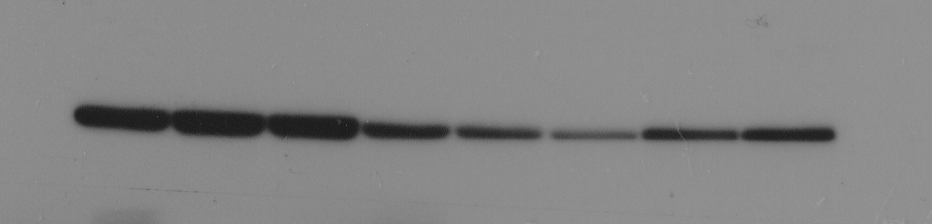

Supplement: Source data 3. [file elife-70151-data3.zip › Source data_v2/Figure 1-figure supplement 2/Figure.S1a_b-actin1_source data.jpg]

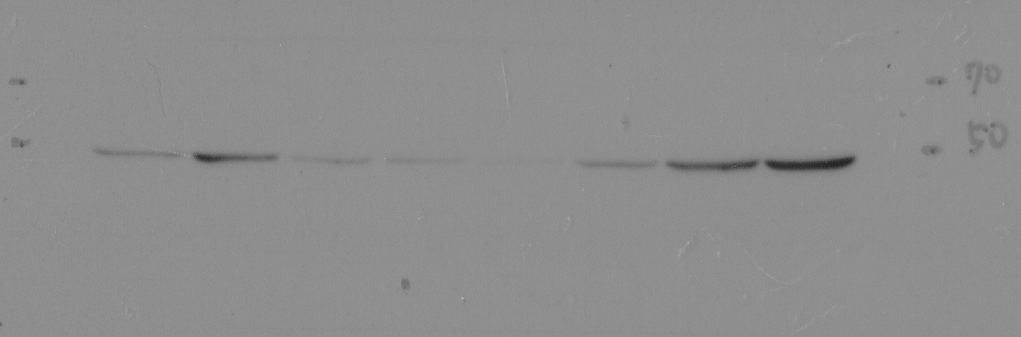

Supplement: Source data 3. [file elife-70151-data3.zip › Source data_v2/Figure 1-figure supplement 2/Figure S1a_Source data_PRMT1.jpg]

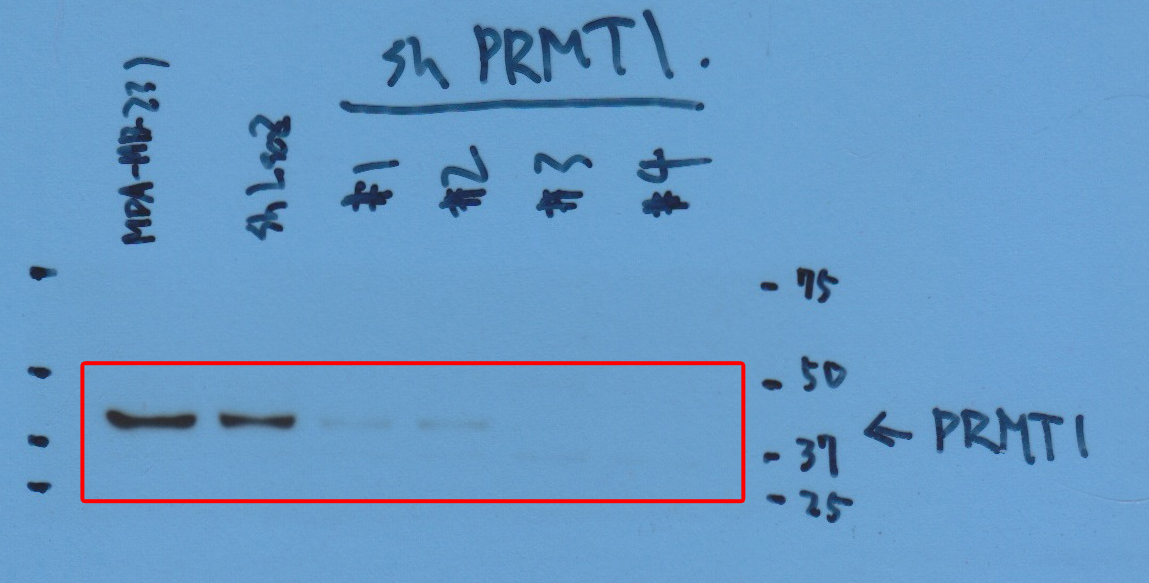

Supplement: Source data 3. [file elife-70151-data3.zip › Source data_v2/Figure 1-figure supplement 2/Figure S1b_sorce data_PRMT1_labelled.jpg]

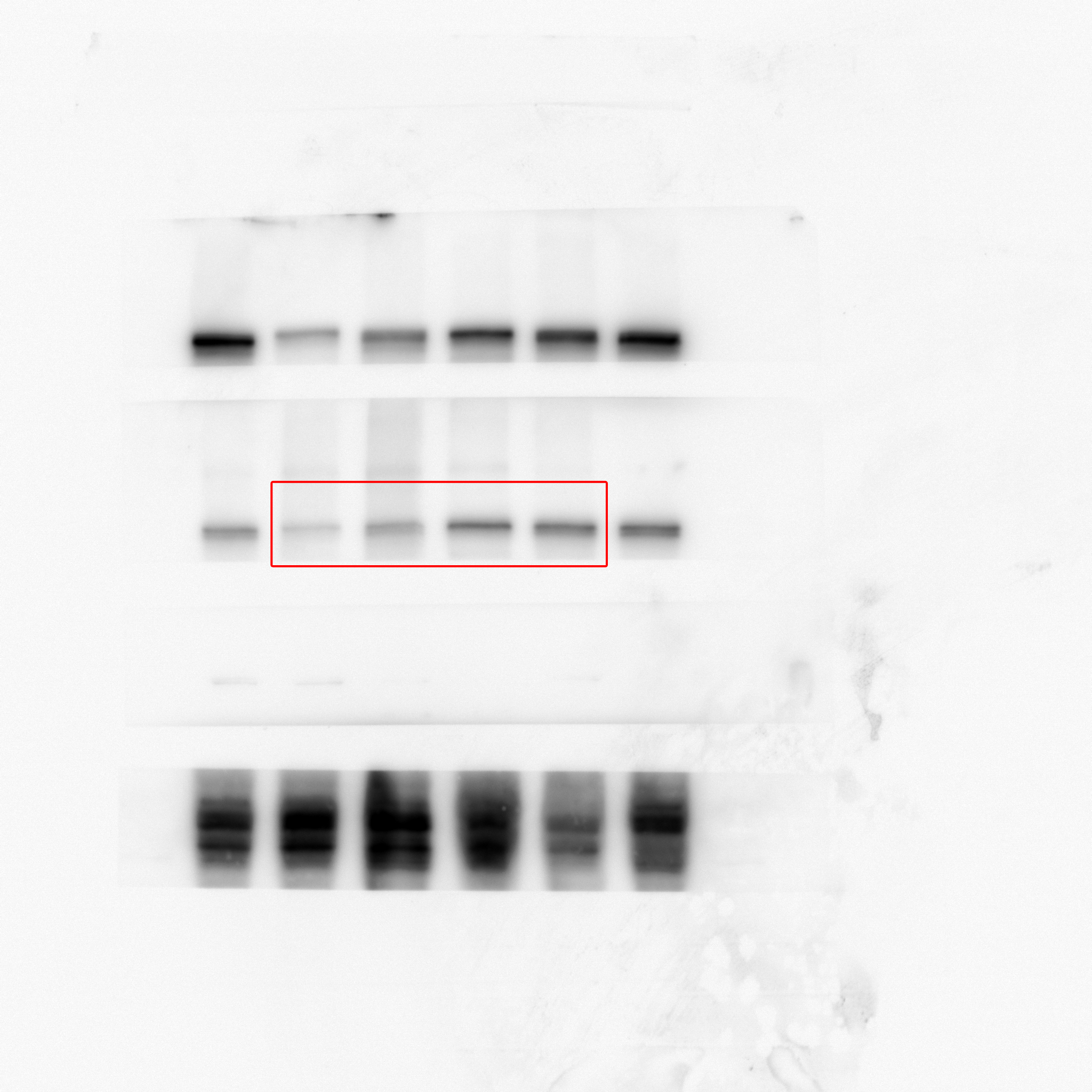

Supplement: Source data 3. [file elife-70151-data3.zip › Source data_v2/Figure 5C/Figure 5C_b-catenin_cytoplasm_source data_labelled.jpg]

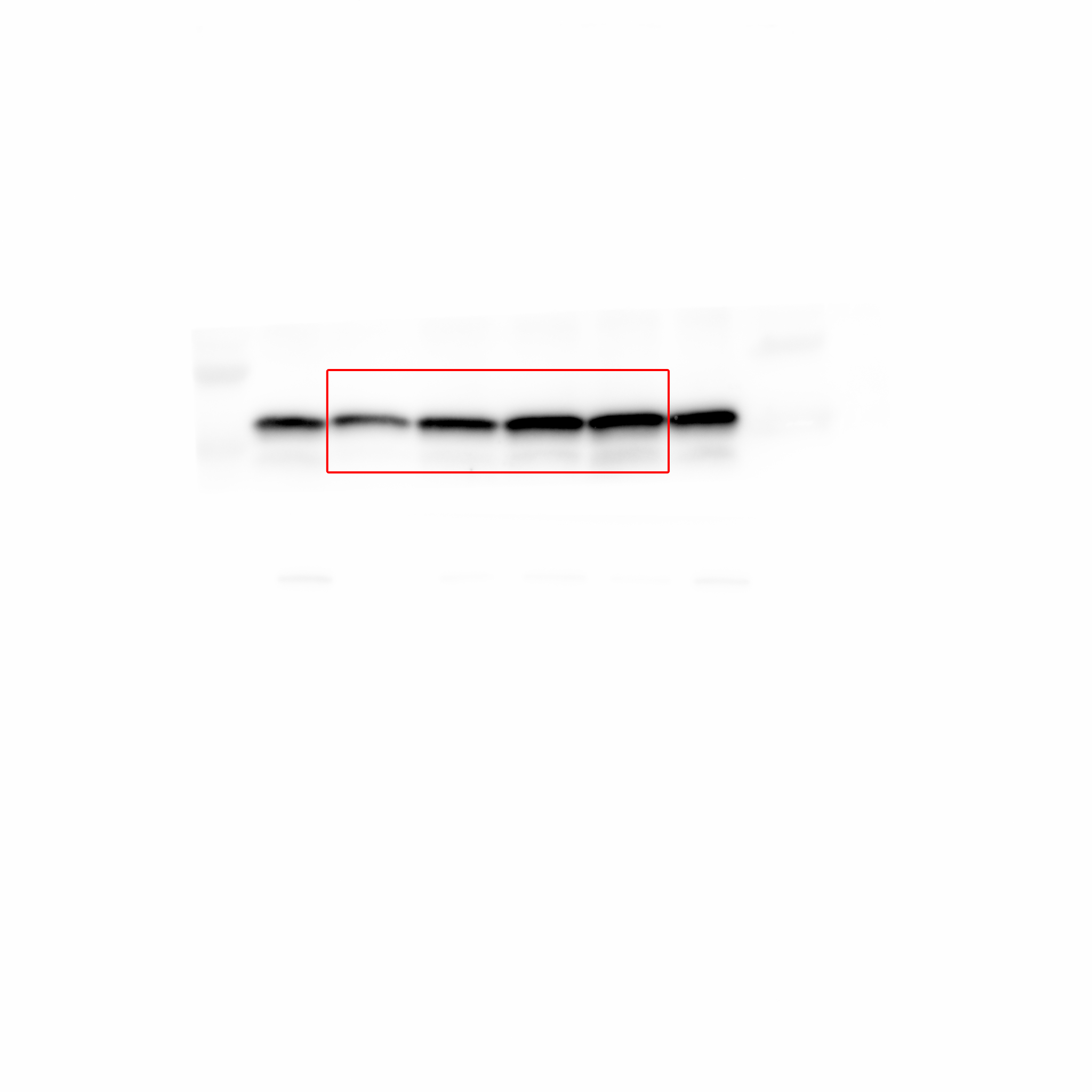

Supplement: Source data 3. [file elife-70151-data3.zip › Source data_v2/Figure 5C/Figure 5C_Histone H3_source data_labelled.jpg]

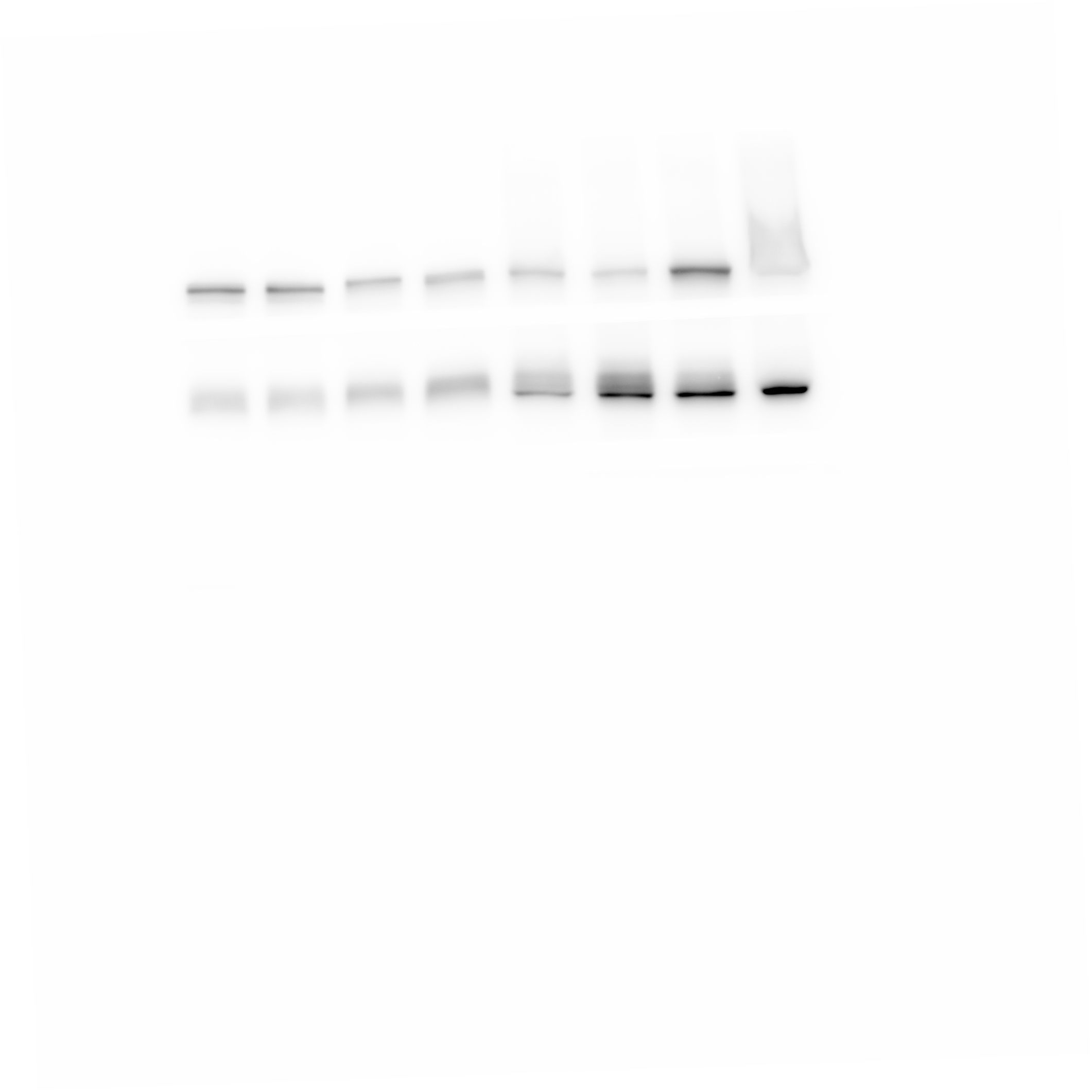

Supplement: Source data 3. [file elife-70151-data3.zip › Source data_v2/Figure 5C/Figure 5C_GSKb_source data.jpg]

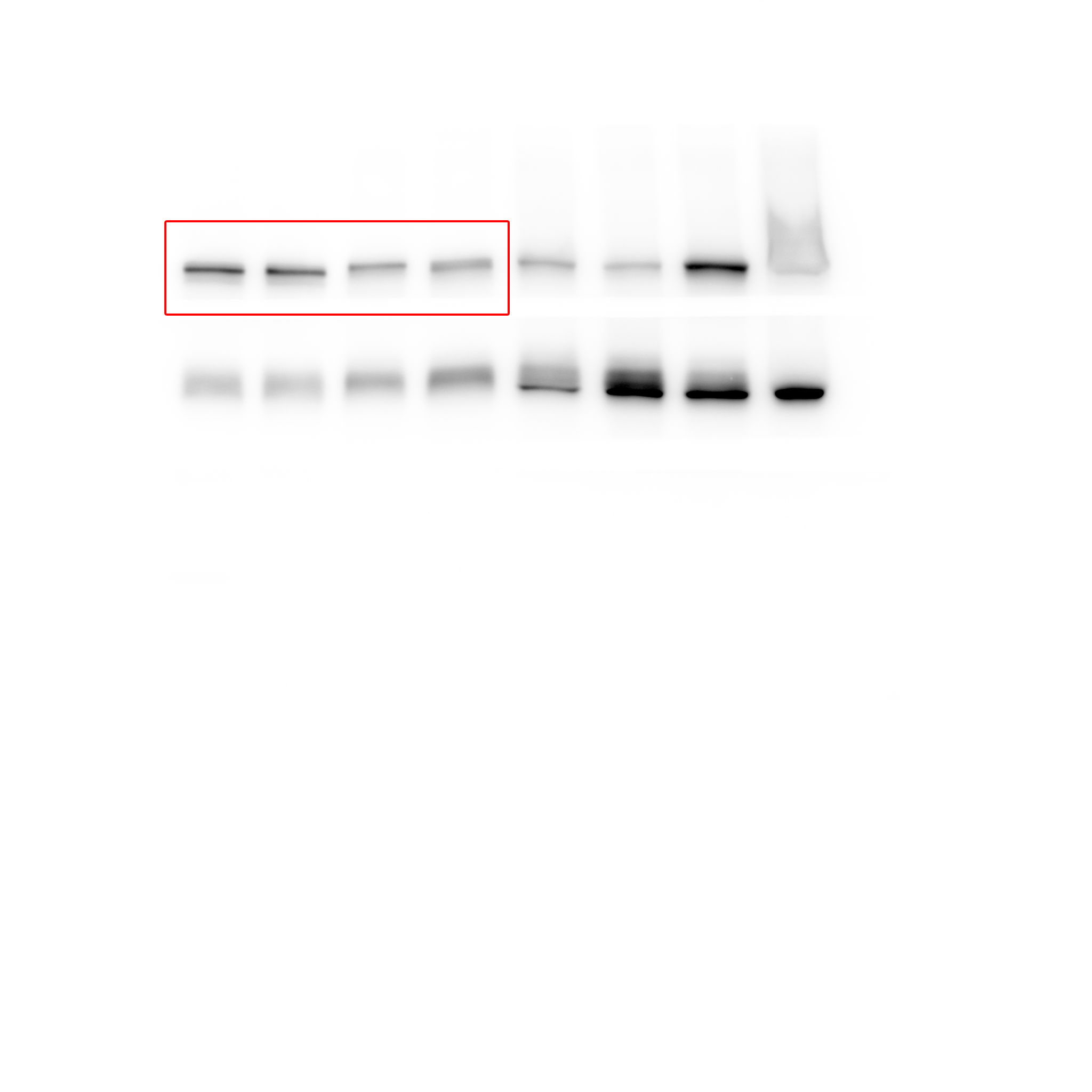

Supplement: Source data 3. [file elife-70151-data3.zip › Source data_v2/Figure 5C/Figure 5C_b-catenin_source data_labelled.jpg]

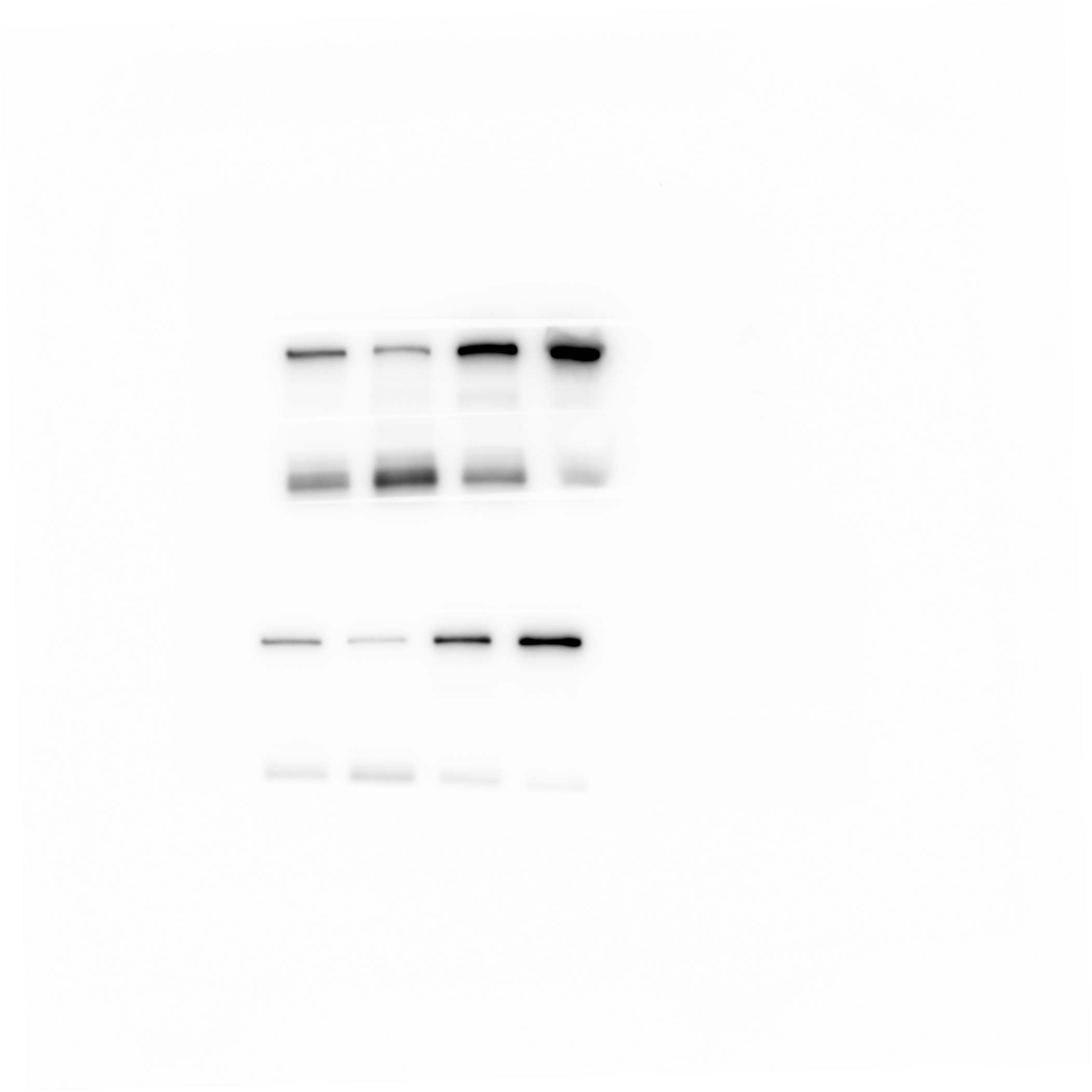

Supplement: Source data 3. [file elife-70151-data3.zip › Source data_v2/Figure 5C/Figure 5C_E-cadherin_source data.jpg]

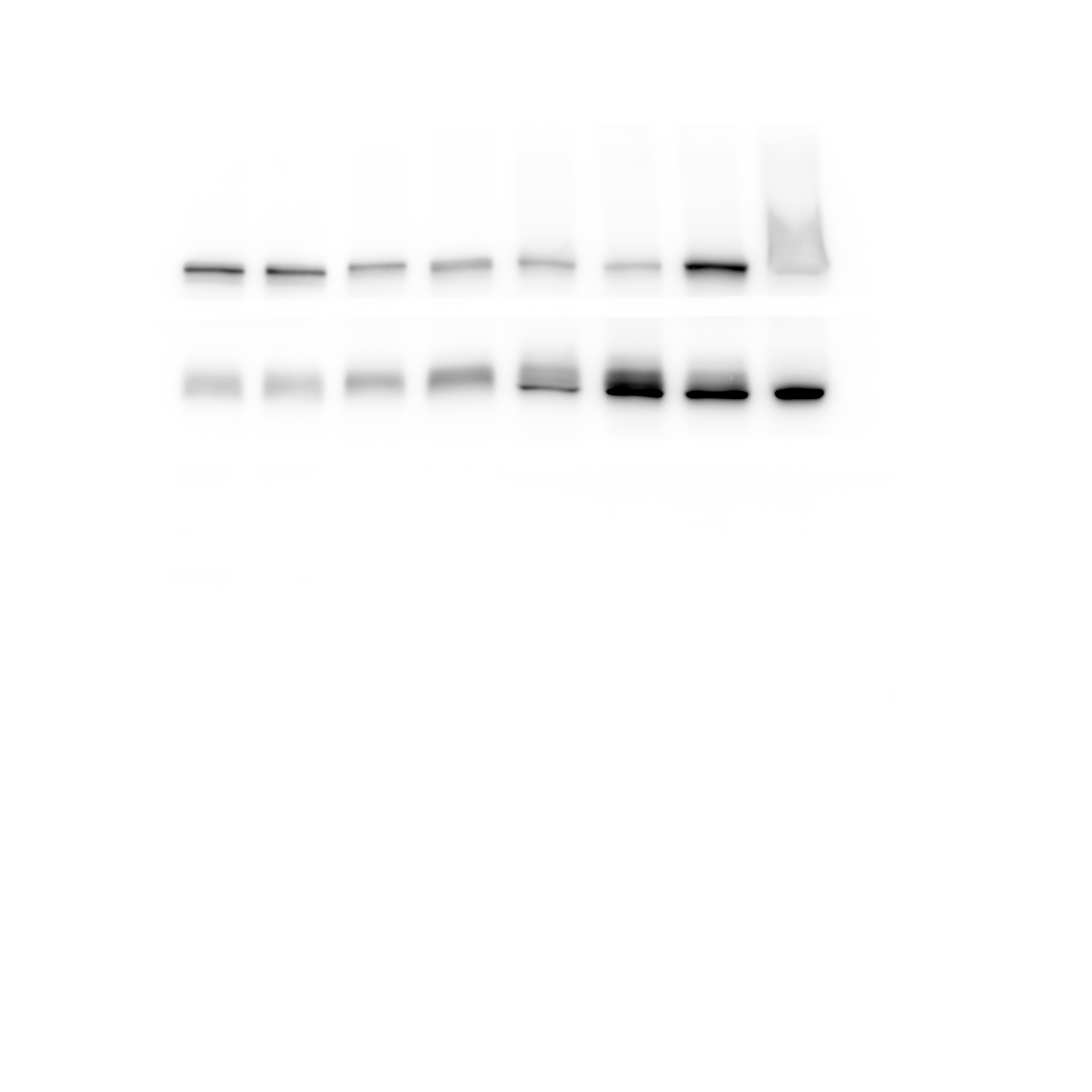

Supplement: Source data 3. [file elife-70151-data3.zip › Source data_v2/Figure 5C/Figure 5C_b-catenin_source data.jpg]

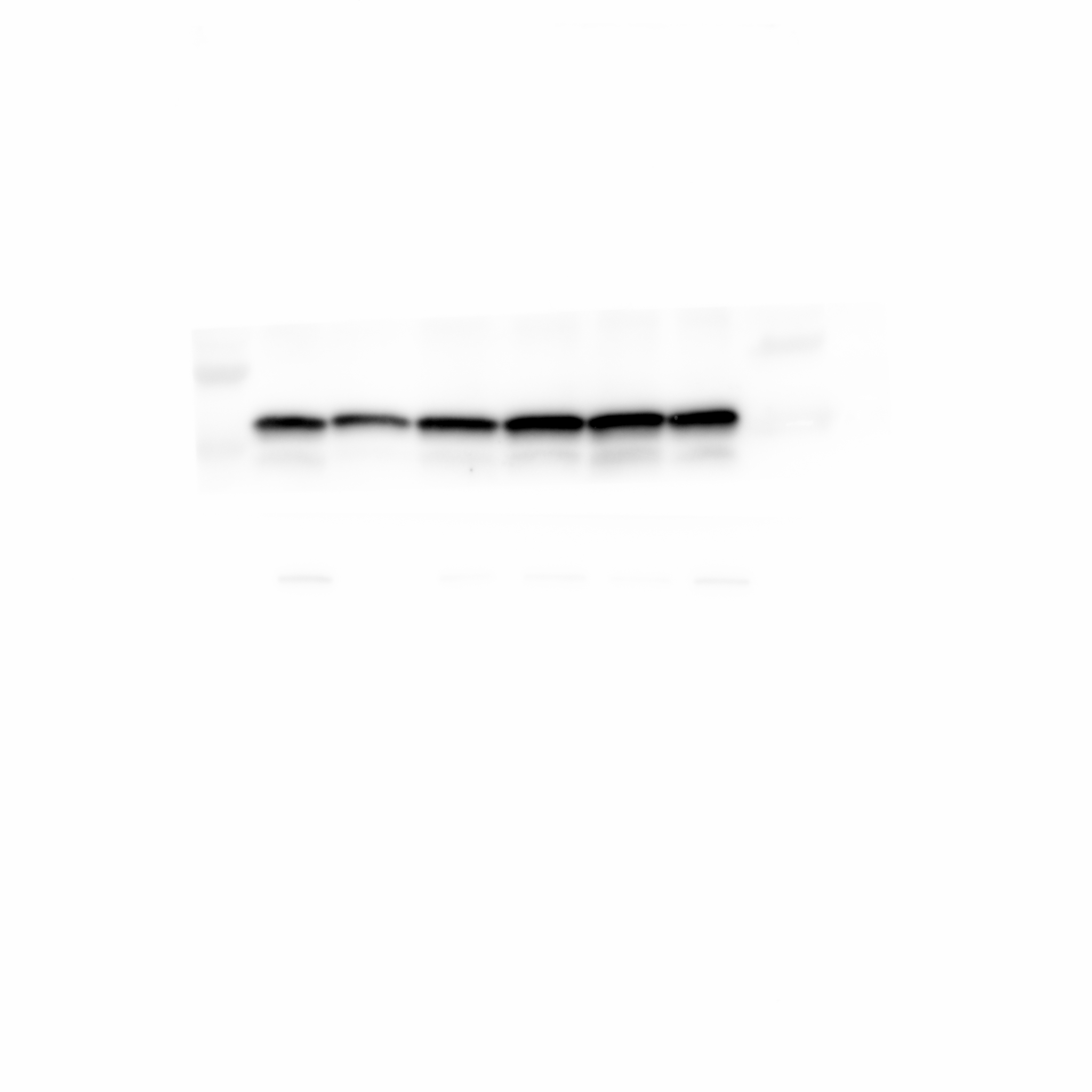

Supplement: Source data 3. [file elife-70151-data3.zip › Source data_v2/Figure 5C/Figure 5C_Histone H3_source data.jpg]

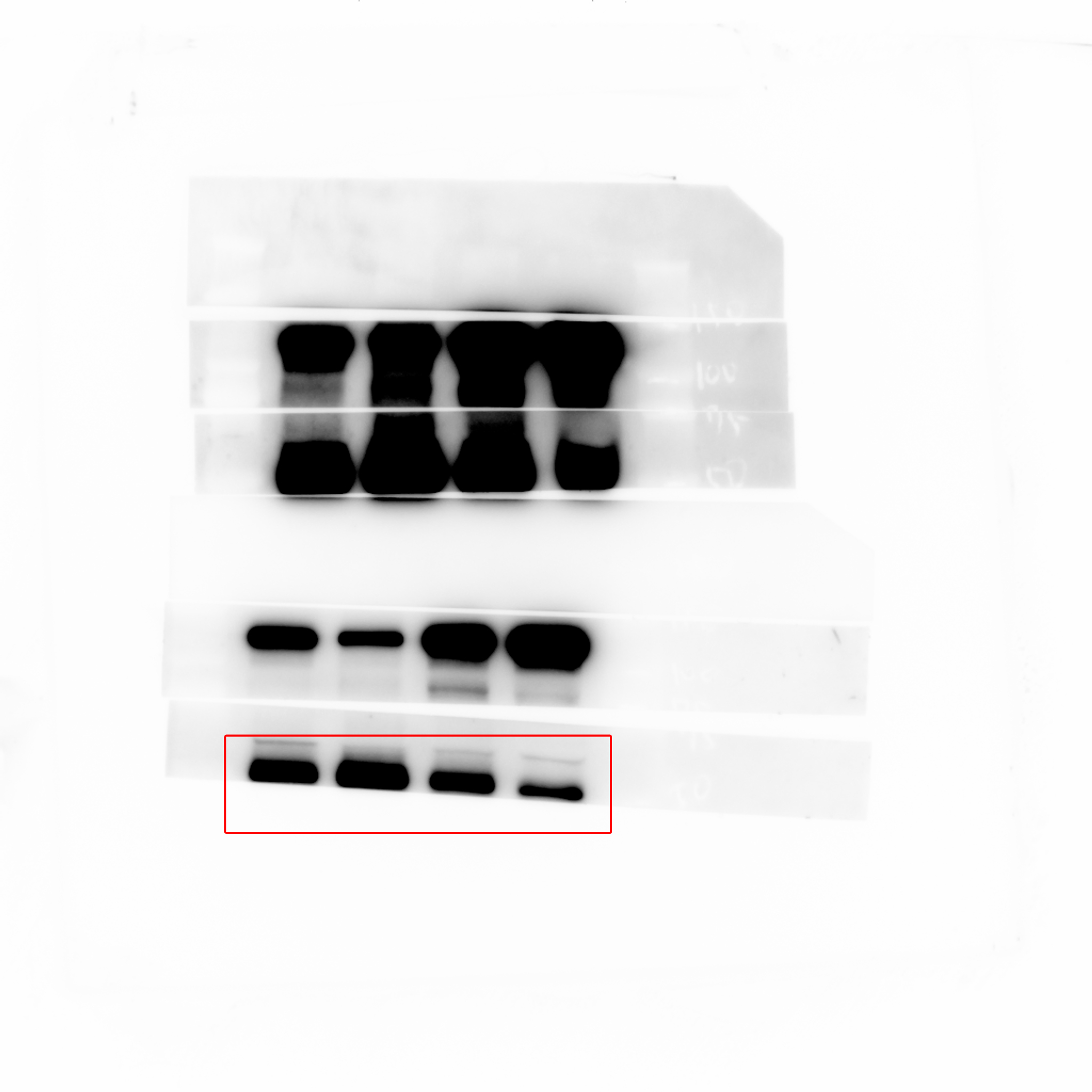

Supplement: Source data 3. [file elife-70151-data3.zip › Source data_v2/Figure 5C/Figure 5C_Luciferase_source data.labelled]

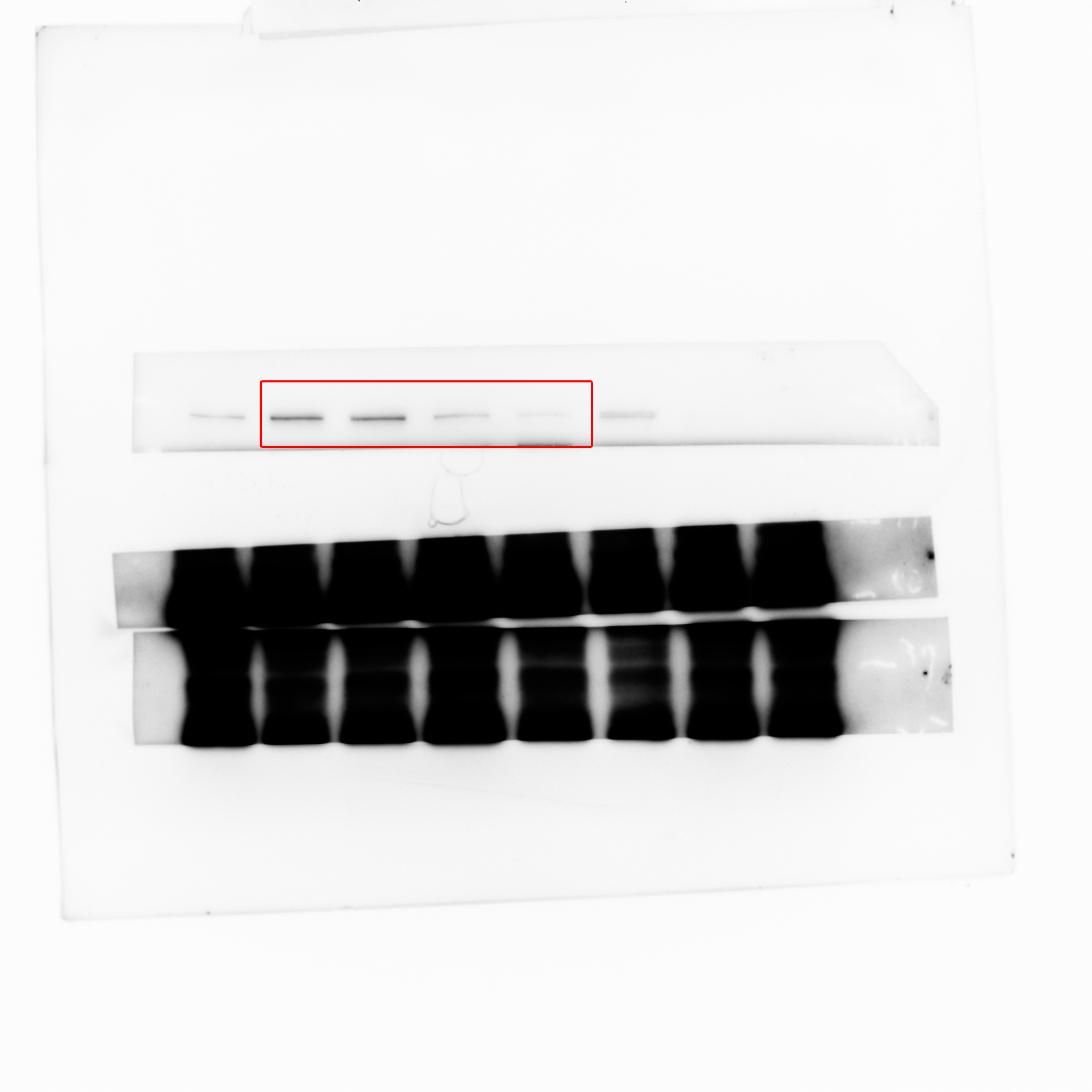

Supplement: Source data 3. [file elife-70151-data3.zip › Source data_v2/Figure 5C/Figure 5C_Zeb1_source data_labelled.jpg]

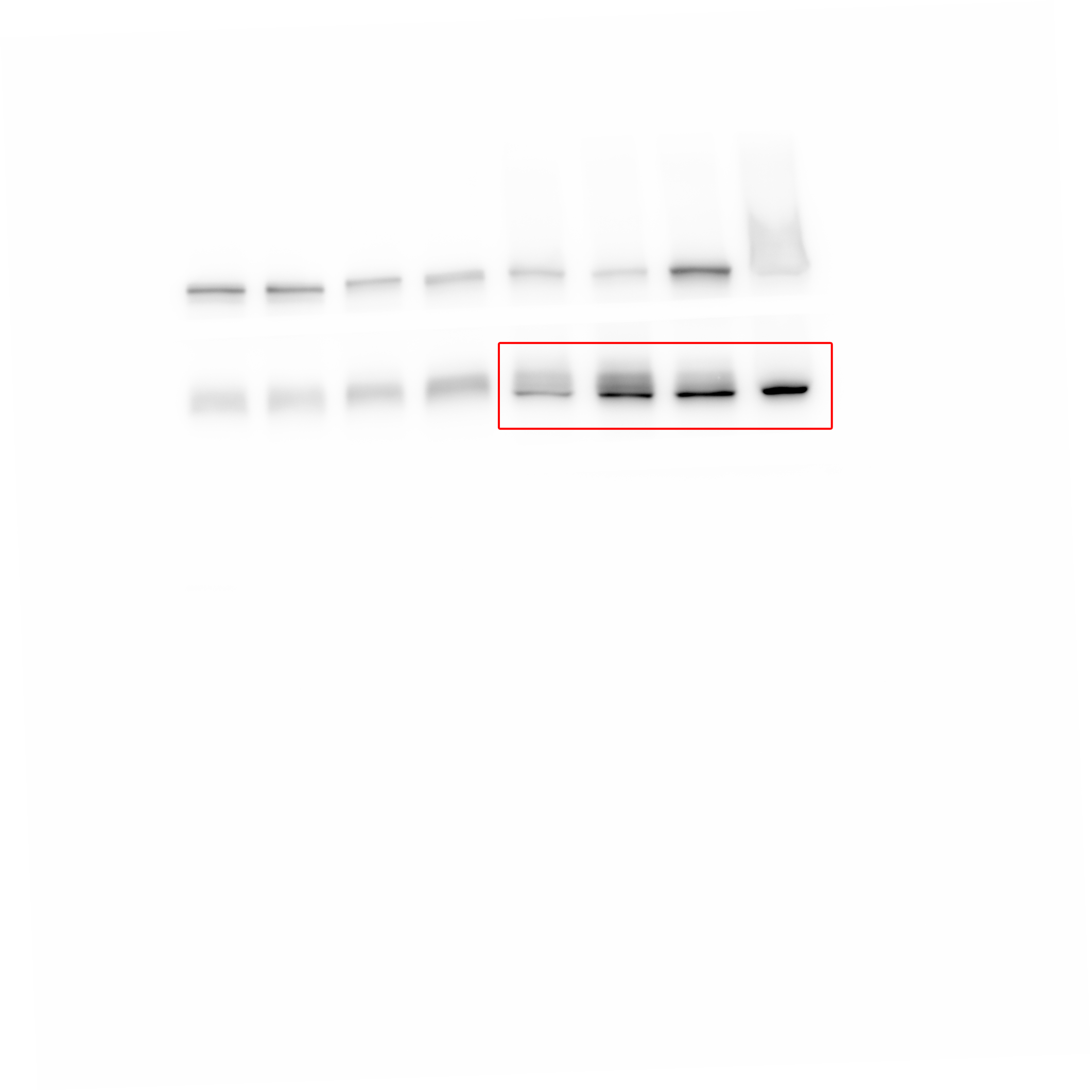

Supplement: Source data 3. [file elife-70151-data3.zip › Source data_v2/Figure 5C/Figure 5C_GSKb_source data_labelled.jpg]

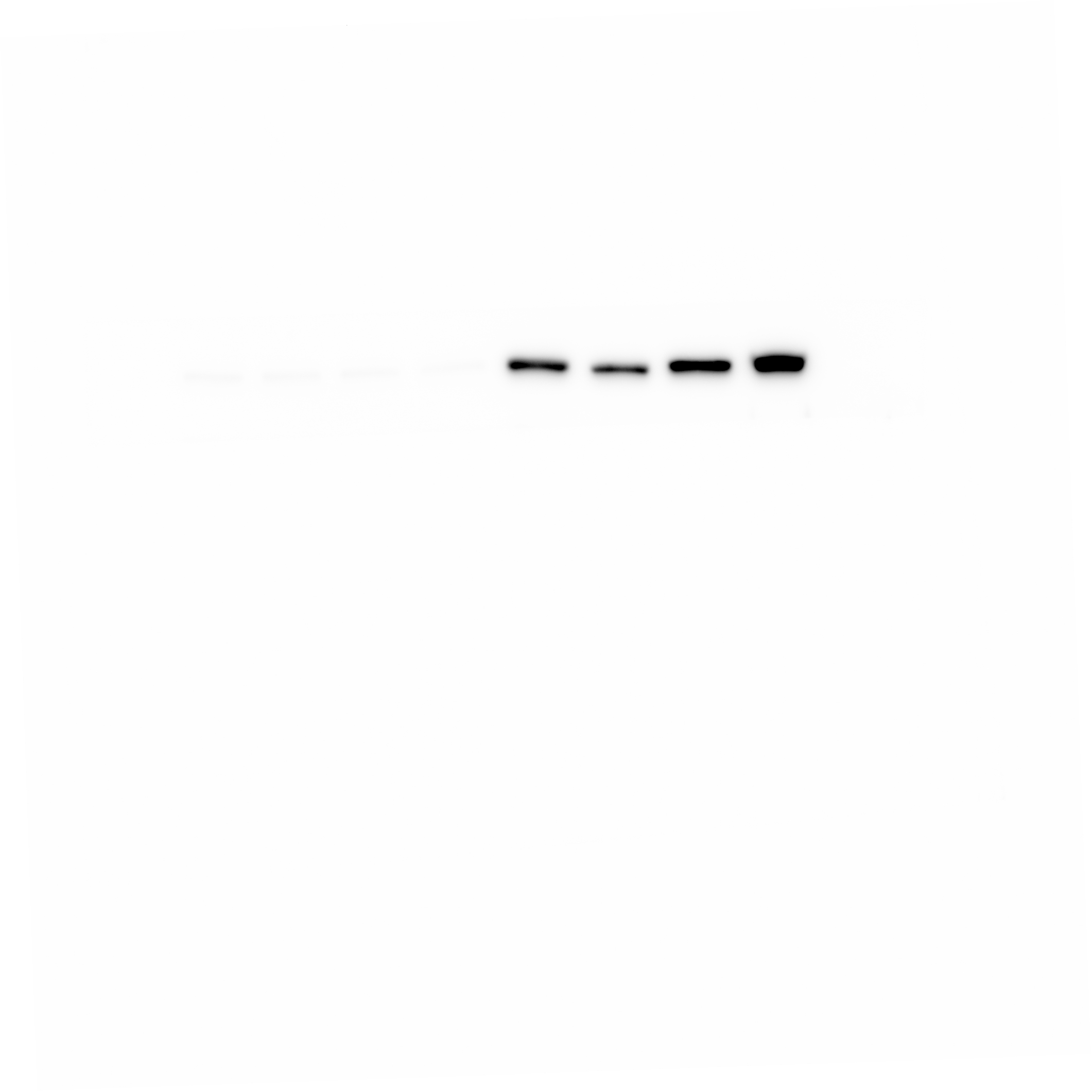

Supplement: Source data 3. [file elife-70151-data3.zip › Source data_v2/Figure 5C/Figure 5C_b-tubulin_source data.jpg]

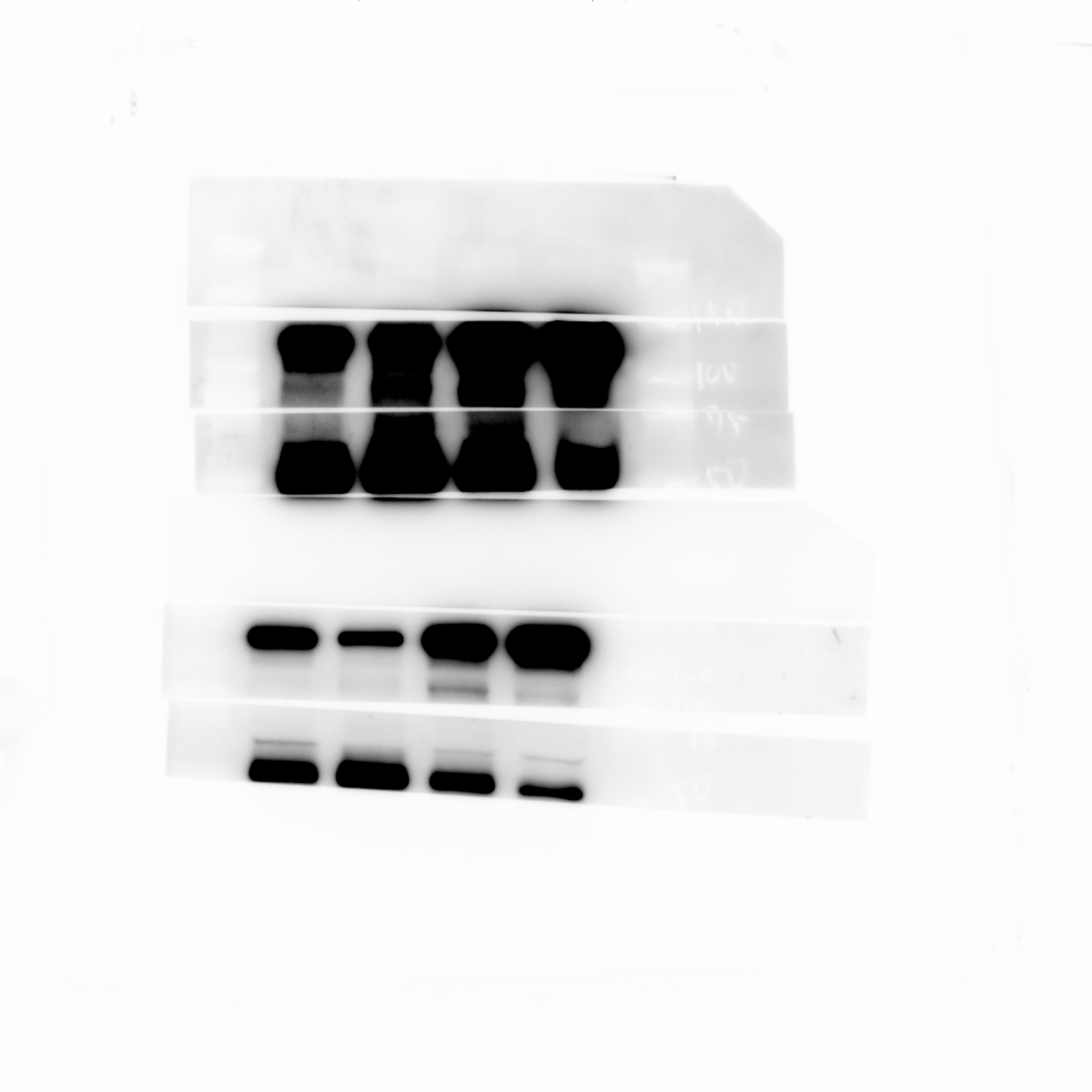

Supplement: Source data 3. [file elife-70151-data3.zip › Source data_v2/Figure 5C/Figure 5C_Luciferase_source data.jpg]

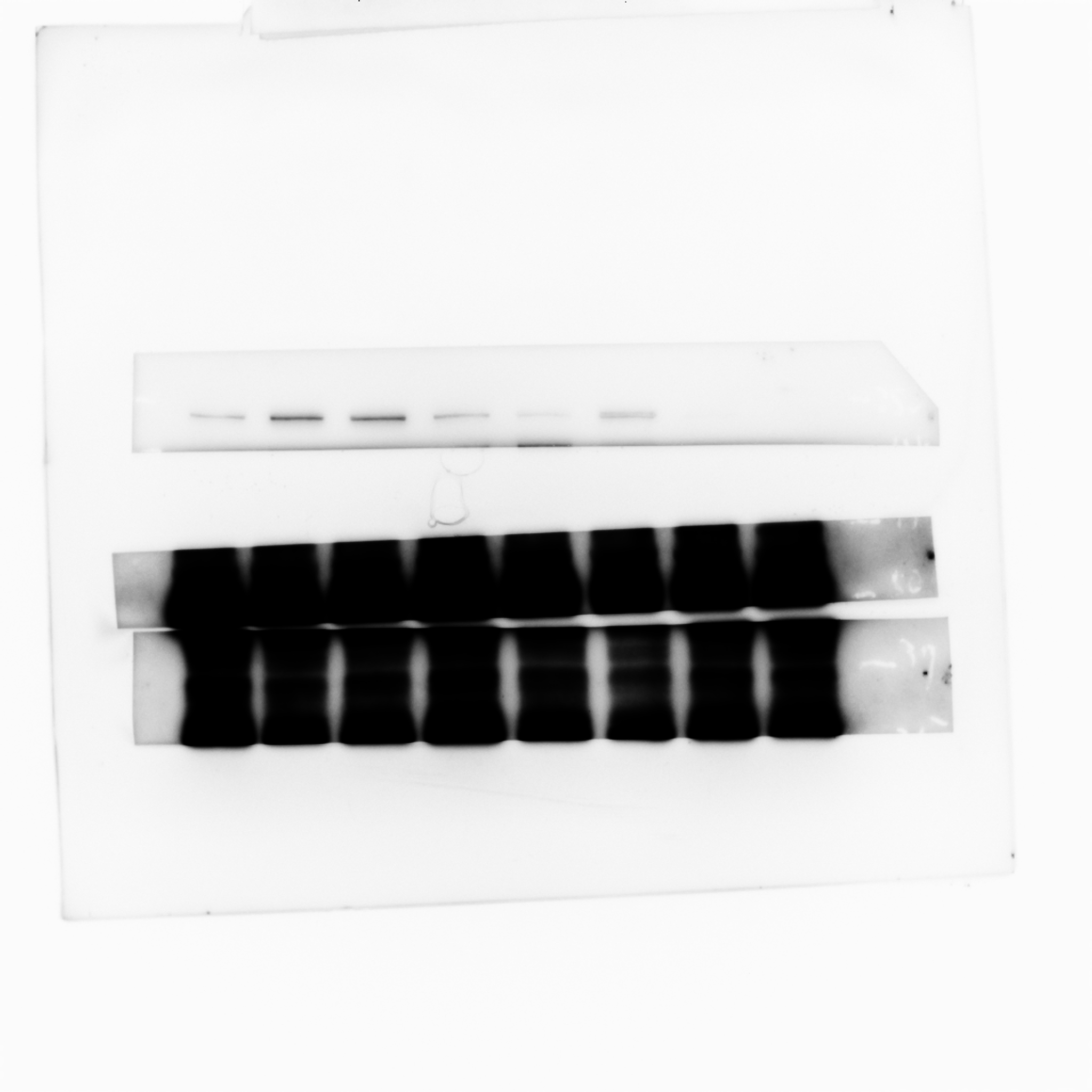

Supplement: Source data 3. [file elife-70151-data3.zip › Source data_v2/Figure 5C/Figure 5C_Zeb1_source data.jpg]

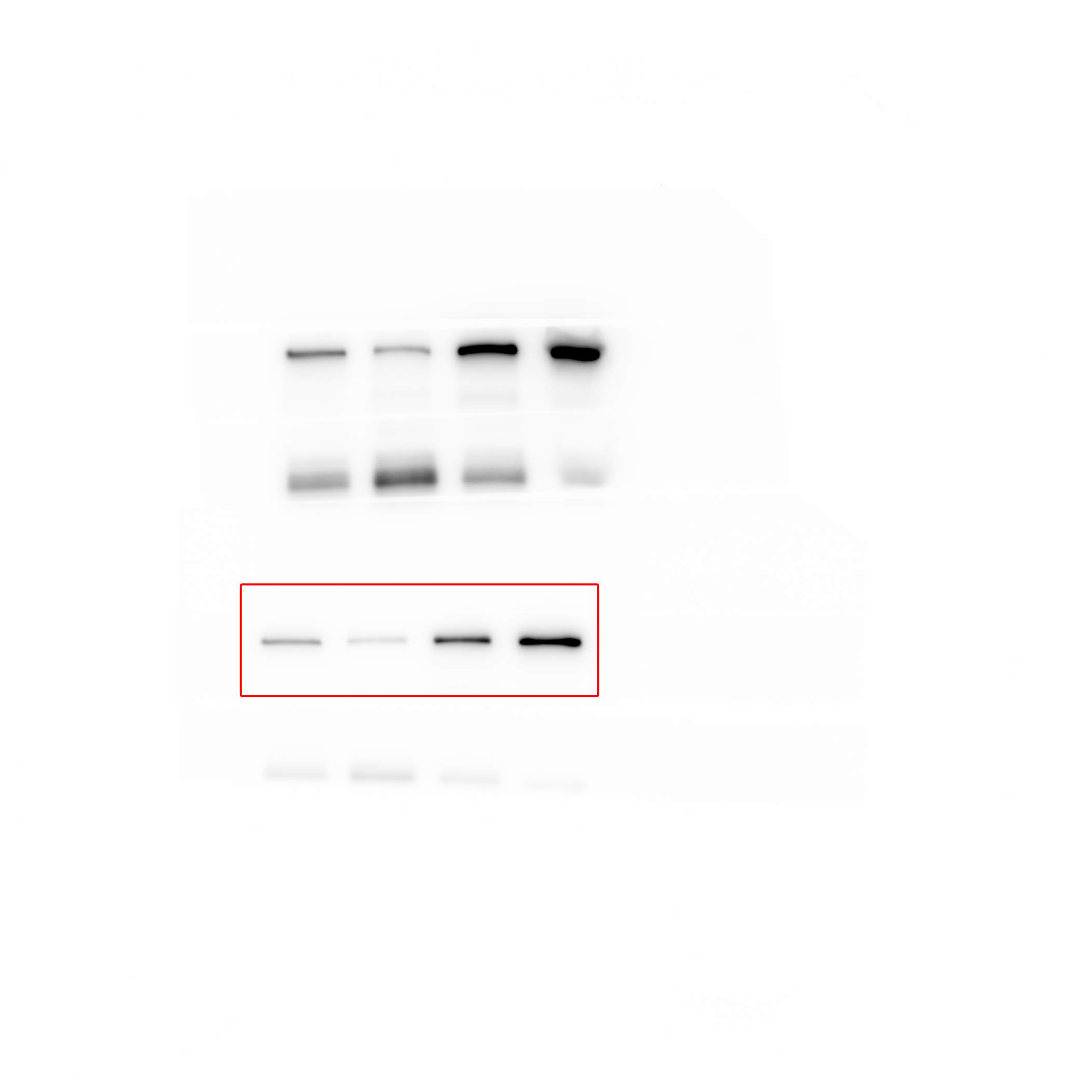

Supplement: Source data 3. [file elife-70151-data3.zip › Source data_v2/Figure 5C/Figure 5C_E-cadherin_source data.labelled]

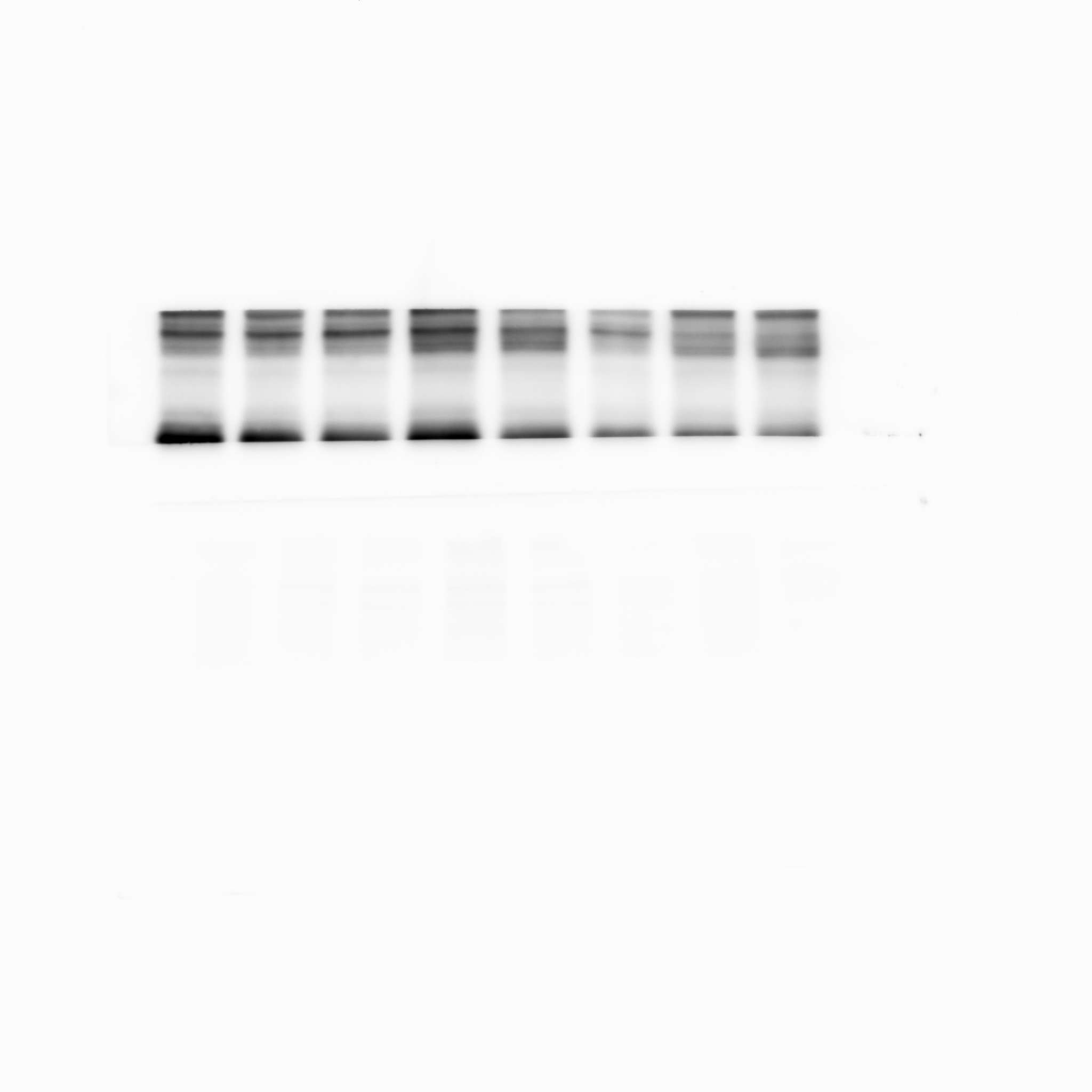

Supplement: Source data 3. [file elife-70151-data3.zip › Source data_v2/Figure 5C/Figure 5C_p-GSK3b_source data.jpg]

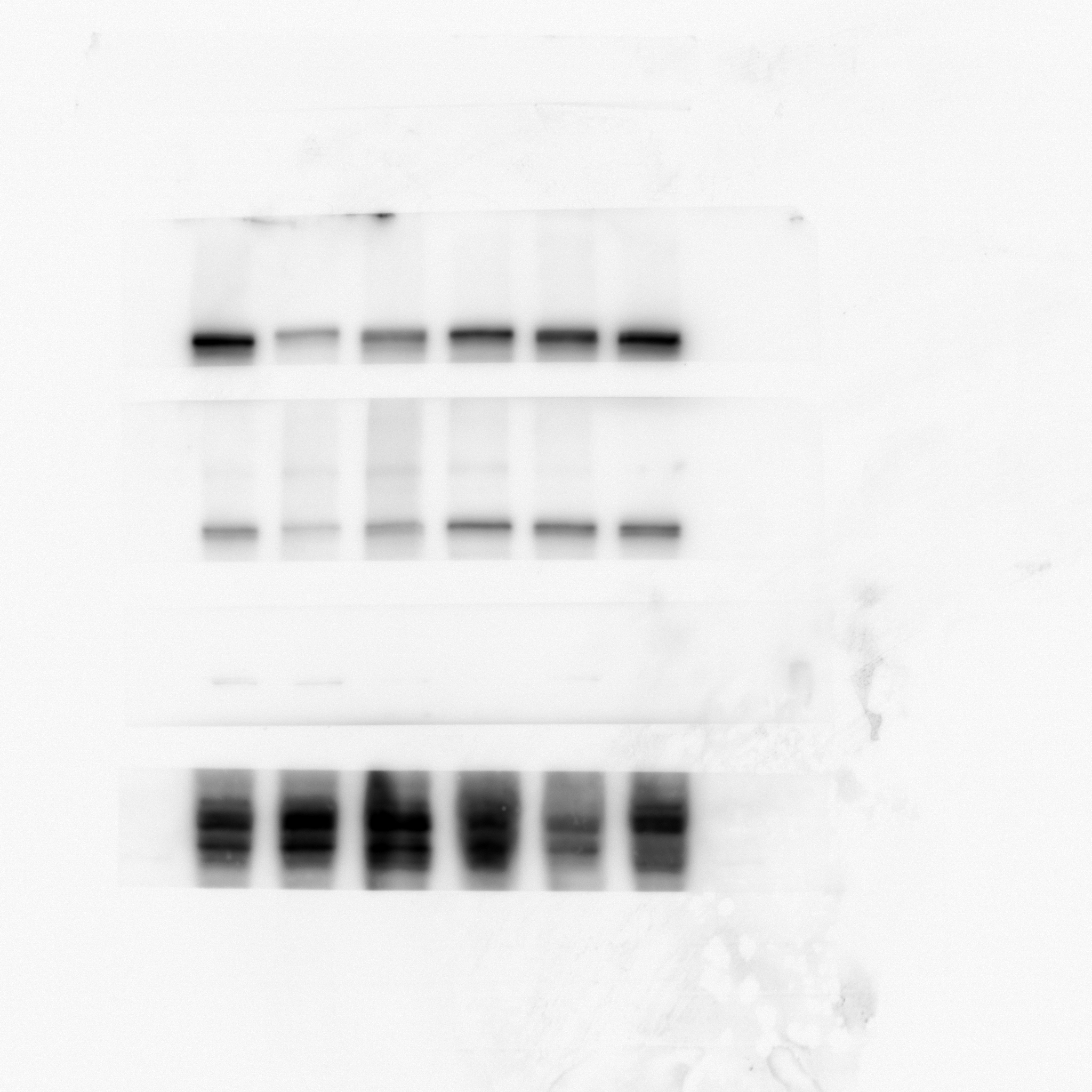

Supplement: Source data 3. [file elife-70151-data3.zip › Source data_v2/Figure 5C/Figure 5C_b-catenin_cytoplasm_source data.jpg]

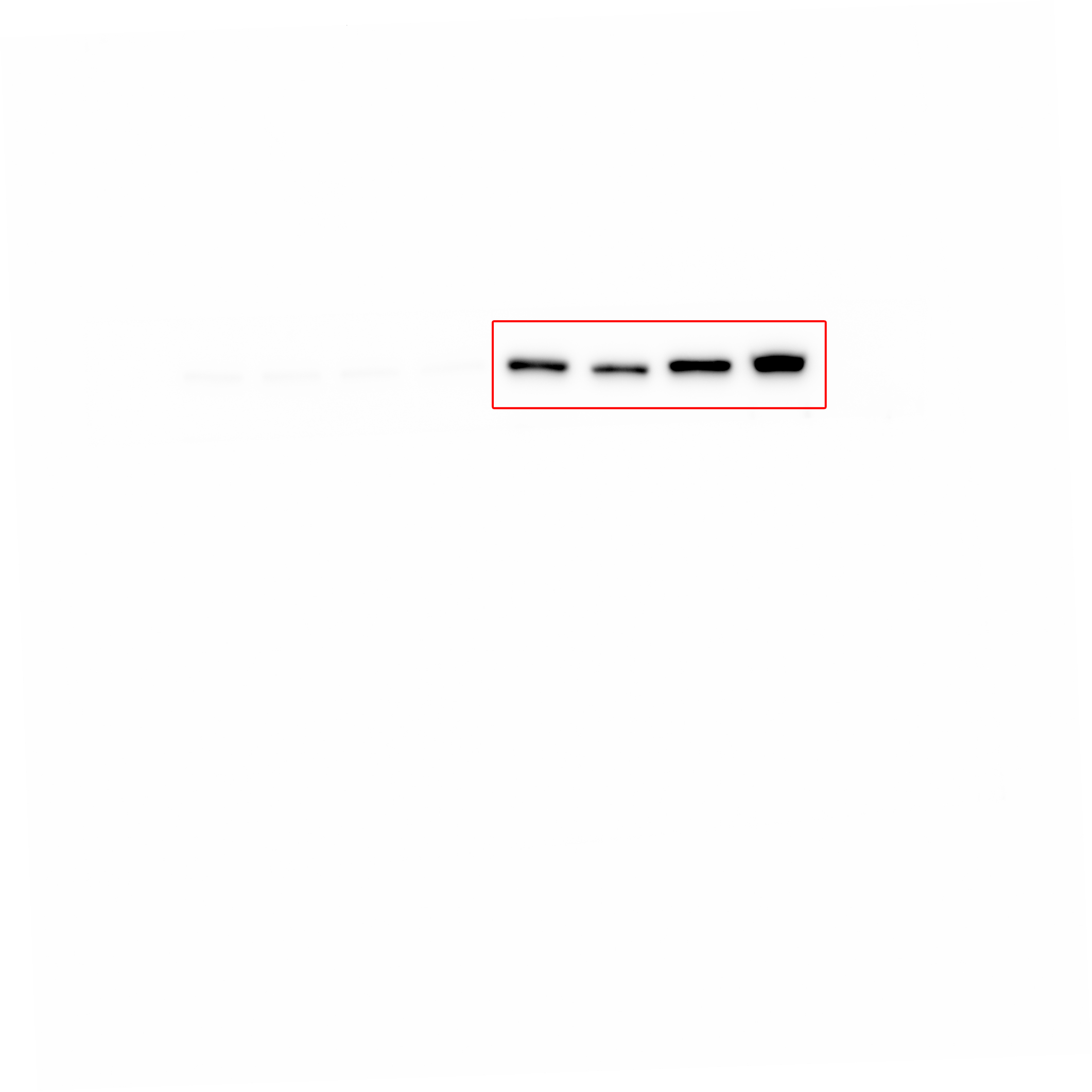

Supplement: Source data 3. [file elife-70151-data3.zip › Source data_v2/Figure 5C/Figure 5C_b-tubulin_source data_labelled.jpg]

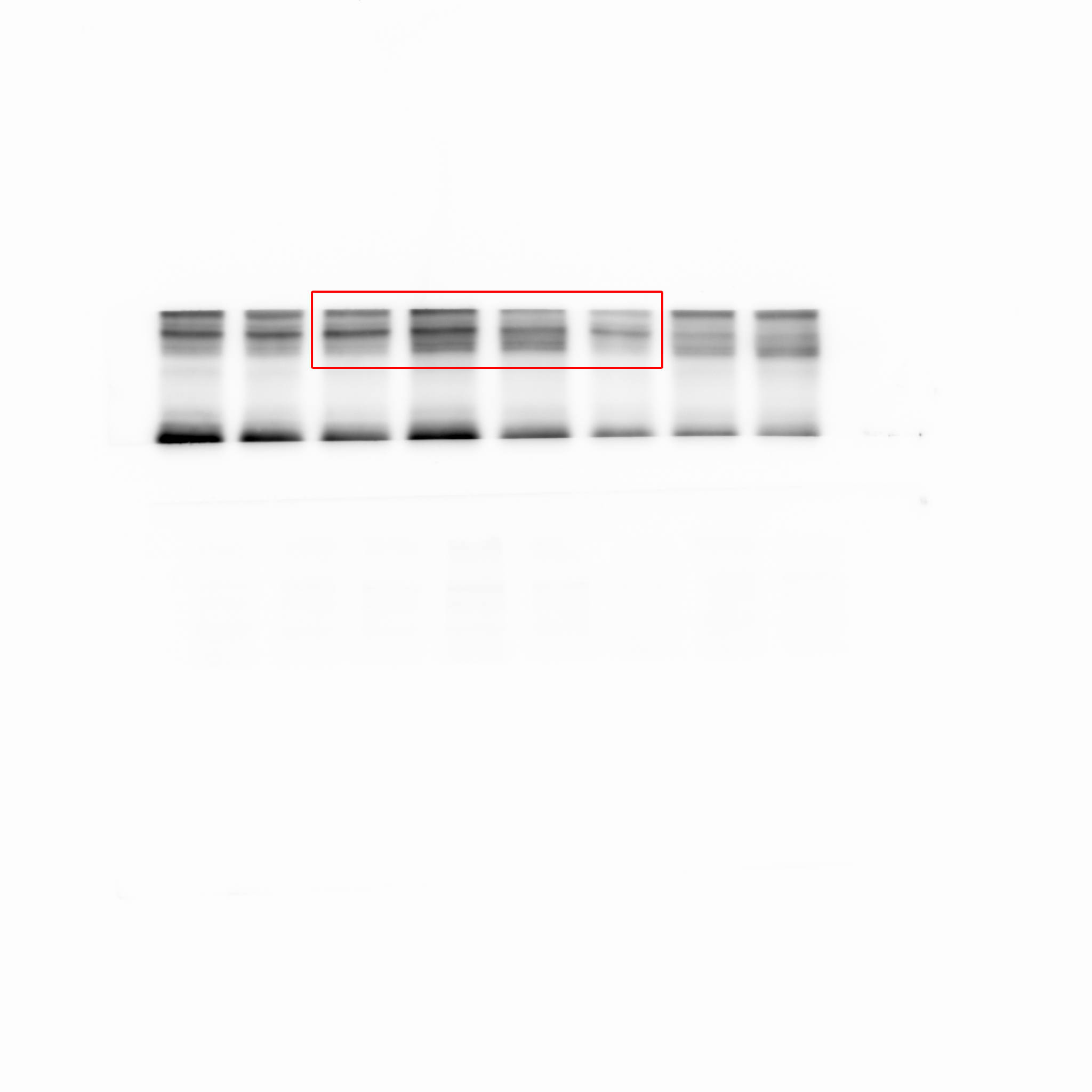

Supplement: Source data 3. [file elife-70151-data3.zip › Source data_v2/Figure 5C/Figure 5C_p-GSK3b_source data_labelled.jpg]

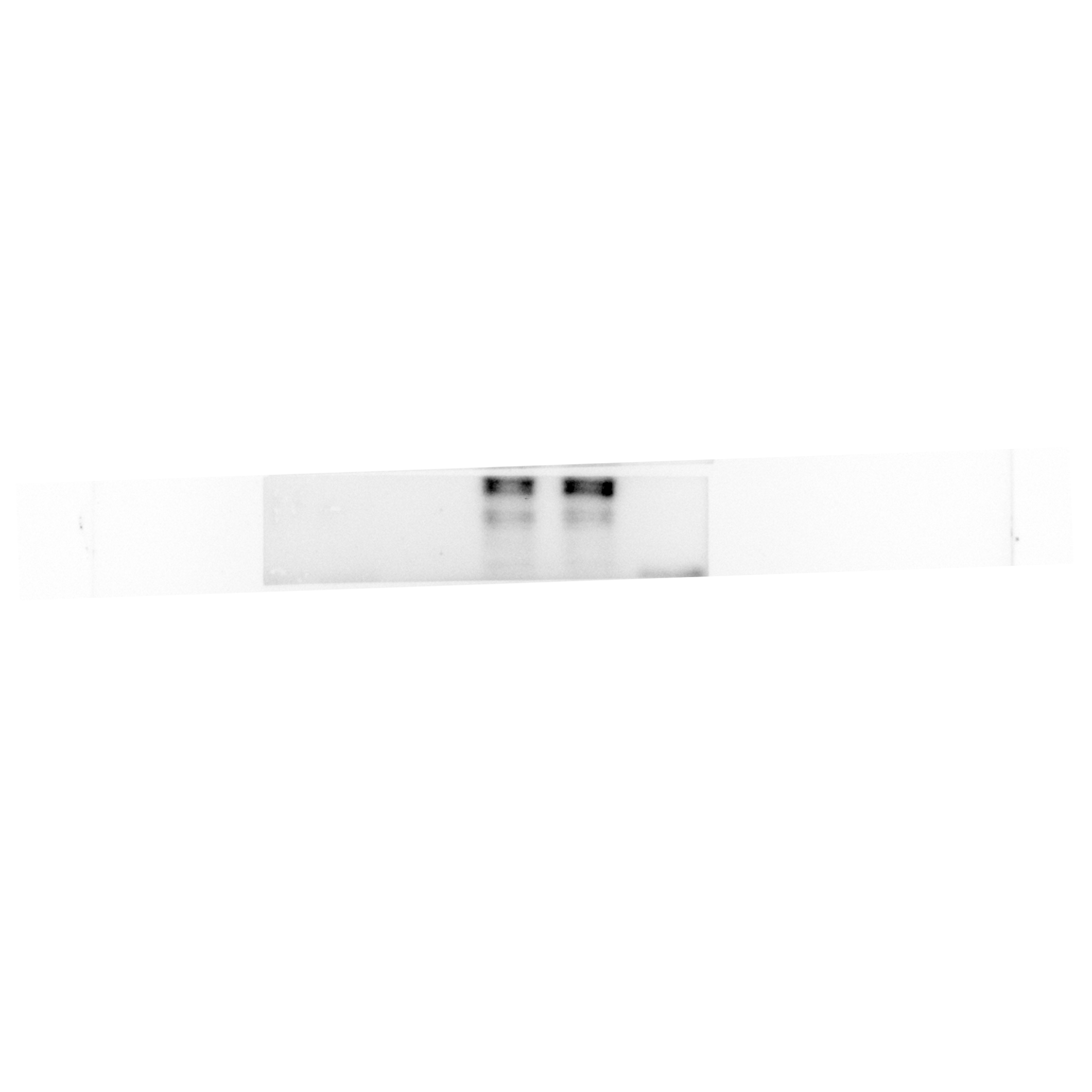

Supplement: Source data 3. [file elife-70151-data3.zip › Source data_v2/Figure 5D/Figure 5D_EpCAM_source data.jpg]

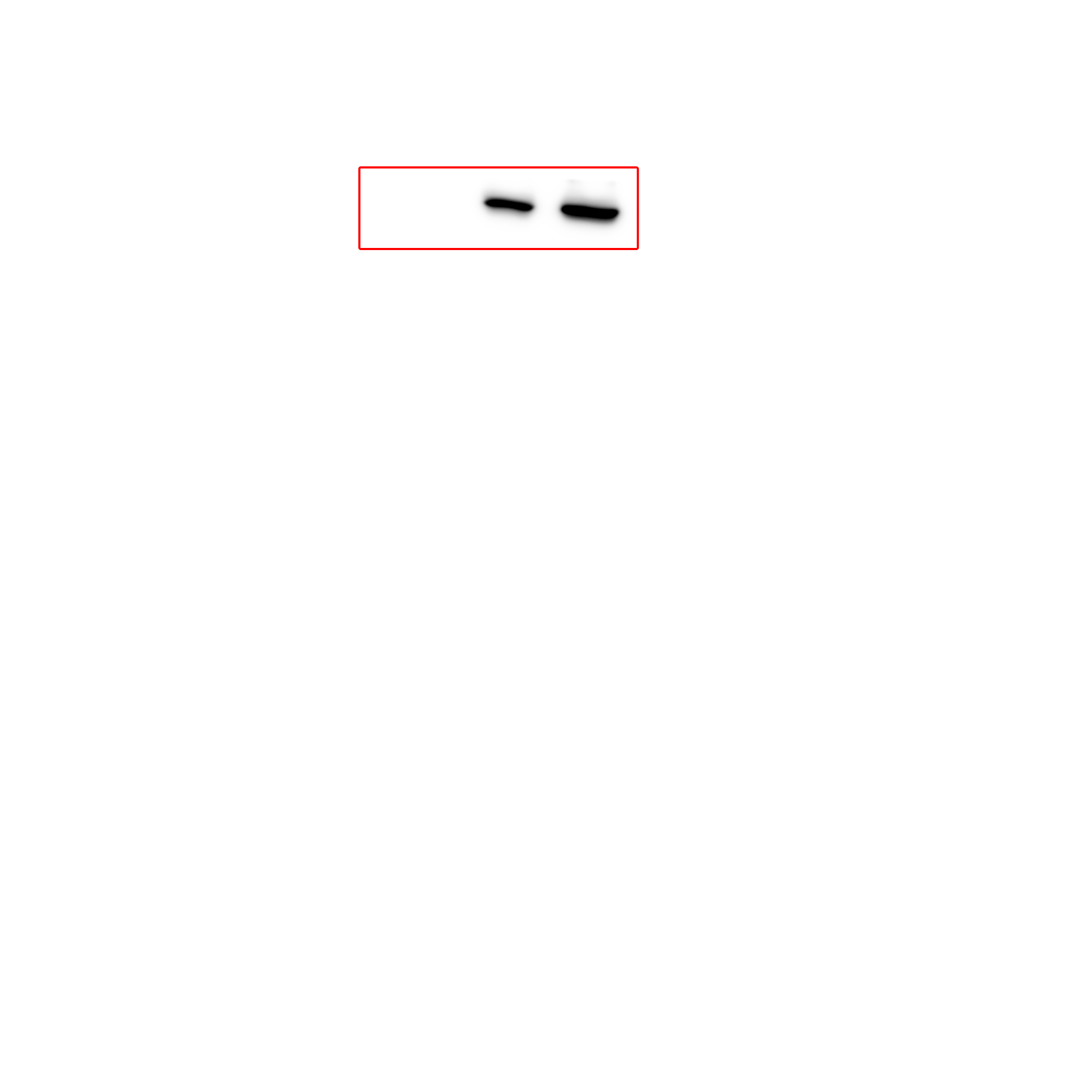

Supplement: Source data 3. [file elife-70151-data3.zip › Source data_v2/Figure 5D/Figure 5D_KRT18_source data_labelled.jpg]

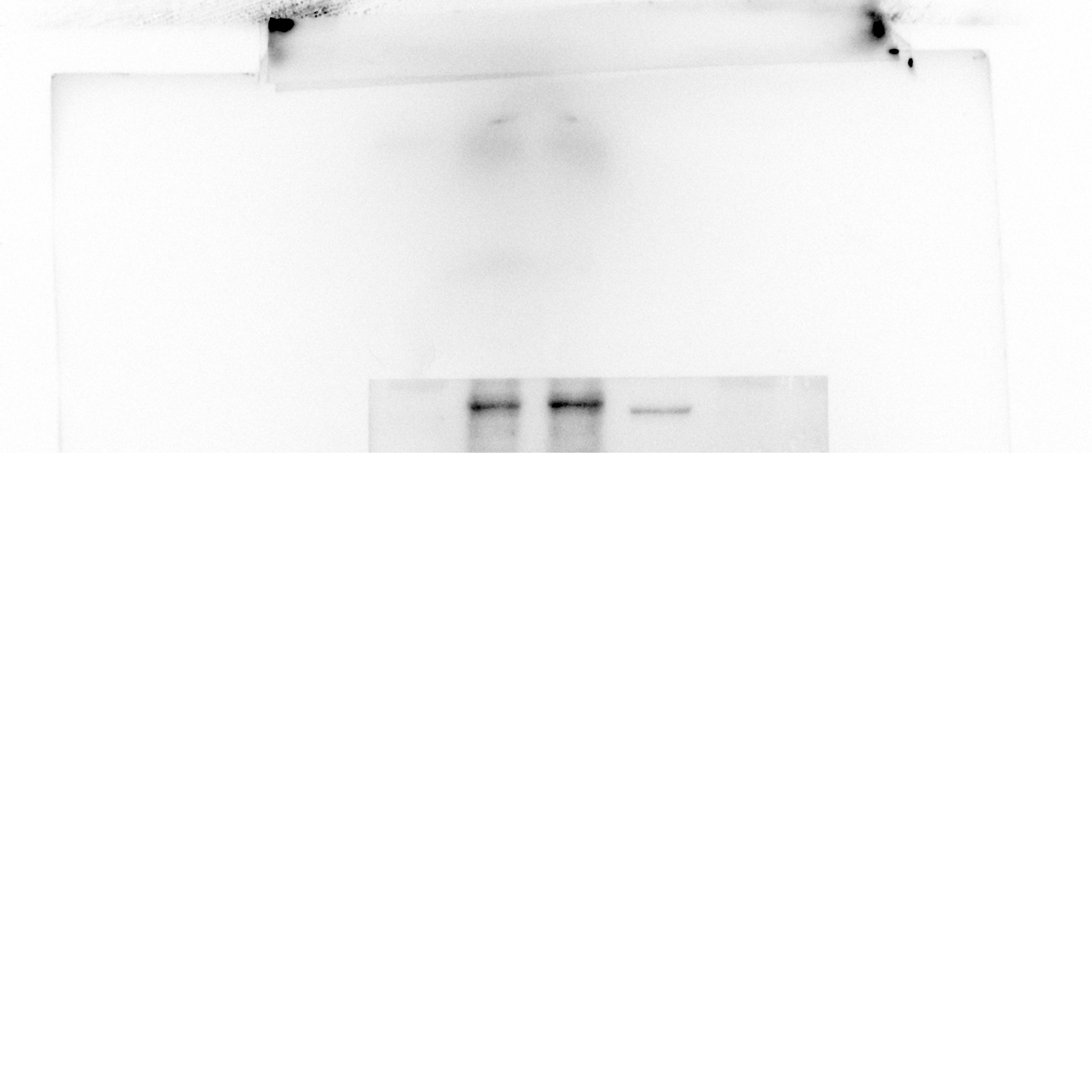

Supplement: Source data 3. [file elife-70151-data3.zip › Source data_v2/Figure 5D/Figure 5D_MMP1_source data.jpg]

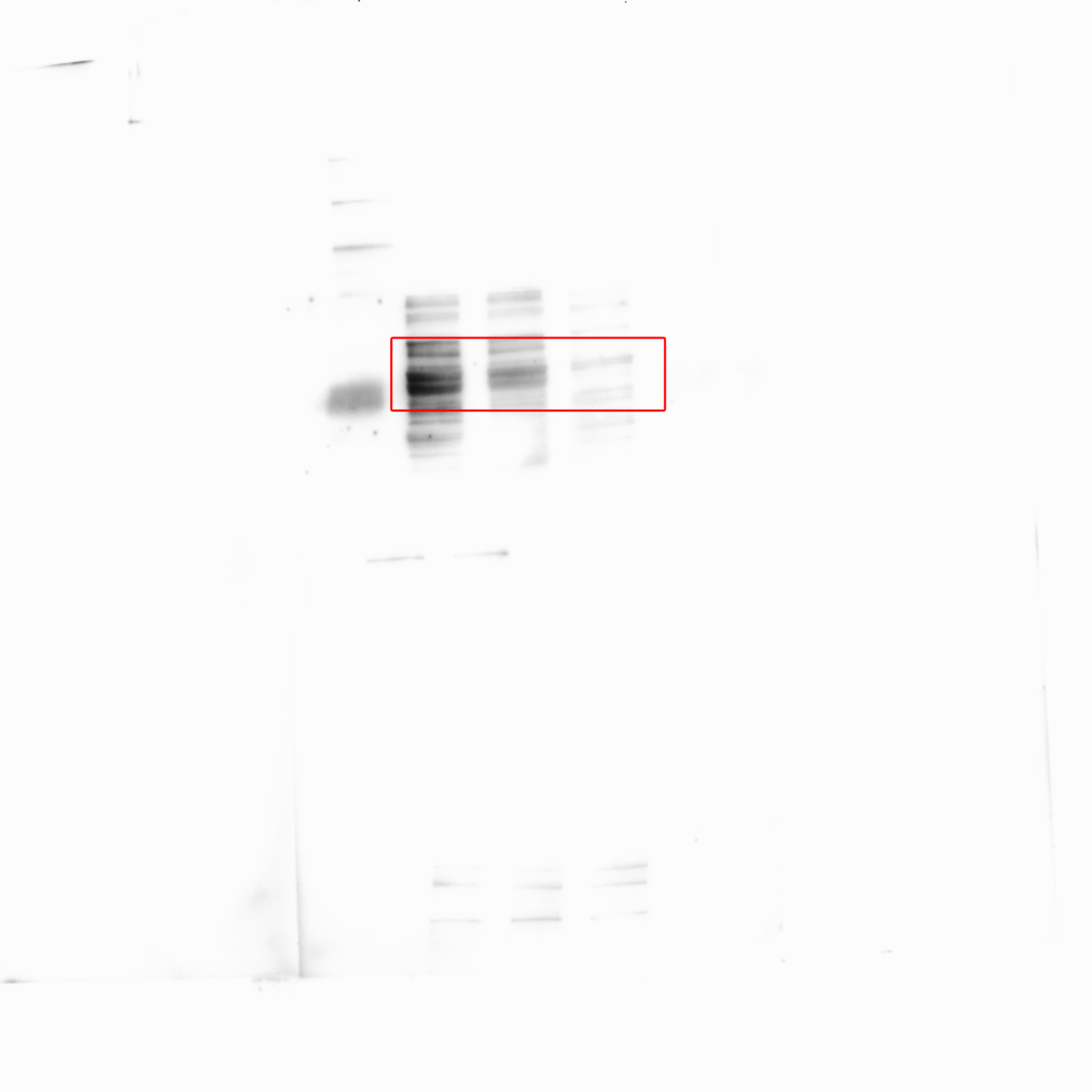

Supplement: Source data 3. [file elife-70151-data3.zip › Source data_v2/Figure 5D/Figure 5D_MMP3_source data_labelled.jpg]

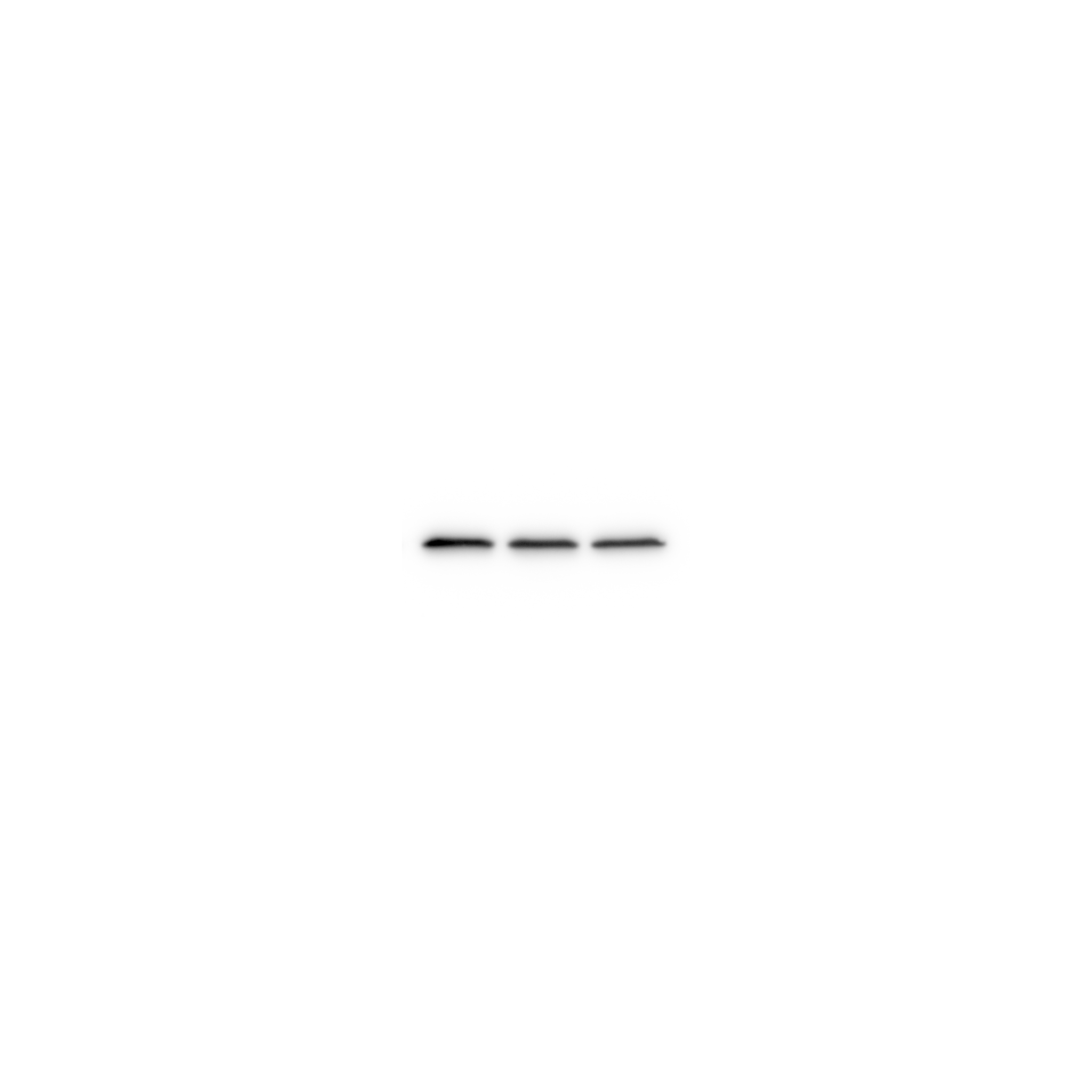

Supplement: Source data 3. [file elife-70151-data3.zip › Source data_v2/Figure 5D/Figure 5D_GAPDH_source data.jpg]

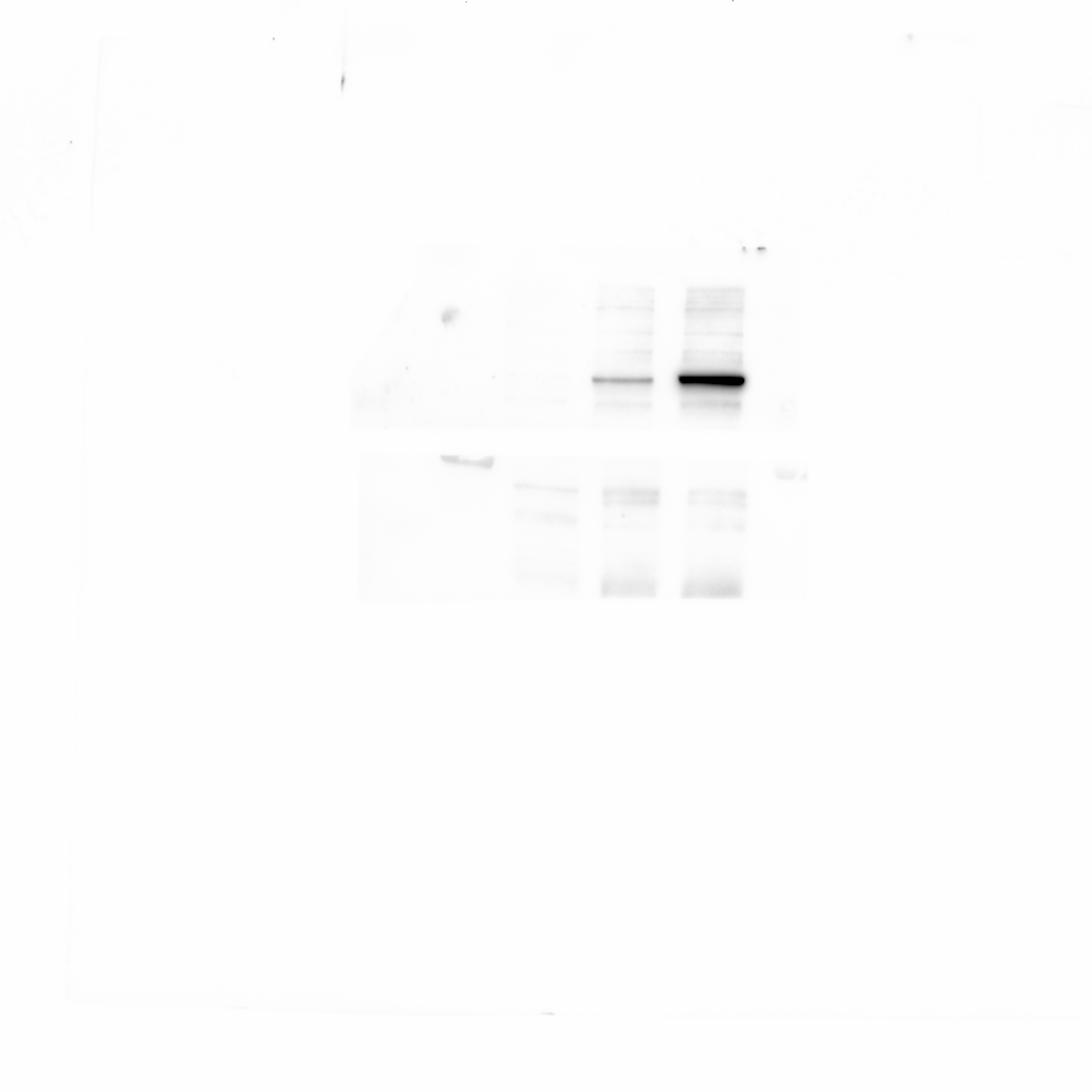

Supplement: Source data 3. [file elife-70151-data3.zip › Source data_v2/Figure 5D/Figure 5D_E-cadherin_source data.jpg]

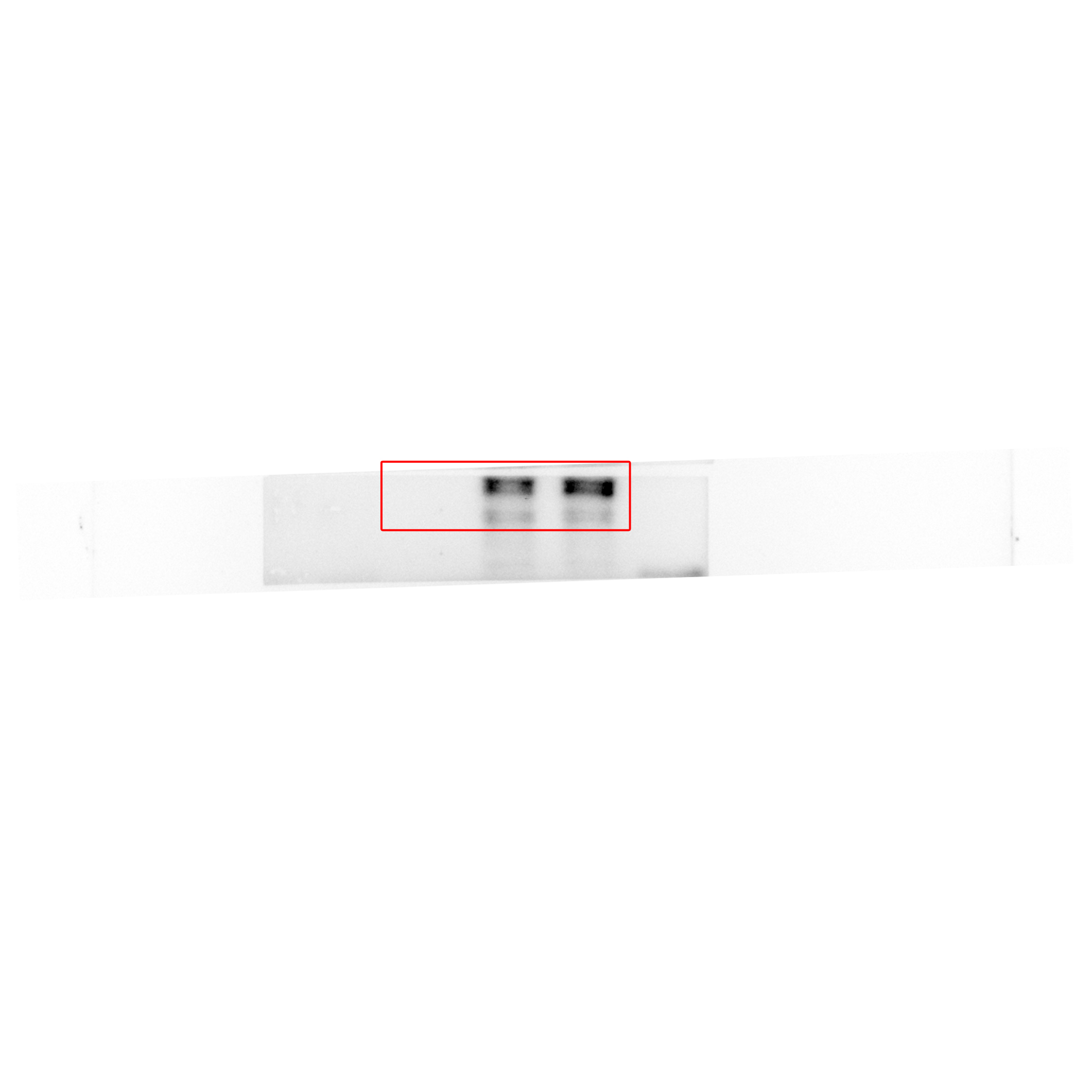

Supplement: Source data 3. [file elife-70151-data3.zip › Source data_v2/Figure 5D/Figure 5D_EpCAM_source data_labelled.jpg]

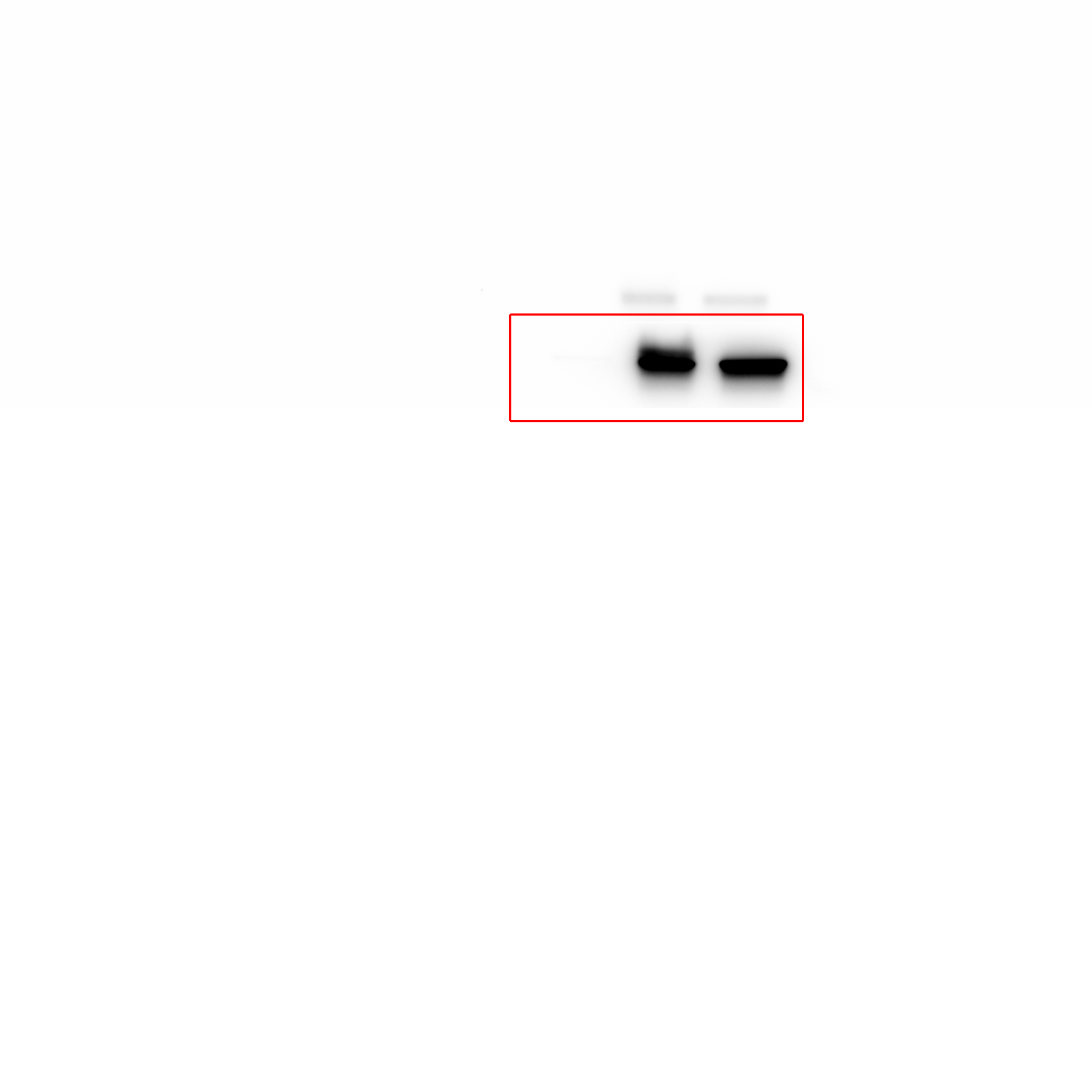

Supplement: Source data 3. [file elife-70151-data3.zip › Source data_v2/Figure 5D/Figure 5D_KRT19_source data_labelled.jpg]

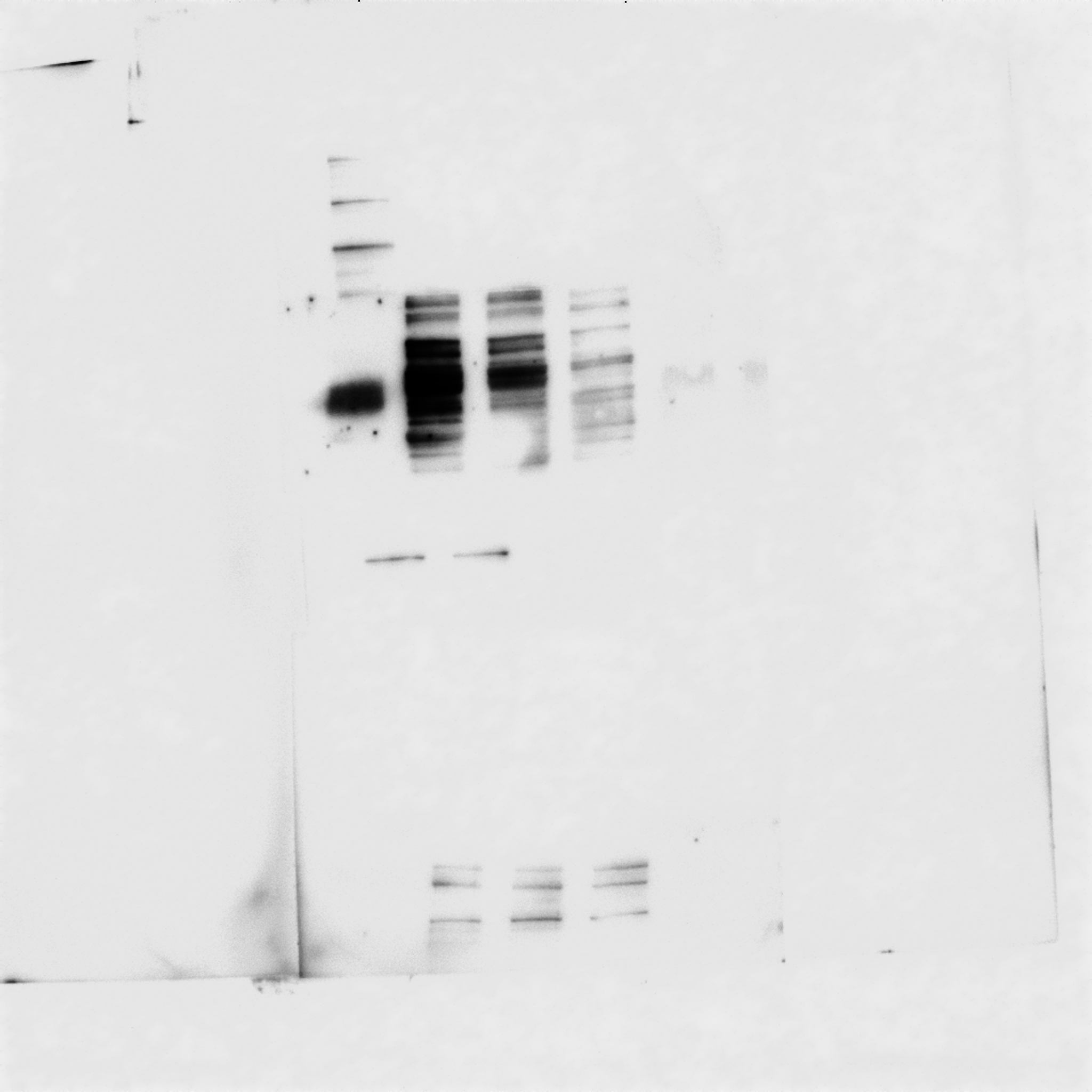

Supplement: Source data 3. [file elife-70151-data3.zip › Source data_v2/Figure 5D/Figure 5D_Vimentin_source data.jpg]

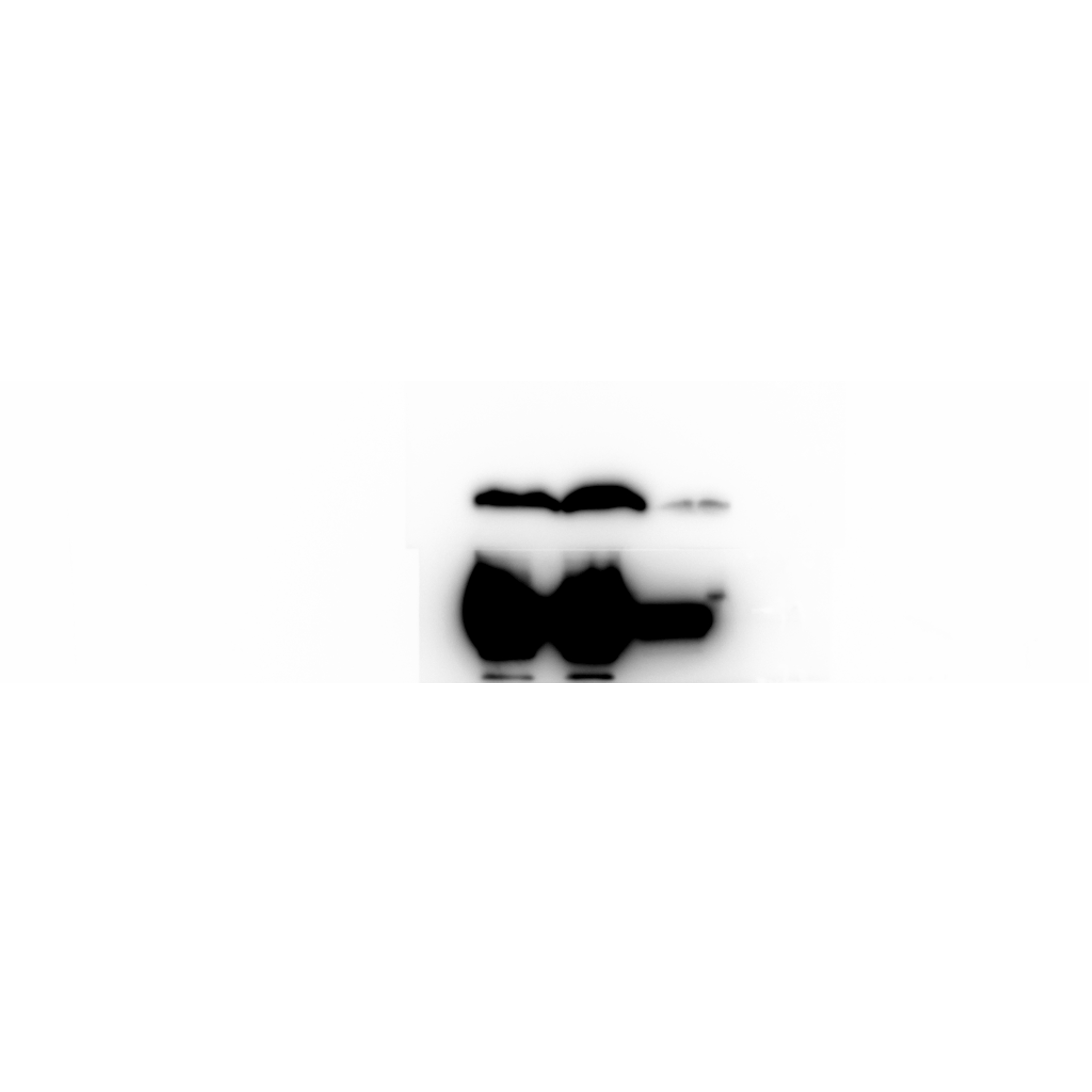

Supplement: Source data 3. [file elife-70151-data3.zip › Source data_v2/Figure 5D/Figure 5D_S100A4_source data.jpg]

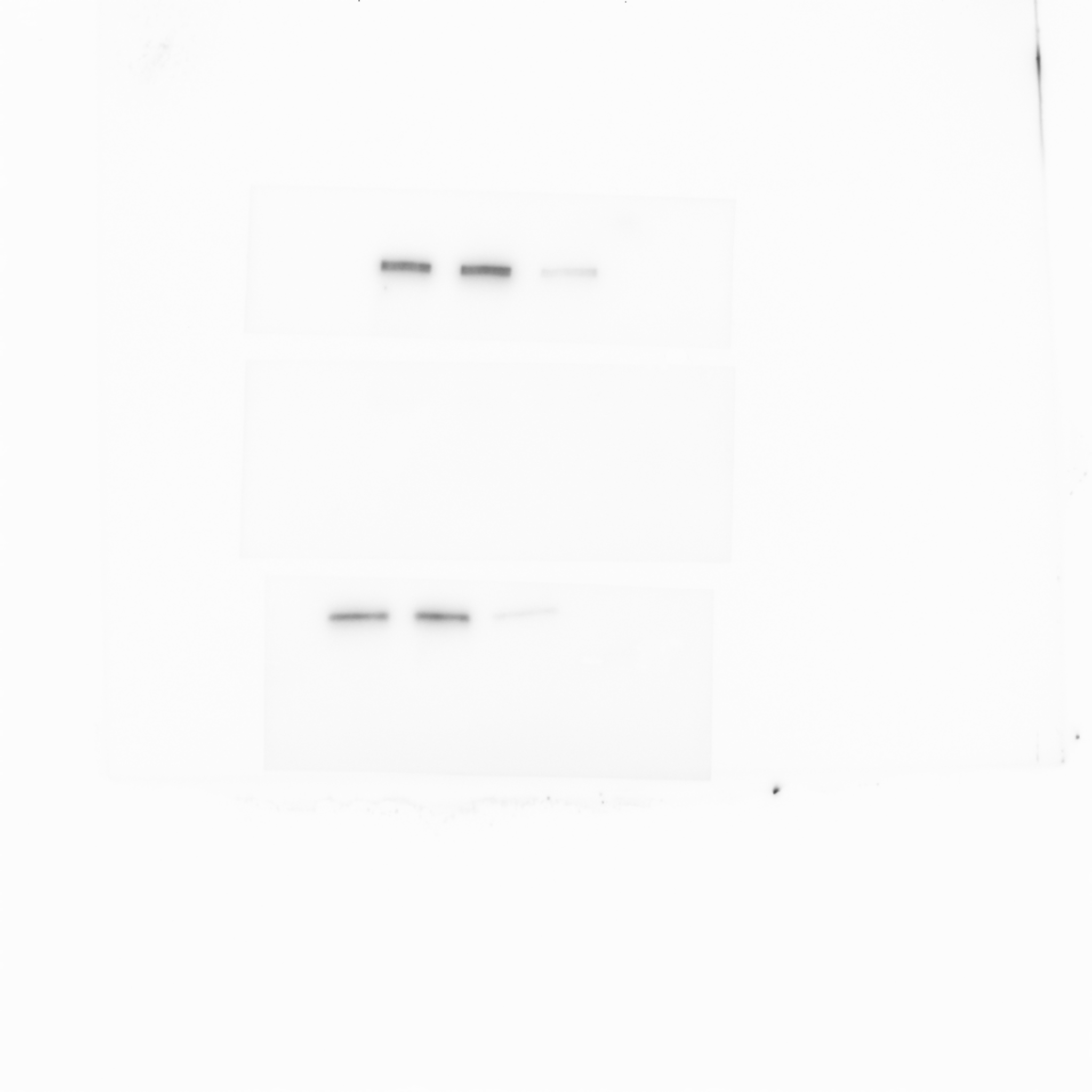

Supplement: Source data 3. [file elife-70151-data3.zip › Source data_v2/Figure 5D/Figure 5D_Zeb1_source data.jpg]

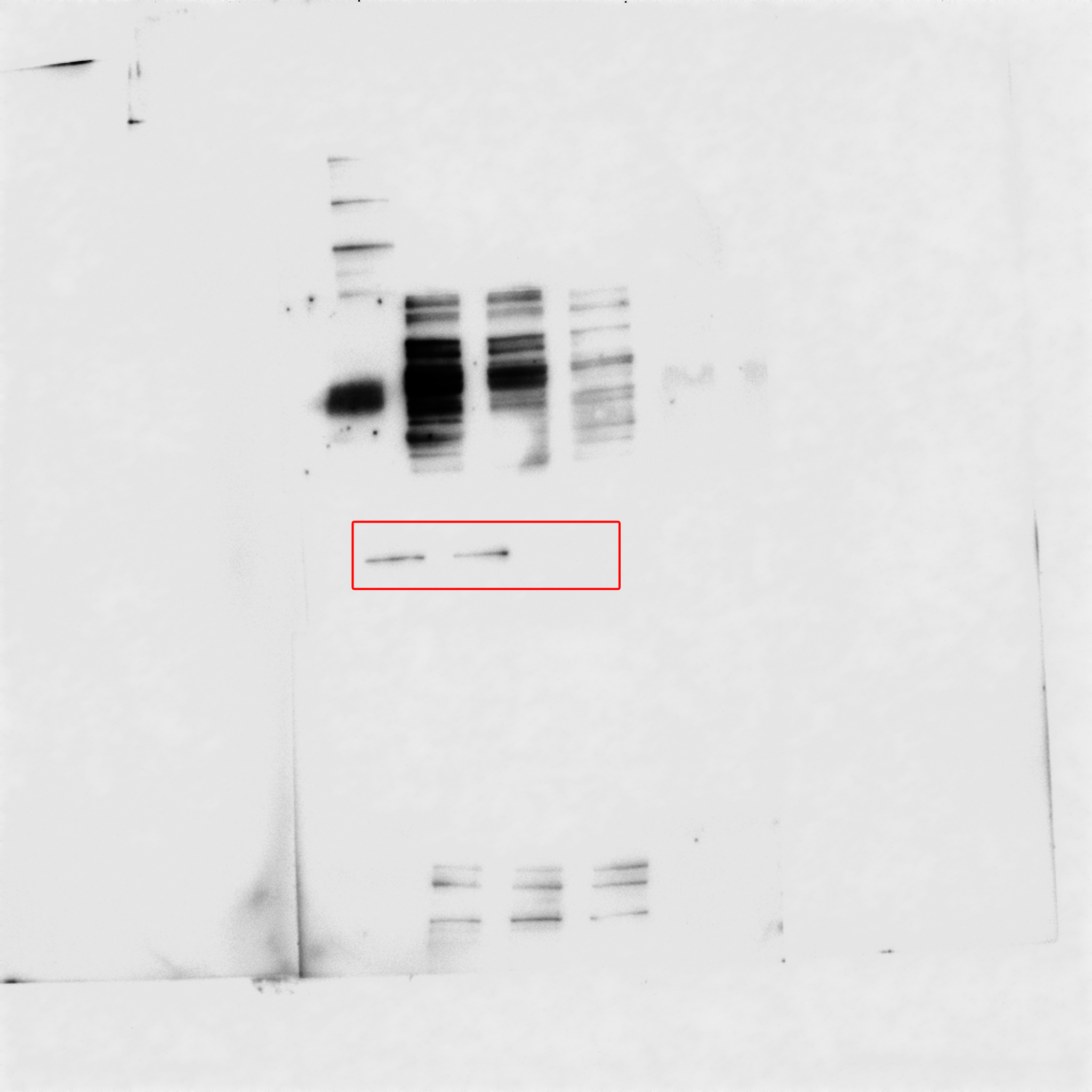

Supplement: Source data 3. [file elife-70151-data3.zip › Source data_v2/Figure 5D/Figure 5D_Vimentin_source data_labelled.jpg]

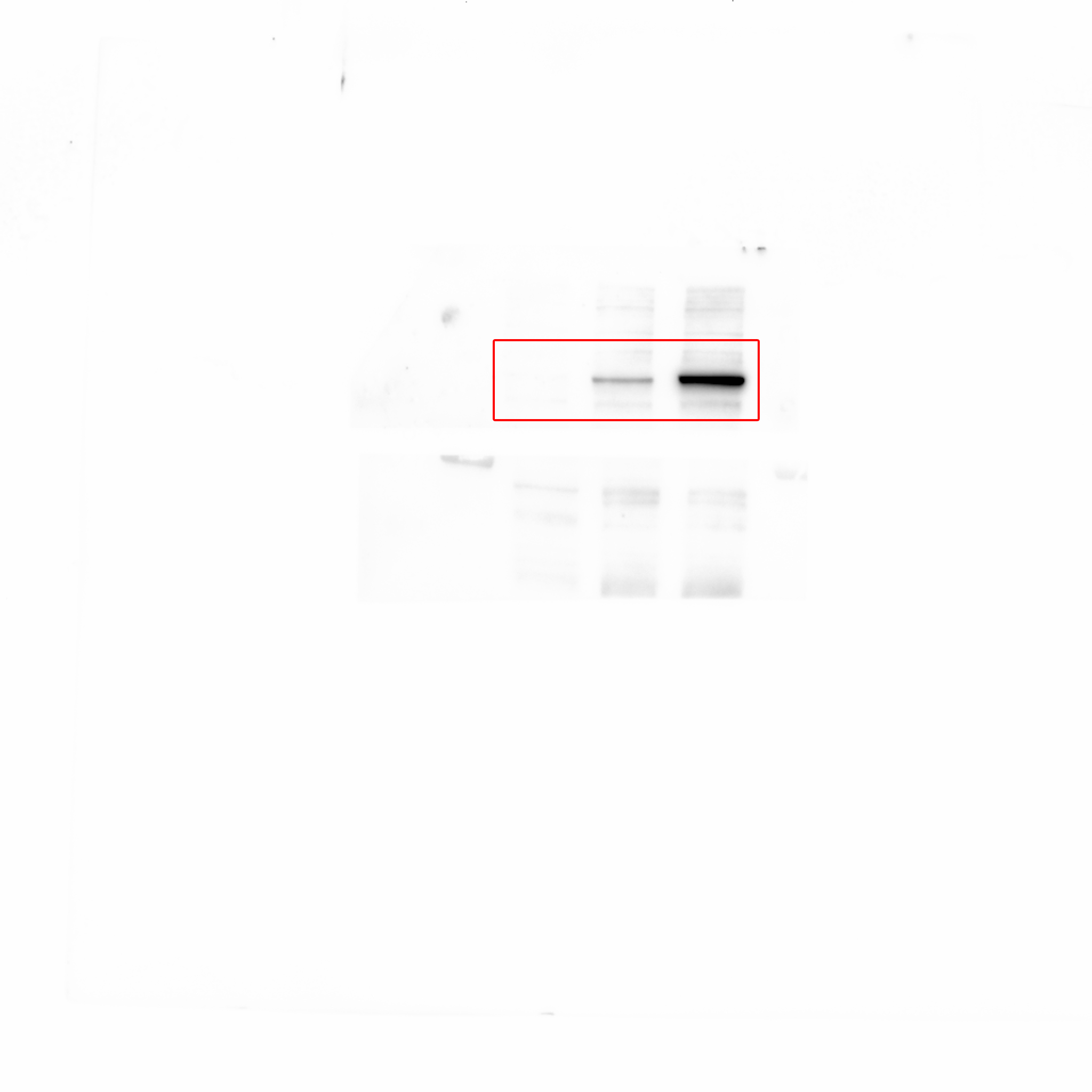

Supplement: Source data 3. [file elife-70151-data3.zip › Source data_v2/Figure 5D/Figure 5D_E-cadherin_source data_labelled.jpg]

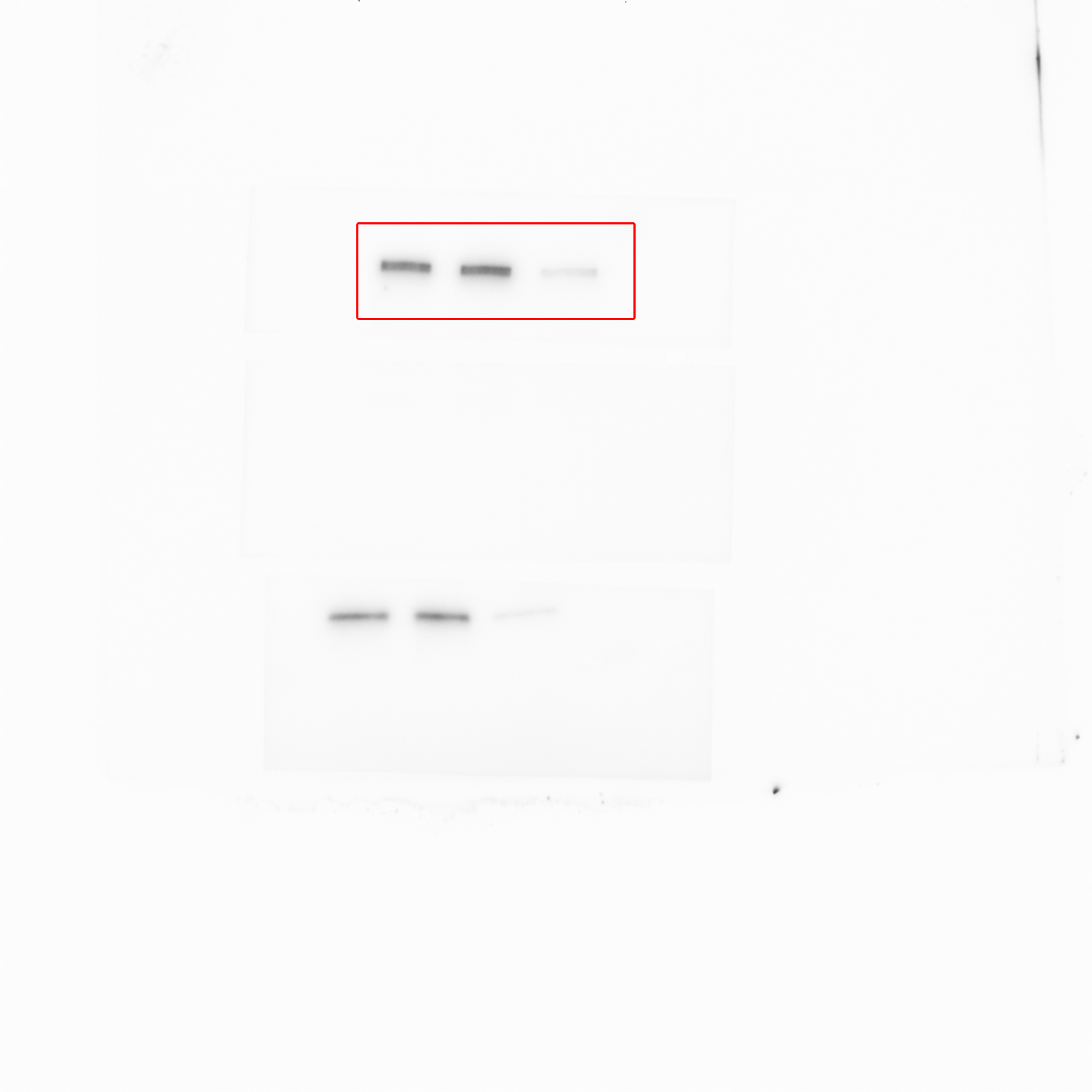

Supplement: Source data 3. [file elife-70151-data3.zip › Source data_v2/Figure 5D/Figure 5D_Zeb1_source data_labelled.jpg]

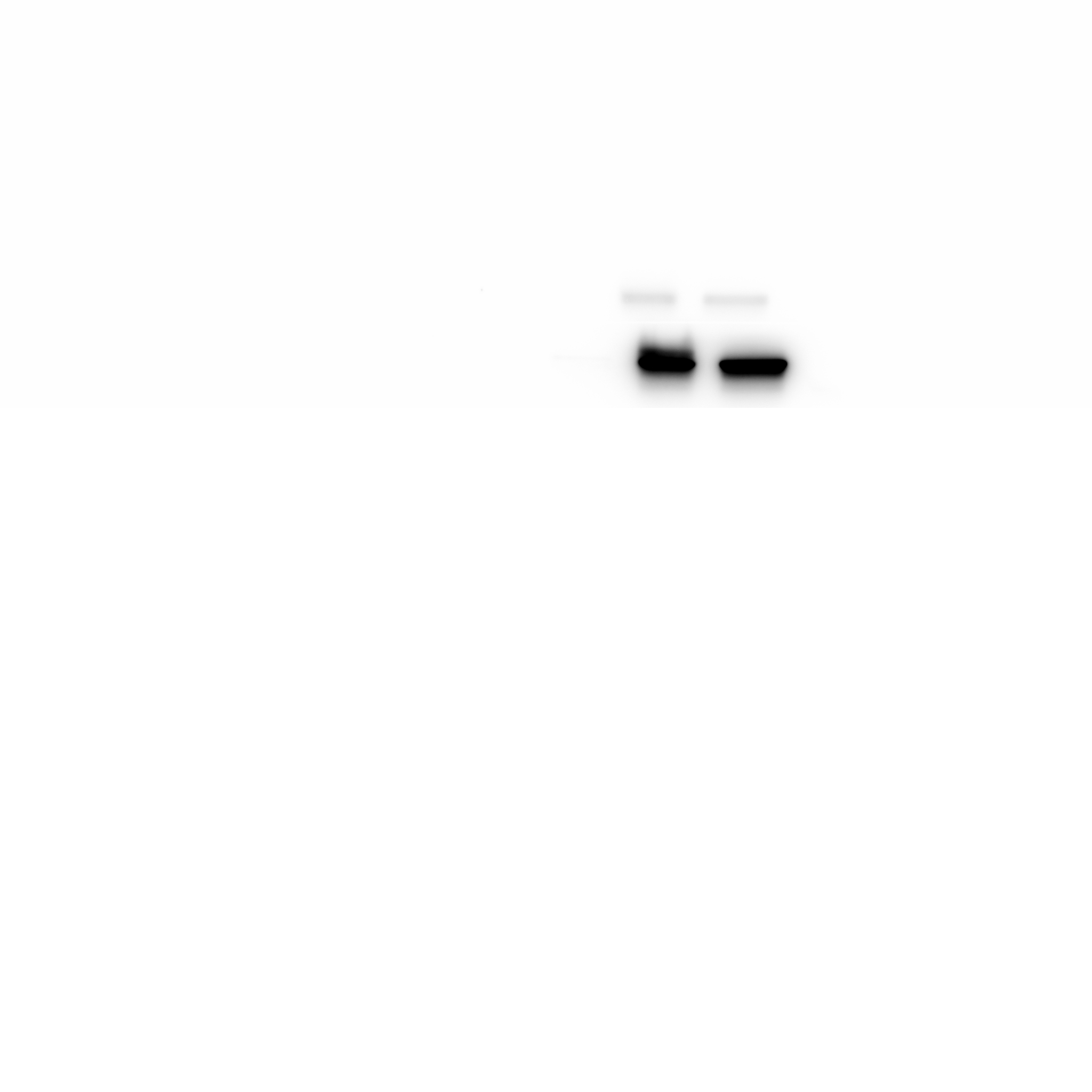

Supplement: Source data 3. [file elife-70151-data3.zip › Source data_v2/Figure 5D/Figure 5D_KRT19_source data.jpg]

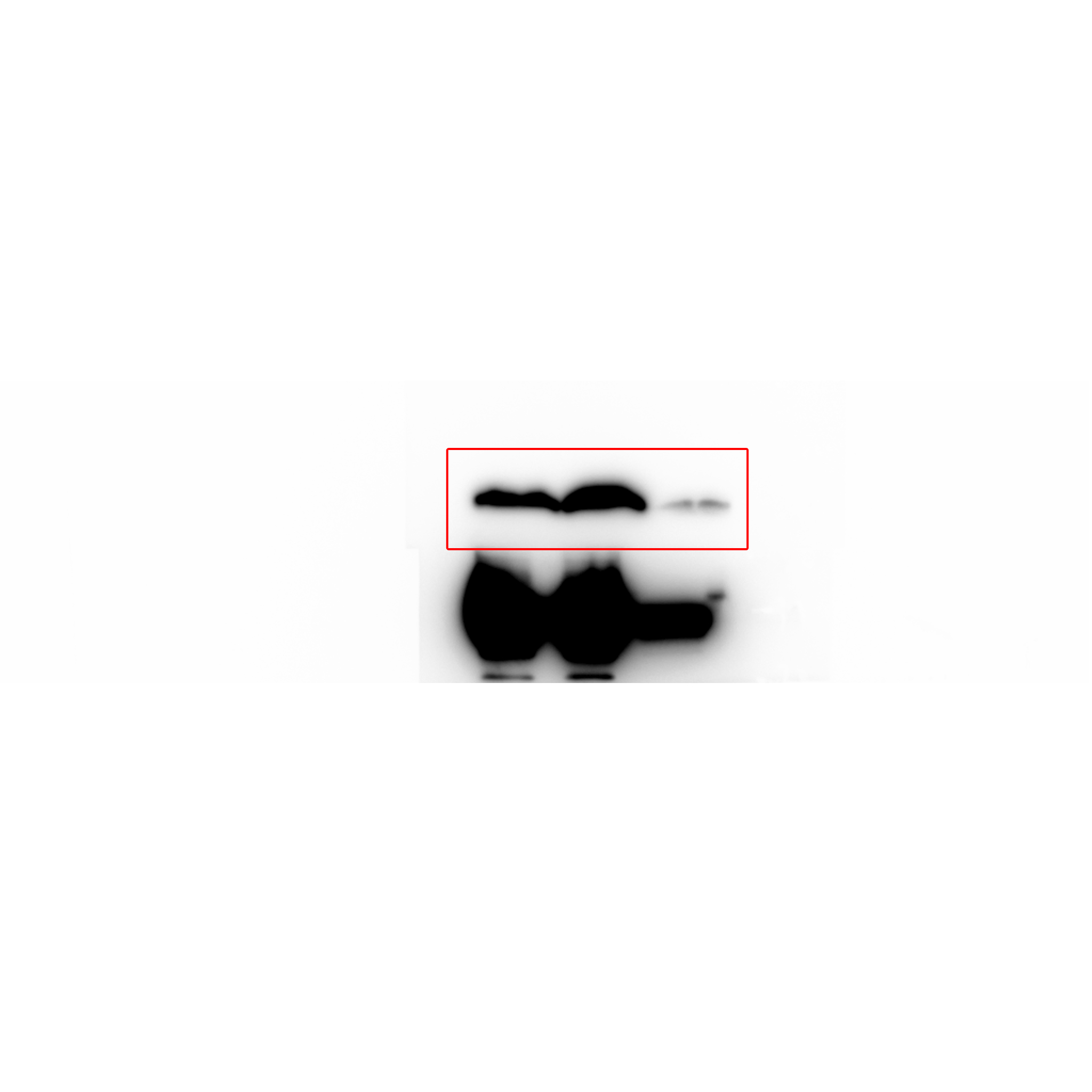

Supplement: Source data 3. [file elife-70151-data3.zip › Source data_v2/Figure 5D/Figure 5D_S100A4_source data_labelled.jpg]

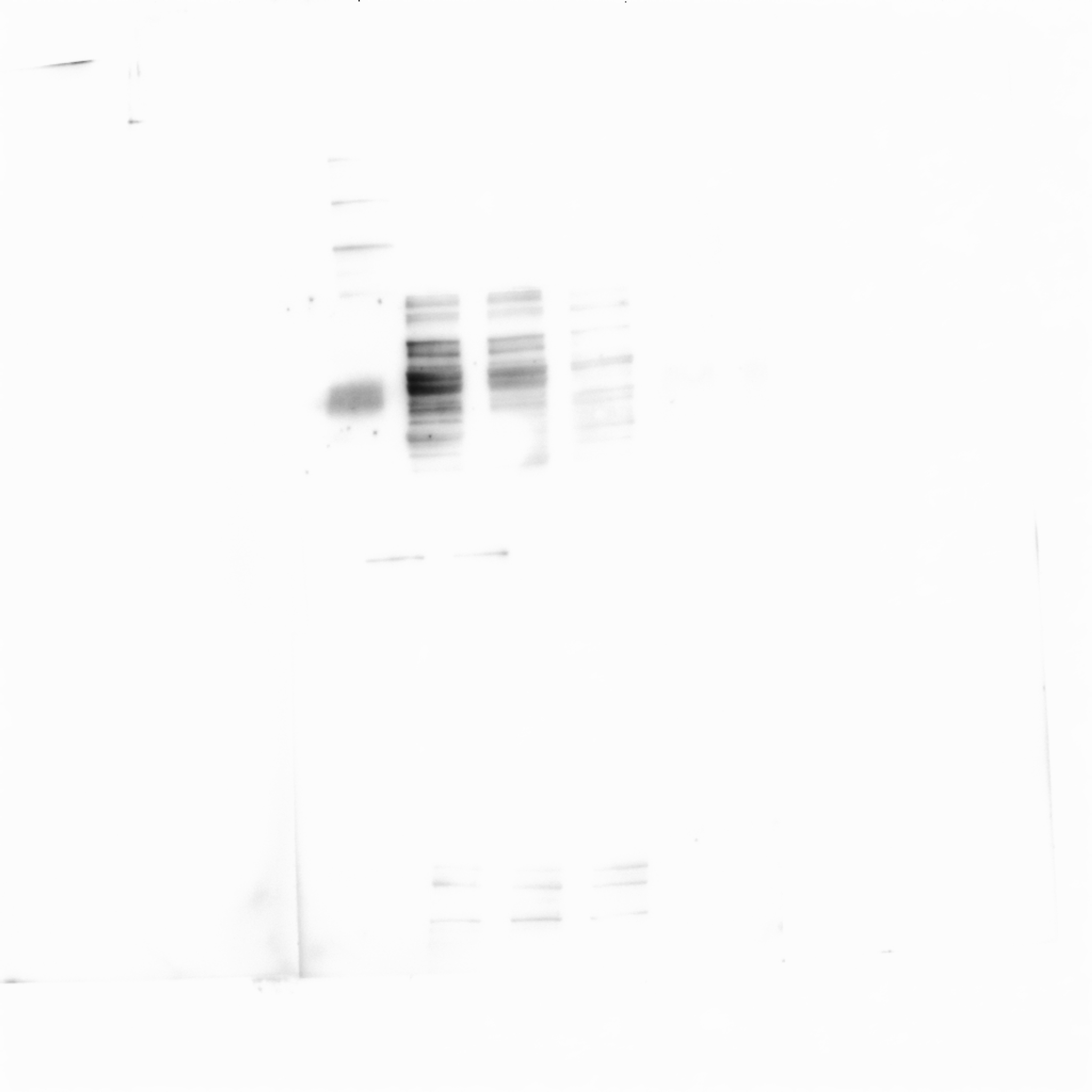

Supplement: Source data 3. [file elife-70151-data3.zip › Source data_v2/Figure 5D/Figure 5D_MMP3_source data.jpg]

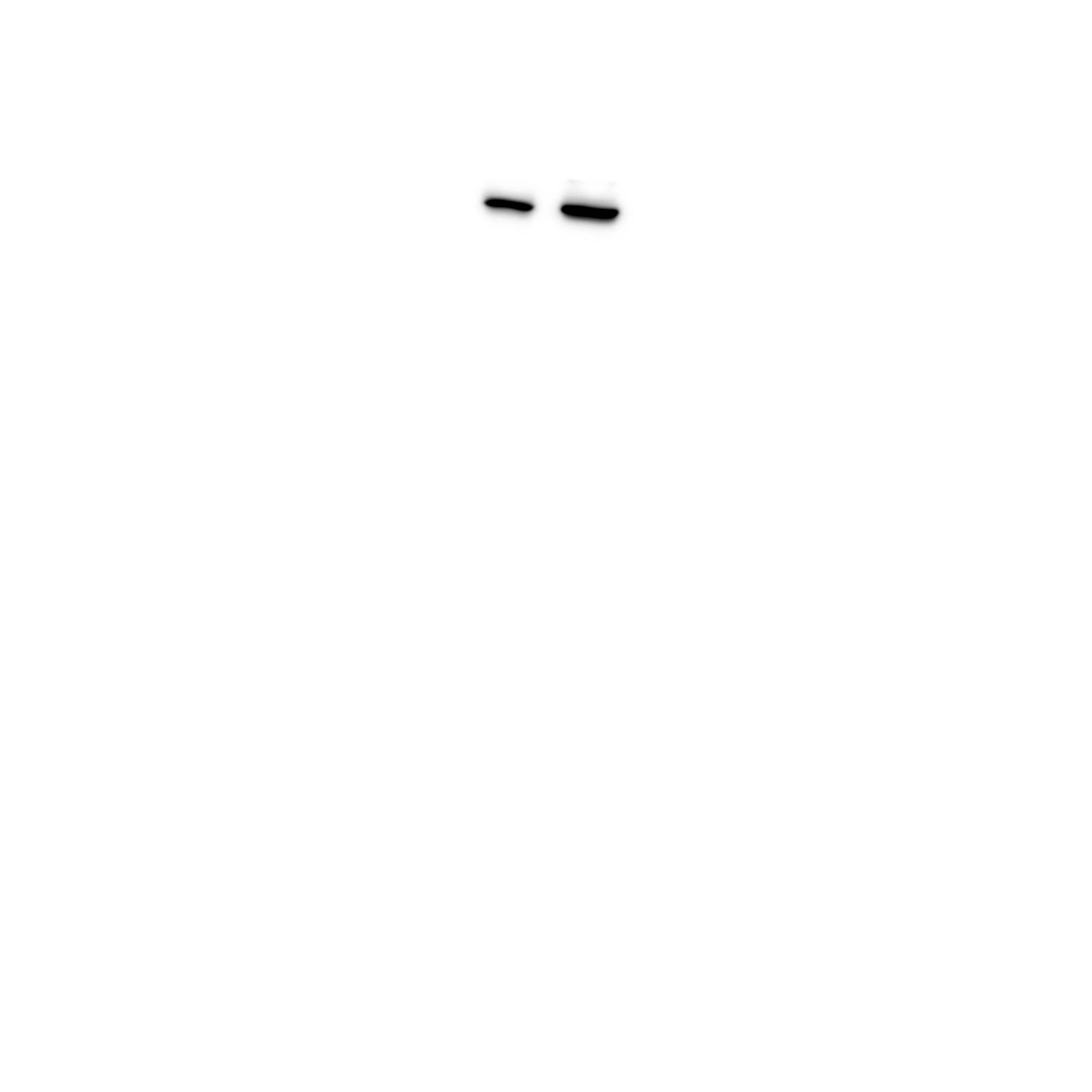

Supplement: Source data 3. [file elife-70151-data3.zip › Source data_v2/Figure 5D/Figure 5D_KRT18_source data.jpg]

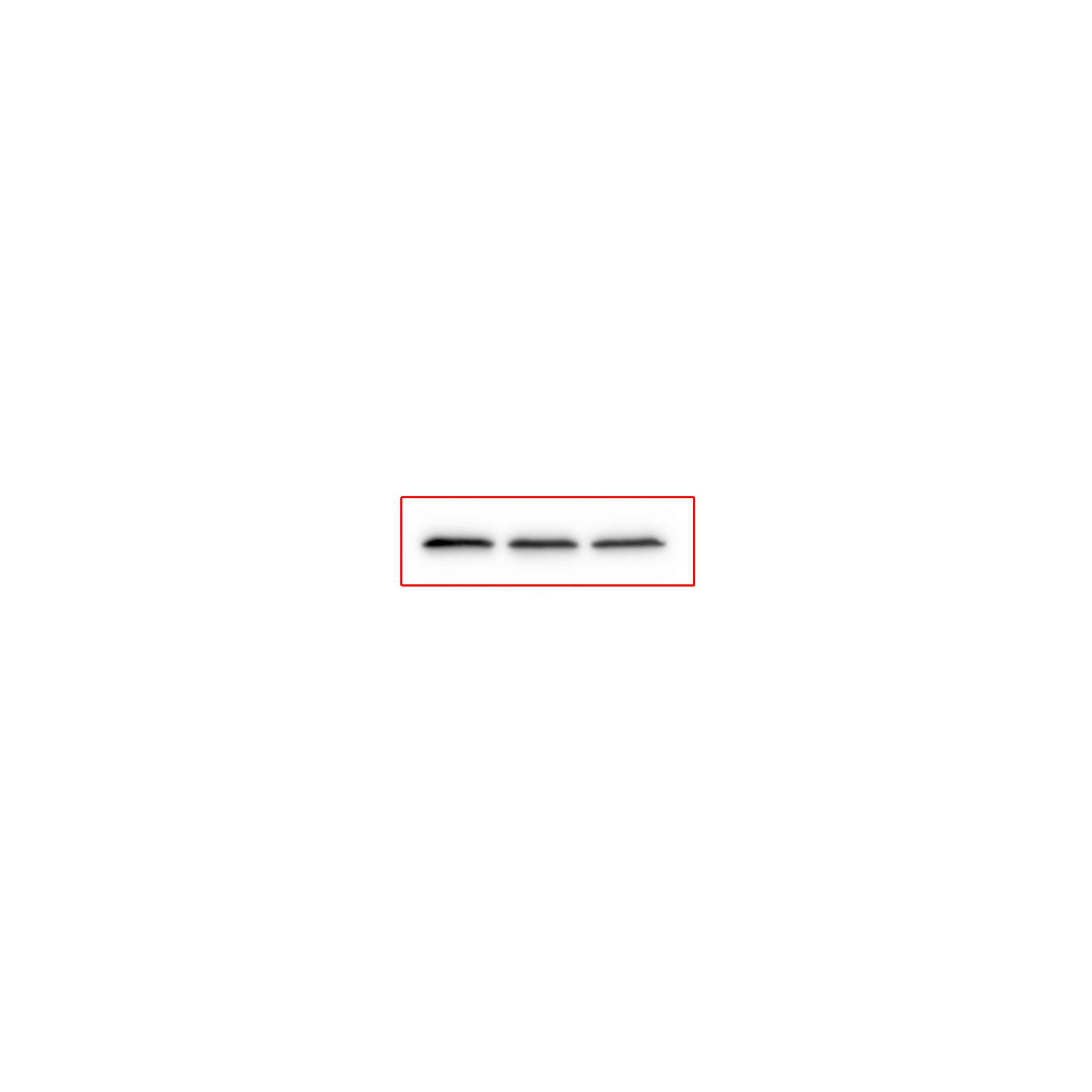

Supplement: Source data 3. [file elife-70151-data3.zip › Source data_v2/Figure 5D/Figure 5D_GAPDH_source data_labelled.jpg]

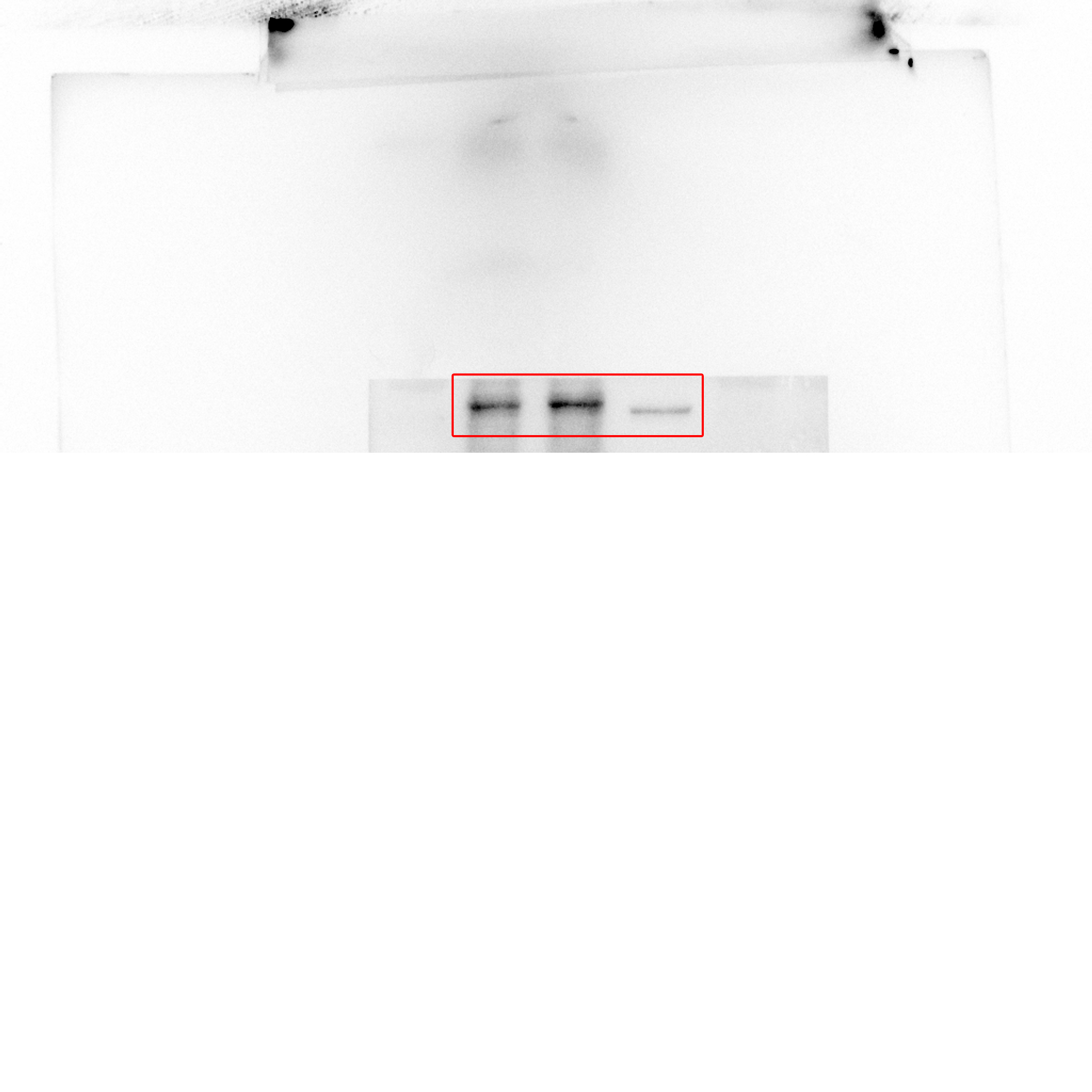

Supplement: Source data 3. [file elife-70151-data3.zip › Source data_v2/Figure 5D/Figure 5D_MMP1_source data_labelled.jpg]

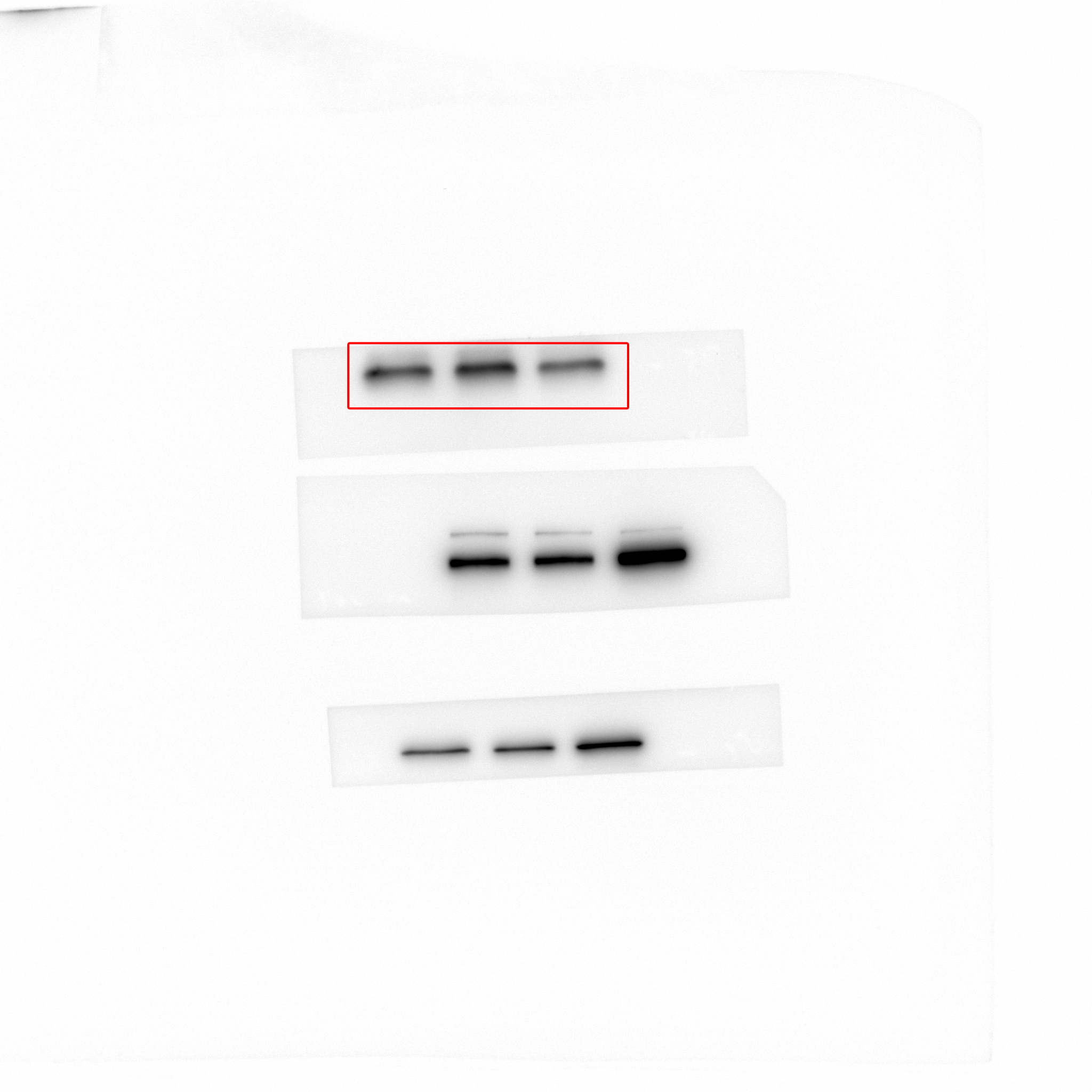

Supplement: Source data 3. [file elife-70151-data3.zip › Source data_v2/Figure 4C/HaCaT/Figure 4C_EpCAM in HaCaT_source data_labelled.jpg]

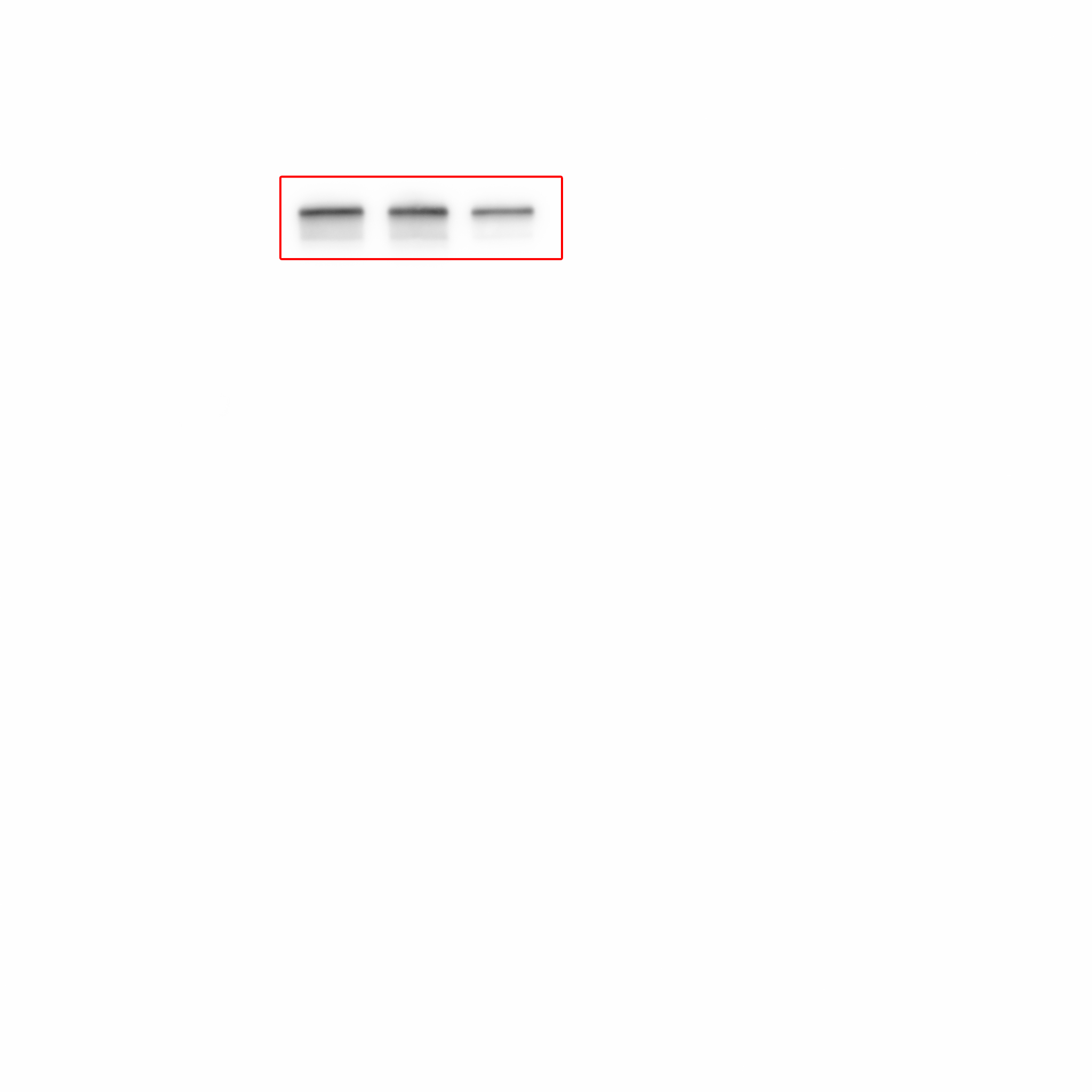

Supplement: Source data 3. [file elife-70151-data3.zip › Source data_v2/Figure 4C/HaCaT/Figure 4C_E-cadherin in HaCaT_source data_labelled.jpg]

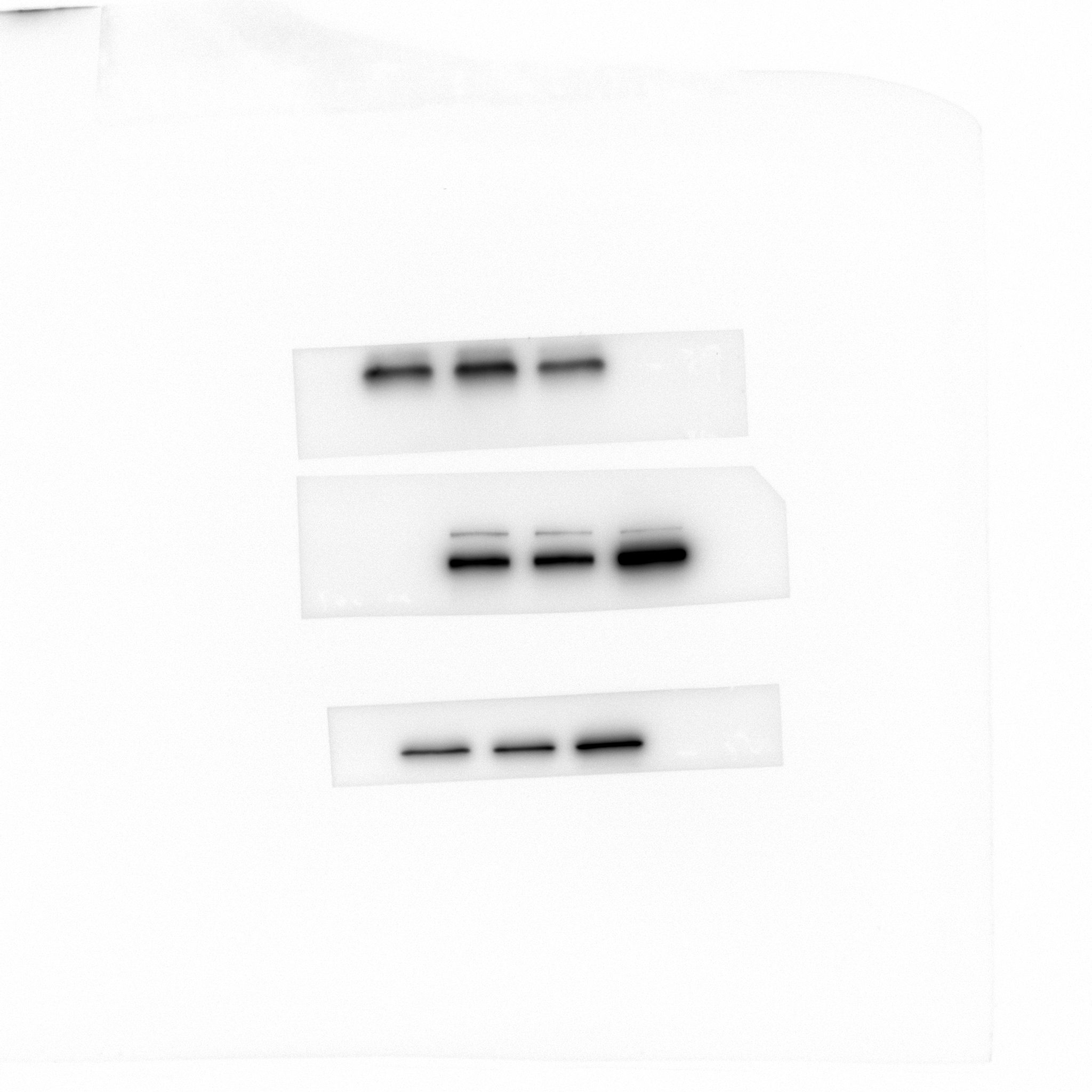

Supplement: Source data 3. [file elife-70151-data3.zip › Source data_v2/Figure 4C/HaCaT/Figure 4C_Vimentin in HaCaT_source data.jpg]

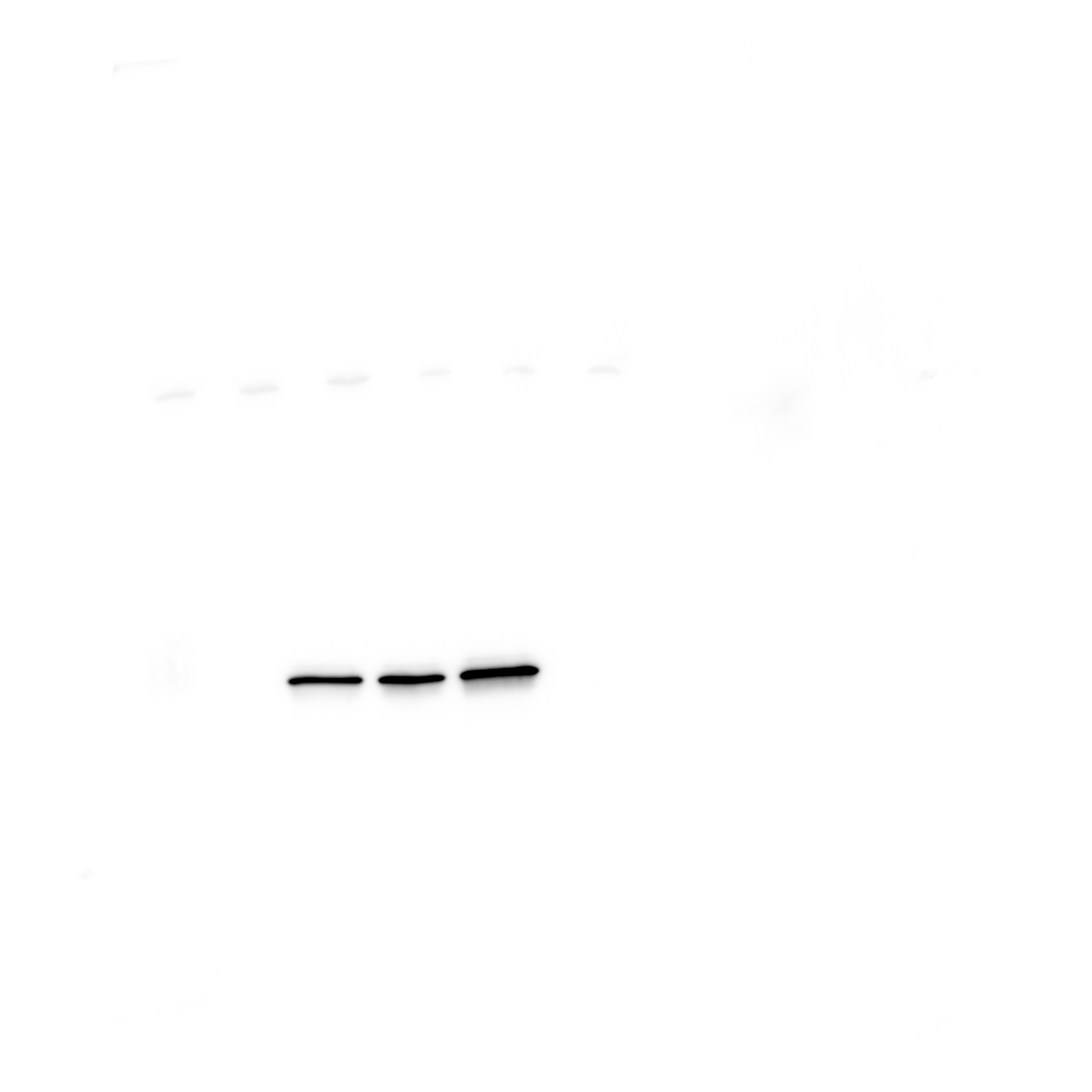

Supplement: Source data 3. [file elife-70151-data3.zip › Source data_v2/Figure 4C/HaCaT/Figure 4C_GAPDH in HaCaT_source data.jpg]

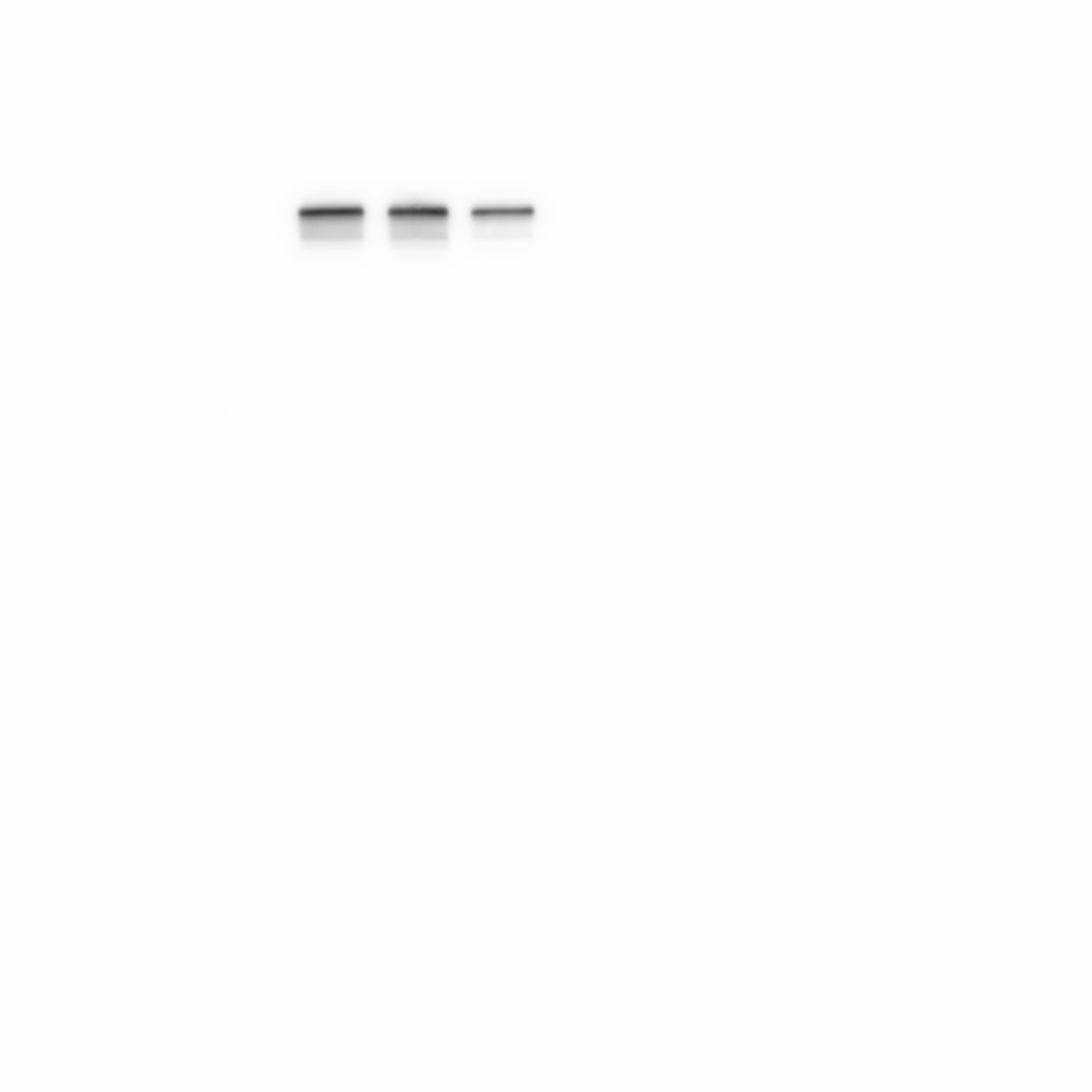

Supplement: Source data 3. [file elife-70151-data3.zip › Source data_v2/Figure 4C/HaCaT/Figure 4C_E-cadherin in HaCaT_source data.jpg]

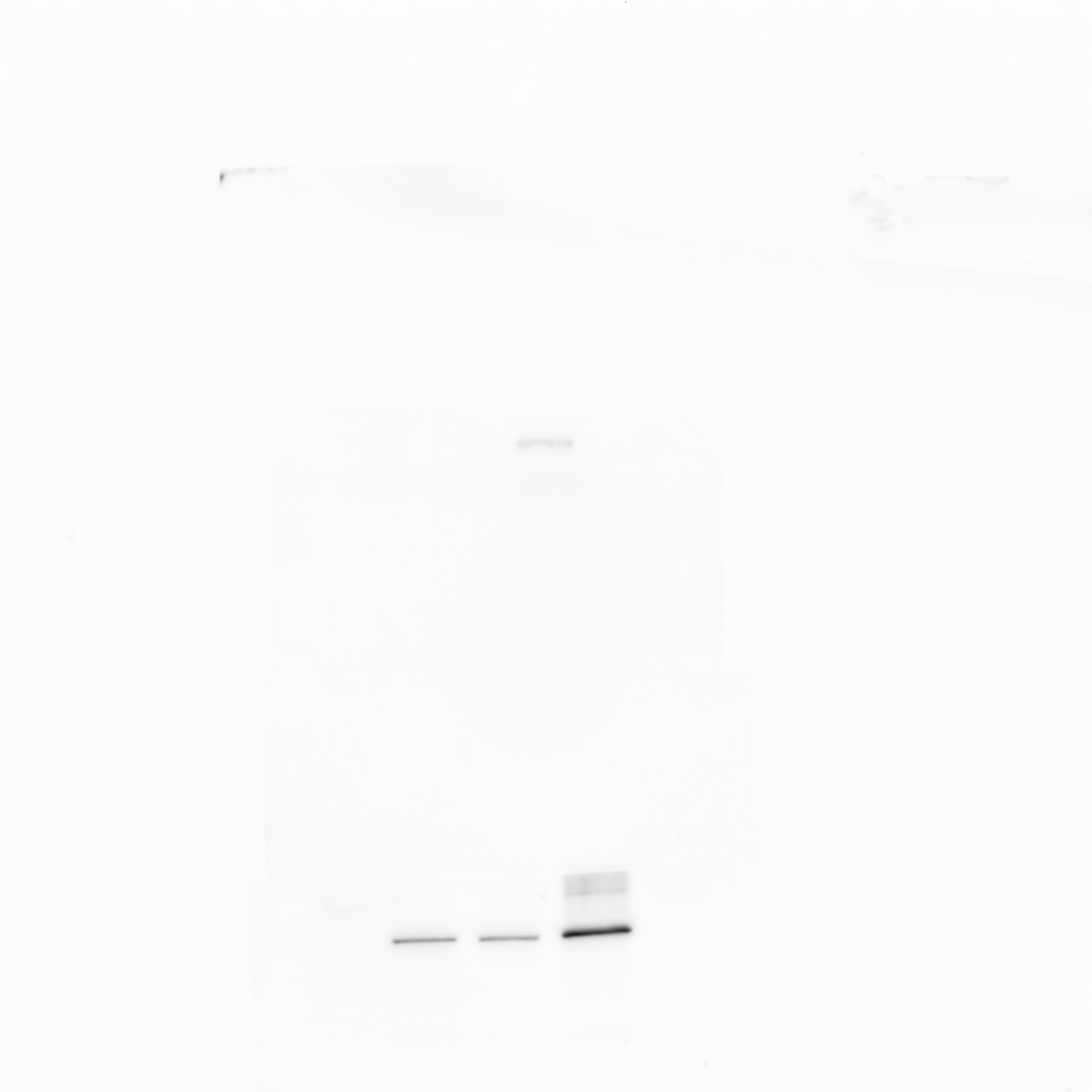

Supplement: Source data 3. [file elife-70151-data3.zip › Source data_v2/Figure 4C/HaCaT/Figure 4C_HTR2C in HaCaT_source data.jpg]

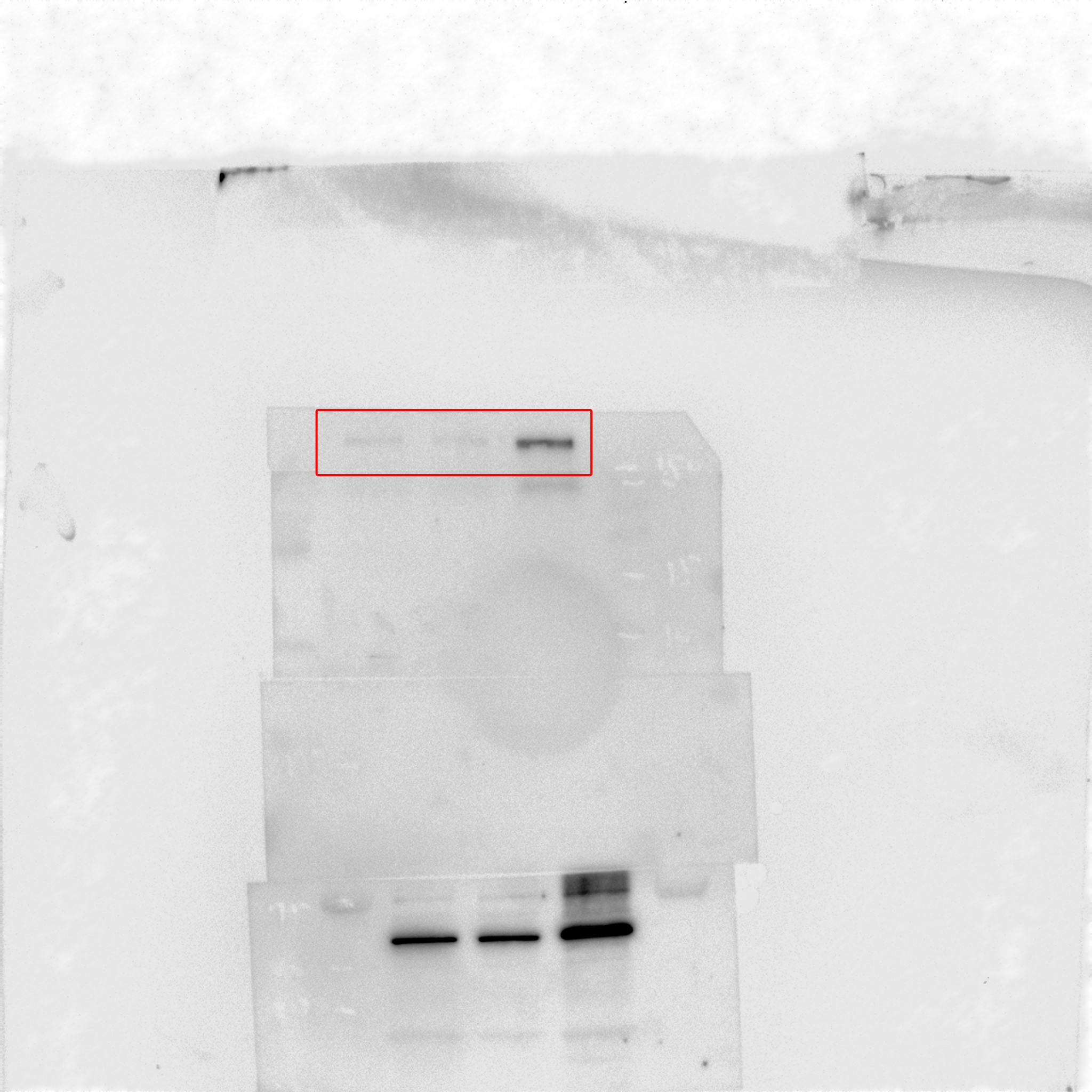

Supplement: Source data 3. [file elife-70151-data3.zip › Source data_v2/Figure 4C/HaCaT/Figure 4C_Zeb1_HaCaT_source data_labelled.jpg]

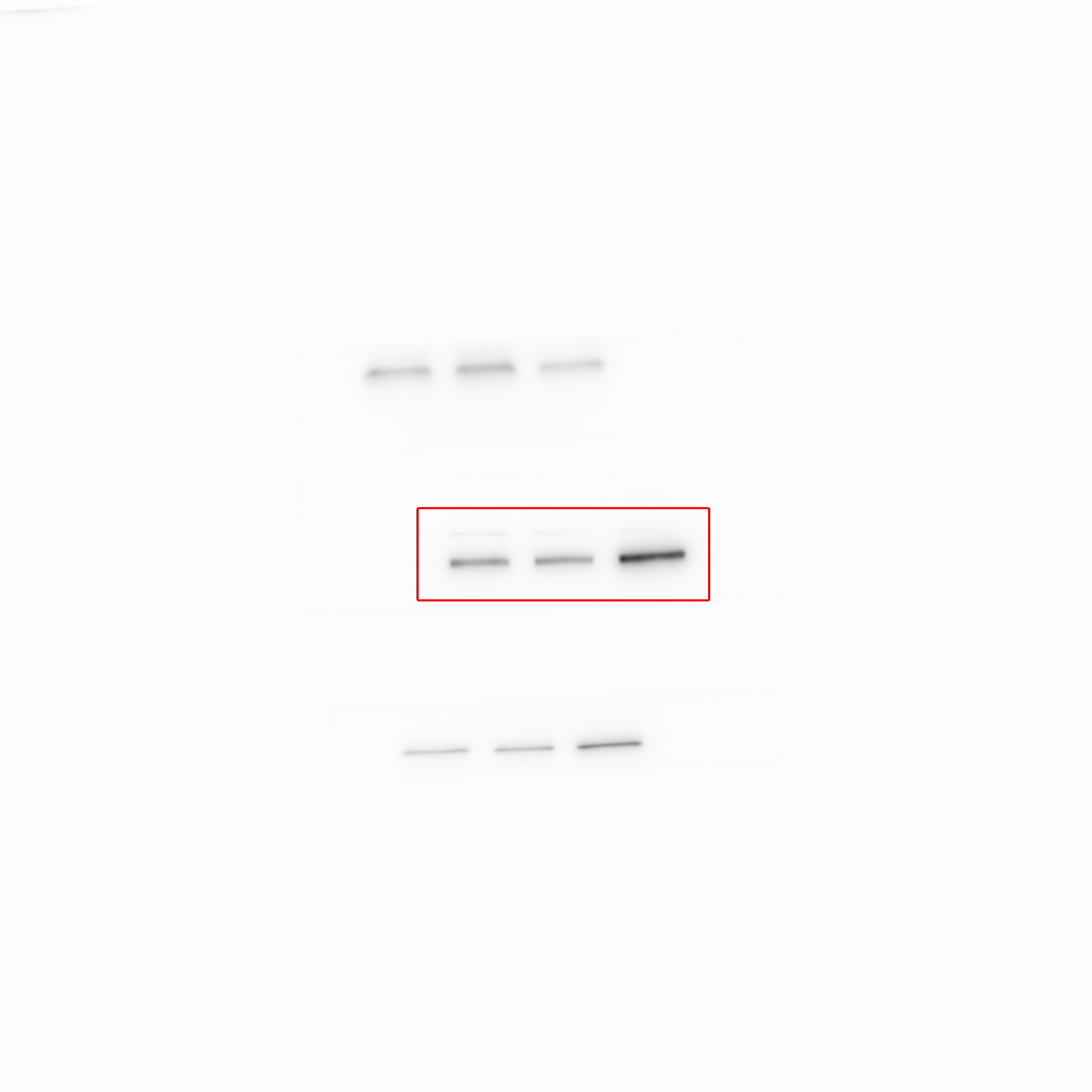

Supplement: Source data 3. [file elife-70151-data3.zip › Source data_v2/Figure 4C/HaCaT/Figure 4C_N-cadherin in HaCaT_source data_labelled.jpg]

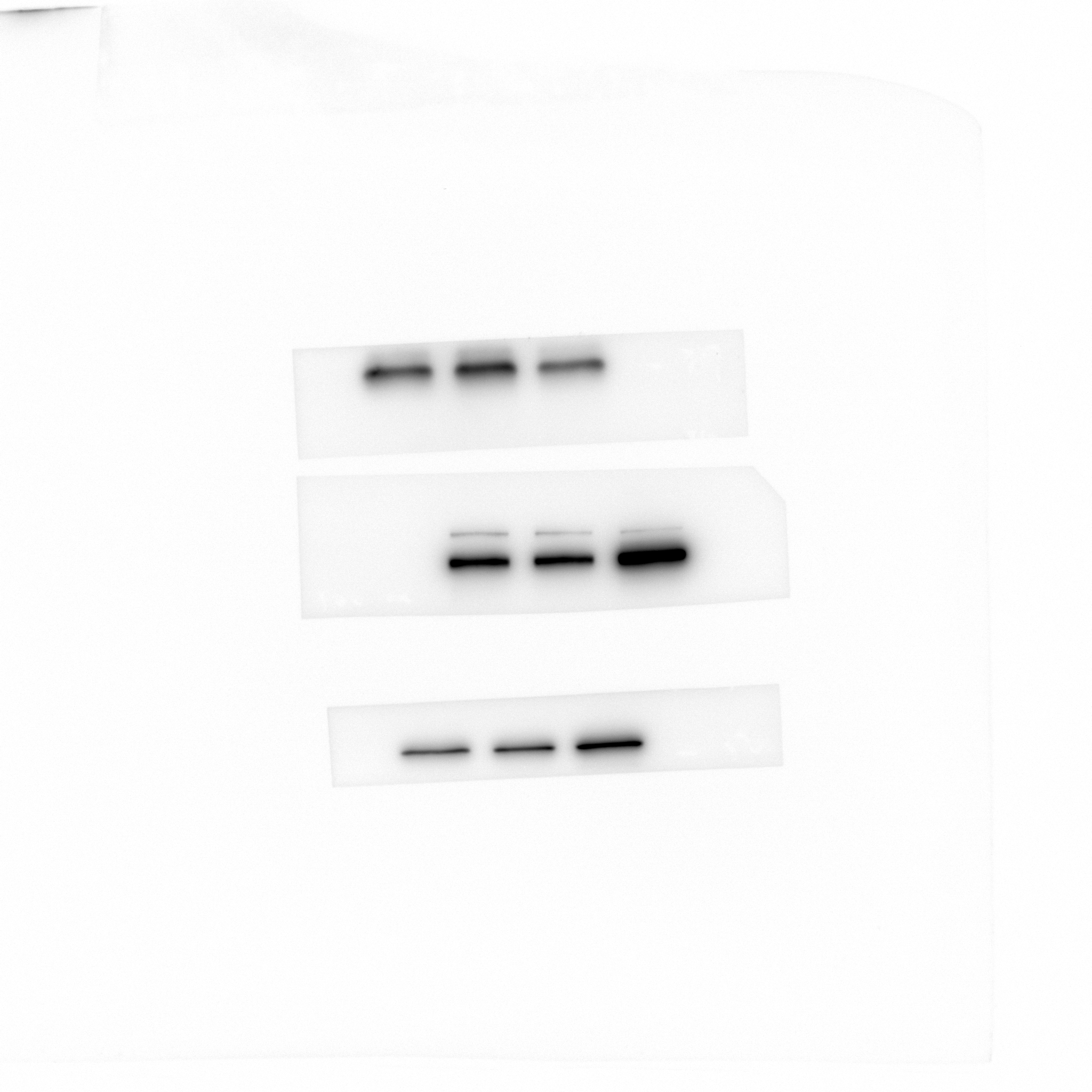

Supplement: Source data 3. [file elife-70151-data3.zip › Source data_v2/Figure 4C/HaCaT/Figure 4C_EpCAM in HaCaT_source data.jpg]

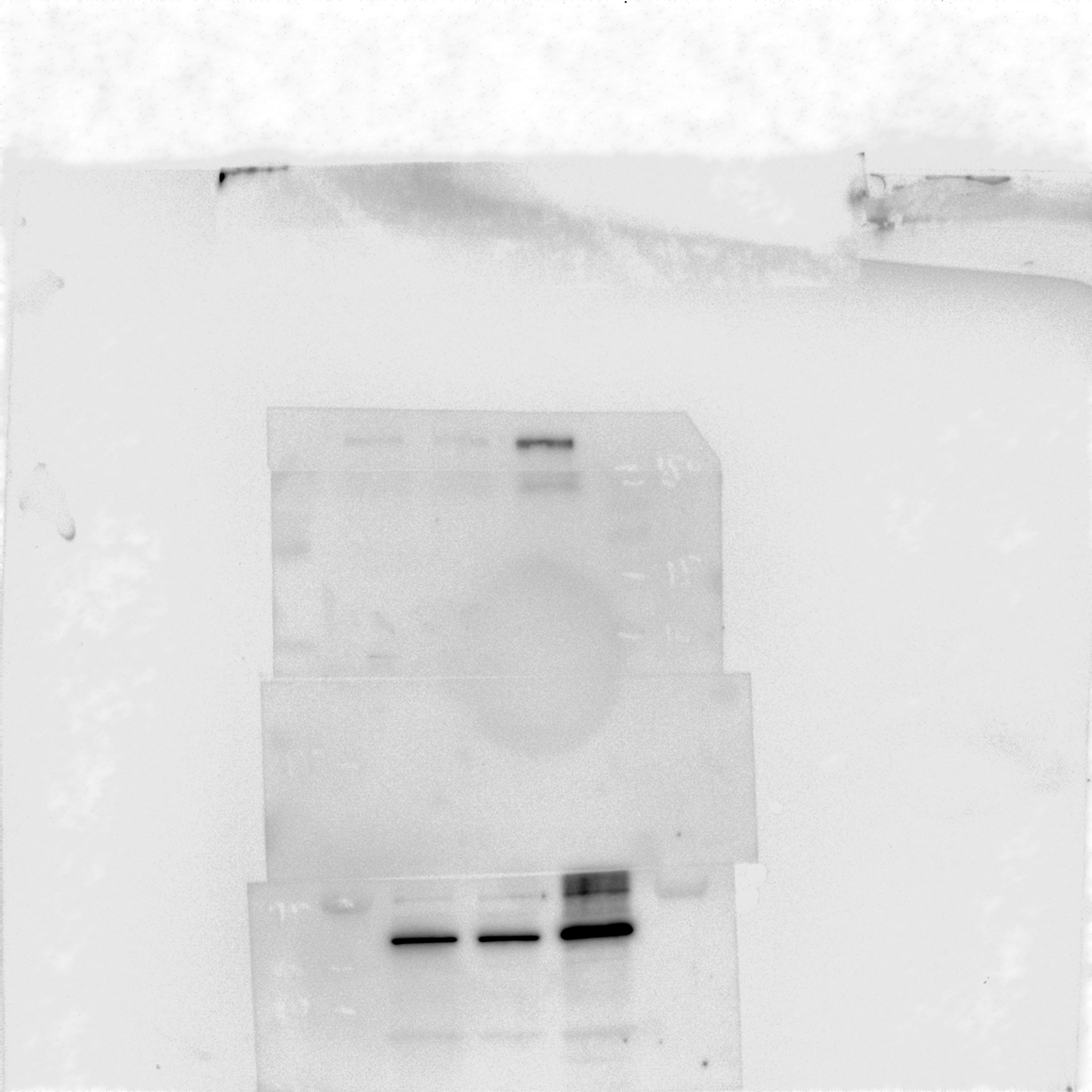

Supplement: Source data 3. [file elife-70151-data3.zip › Source data_v2/Figure 4C/HaCaT/Figure 4C_Zeb1_HaCaT_source data.jpg]

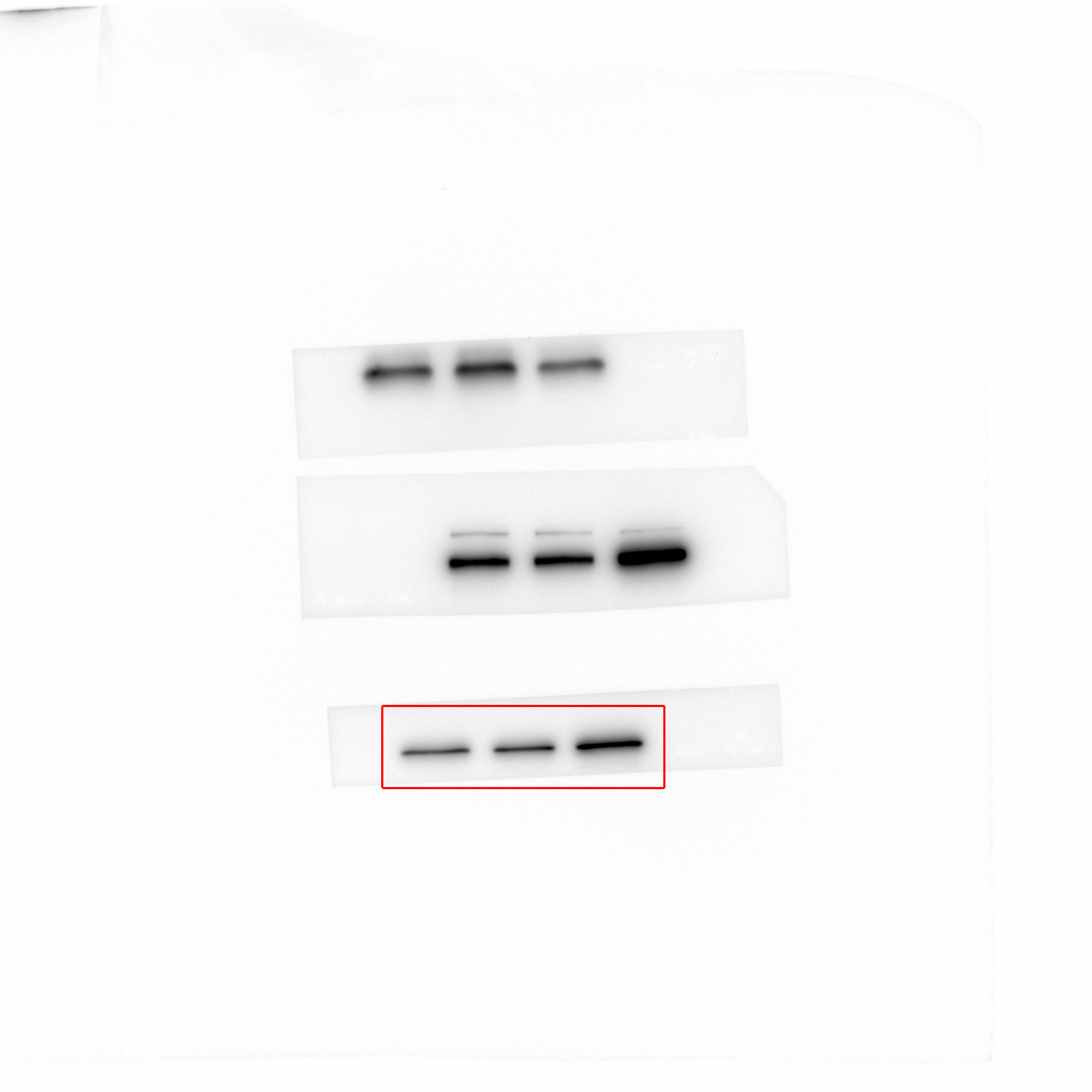

Supplement: Source data 3. [file elife-70151-data3.zip › Source data_v2/Figure 4C/HaCaT/Figure 4C_Vimentin in HaCaT_source data_labelled.jpg]

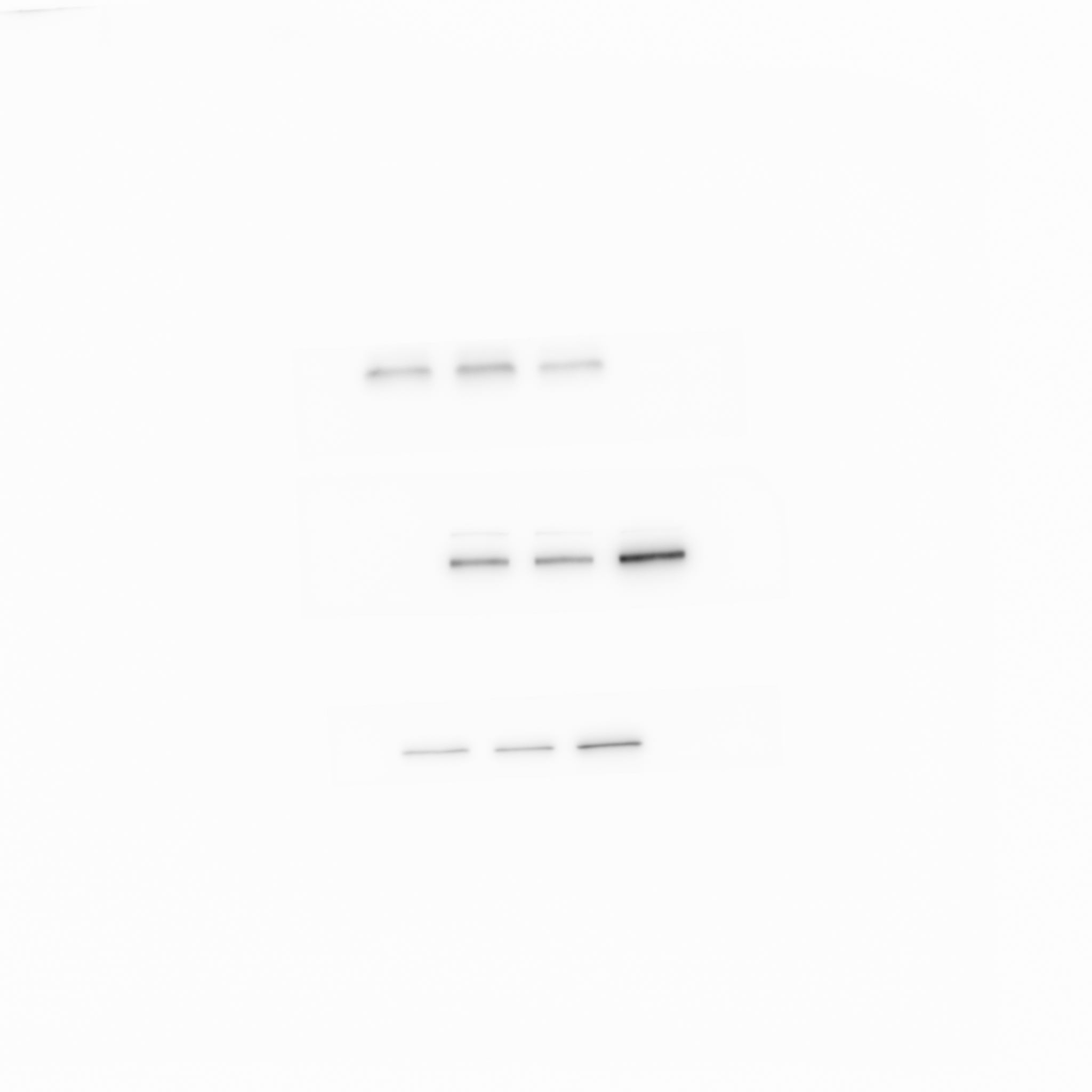

Supplement: Source data 3. [file elife-70151-data3.zip › Source data_v2/Figure 4C/HaCaT/Figure 4C_N-cadherin in HaCaT_source data.jpg]

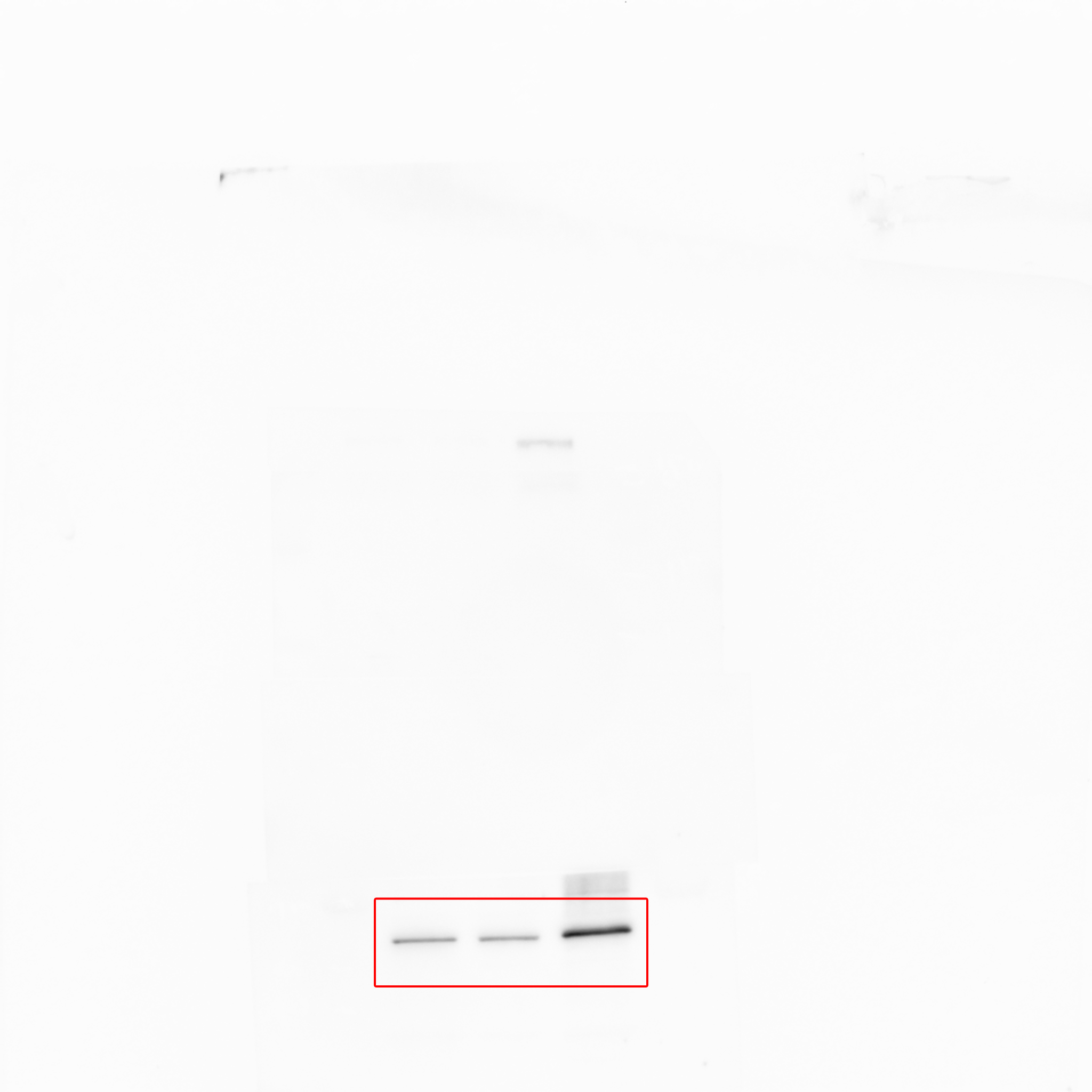

Supplement: Source data 3. [file elife-70151-data3.zip › Source data_v2/Figure 4C/HaCaT/Figure 4C_HTR2C in HaCaT_source data_labelled.jpg]

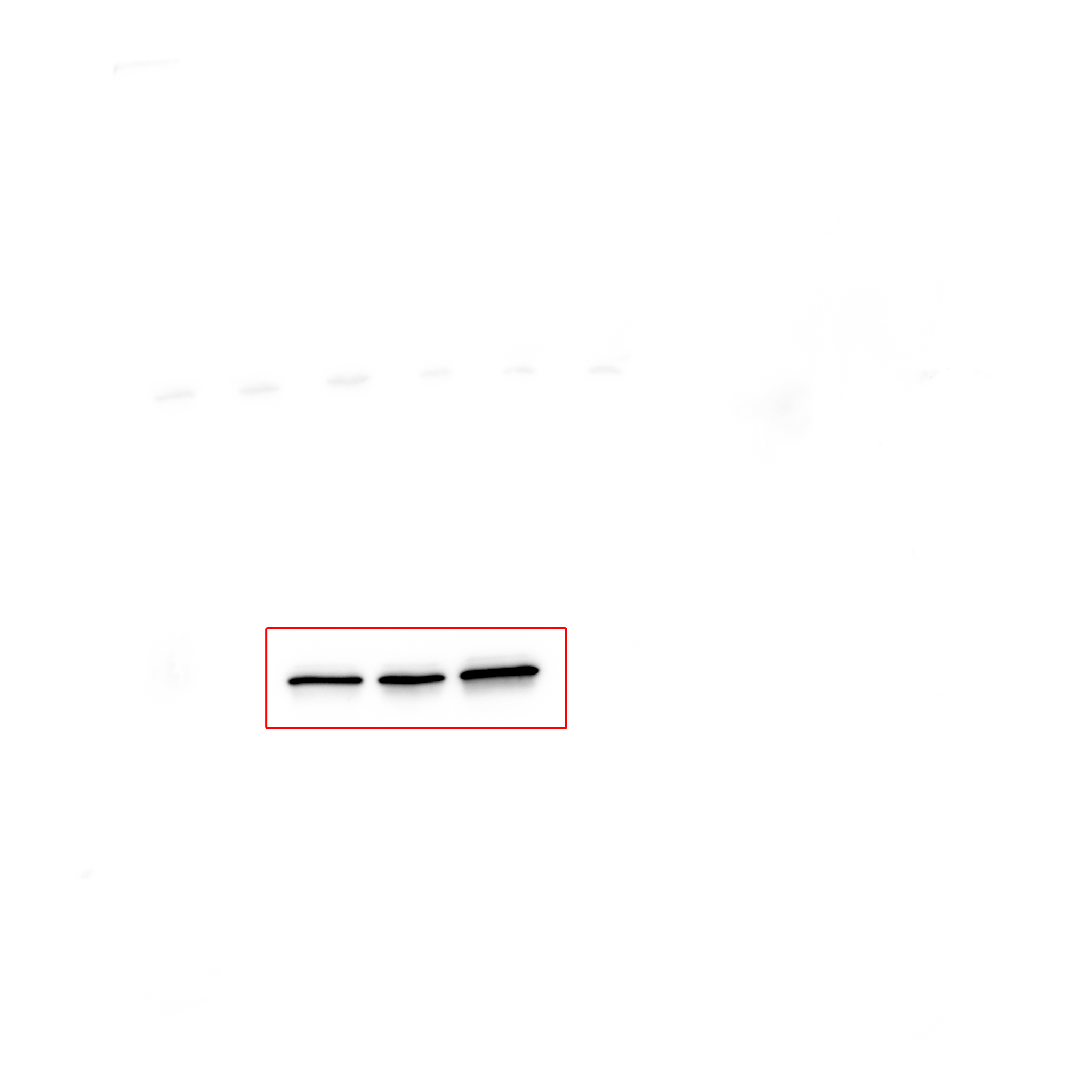

Supplement: Source data 3. [file elife-70151-data3.zip › Source data_v2/Figure 4C/HaCaT/Figure 4C_GAPDH in HaCaT_source data_labelled.jpg]

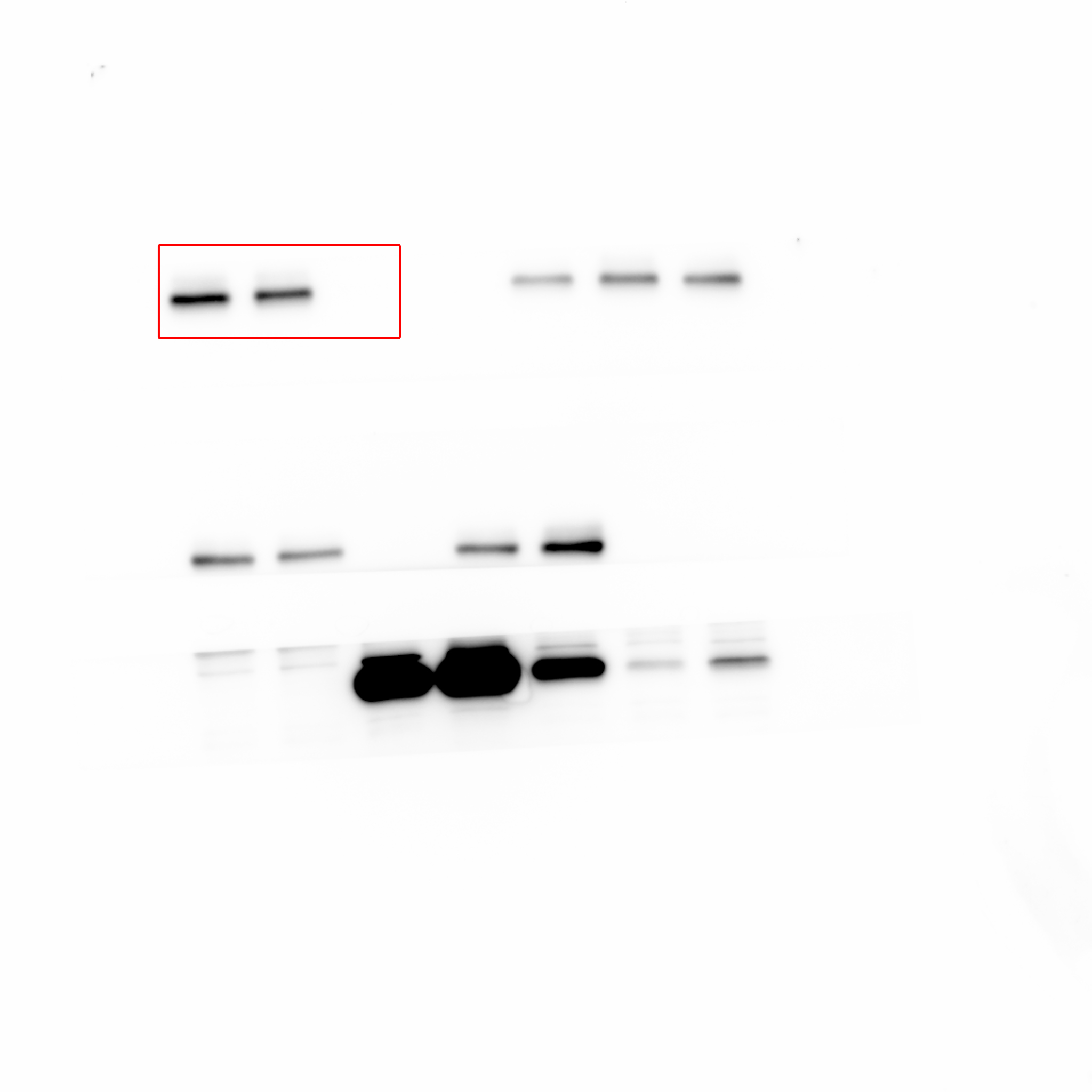

Supplement: Source data 3. [file elife-70151-data3.zip › Source data_v2/Figure 4C/MCF7/Figure 4C_EpCAM in MCF7_source data.labelled.jpg]
