# Supplementary material for: A zebrafish embryo screen utilizing gastrulation identifies the HTR2C inhibitor pizotifen as a suppressor of EMT-mediated metastasis
Source: eLife. 2021 Dec 17;10:e70151. doi: 10.7554/eLife.70151 (PMC8824480; doi:10.7554/eLife.70151)
Supplement: Source data 4. [file elife-70151-data4.docx]

**Source data and** **legend for source data**

**Figure 1-figure supplement 2A_PRMT1_source data_source data**

**
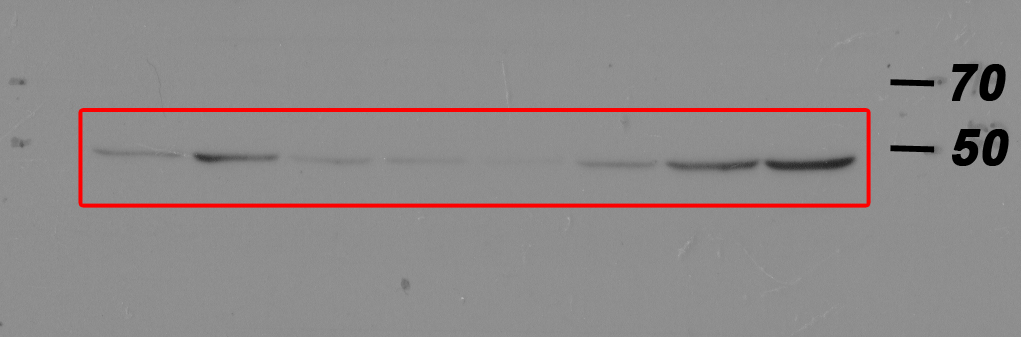

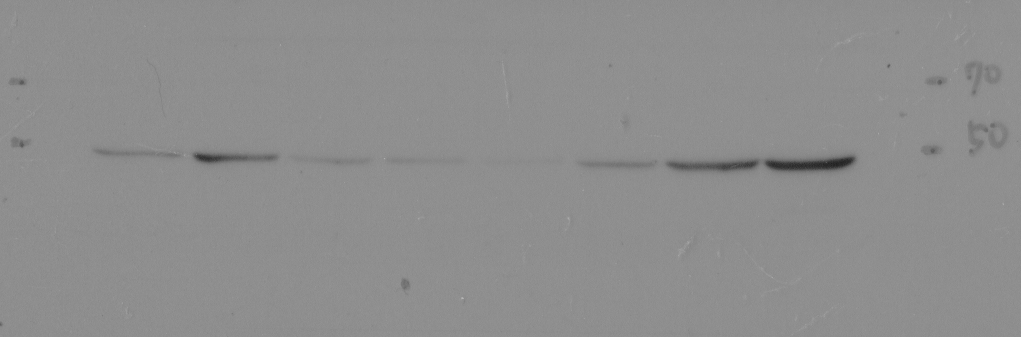
**

Western blot analysis of PRMT1 protein levels in non-metastatic human cancer cell line (MCF7) and highly metastatic human cancer cell lines (MDA-MB-231, MDA-MB-435, MIA-PaCa2, PC9, HCCLM3, SW620 and PC3).

**Figure 1-figure supplement 2A_CYP11A1_source data**

**
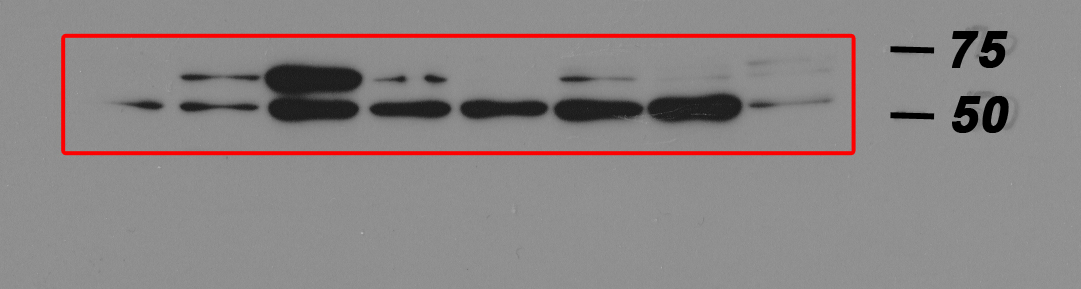

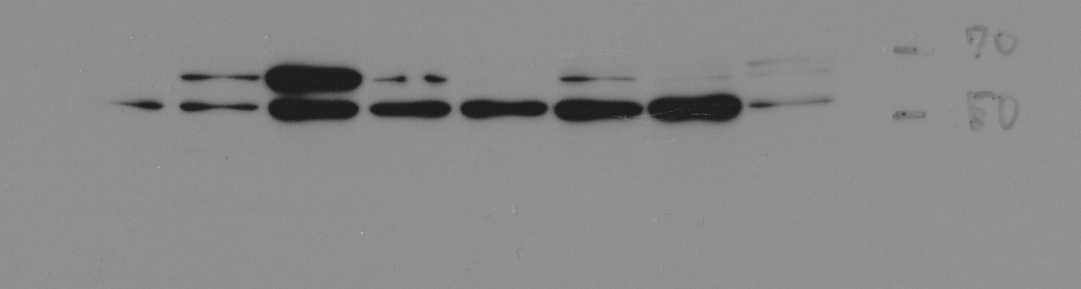
**

Western blot analysis of CYP11A1 protein levels in non-metastatic human cancer cell line (MCF7) and highly metastatic human cancer cell lines (MDA-MB-231, MDA-MB-435, MIA-PaCa2, PC9, HCCLM3, SW620 and PC3).

**Figure 1-figure supplement 2A_β-actin_source data**

**
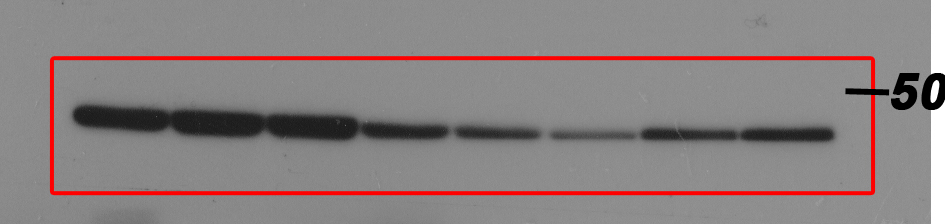

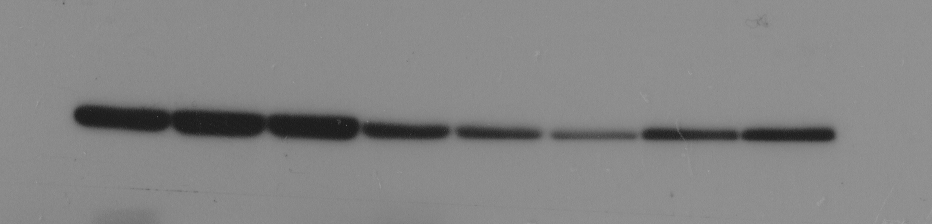
**

Western blot analysis of β-actin protein levels in non-metastatic human cancer cell line (MCF7) and highly metastatic human cancer cell lines (MDA-MB-231, MDA-MB-435, MIA-PaCa2, PC9, HCCLM3, SW620 and PC3).

**Figure 1-figure supplement 2A _PRMT1_source data**

**
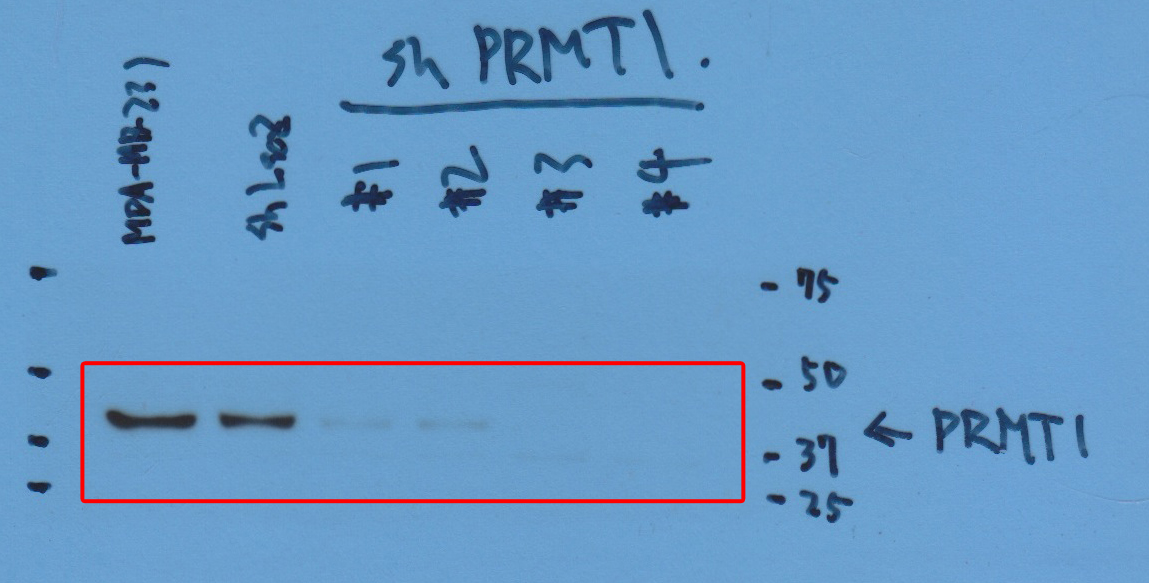

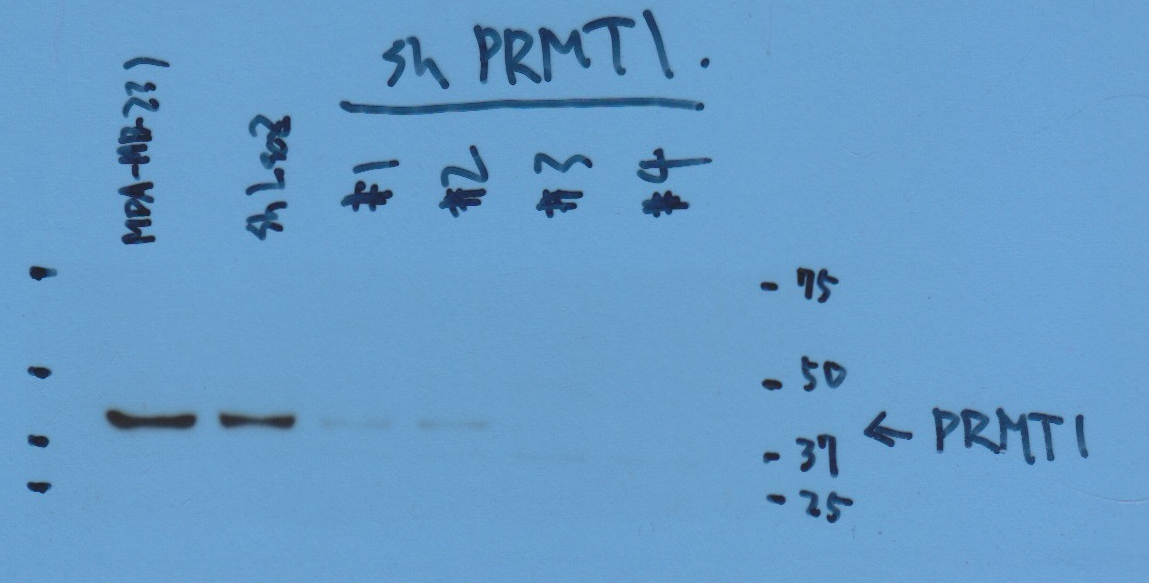
**

Western blot analysis of PRMT1 protein levels in sub-clones of MBA-MB-231 cells which were transfected with either a control shRNA targeting LacZ or one of four independent shRNAs targeting PRMT1 (clone #1 to #4).

**Figure 1-figure supplement 2A_β-actin _source data**

**
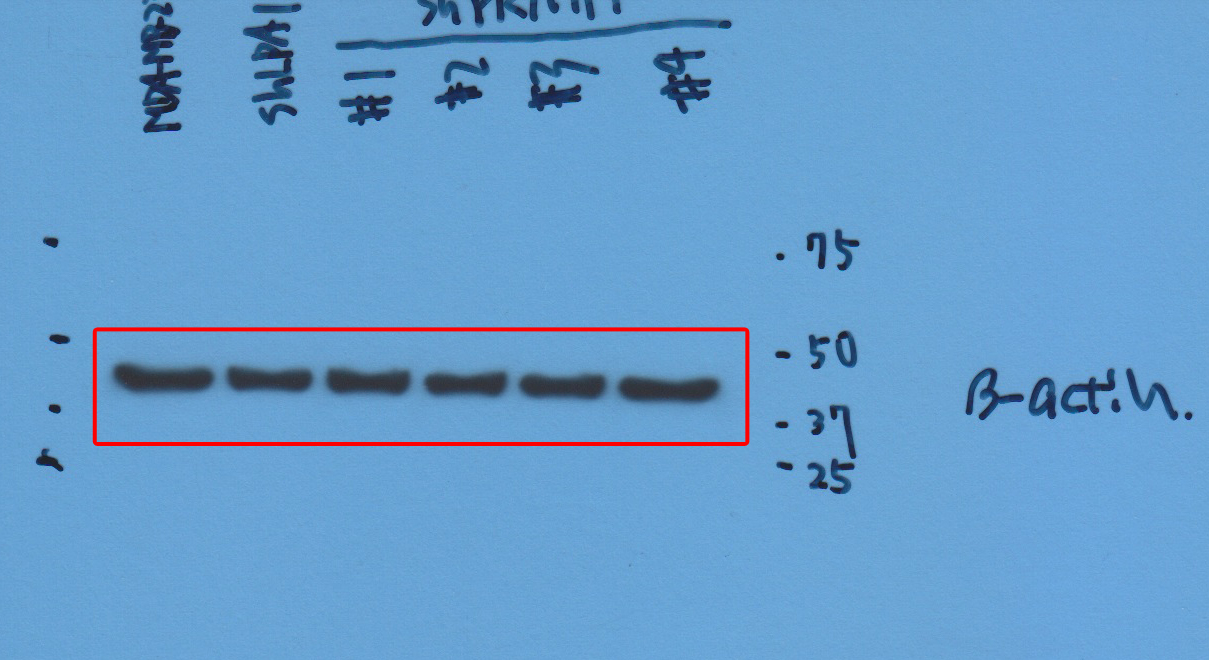

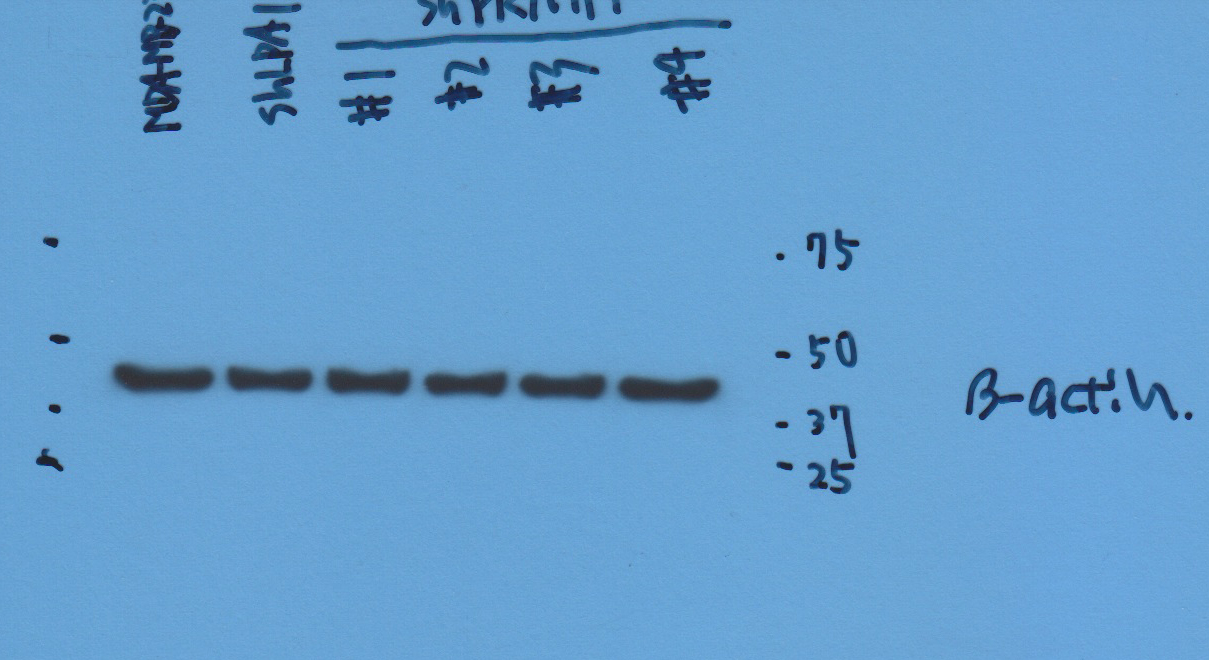
**

Western blot analysis of β-actin protein levels in sub-clones of MBA-MB-231 cells which were transfected with either a control shRNA targeting LacZ or one of four independent shRNAs targeting PRMT1 (clone #1 to #4).

**Figure 1-figure supplement 2A_CYP11A1_source data**

**
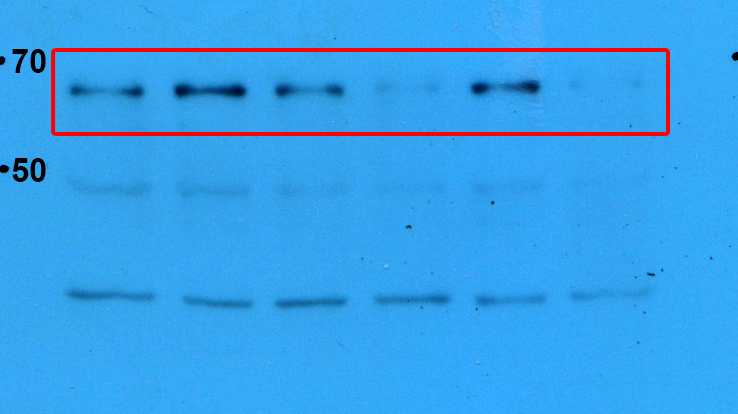

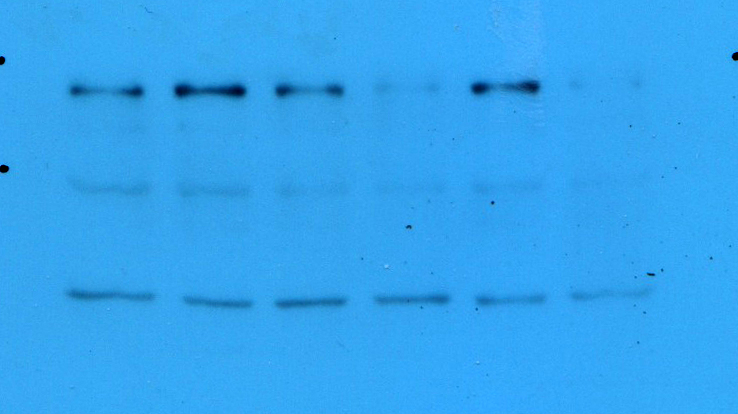
**

Western blot analysis of CYP11A1 protein levels in sub-clones of MBA-MB-231 cells which were transfected with either a control shRNA targeting LacZ or one of four independent shRNAs targeting CYP11A1 (clone #1 to #4).

**Figure 1-figure supplement 2A_ β-actin_source data**

**
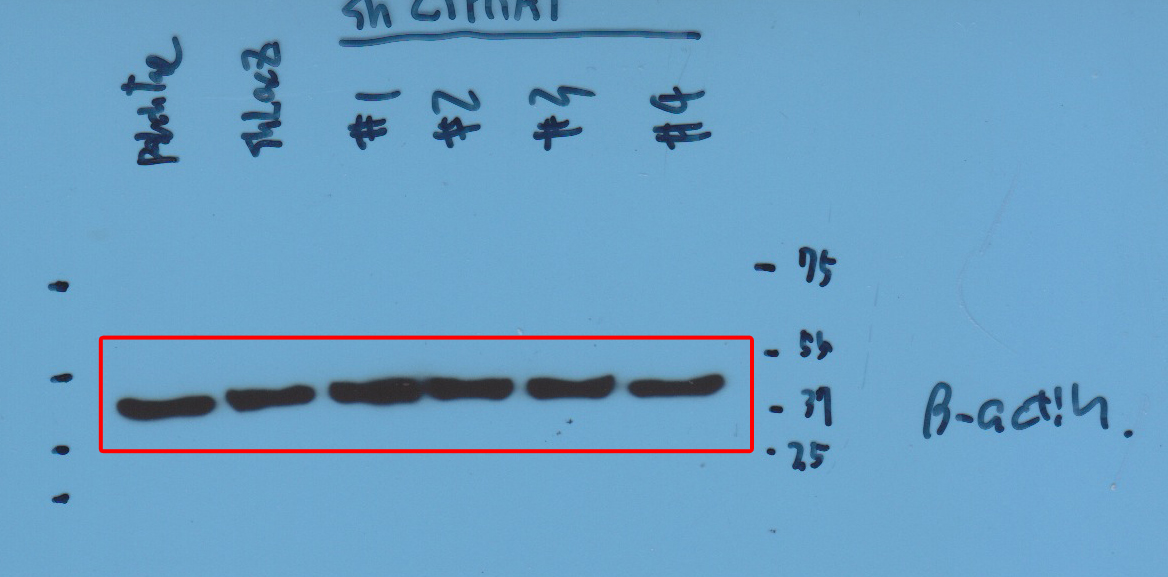

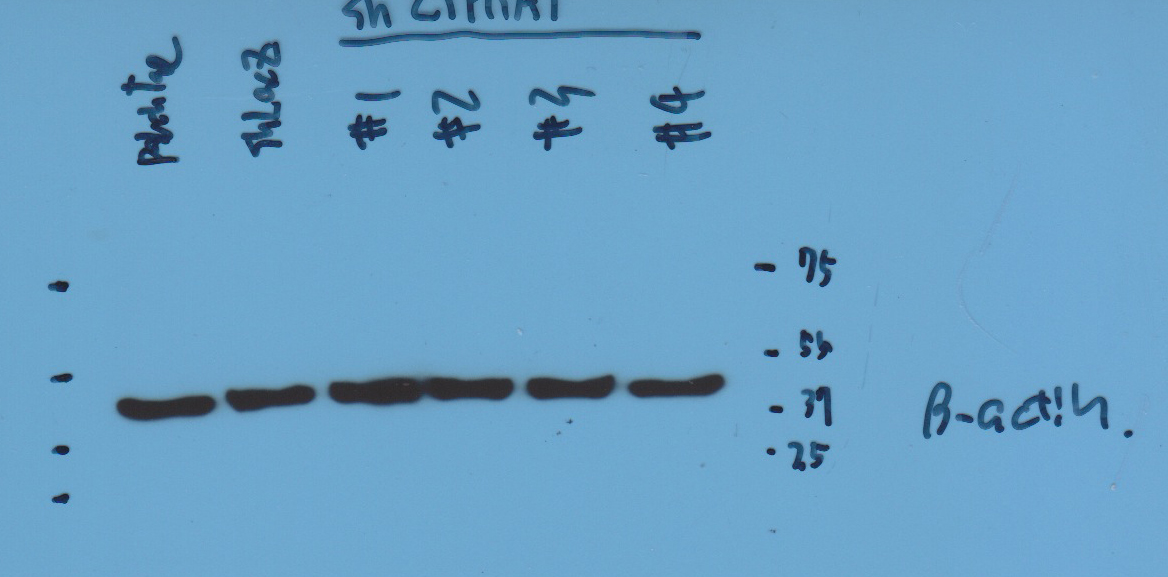
**

Western blot analysis of β-actin protein levels in sub-clones of MBA-MB-231 cells which were transfected with either a control shRNA targeting LacZ or one of four independent shRNAs targeting CYP11A1 (clone #1 to #4).

**Figure 2A_HRT2C_source data**

**
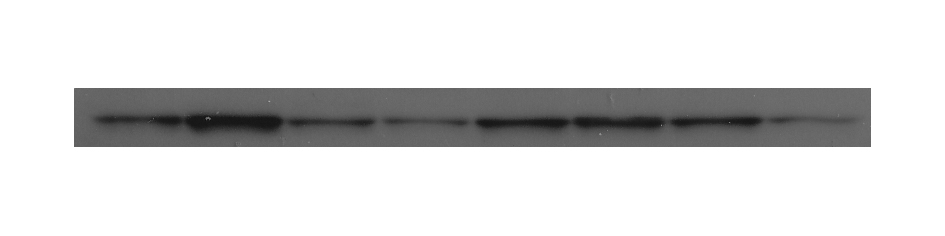

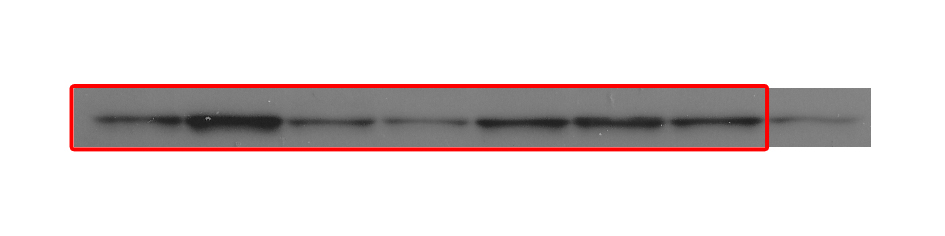
**

Western blot analysis of HRT2C protein levels in non-metastatic human cancer cell line, MCF7 (breast) and highly metastatic human cancer cell lines, MDA-MB-231 (breast), MDA-MB-435 (melanoma), MIA-PaCa2 (pancreas), SW620 (colon) and PC3 (prostate).

**Figure 2A_GAPDH_source data**

**
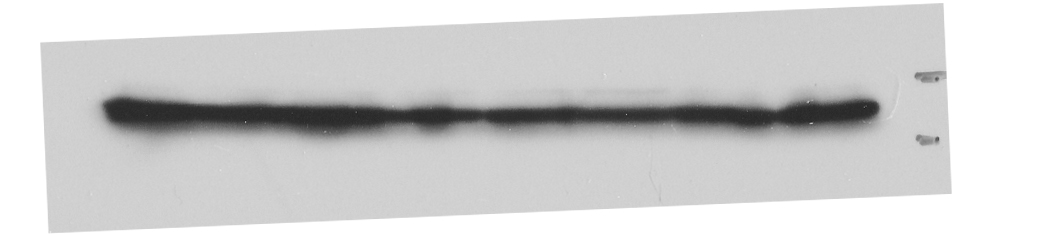

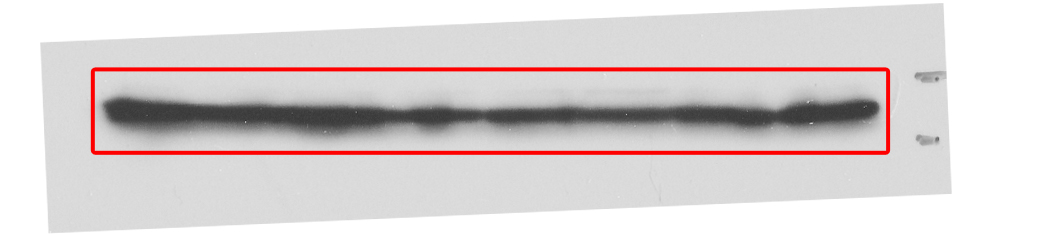
**

Western blot analysis of GAPDH protein levels in non-metastatic human cancer cell line, MCF7 (breast) and highly metastatic human cancer cell lines, MDA-MB-231 (breast), MDA-MB-435 (melanoma), MIA-PaCa2 (pancreas), SW620 (colon) and PC3 (prostate).

**Figure 2-figure supplement 1B_DRD2_source data**

**
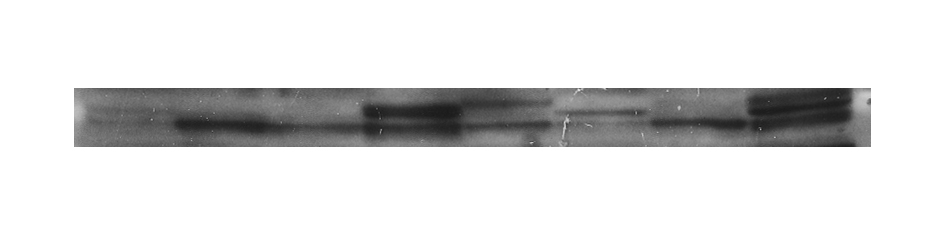

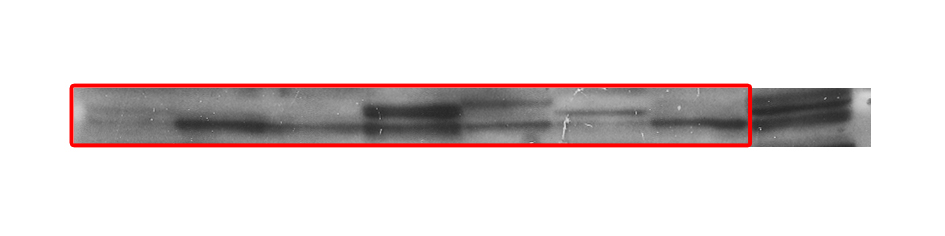
**

Western blot analysis of DRD2 protein levels in non-metastatic human cancer cell line, MCF7 (breast) and highly metastatic human cancer cell lines, MDA-MB-231 (breast), MDA-MB-435 (melanoma), MIA-PaCa2 (pancreas), PC3 (prostate) and SW620 (colon)

**Figure 2-figure supplement 1B_GAPDH_source data**

**
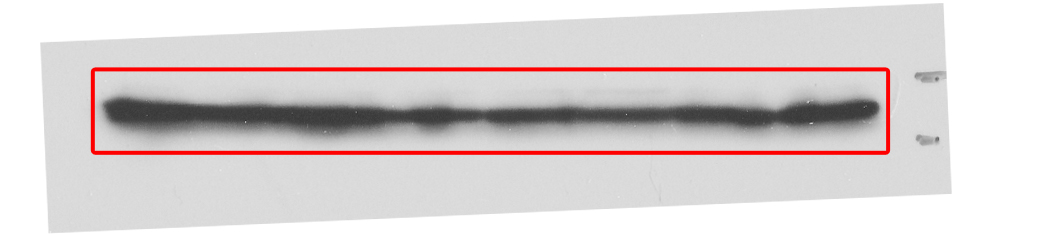

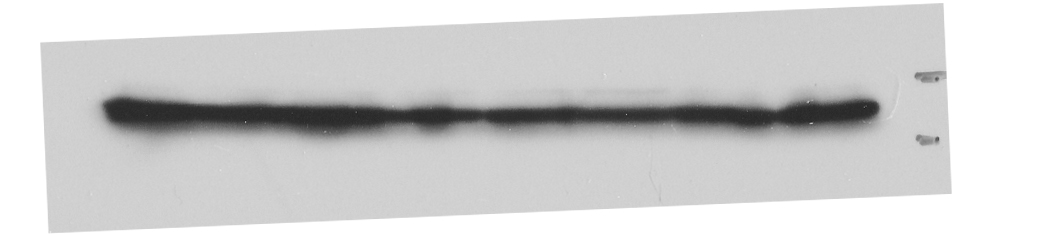
**

Western blot analysis of GAPDH protein levels in non-metastatic human cancer cell line, MCF7 (breast) and highly metastatic human cancer cell lines, MDA-MB-231 (breast), MDA-MB-435 (melanoma), MIA-PaCa2 (pancreas), PC3 (prostate) and SW620 (colon)

**Figure 4C_E-cadherin in MCF7_ source data**

**
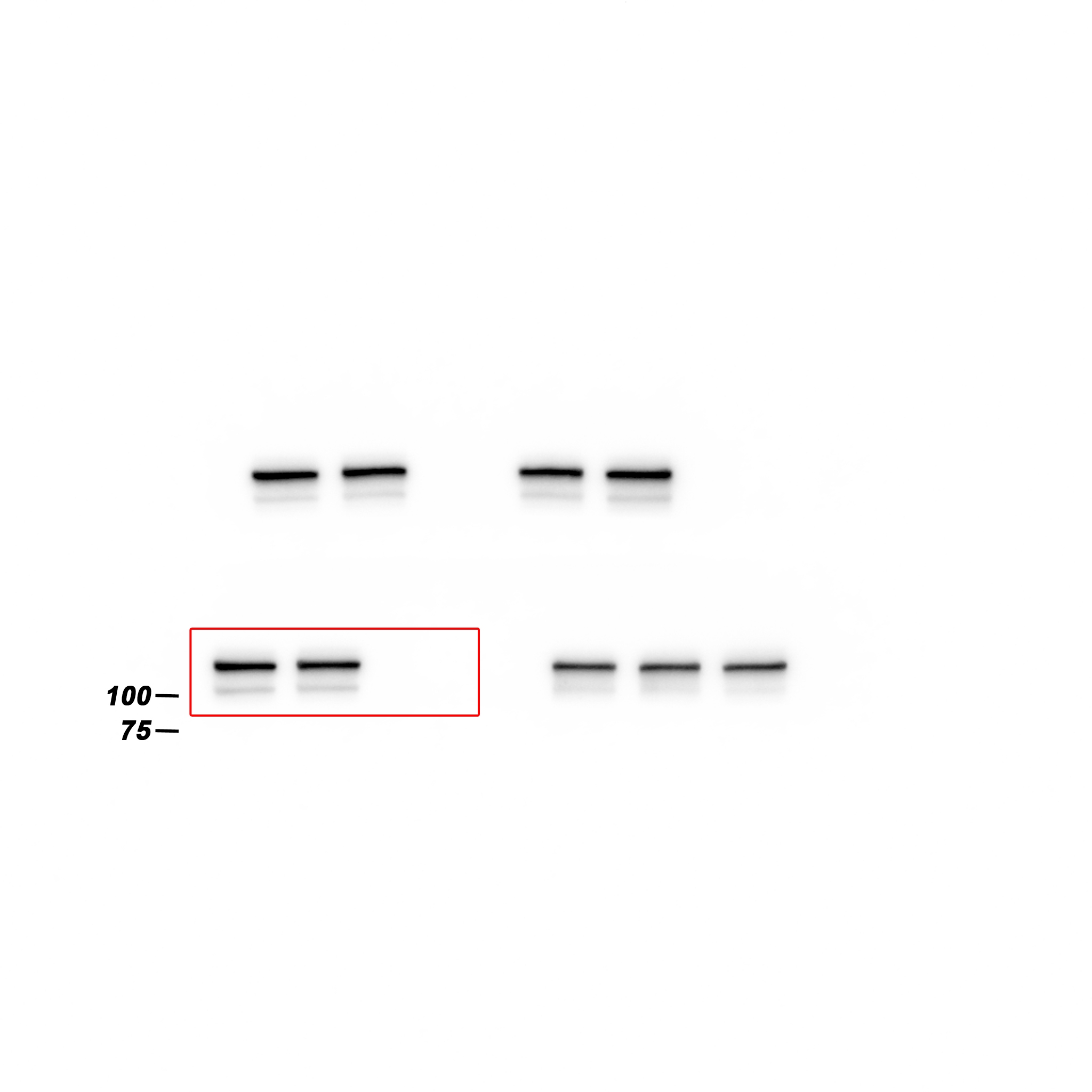

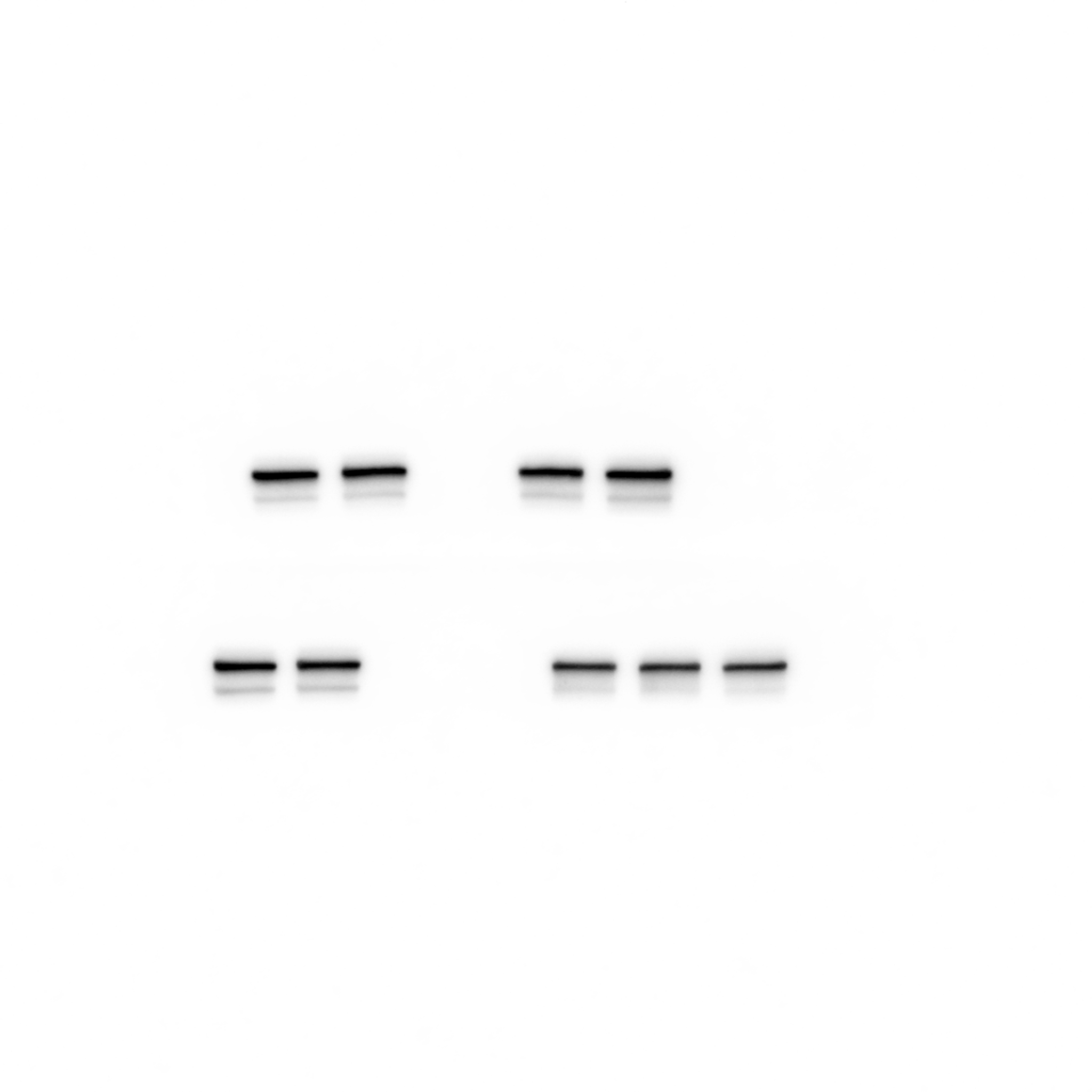
**

Western blot analysis of E-cadherin protein levels in MCF7 cells expressing either the control vector or HTR2C.

**Figure 4C_EpCAM in MCF7_ source data**

**
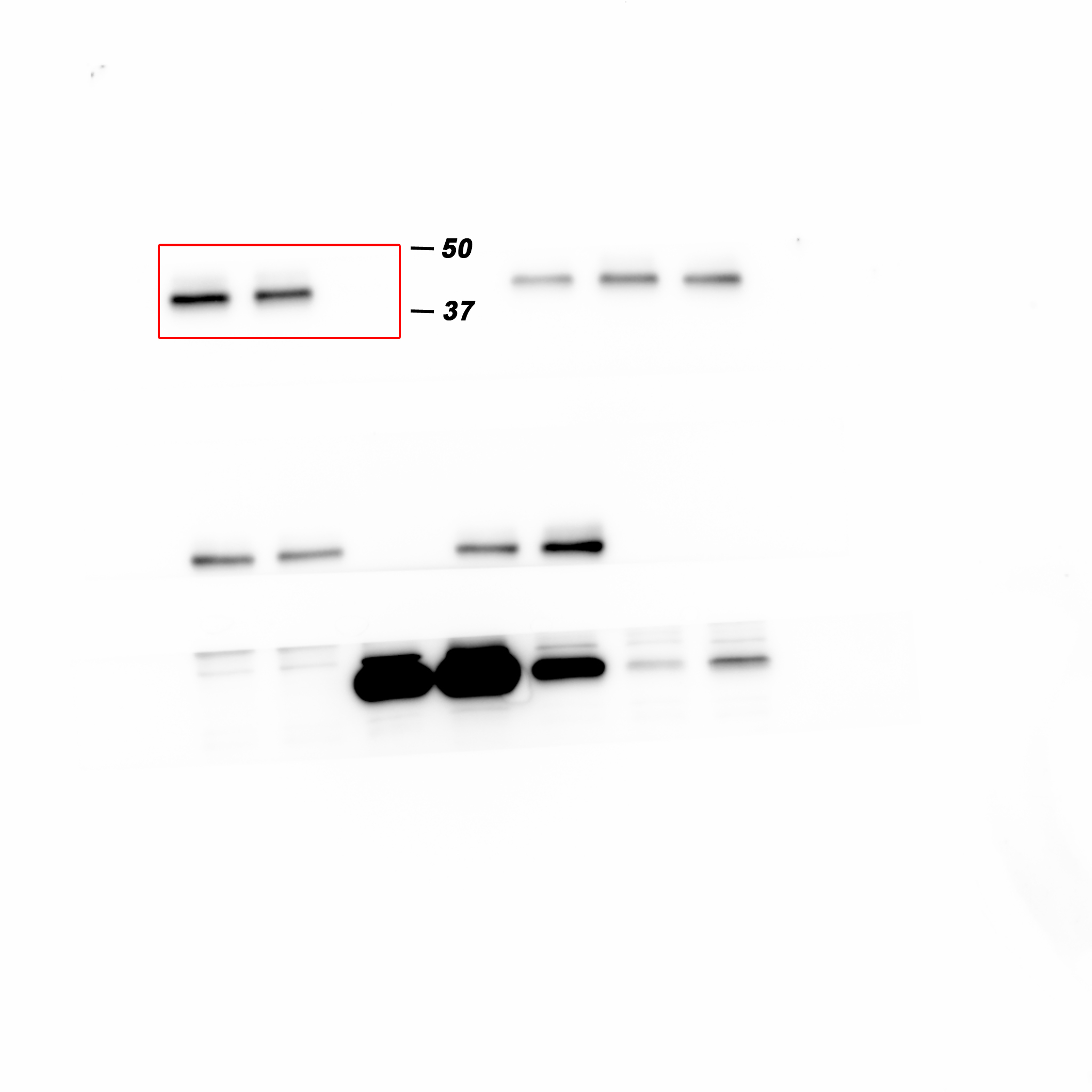

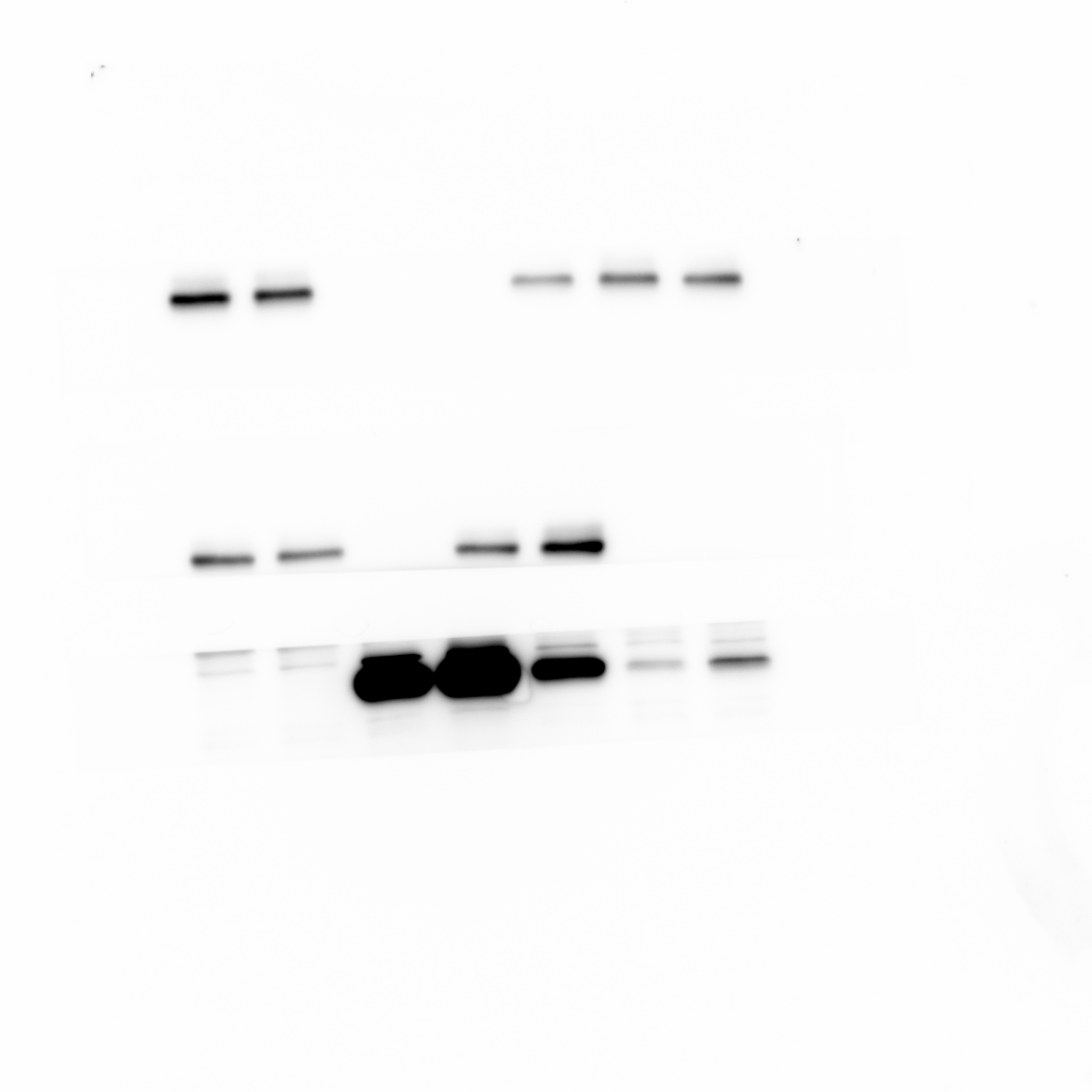
**

Western blot analysis of EpCAM protein levels in MCF7 cells expressing either the control vector or HTR2C.

**Figure 4C_Vimentin in MCF7_ source data**

**
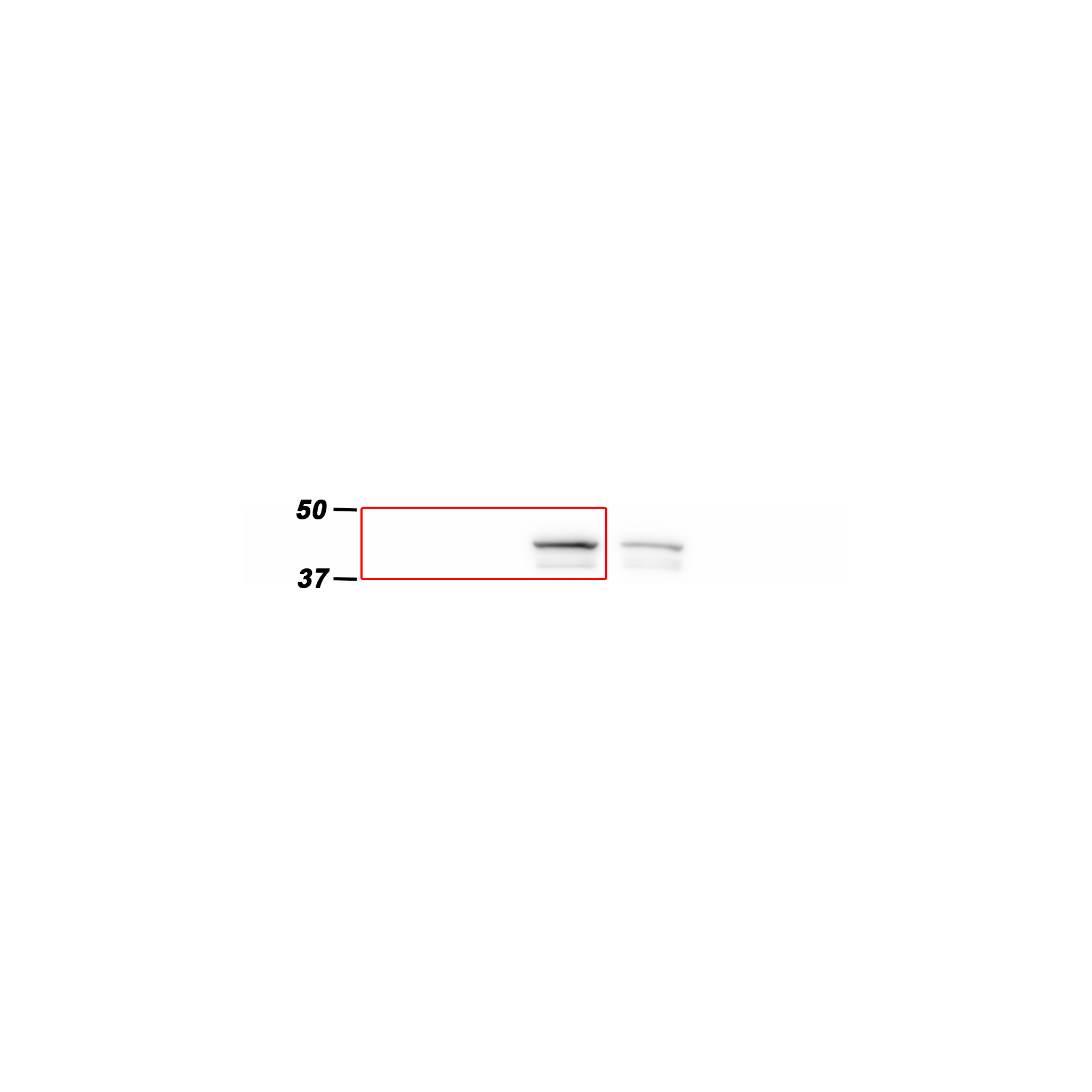

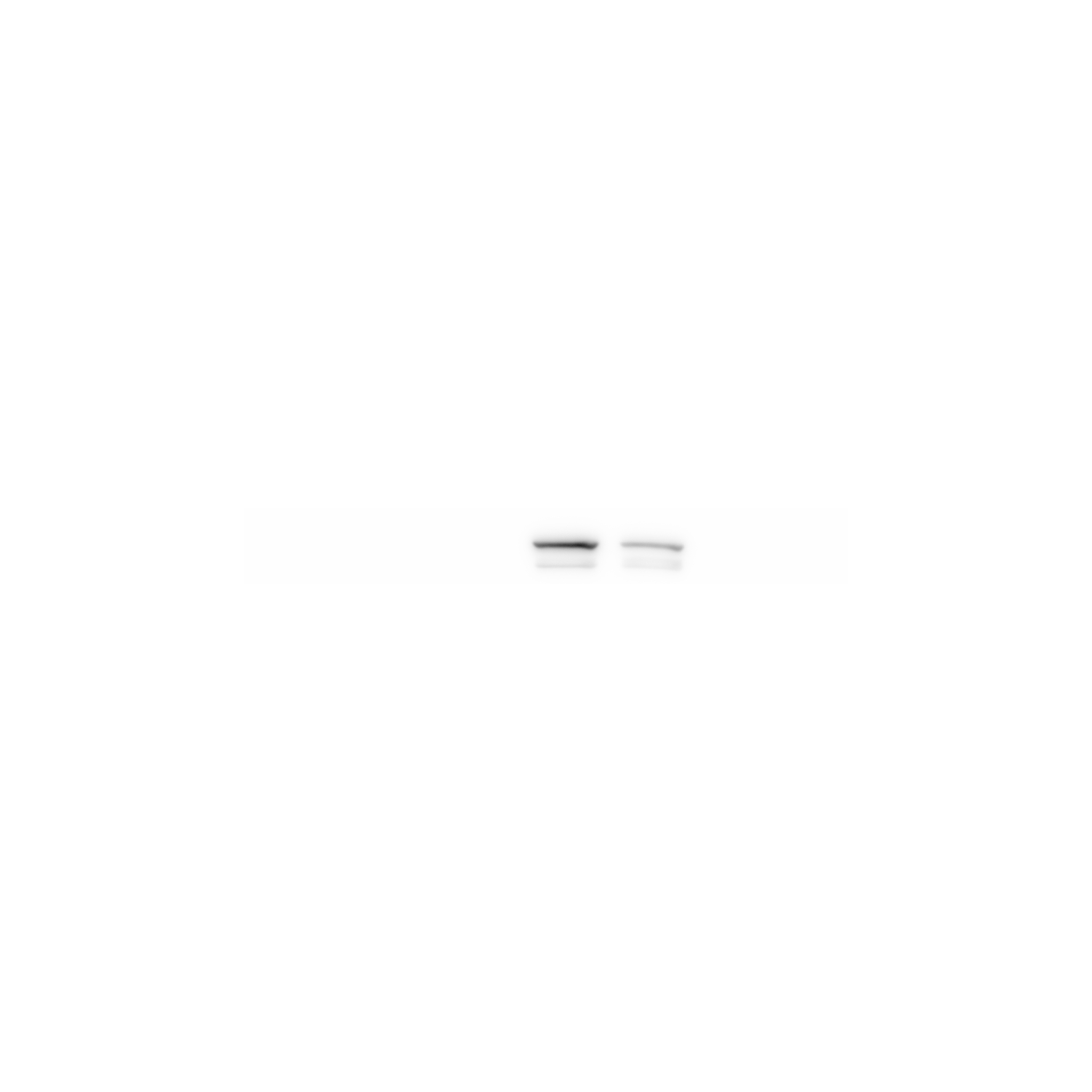
**

Western blot analysis of Vimentin protein levels in MCF7 cells expressing either the control vector or HTR2C.

**Figure 4C_N-cadherin in MCF7_ source data**

**
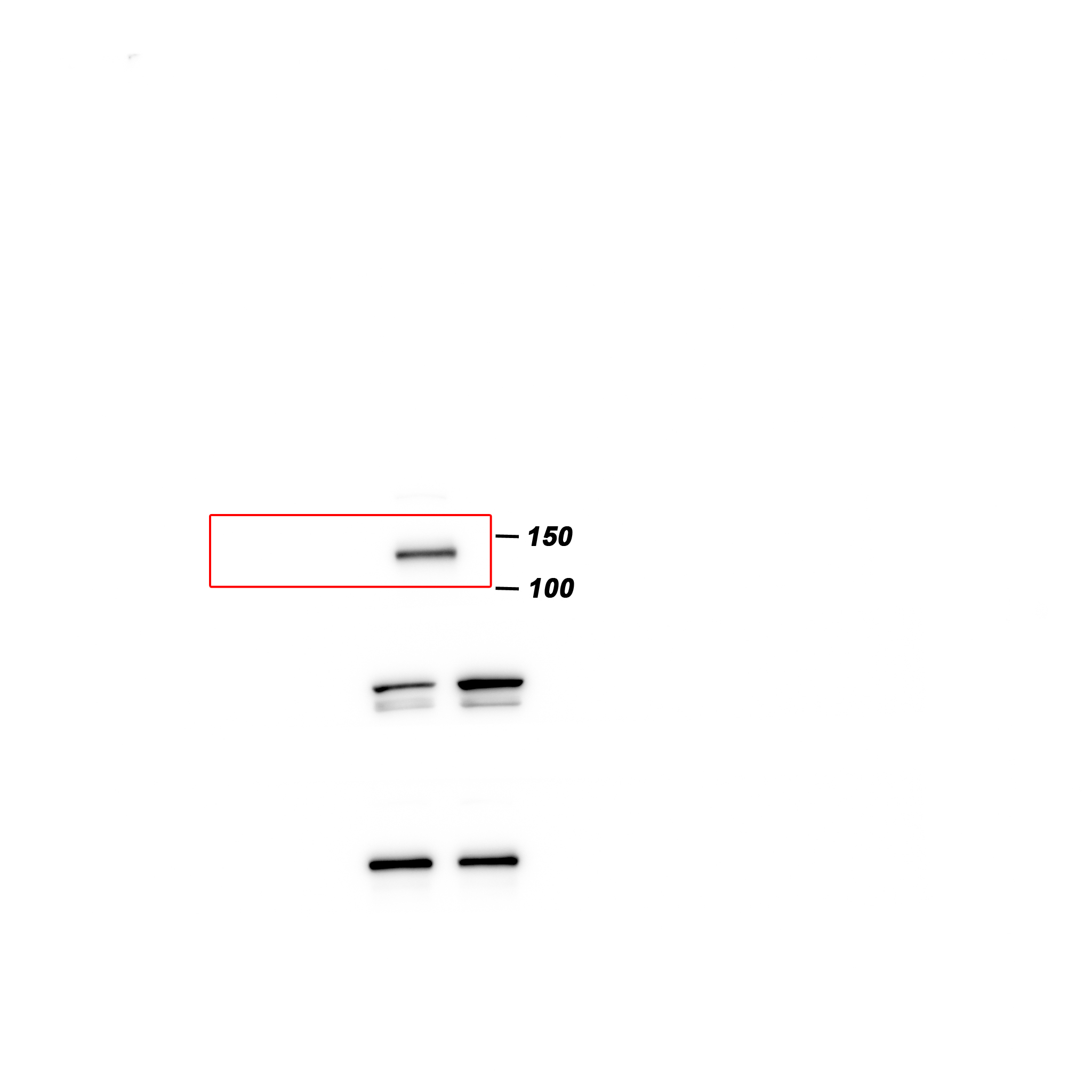

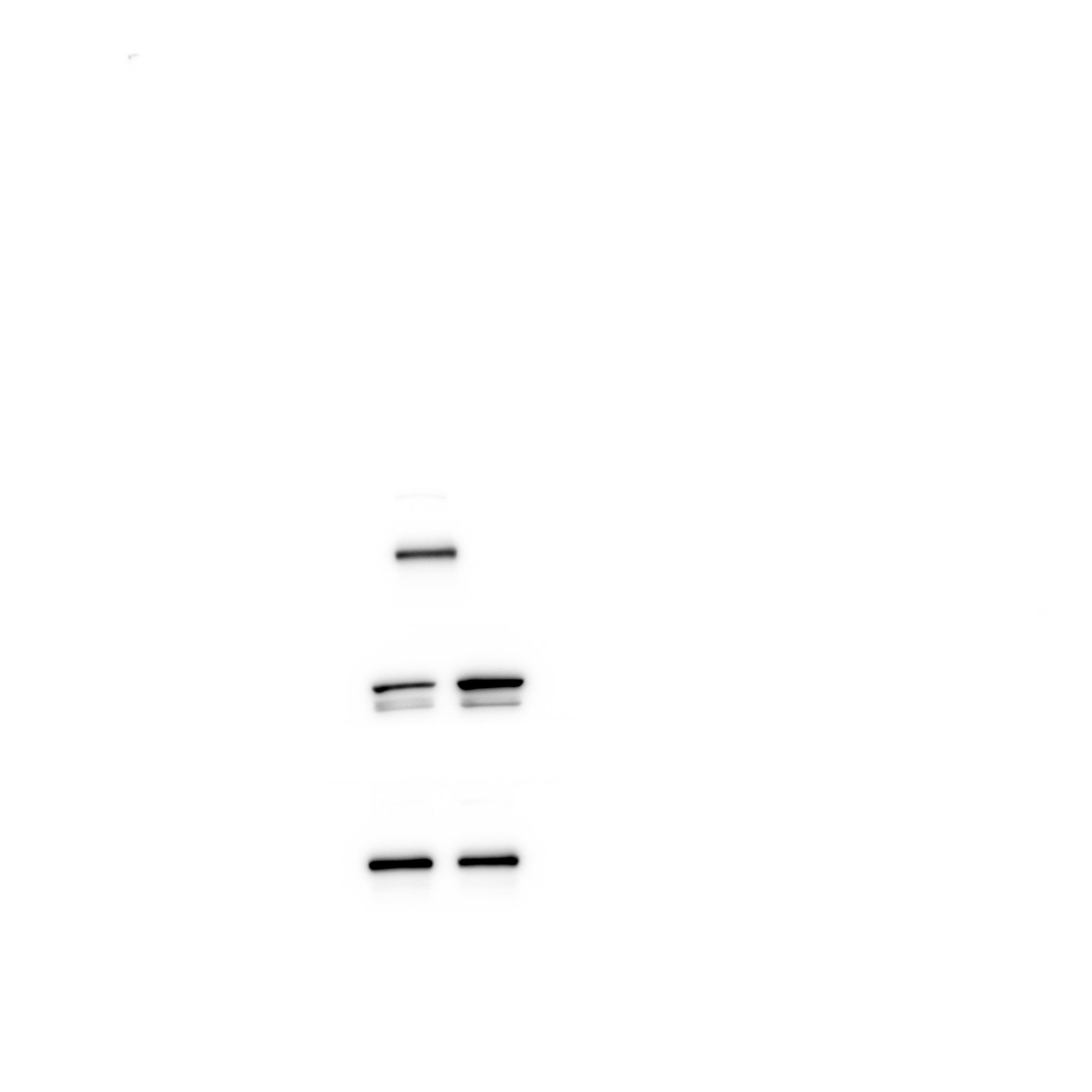
**

Western blot analysis of N-cadherin protein levels in MCF7 cells expressing either the control vector or HTR2C.

**Figure 4C_ZEB1 in MCF7_ source data**

**
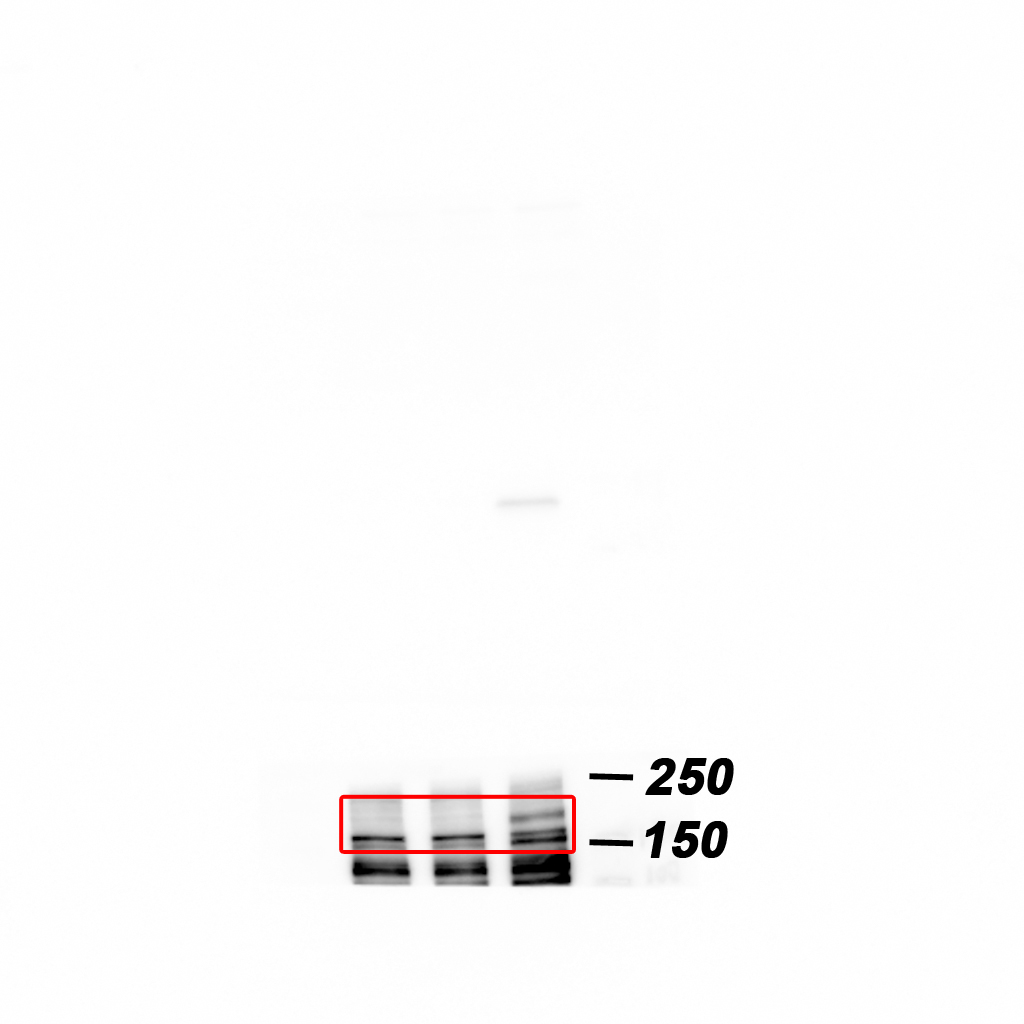

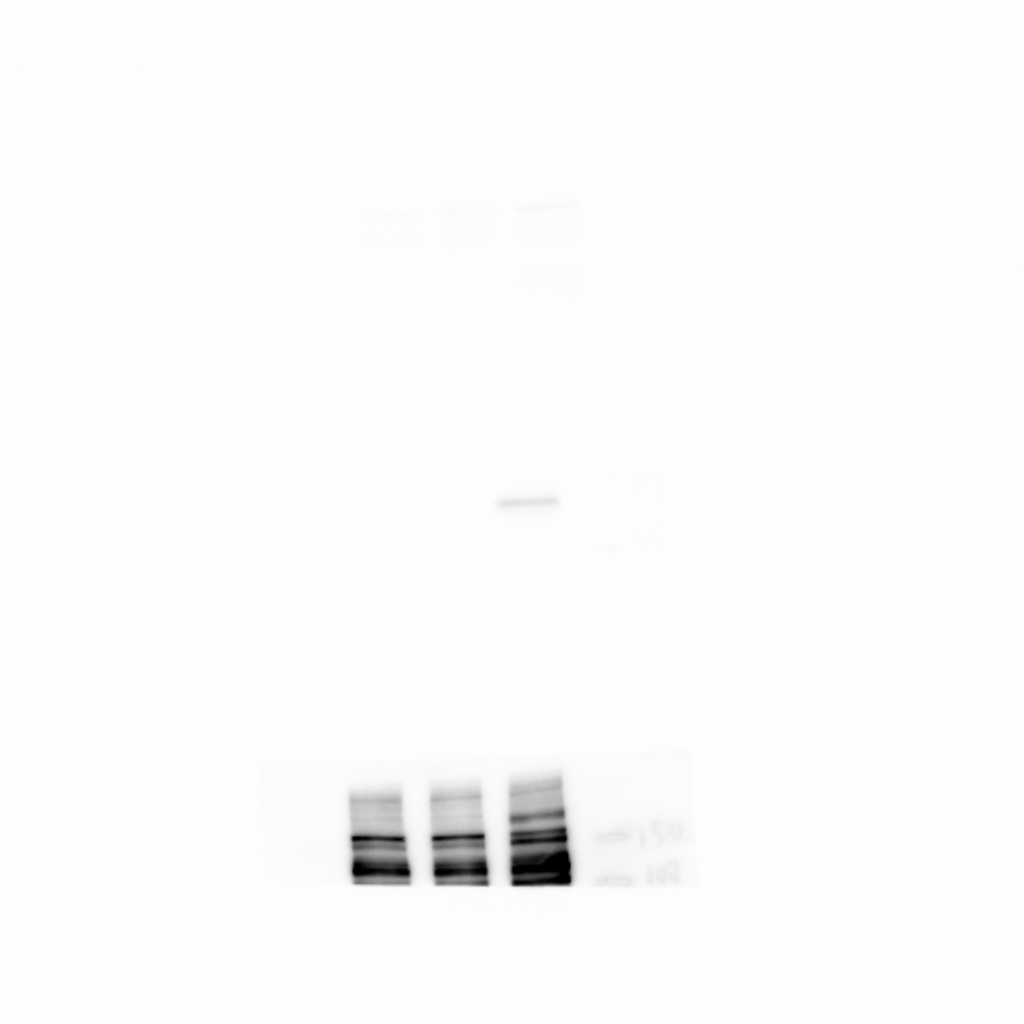
**

Western blot analysis of ZEB1 protein levels in MCF7 cells expressing either the control vector or HTR2C.

**Figure 4C_HRT2C in MCF7_ source data**

**
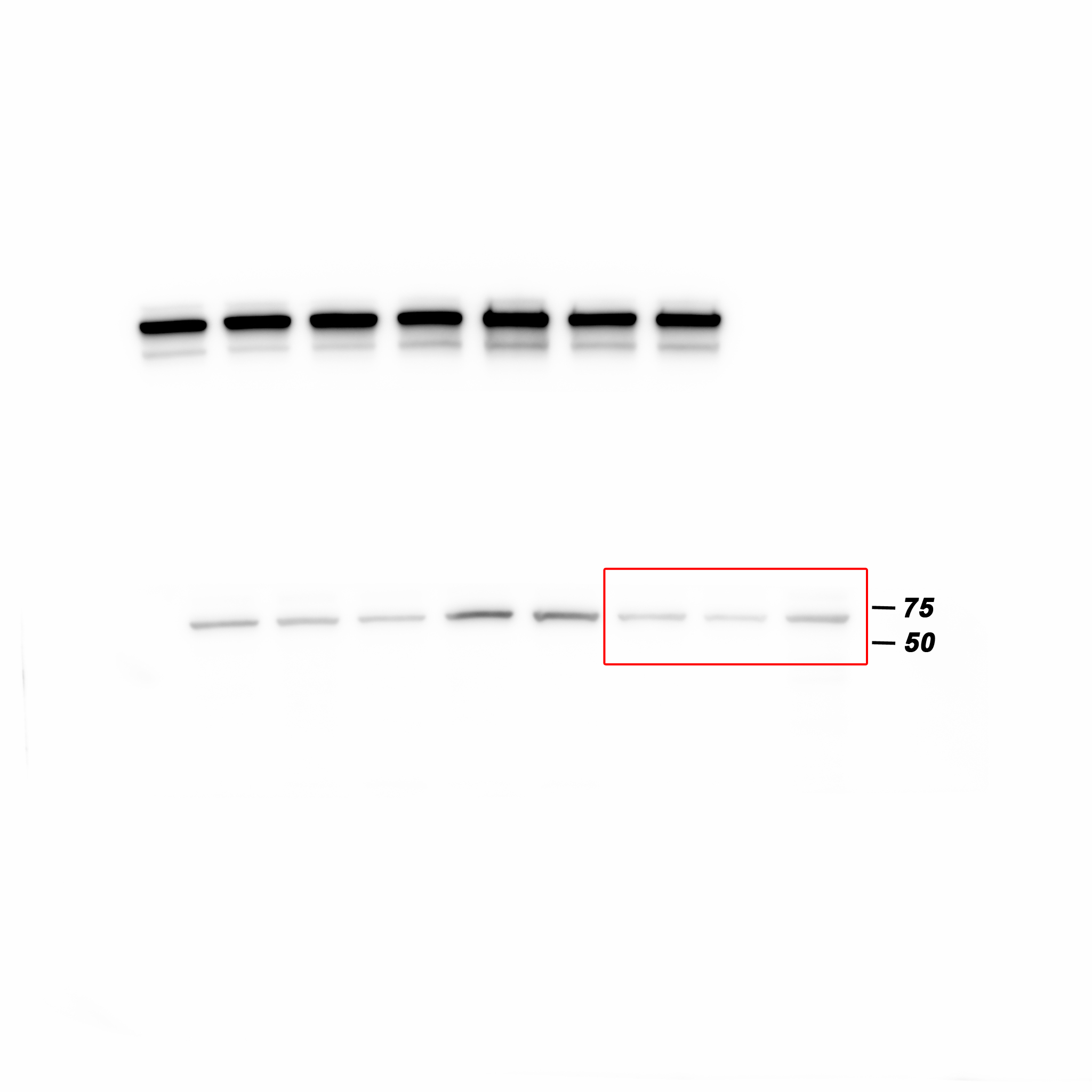

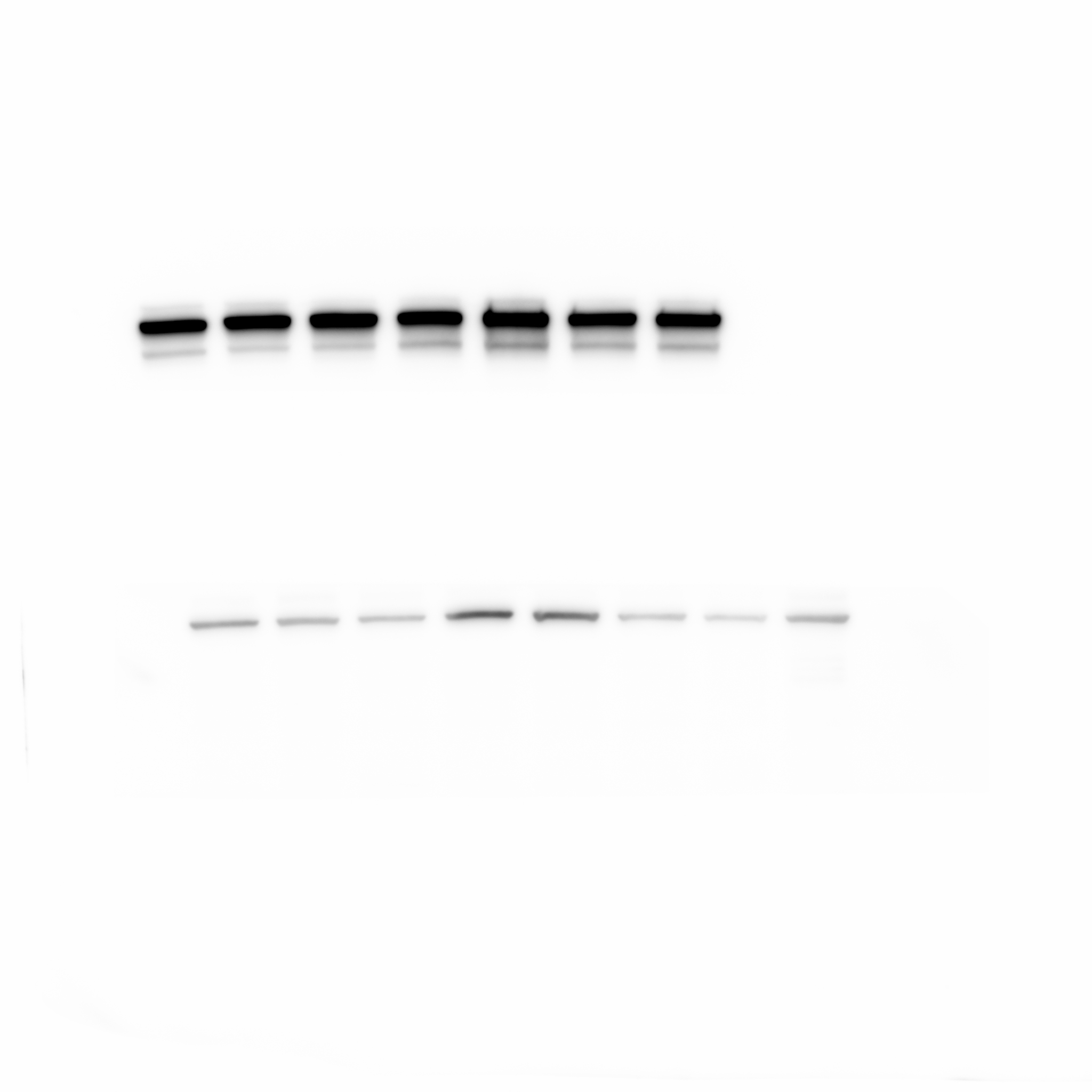
**

Western blot analysis of HRT2C protein levels in MCF7 cells expressing either the control vector or HTR2C.

**Figure 4C_GAPDH in MCF7_source data**

**
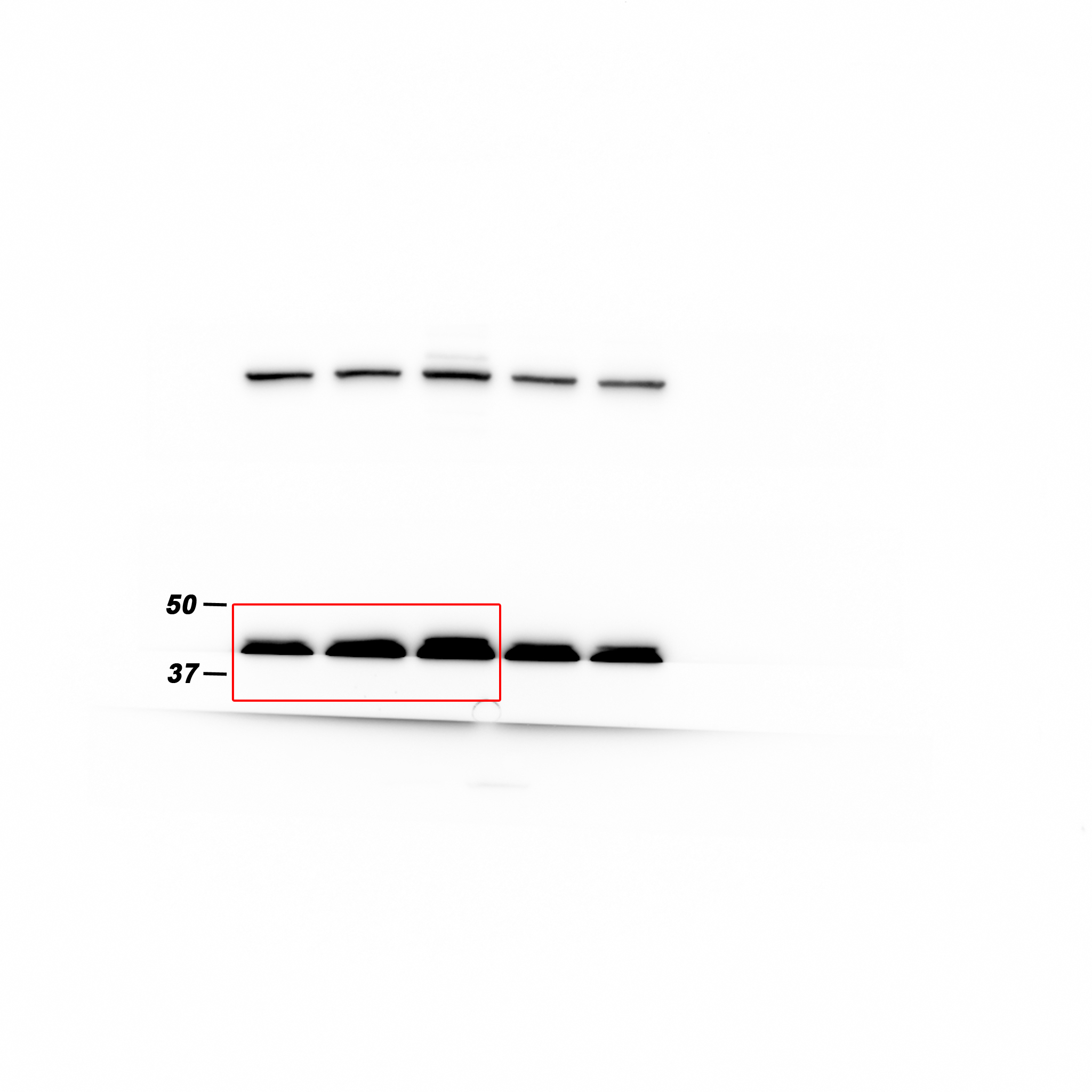

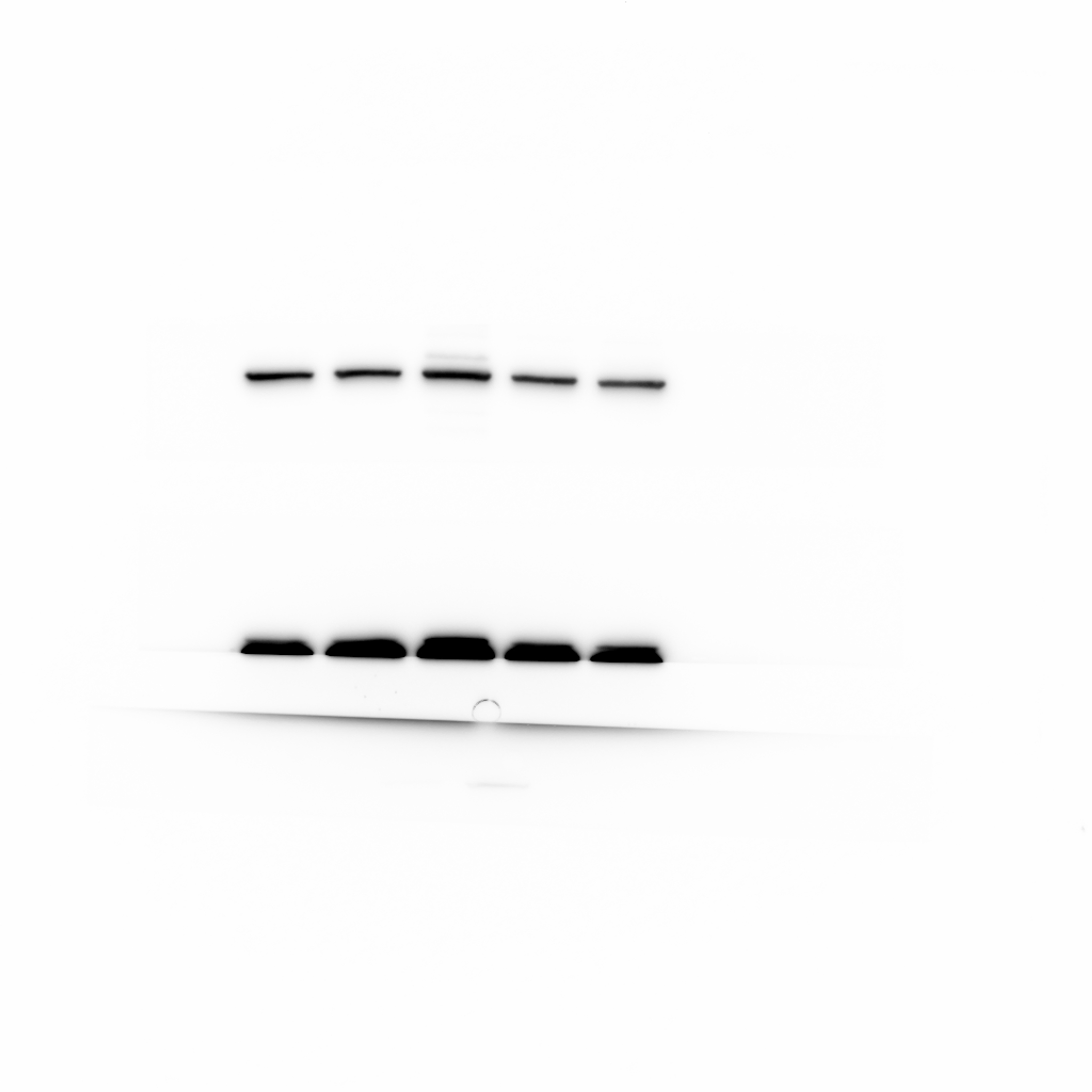
**

Western blot analysis of GAPDH protein levels in MCF7 cells expressing either the control vector or HTR2C.

**Figure 4C_E-cadherin in HaCaT_source data**

**
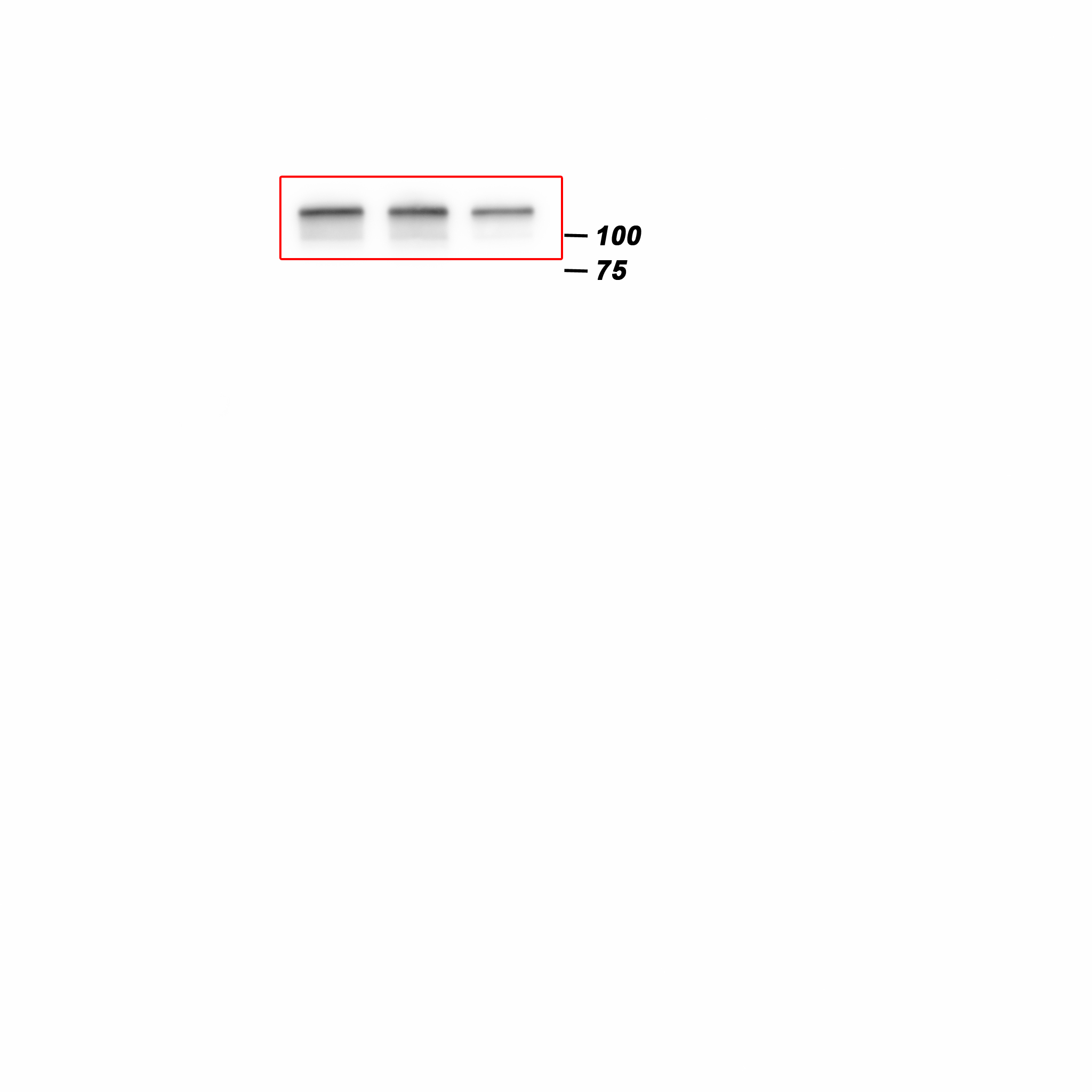

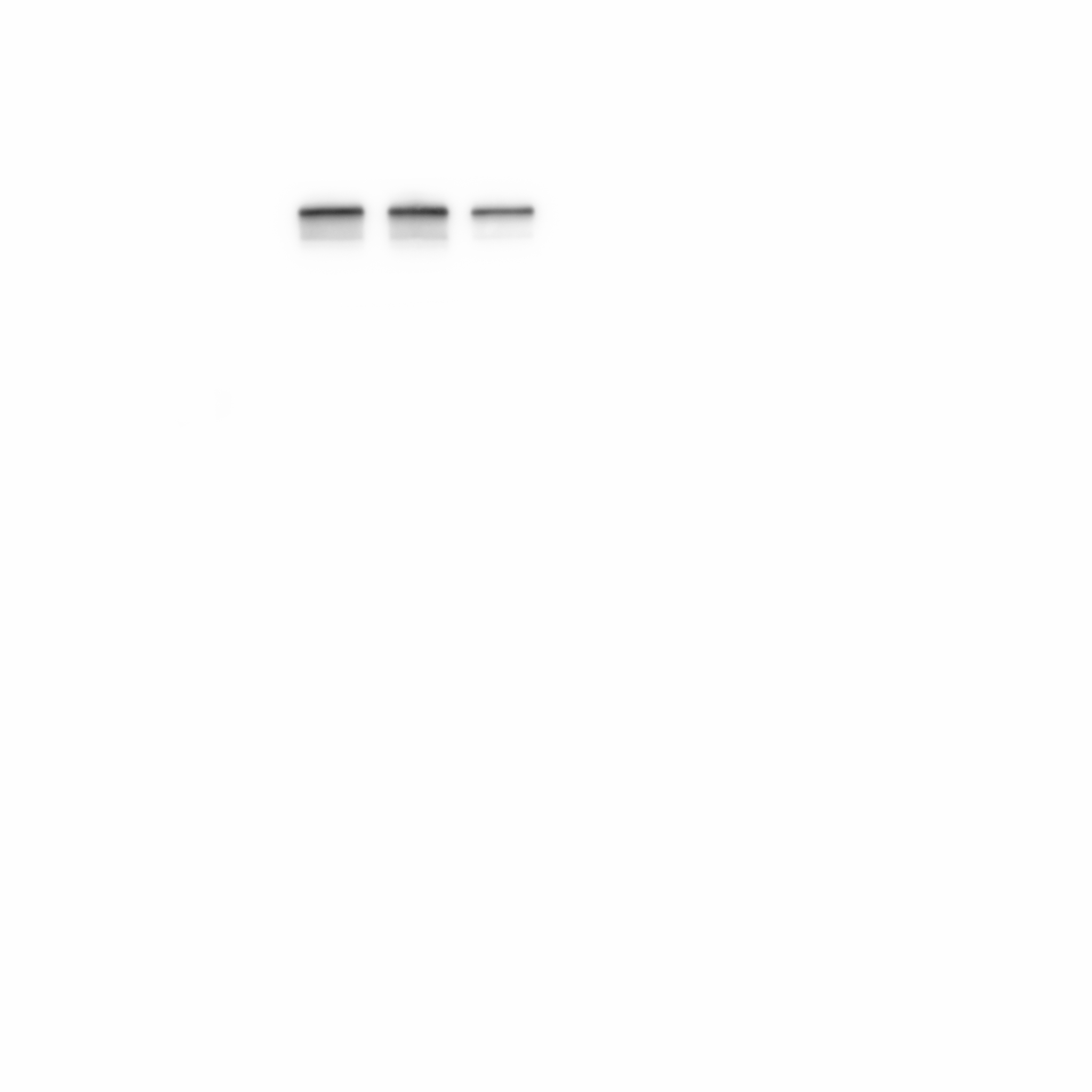
**

Western blot analysis of E-cadherin protein levels in HaCaT cells expressing either the control vector or HTR2C.

**Figure 4C_EpCAM in HaCaT_source data**

**
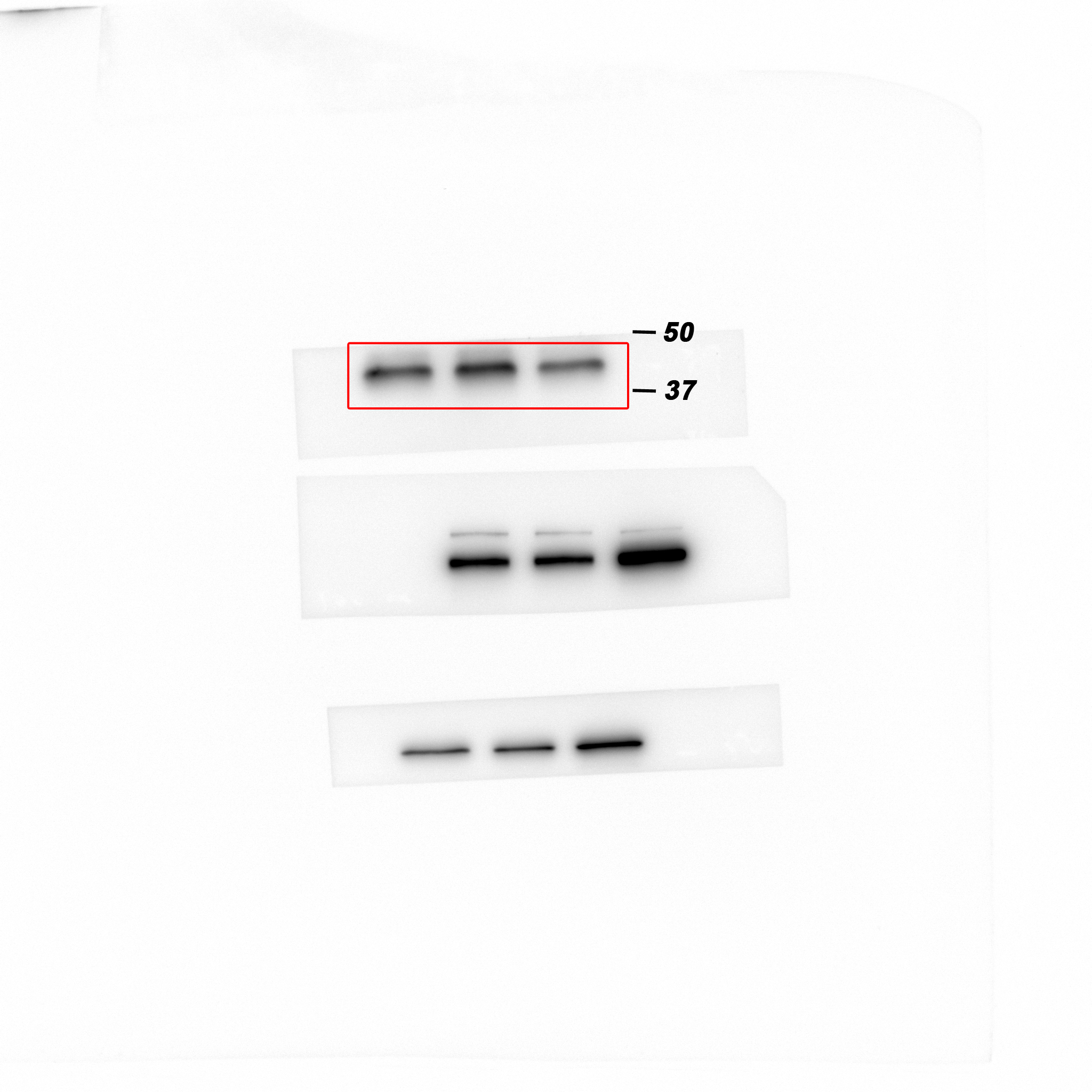

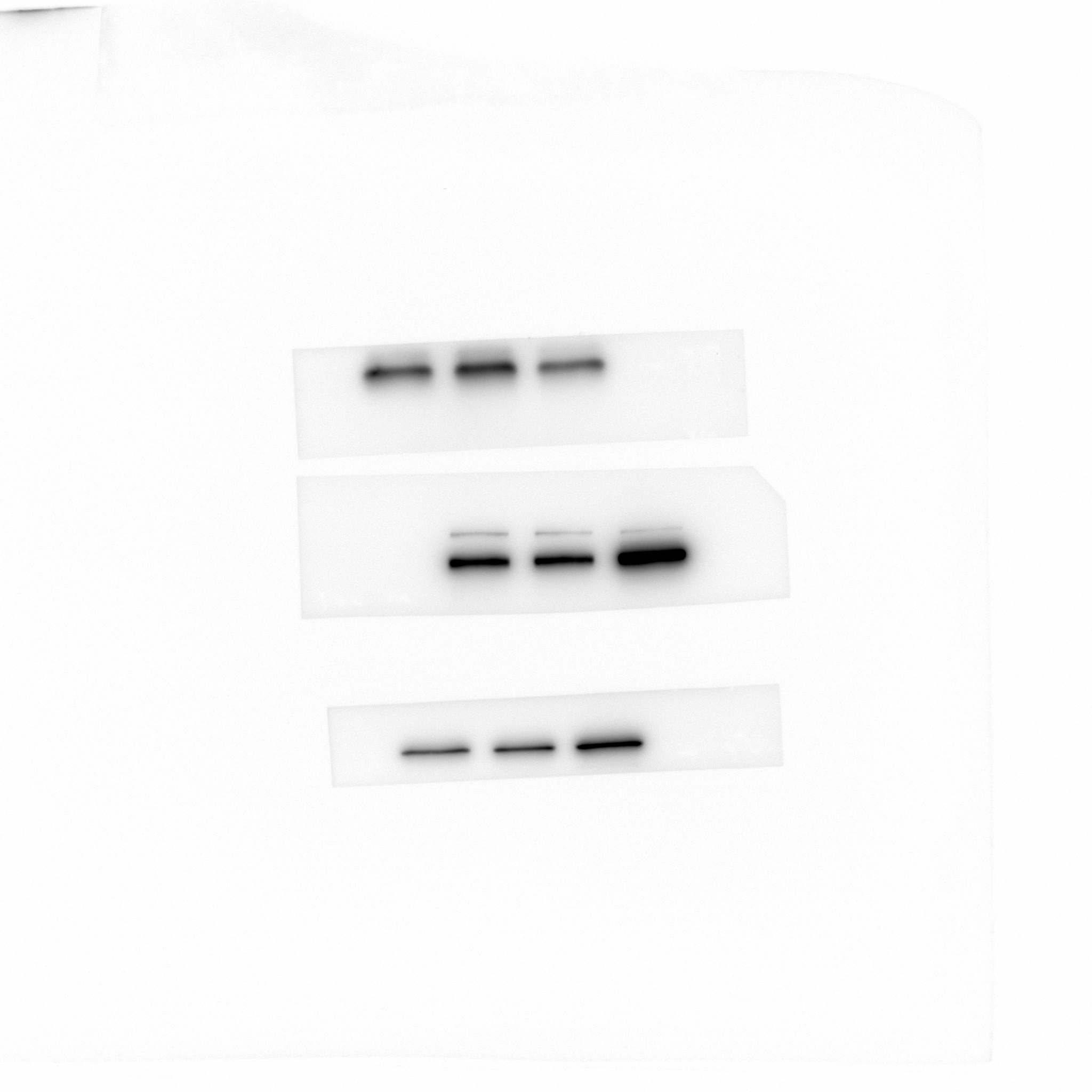
**

Western blot analysis of EpCAM protein levels in HaCaT cells expressing either the control vector or HTR2C.

**Figure 4C_Vimentin in HaCaT_source data**

**
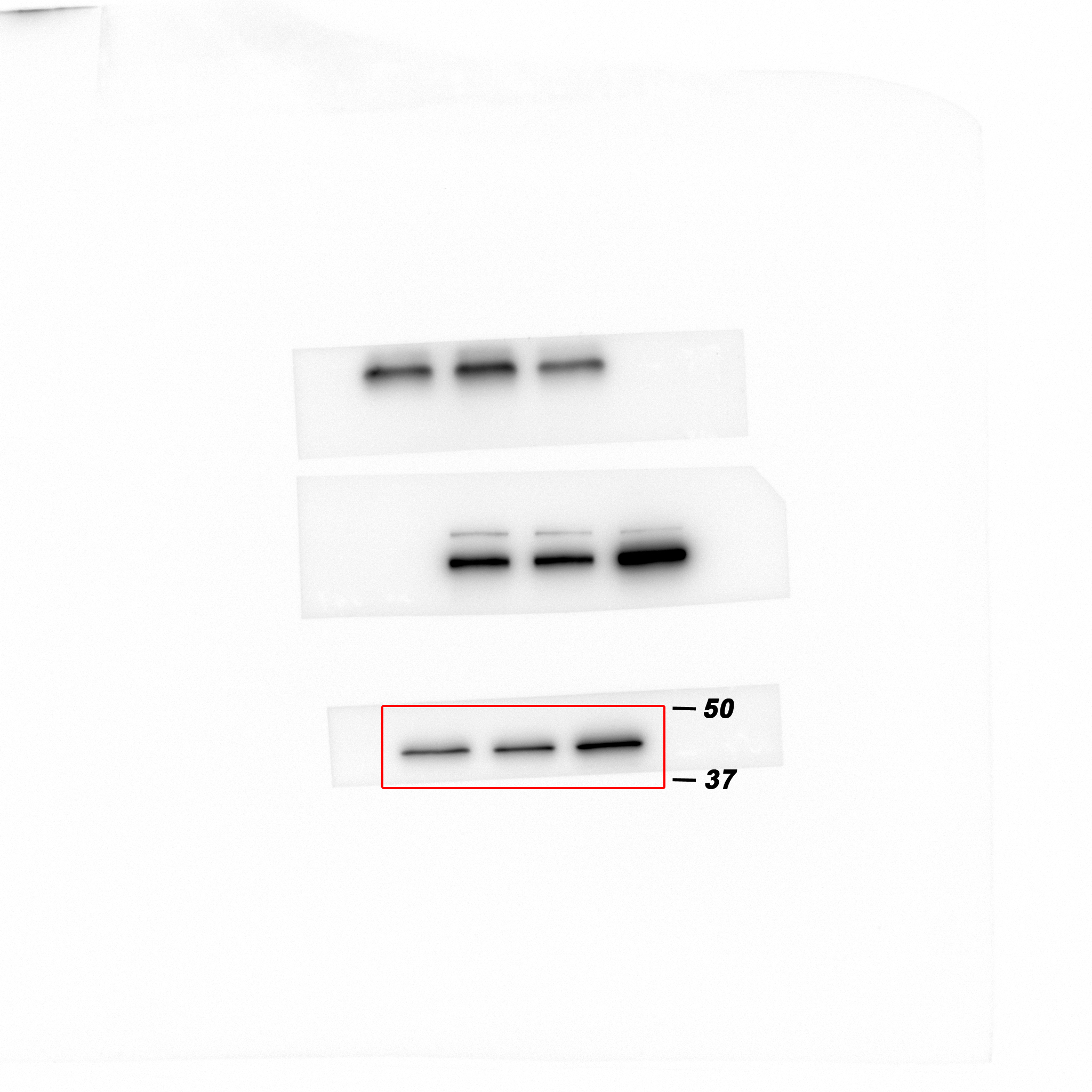

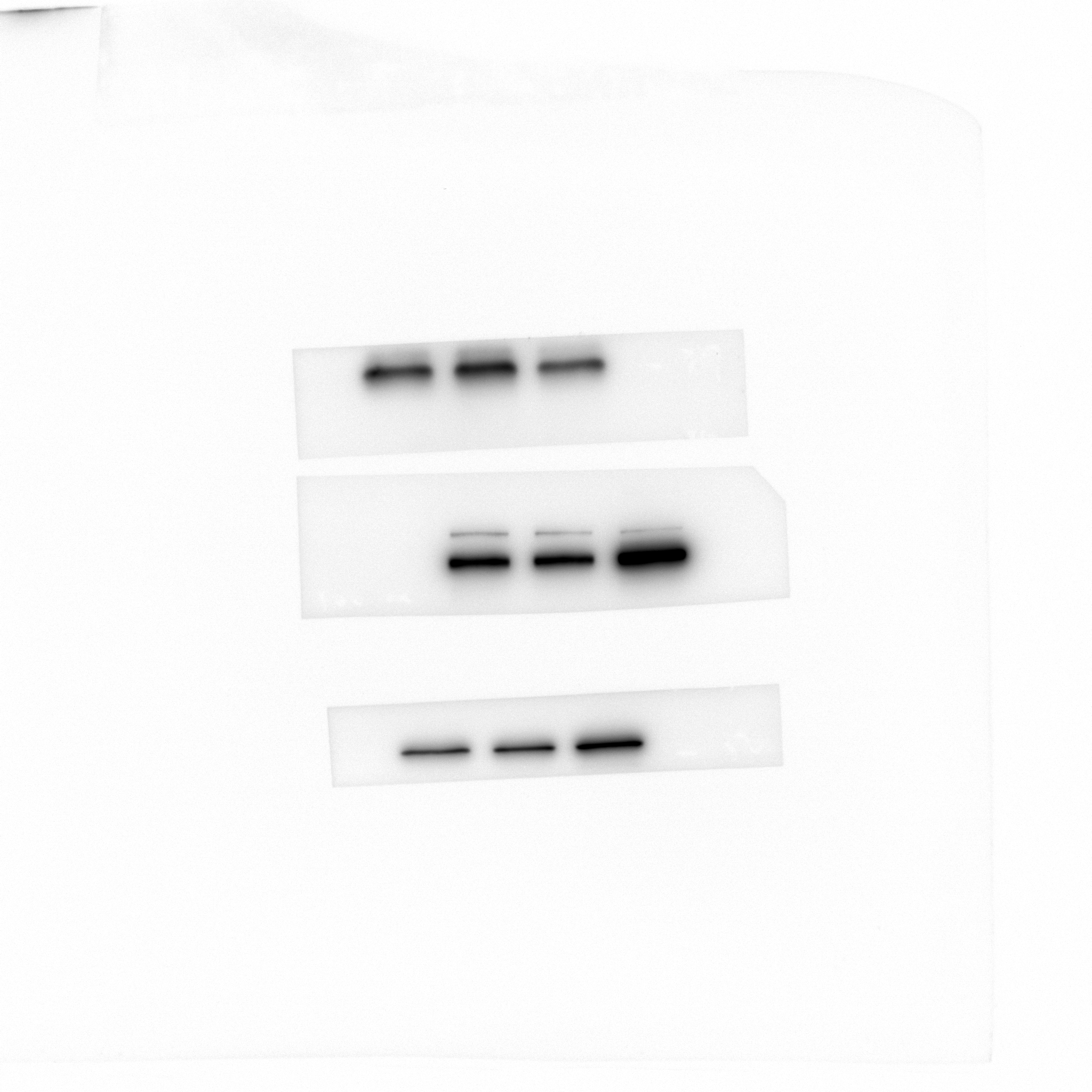
**

Western blot analysis of Vimentin protein levels in HaCaT cells expressing either the control vector or HTR2C.

**Figure 4C_N-cadherin in HaCaT_source data**

**
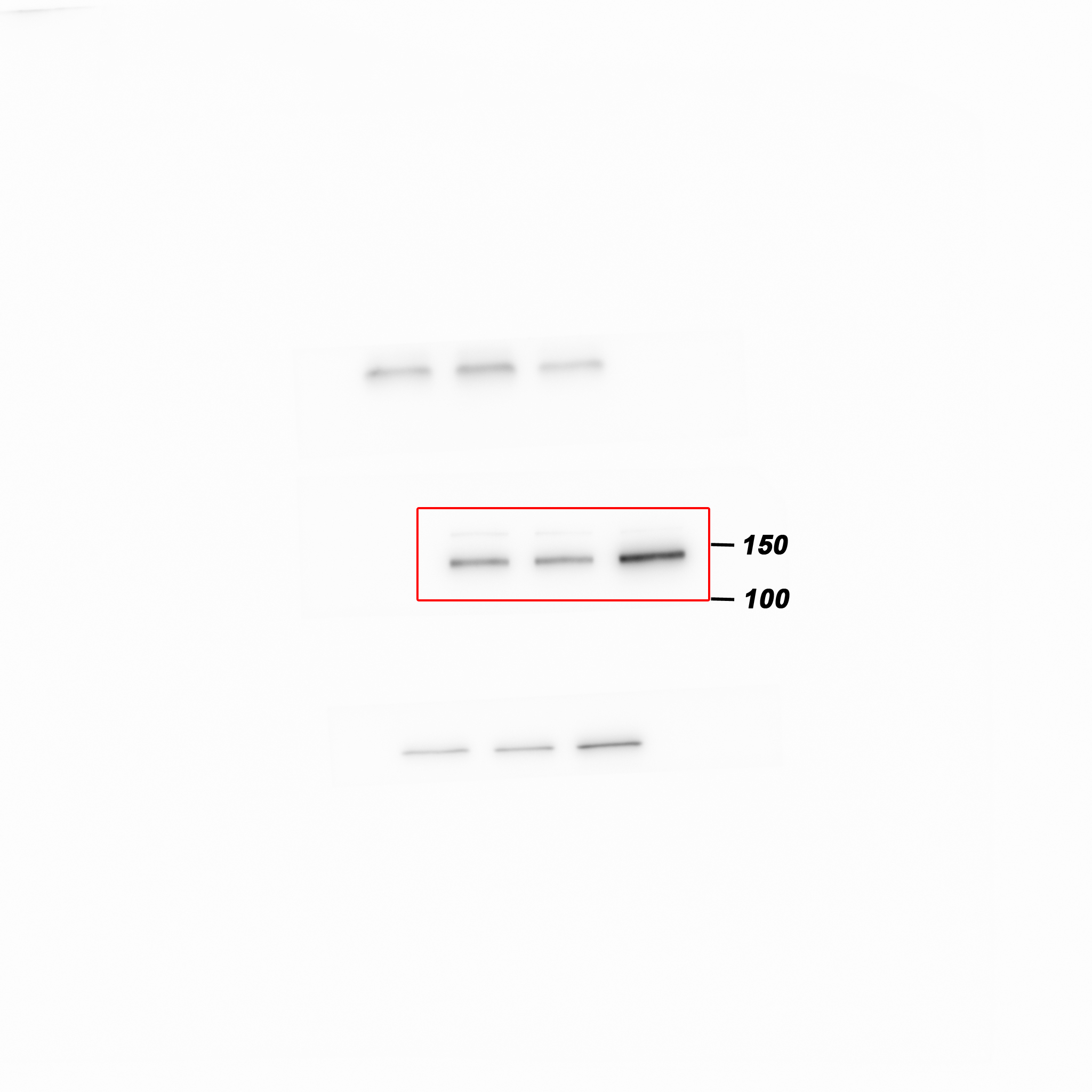

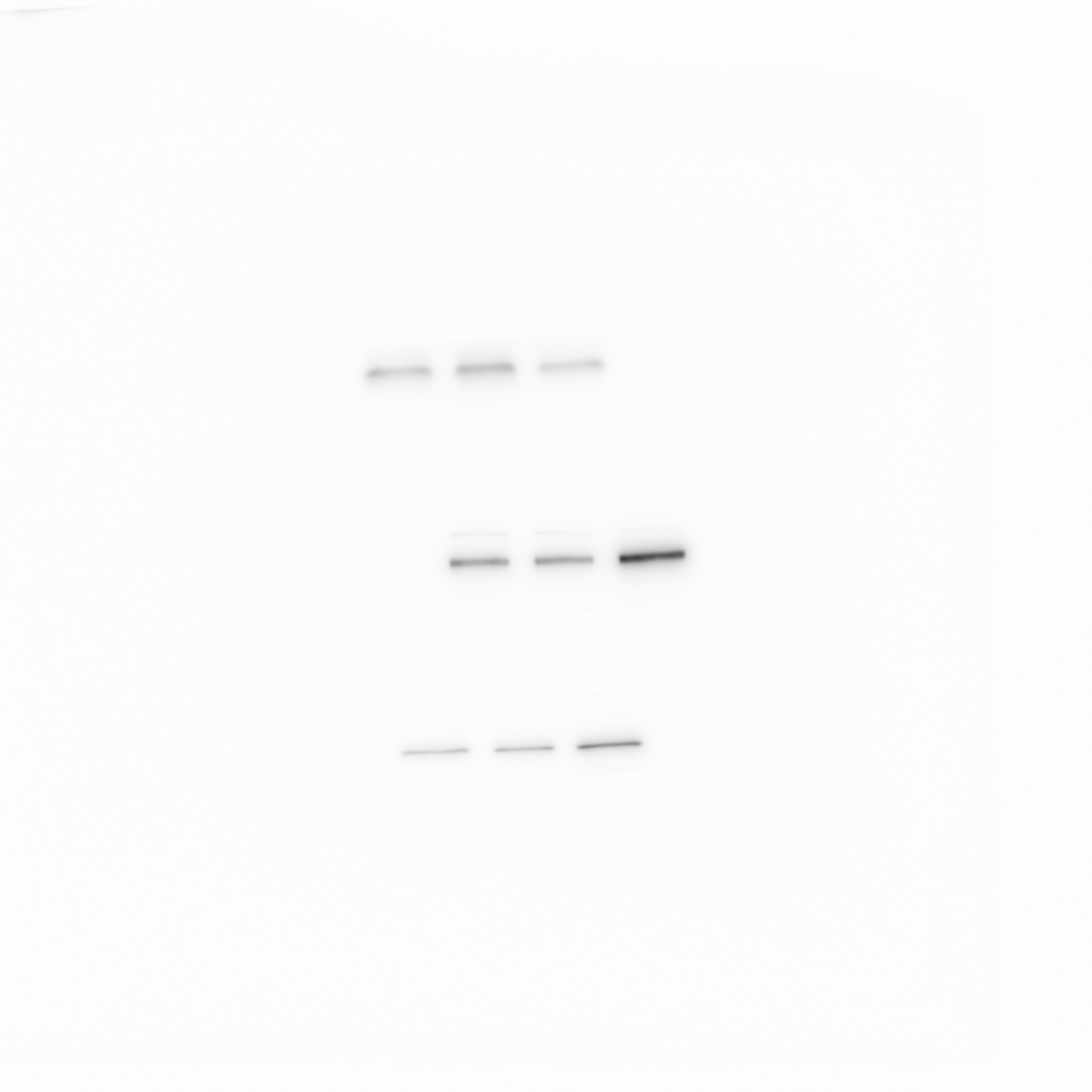
**

Western blot analysis of N-cadherin protein levels in HaCaT cells expressing either the control vector or HTR2C.

**Figure 4C_ZEB1 in HaCaT source data**

**
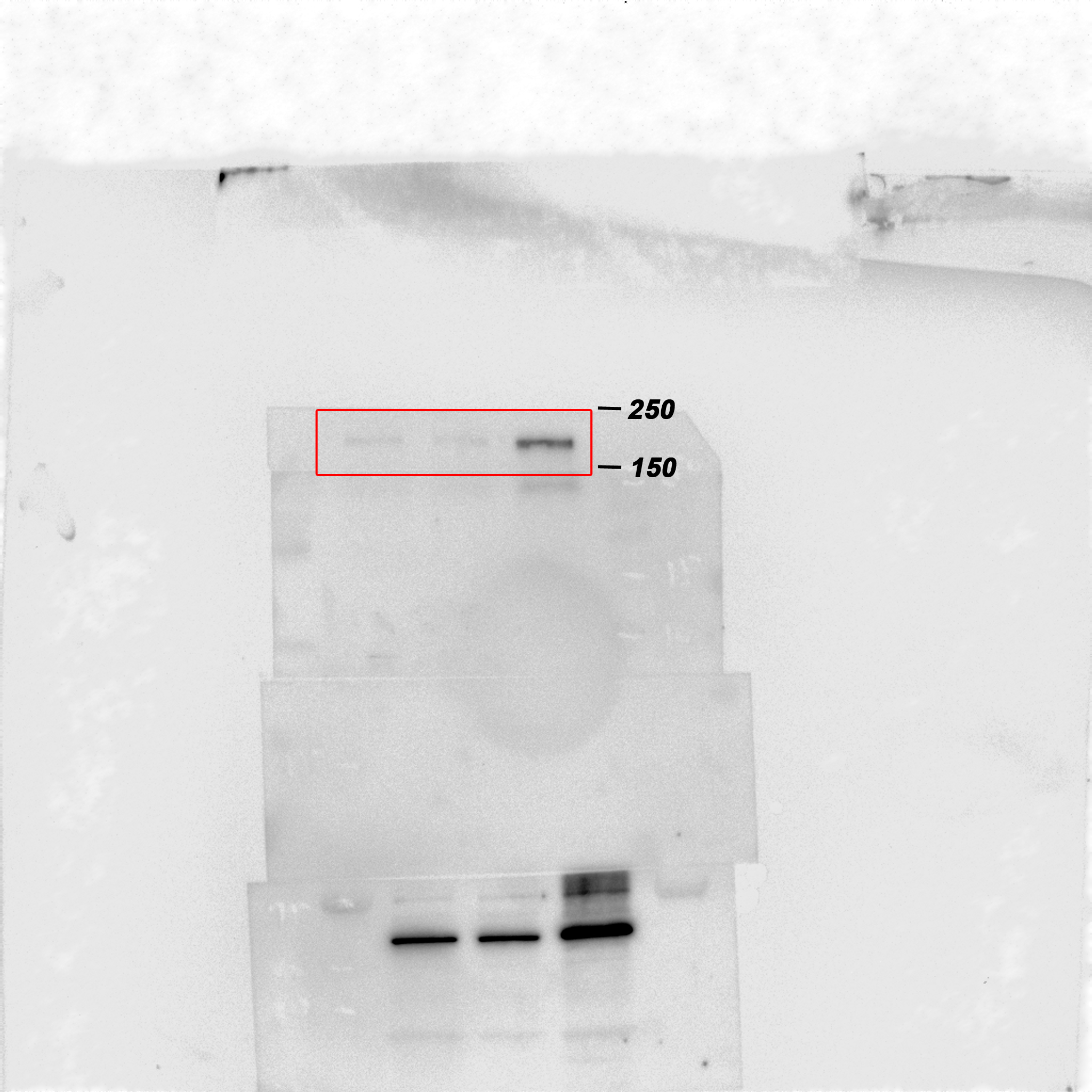

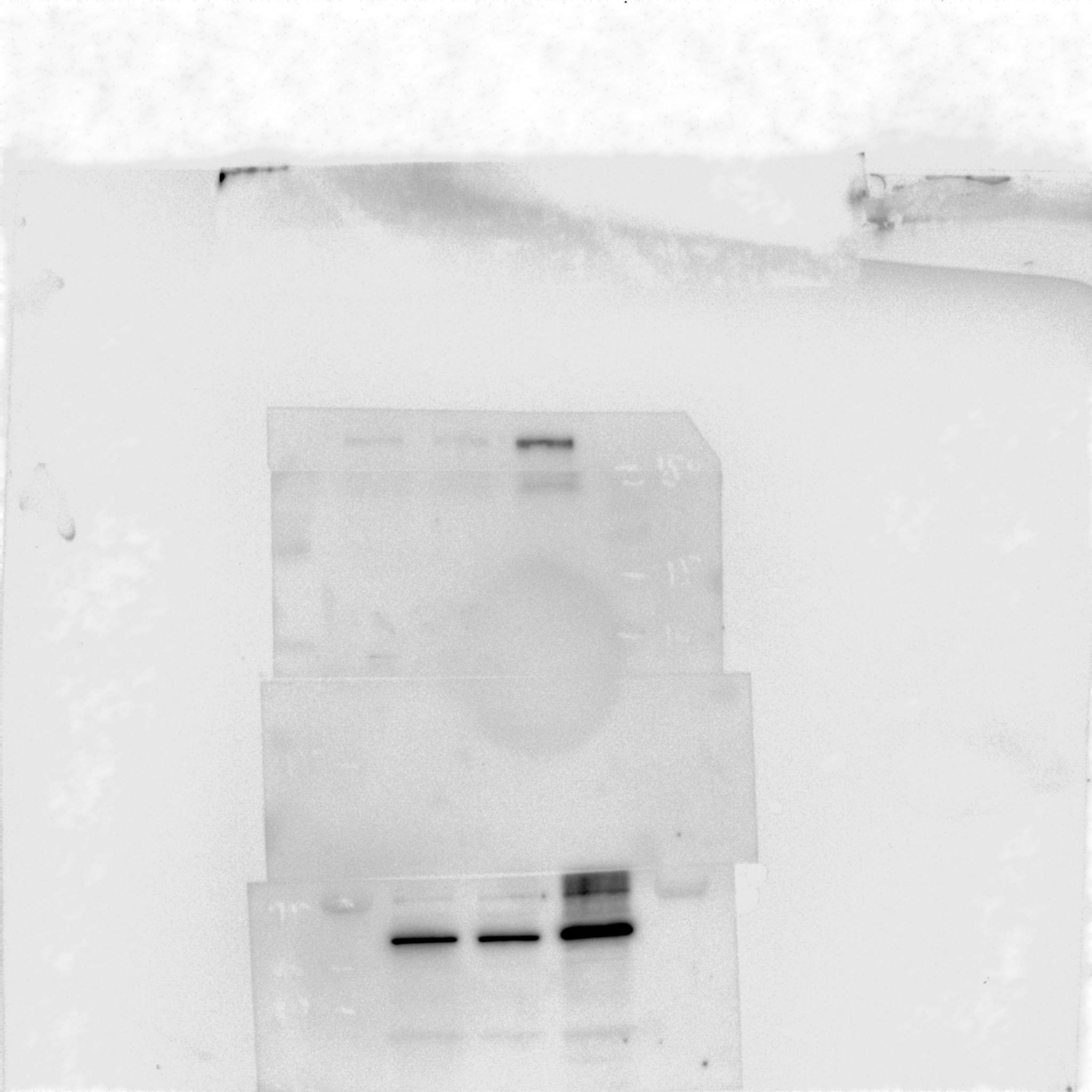
**

Western blot analysis of ZEB1 protein levels in HaCaT cells expressing either the control vector or HTR2C.

**Figure 4C_HRT2C in HaCaT_source data**

**
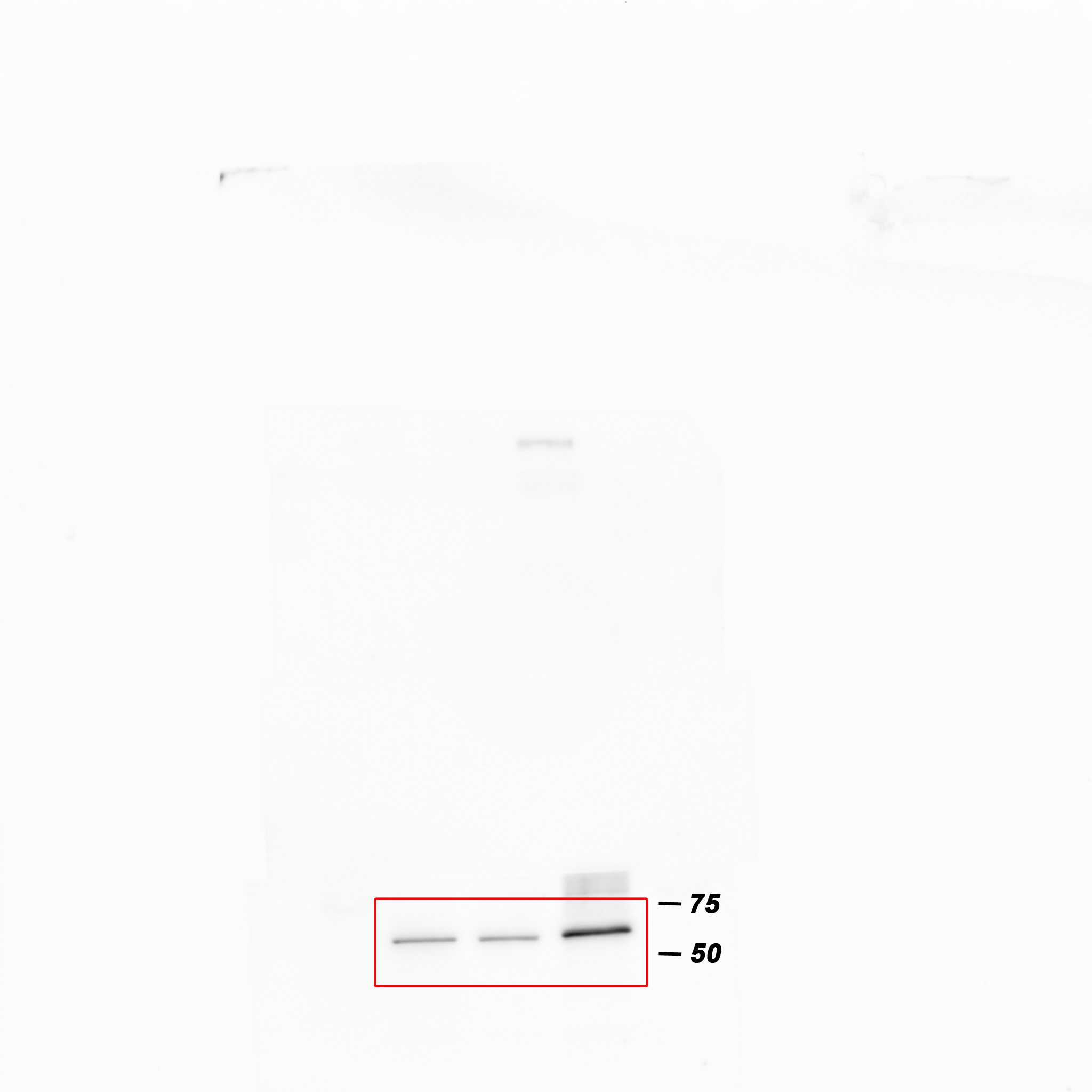

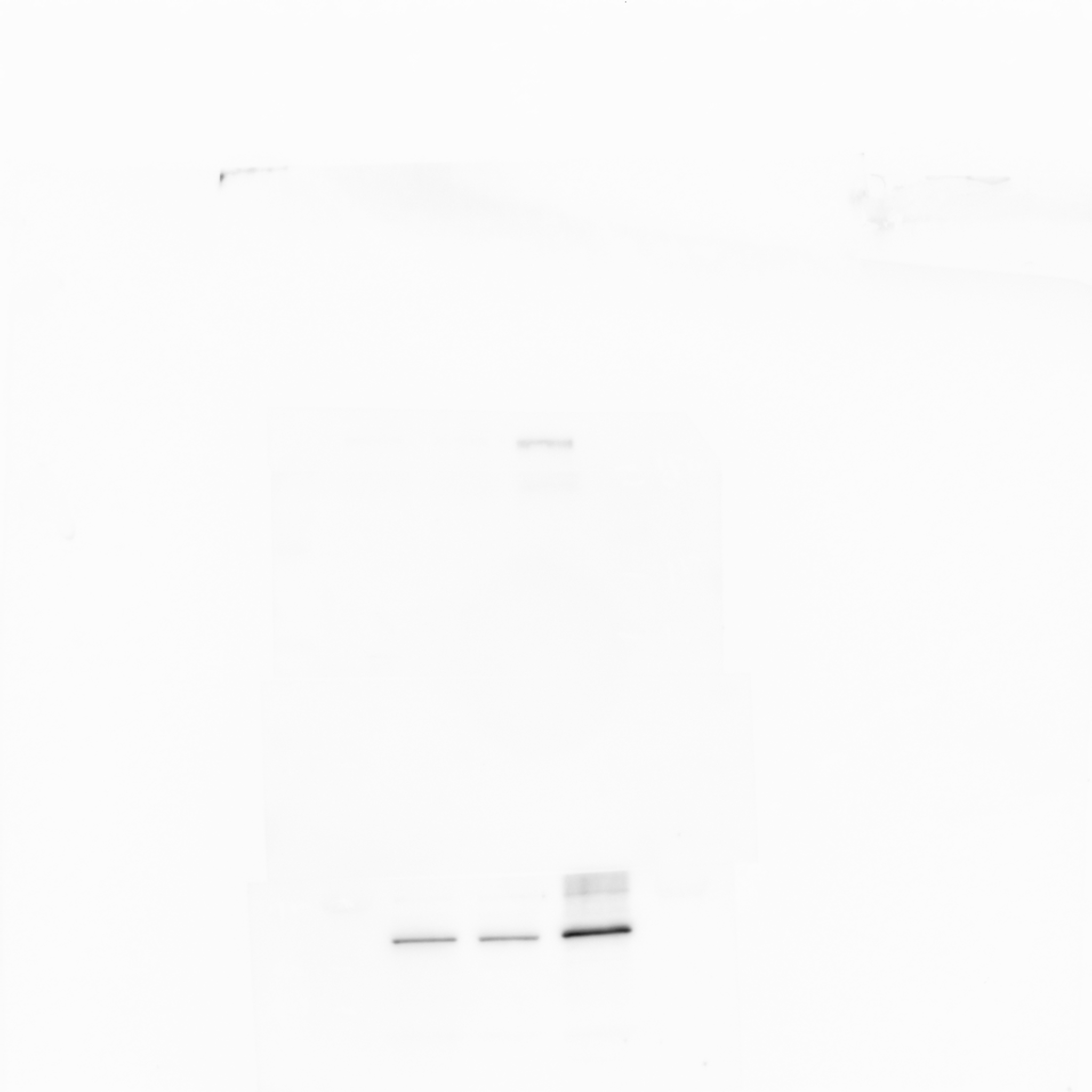
**

Western blot analysis of HRT2C protein levels in HaCaT cells expressing either the control vector or HTR2C.

**Figure 4C_GAPDH in HaCaT_source data**

**
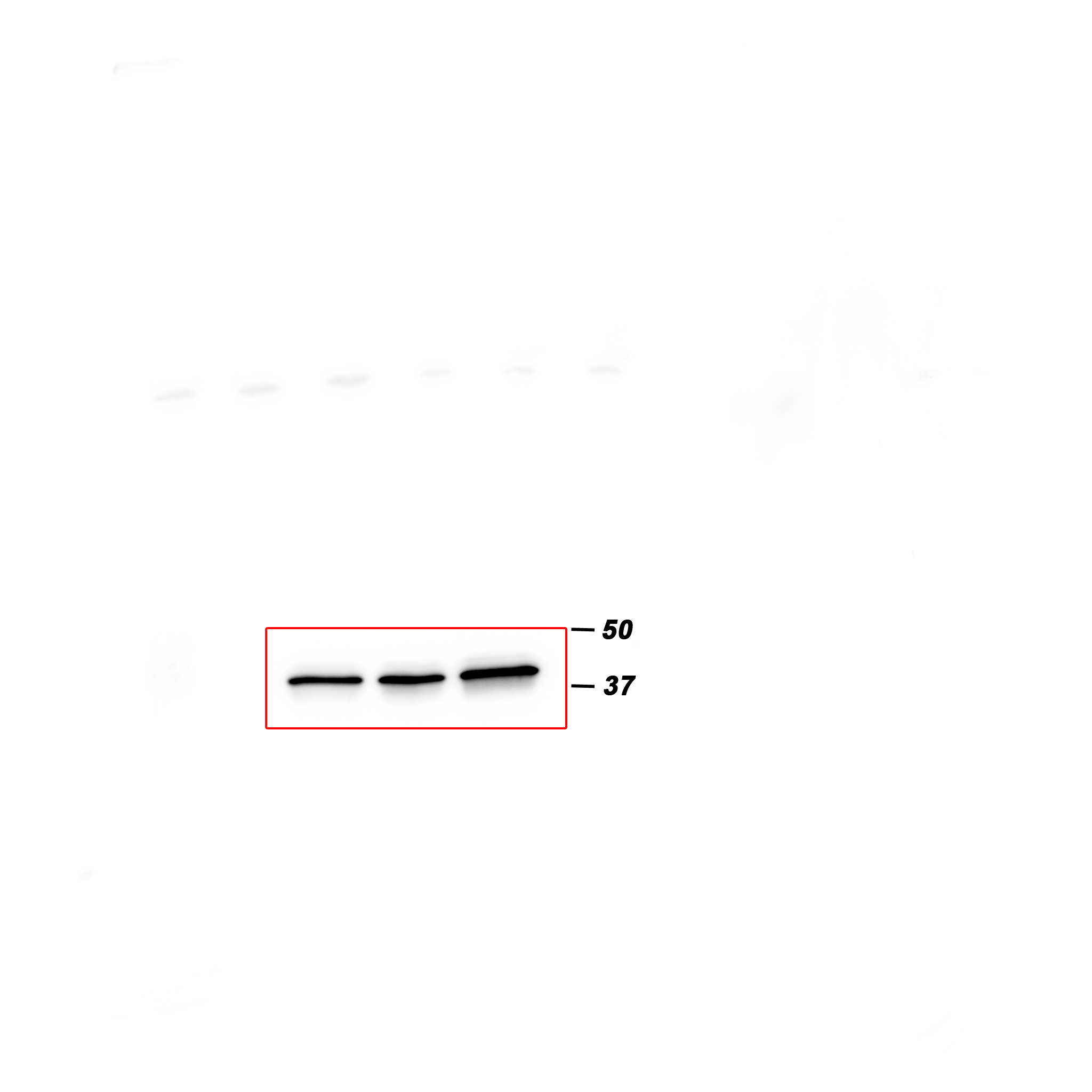

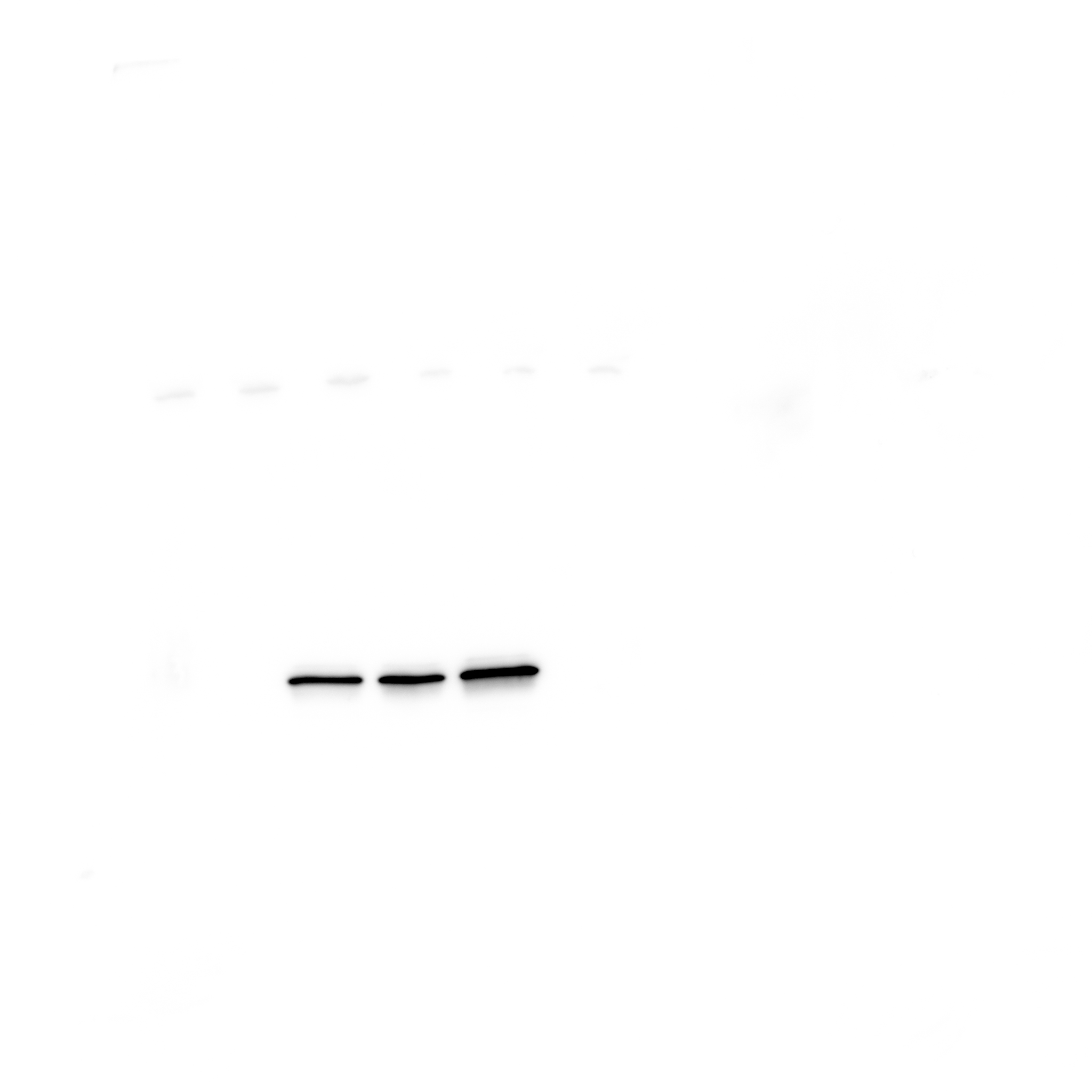
**

Western blot analysis of GAPDH protein levels in HaCaT cells expressing either the control vector or HTR2C.

**Figure 5C_E-cadherin_source data**

**
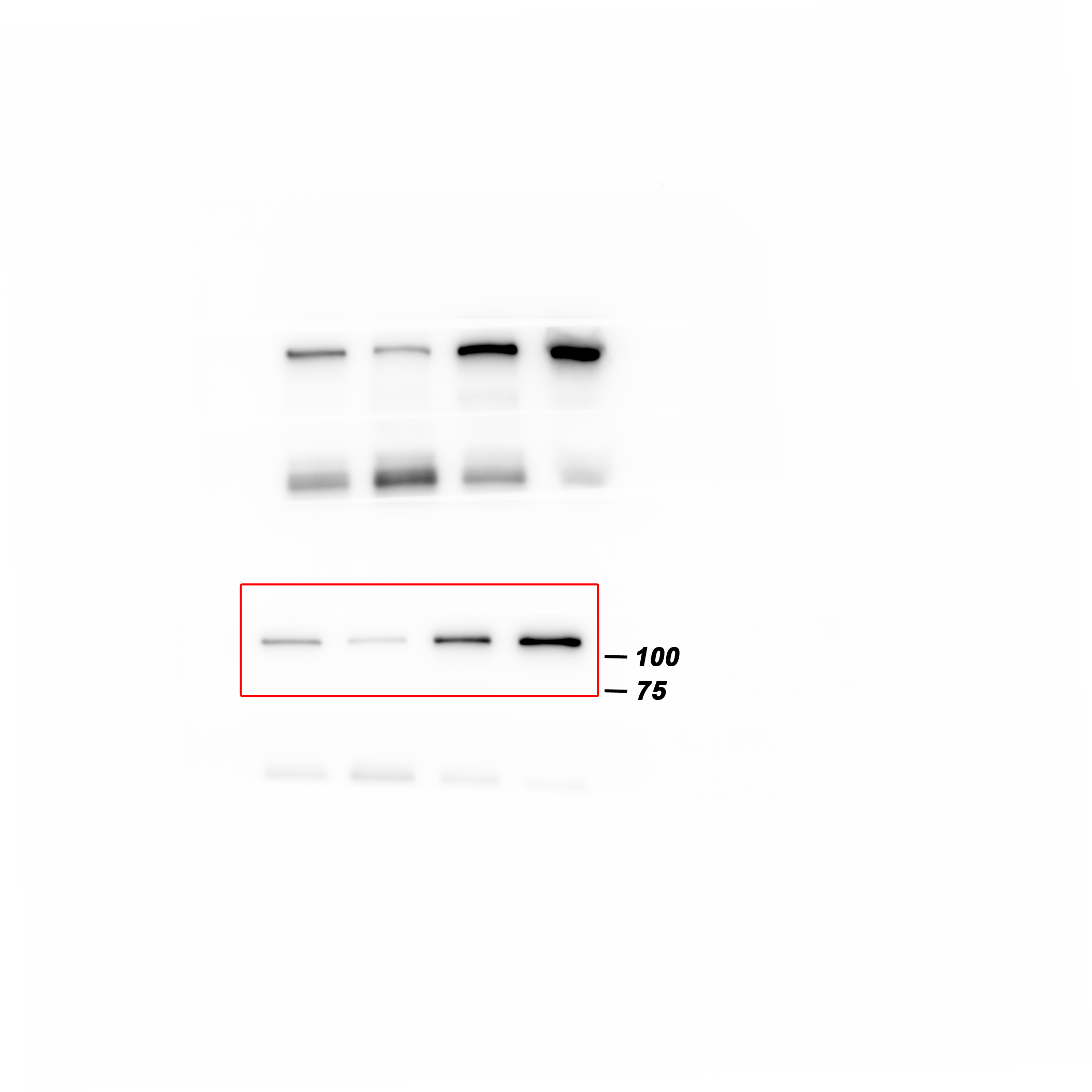

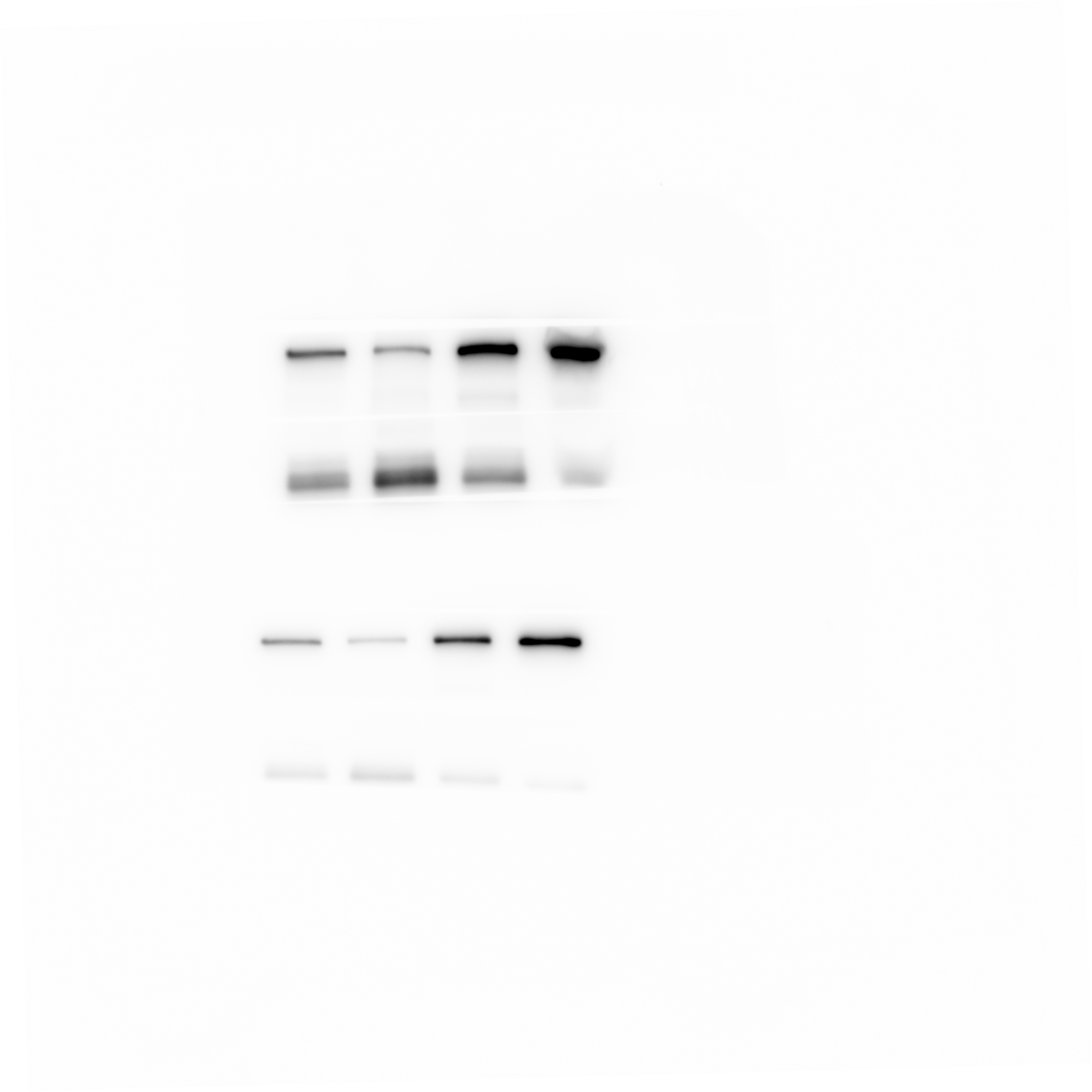
**

Western blot analysis of E-cadherin protein levels in whole cell lysate of 4T1 primary tumors from either vehicle or Pizotifen-treated mice.

**Figure 5C_ZEB1 _source data**

**
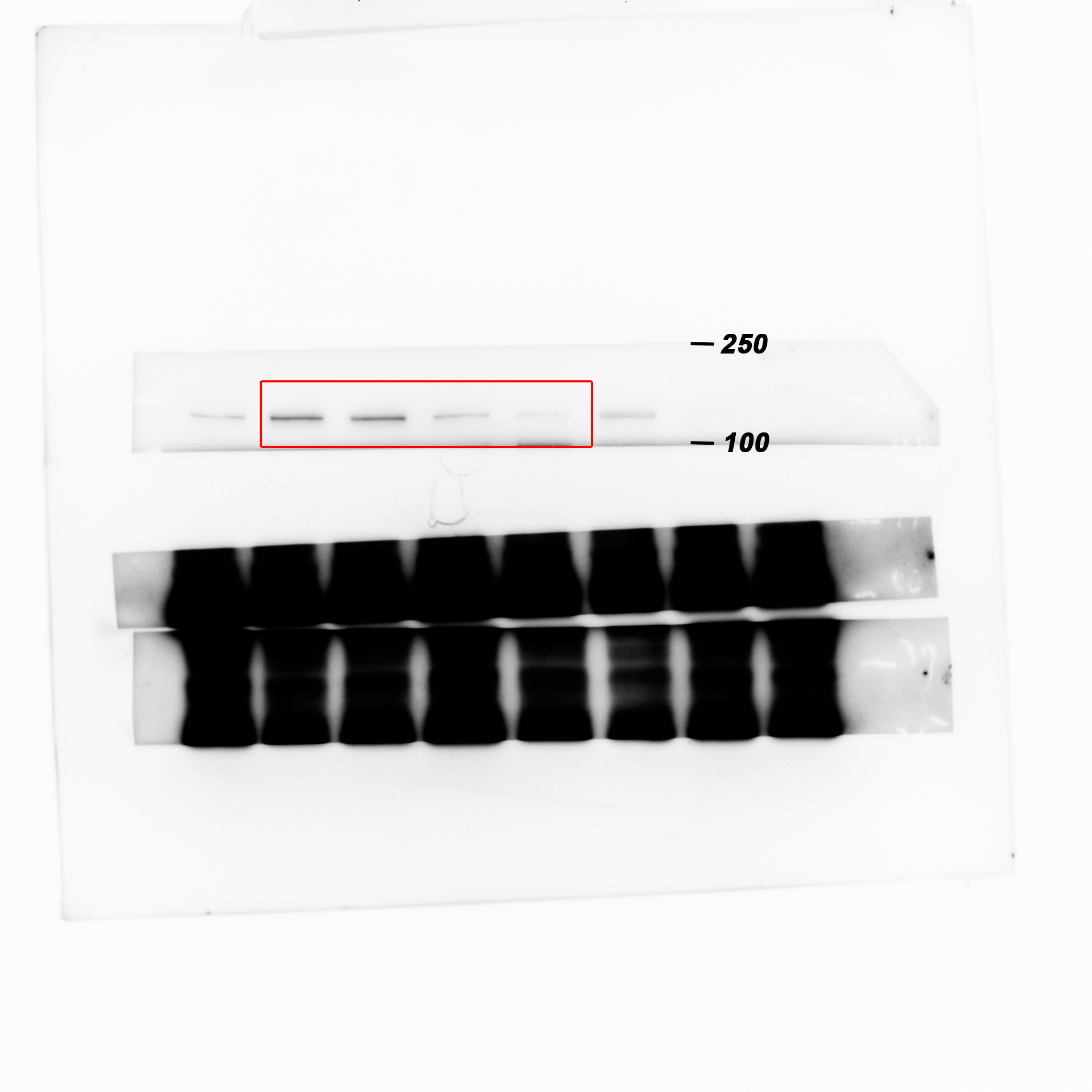

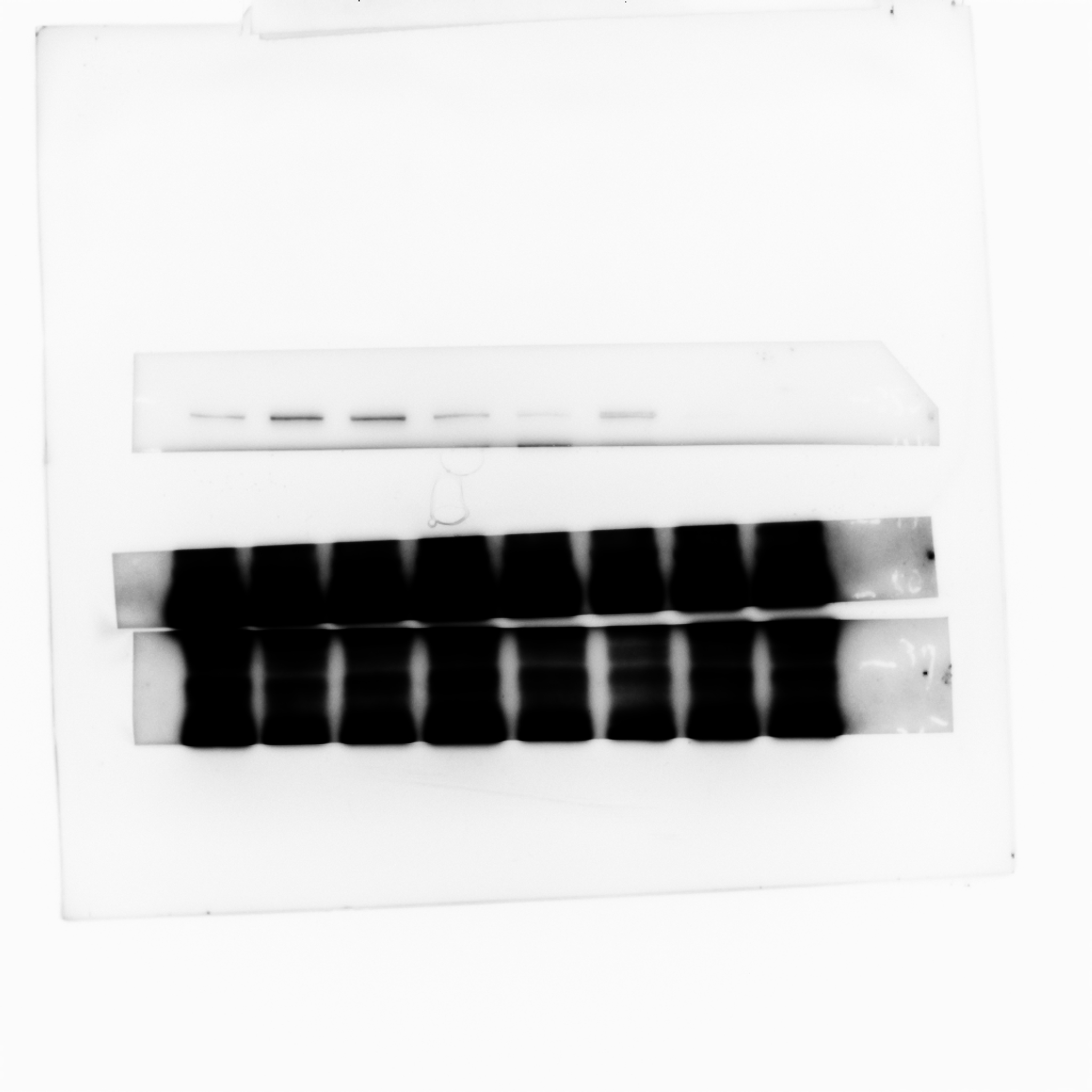
**

Western blot analysis of ZEB1 protein levels in whole cell lysate of 4T1 primary tumors from either vehicle or Pizotifen-treated mice.

**Figure 5C_ Phosphorylation of serine-9 in GSK3**β**_source data**

**
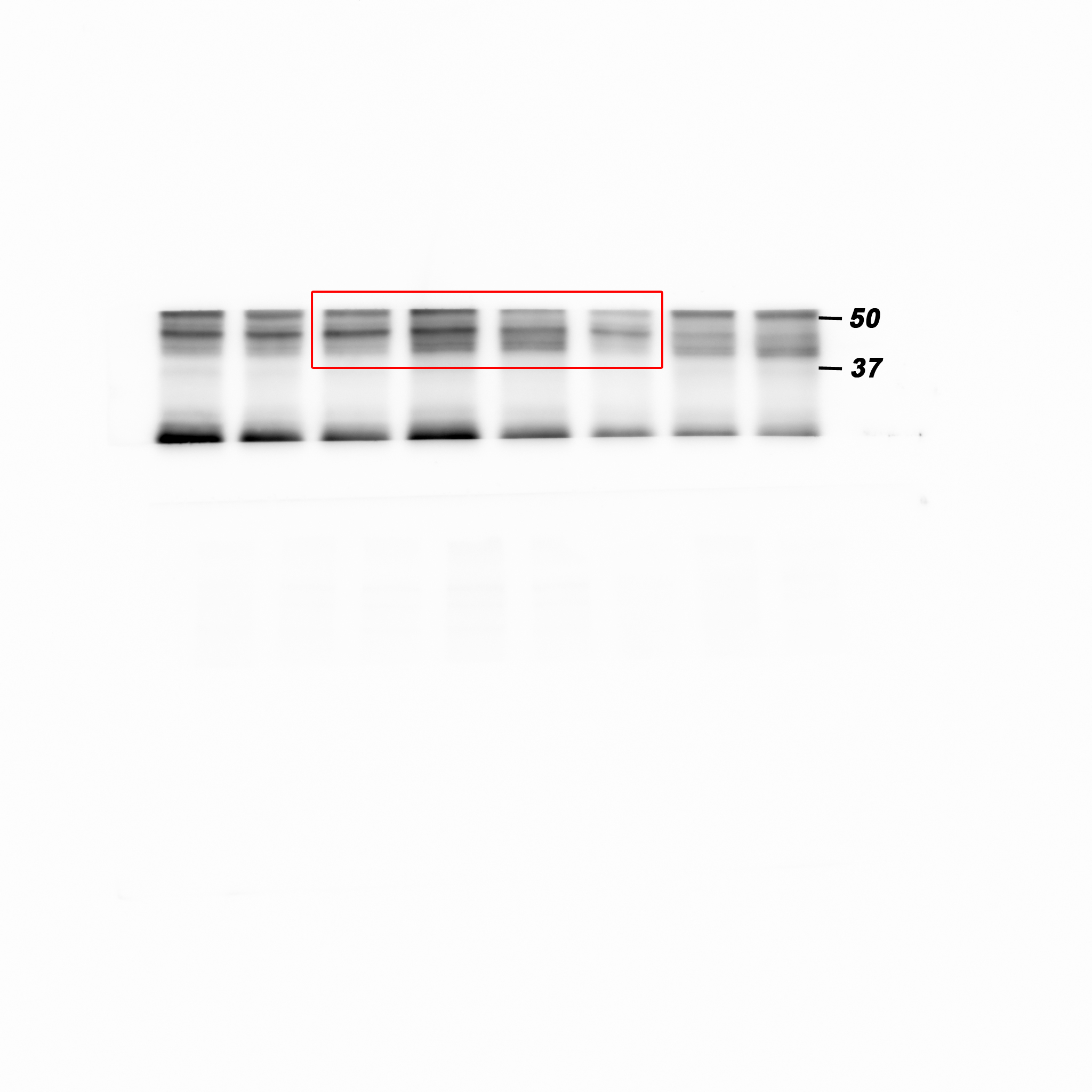

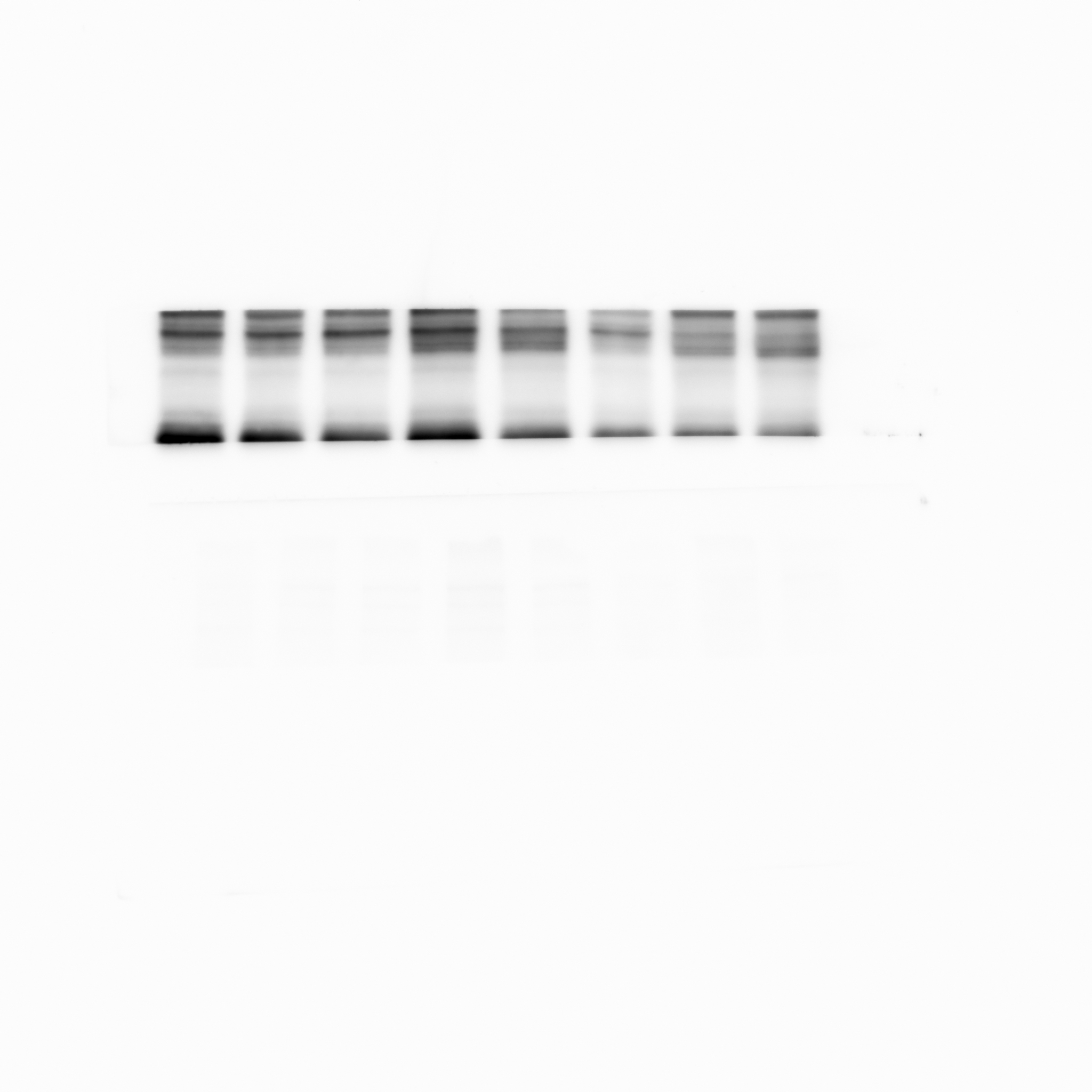
**

Western blot analysis of the protein levels of phosphorylation of serine-9 in GSK3β in whole cell lysate of 4T1 primary tumors from either vehicle or Pizotifen-treated mice.

**Figure 5C_ GSK3**β**_source data**

**
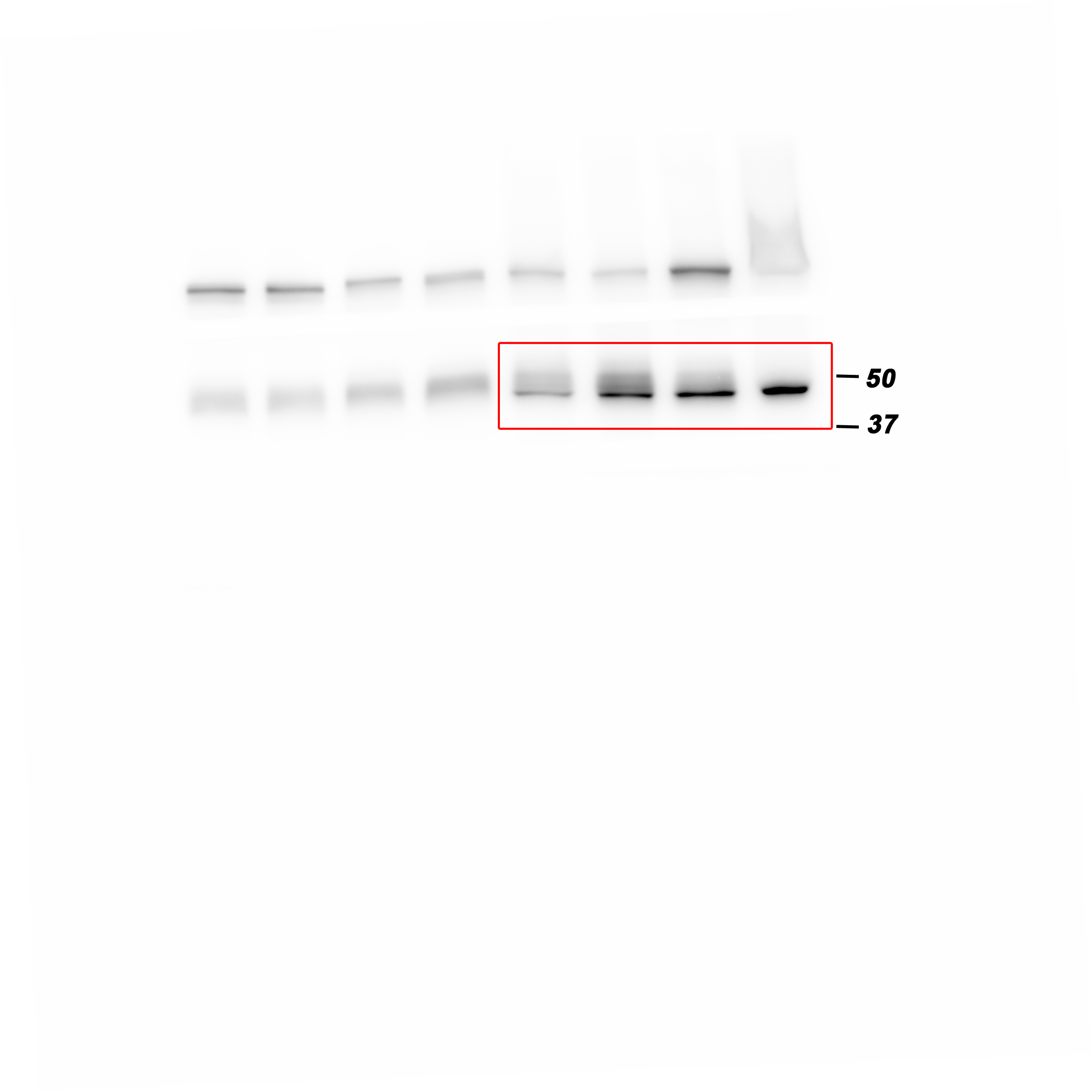

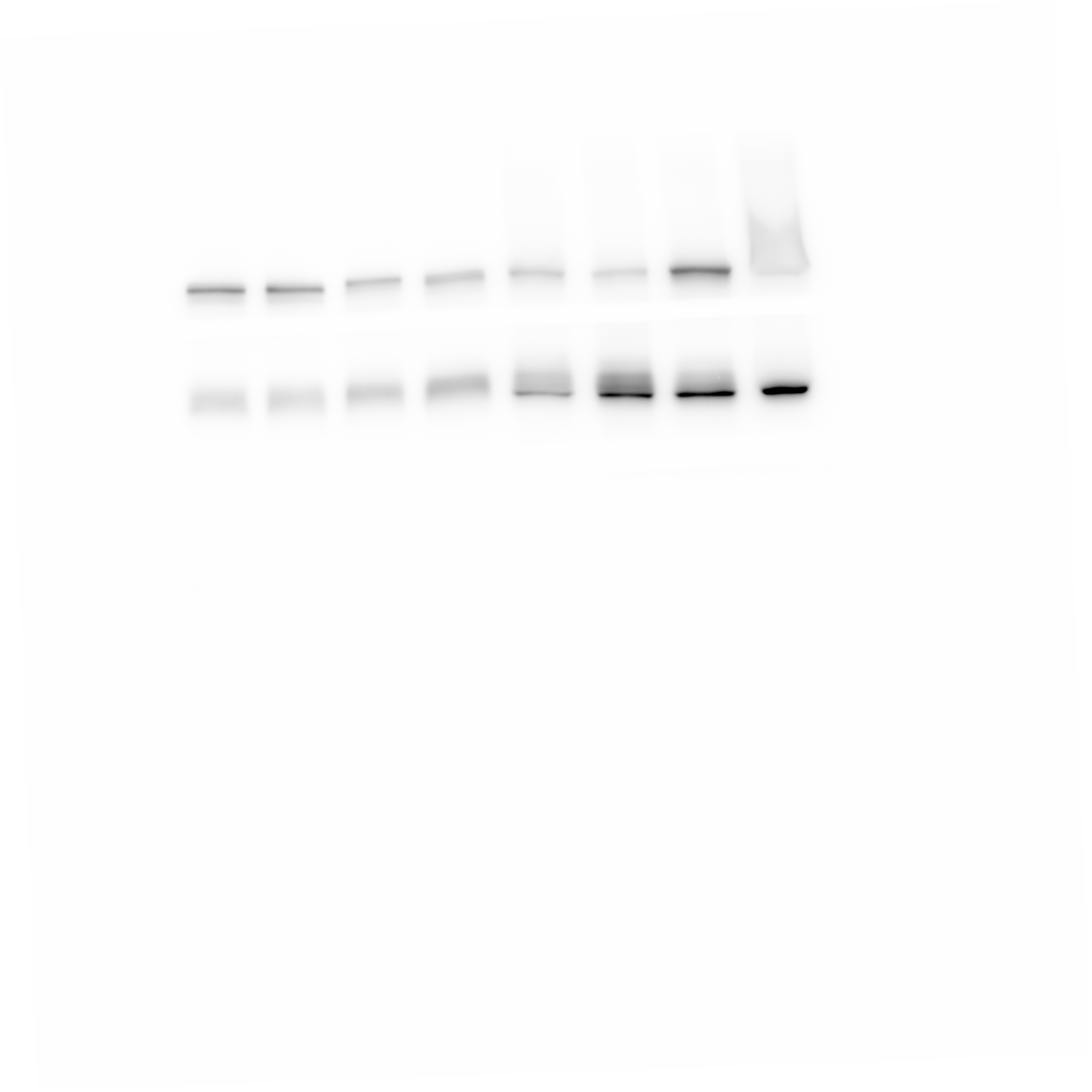
**

Western blot analysis of GSK3β protein levels in whole cell lysate of 4T1 primary tumors from either vehicle or Pizotifen-treated mice.

**Figure 5C_Luciferase_source data**

**
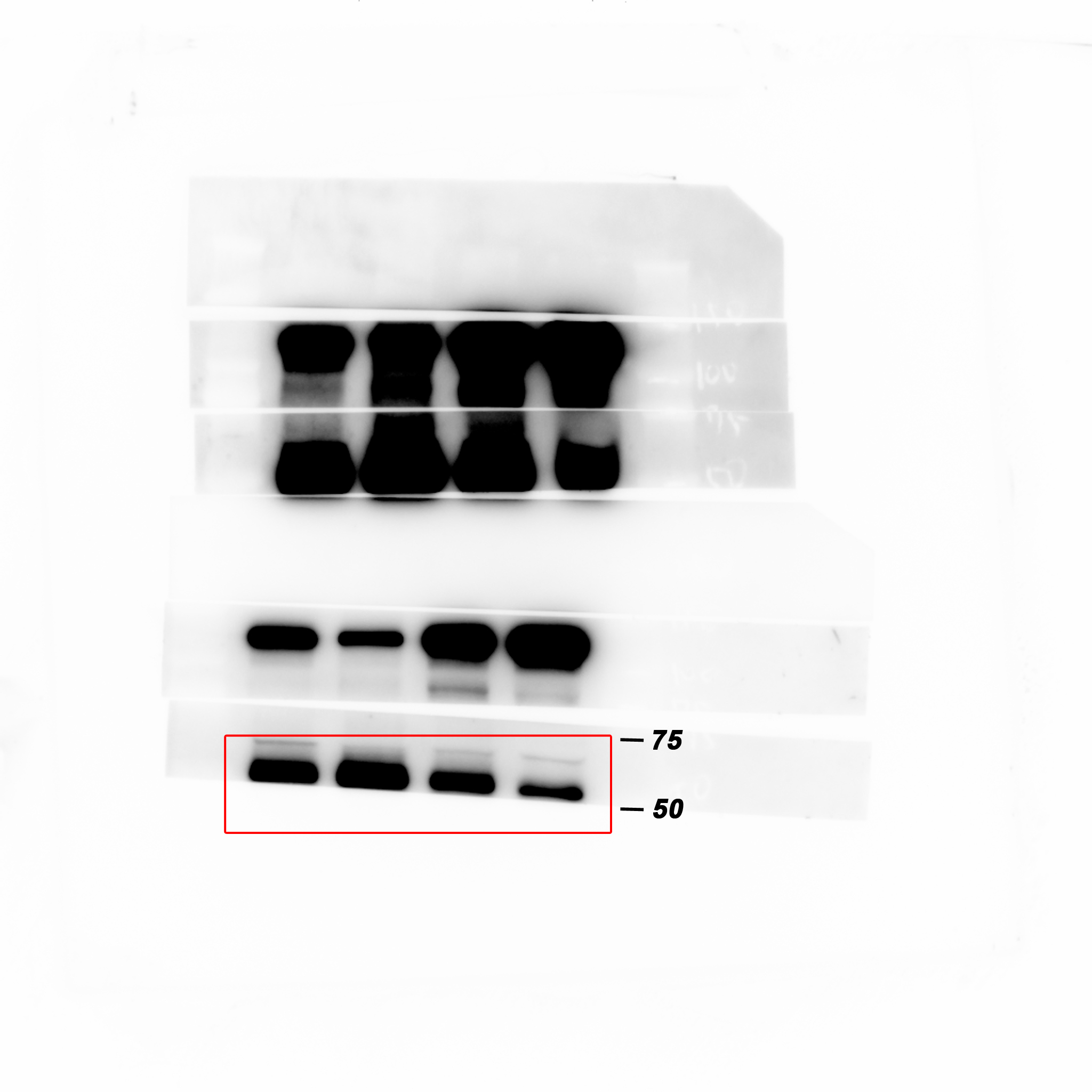

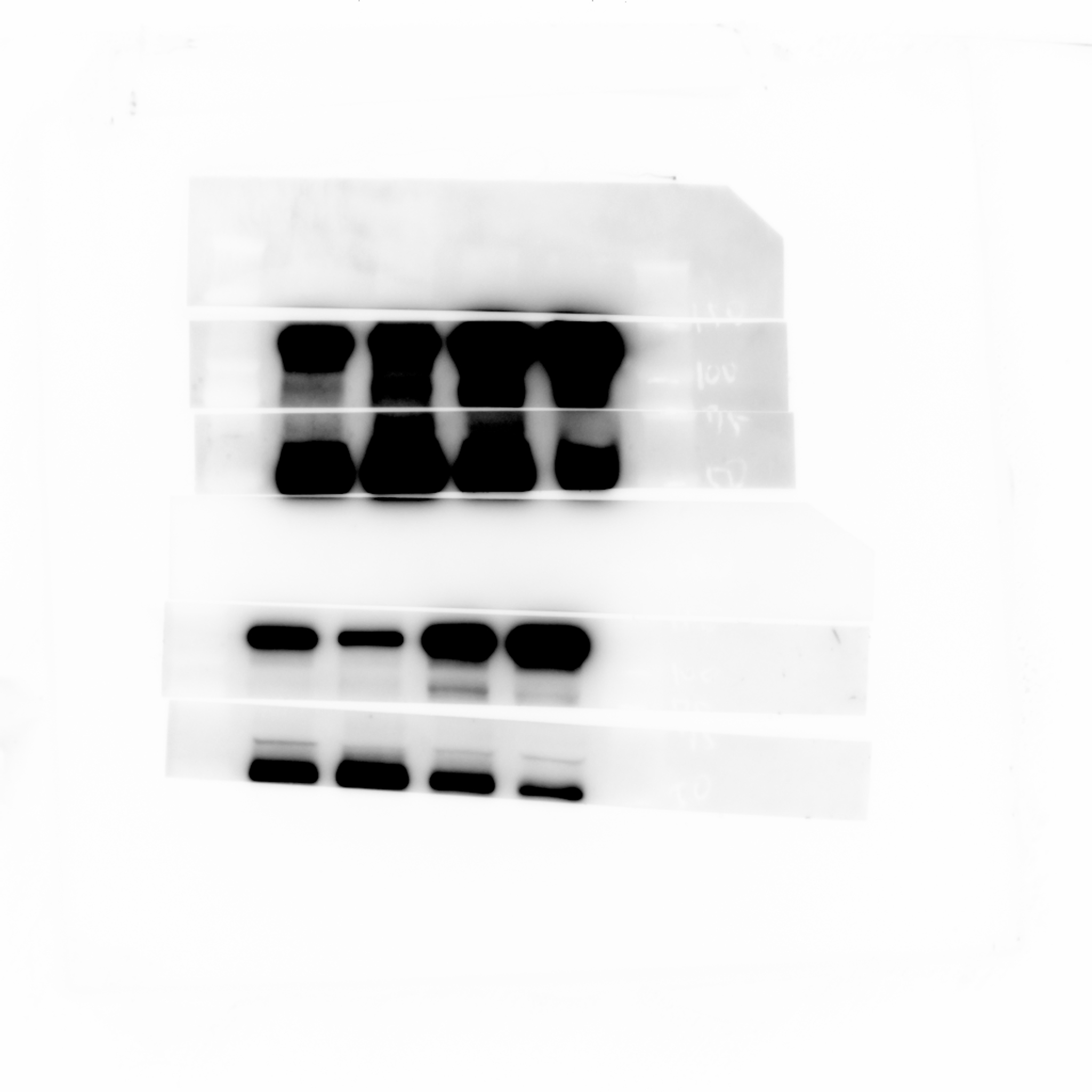
**

Western blot analysis of Luciferase protein levels in whole cell lysate of 4T1 primary tumors from either vehicle or Pizotifen-treated mice.

**Figure 5C_**β**-catenin in the nucleus_source data**

**
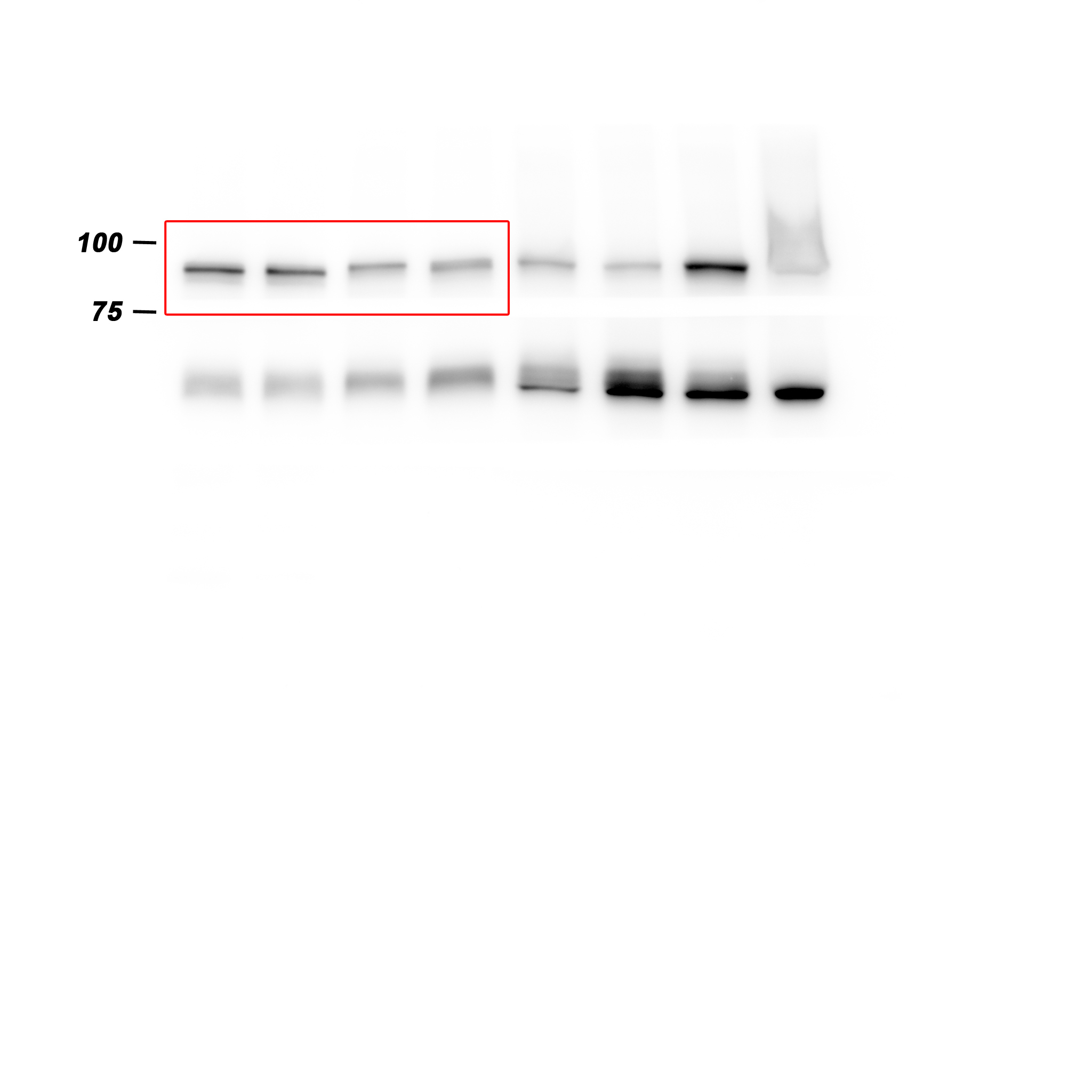

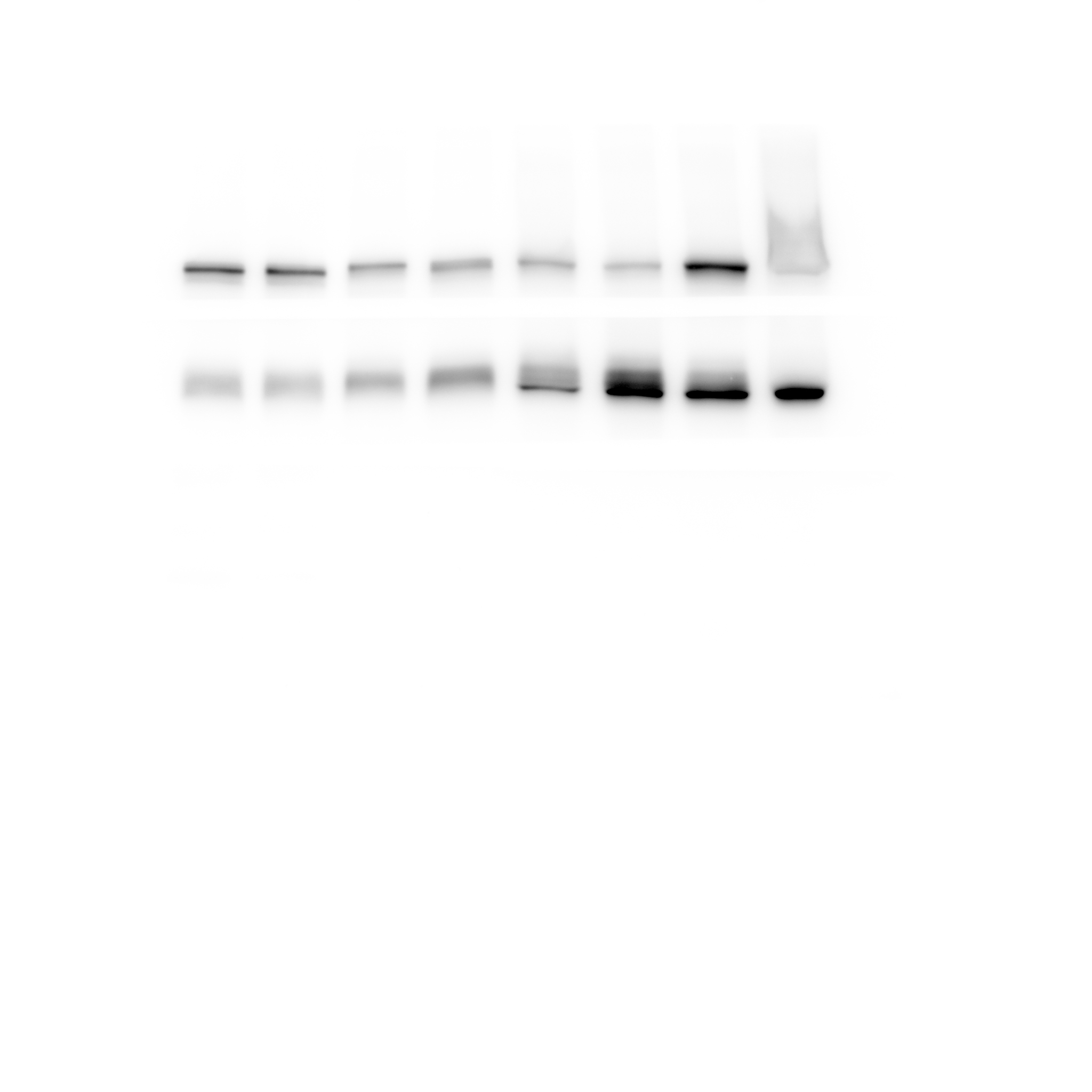
**

Western blot analysis of β-catenin protein levels in the nucleus of 4T1 primary tumors from either vehicle or Pizotifen-treated mice.

**Figure 5C**_**Histone H3** **in the nucleus_source data**

**
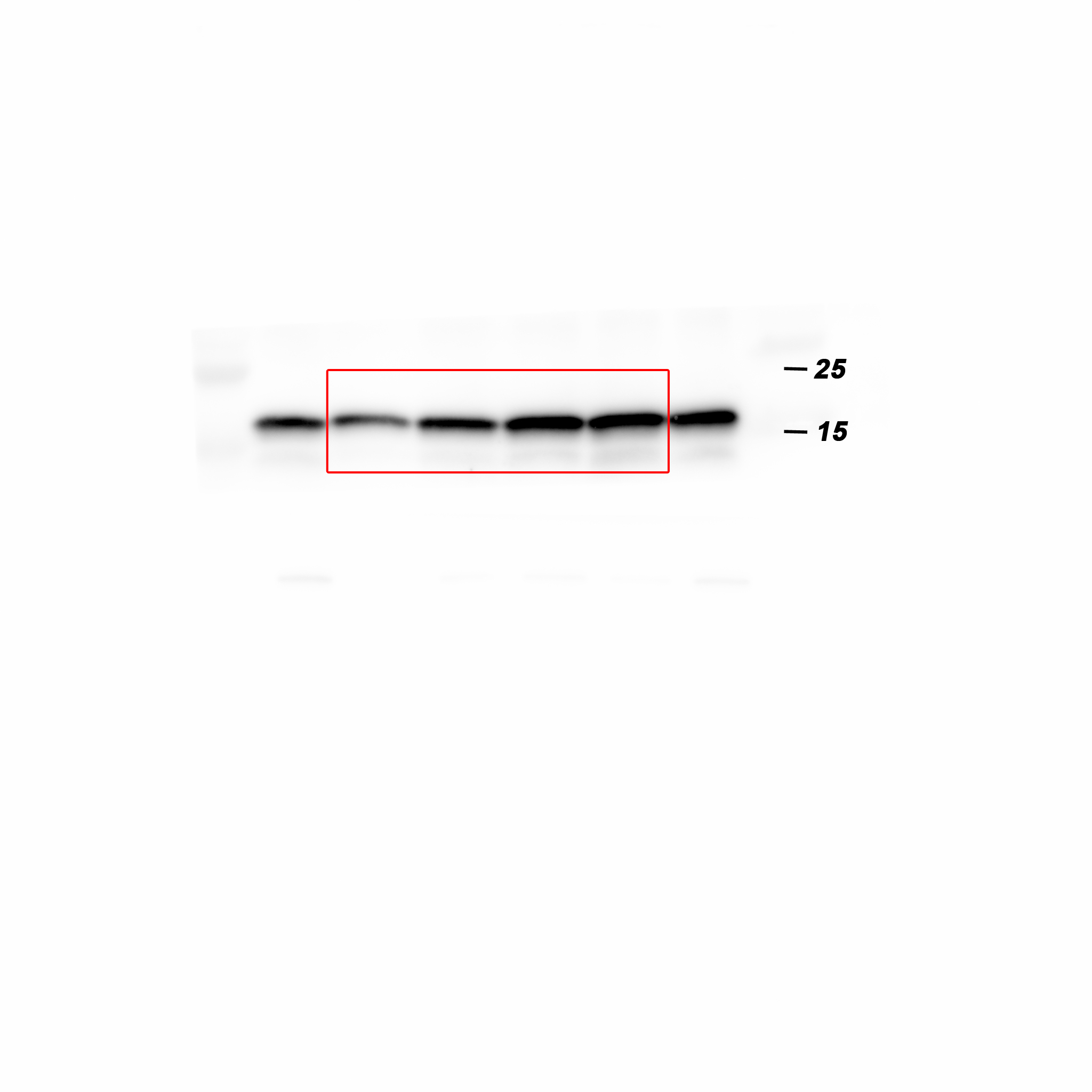

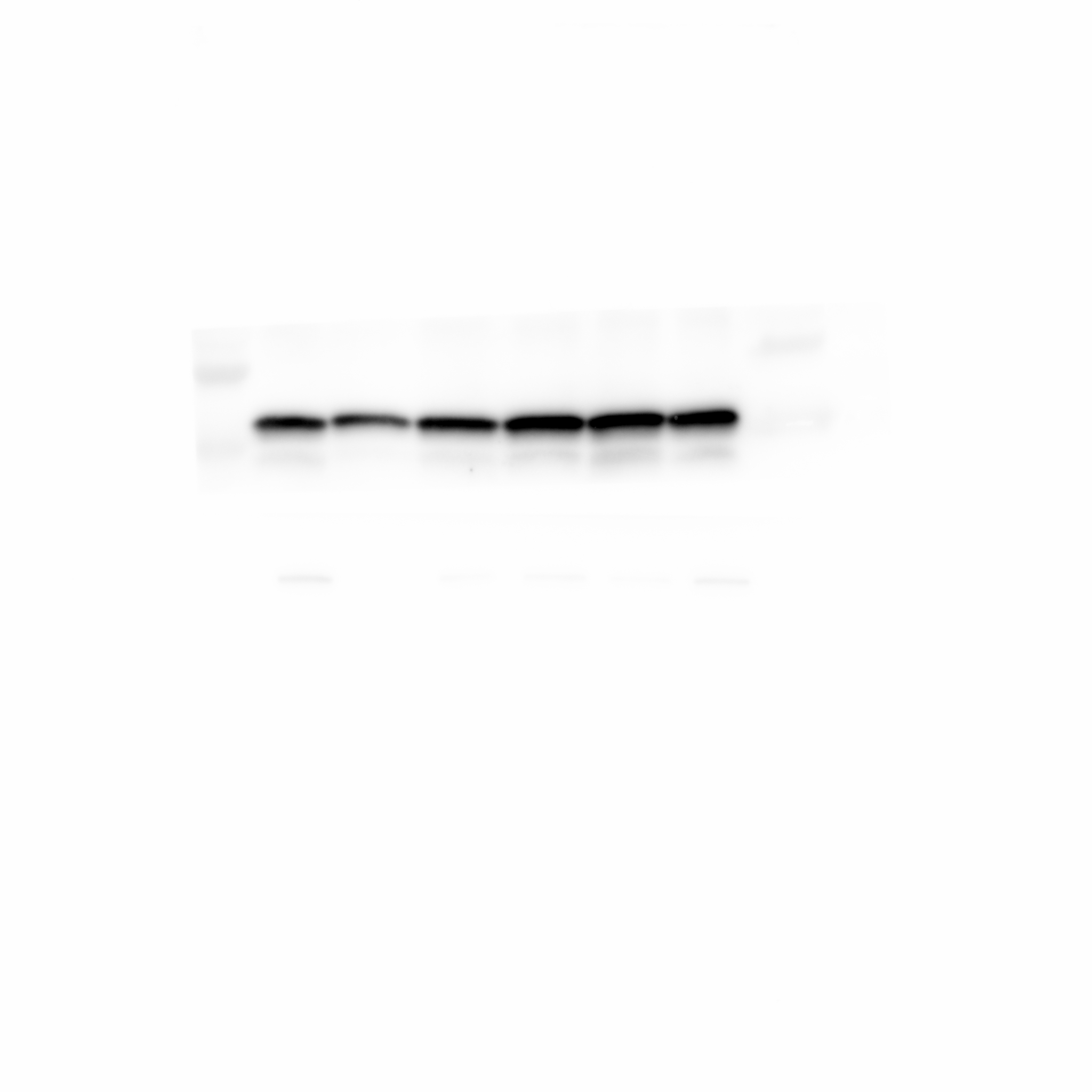
**

Western blot analysis of Histone H3 protein levels in the nucleus of 4T1 primary tumors from either vehicle or Pizotifen-treated mice.

**Figure 5C_**β**-catenin in the cytoplasm_source data**


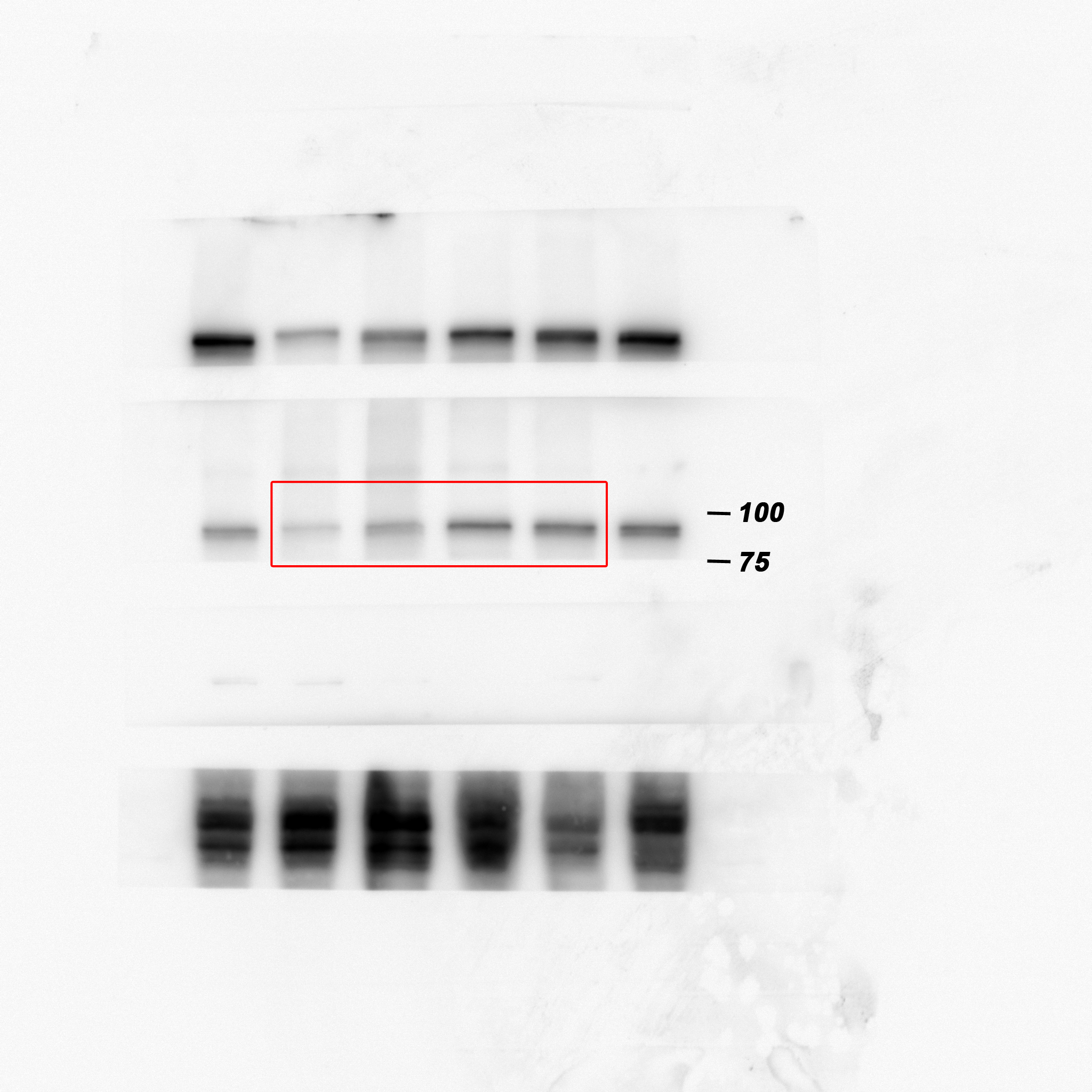

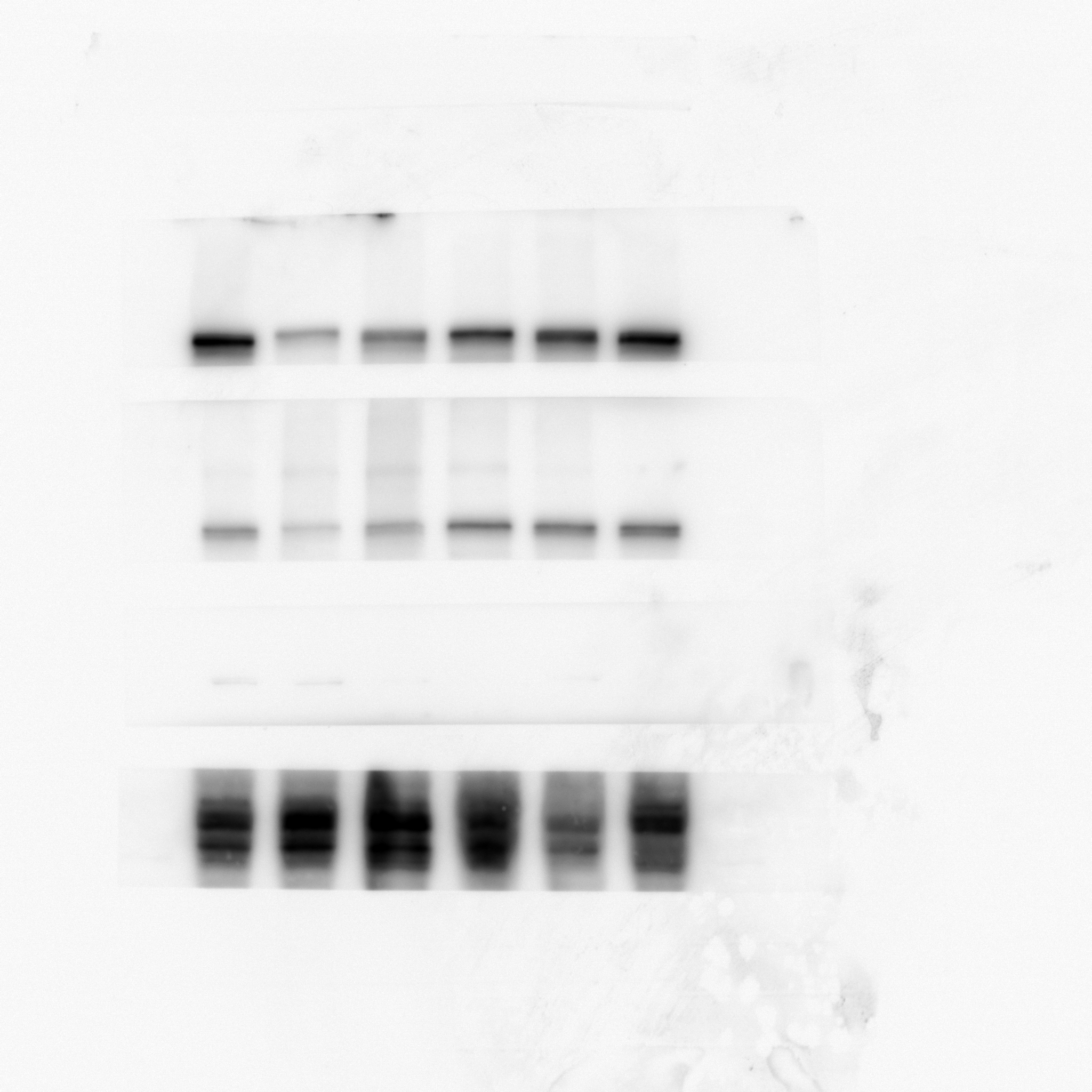


Western blot analysis of β-catenin protein levels in the cytoplasm of 4T1 primary tumors from either vehicle or Pizotifen-treated mice.

**Figure 5C_**β**-tubulin in the cytoplasm_source data**

**
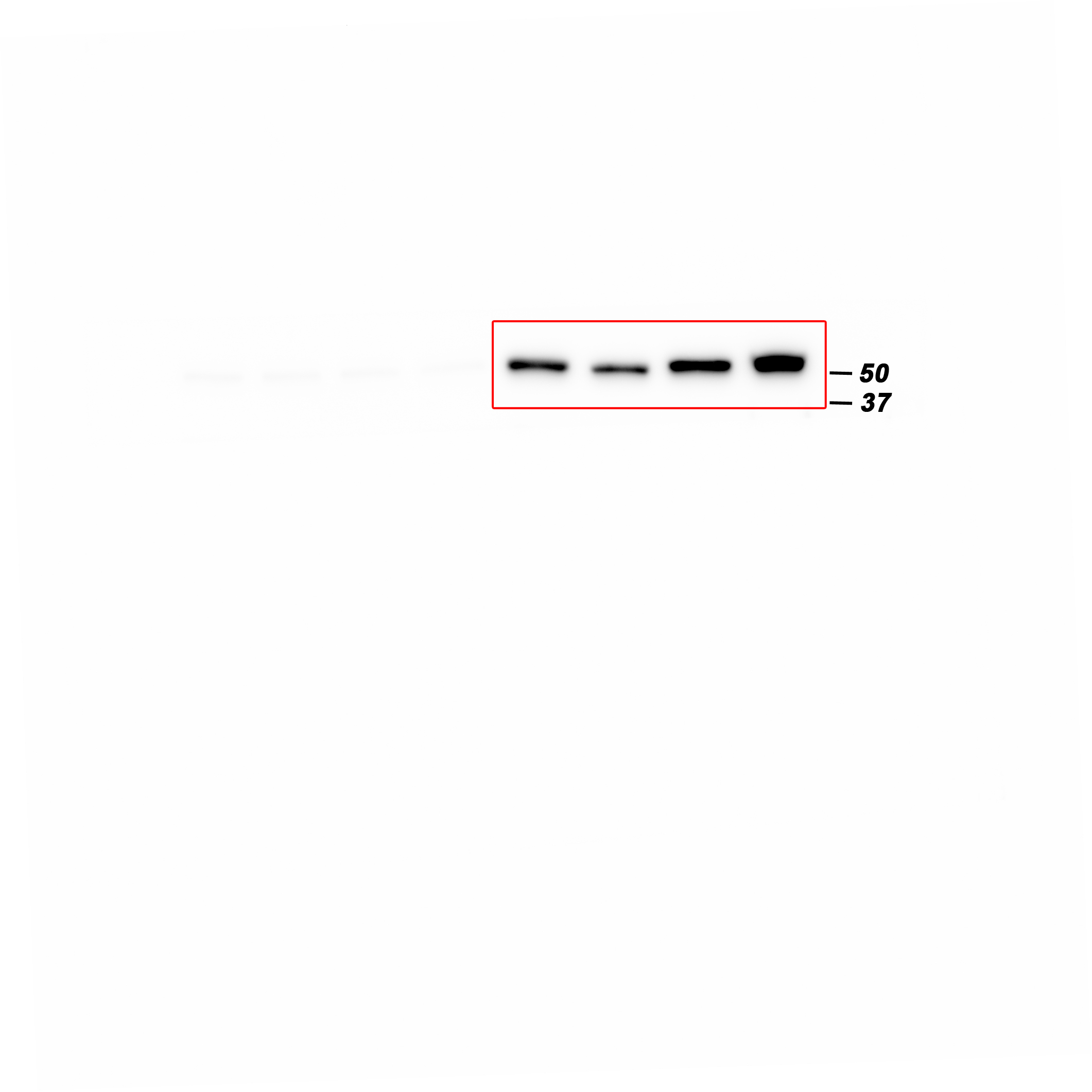

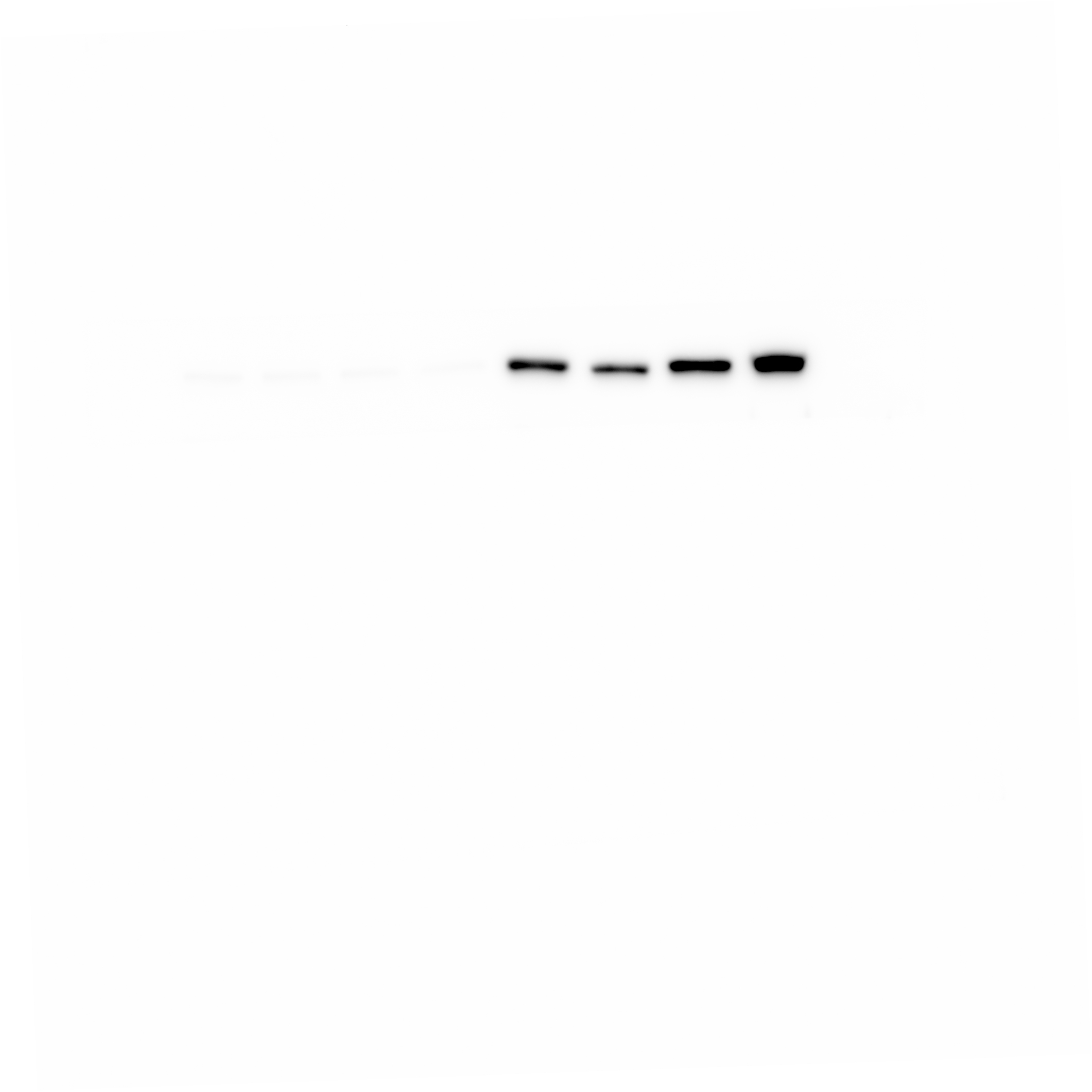
**

Western blot analysis of β-tubulin protein levels in the cytoplasm of 4T1 primary tumors from either vehicle or Pizotifen-treated mice.

**Figure 5D_E-cadherin_source data**

**
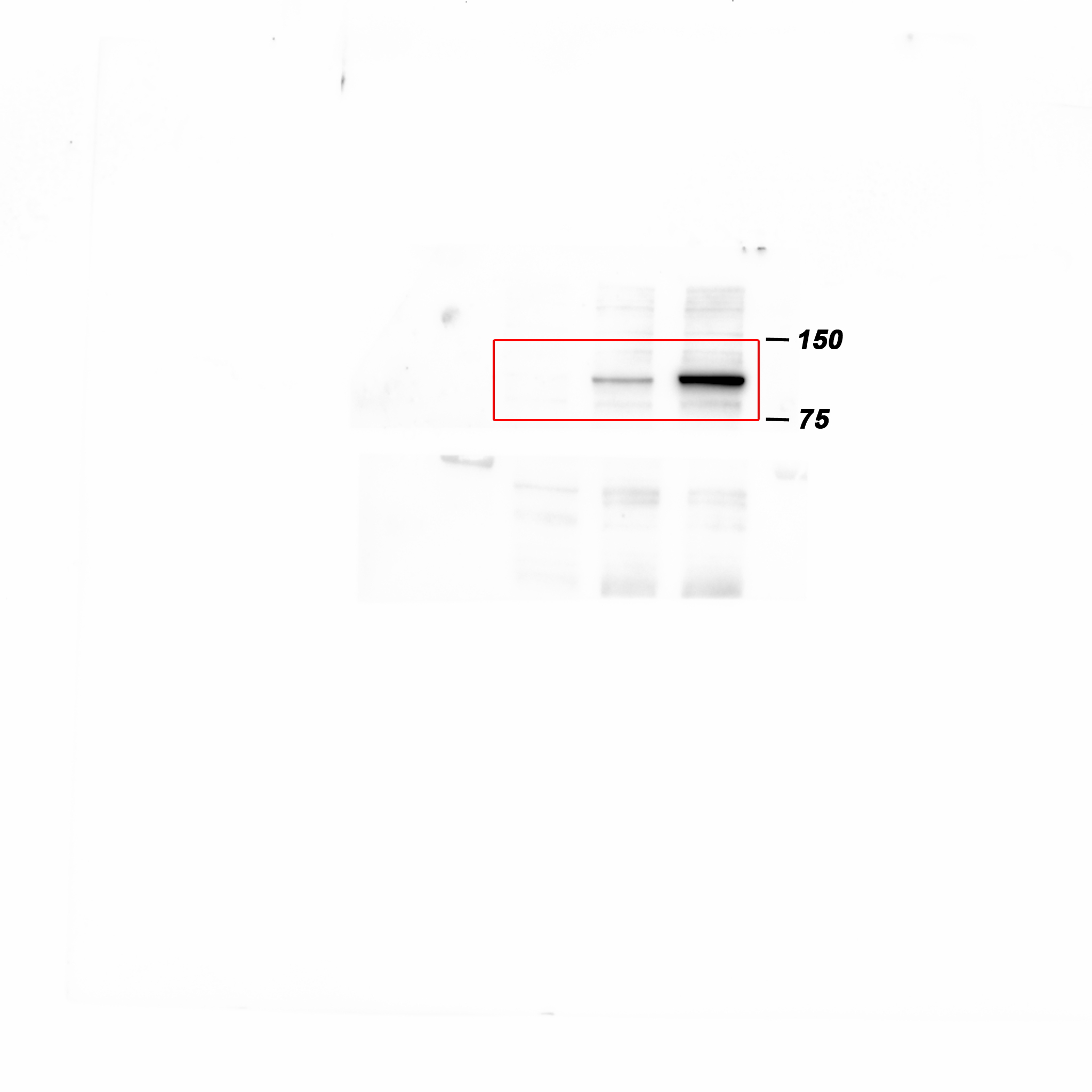

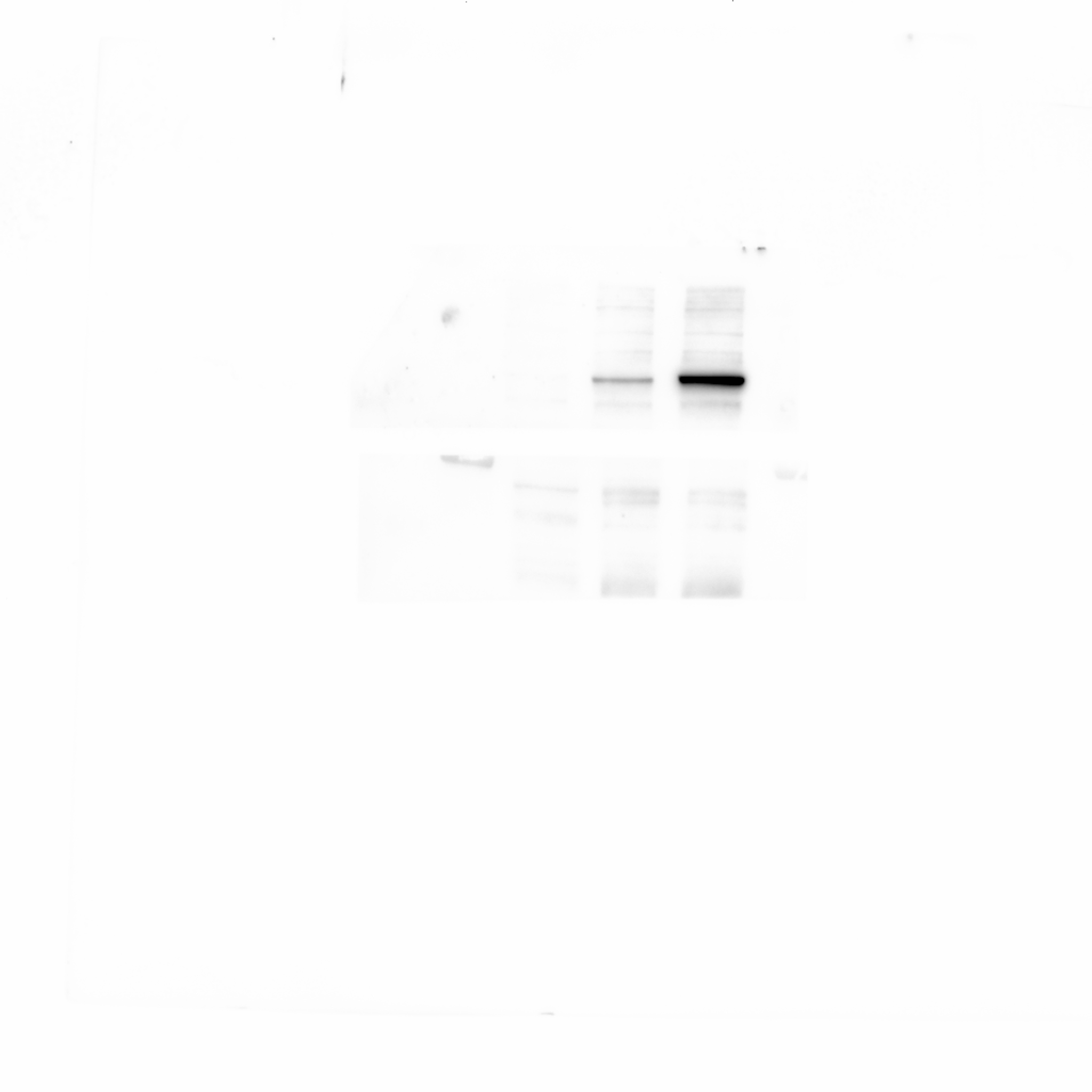
**

Western blot analysis of E-cadherin protein levels in either vehicle or Pizotifen-treated MDA-MB-231 cells or E-cadherin positive cells in Pizotifen-treated MDA-MB-231 cells.

**Figure 5D_EpCAM_source data**

**
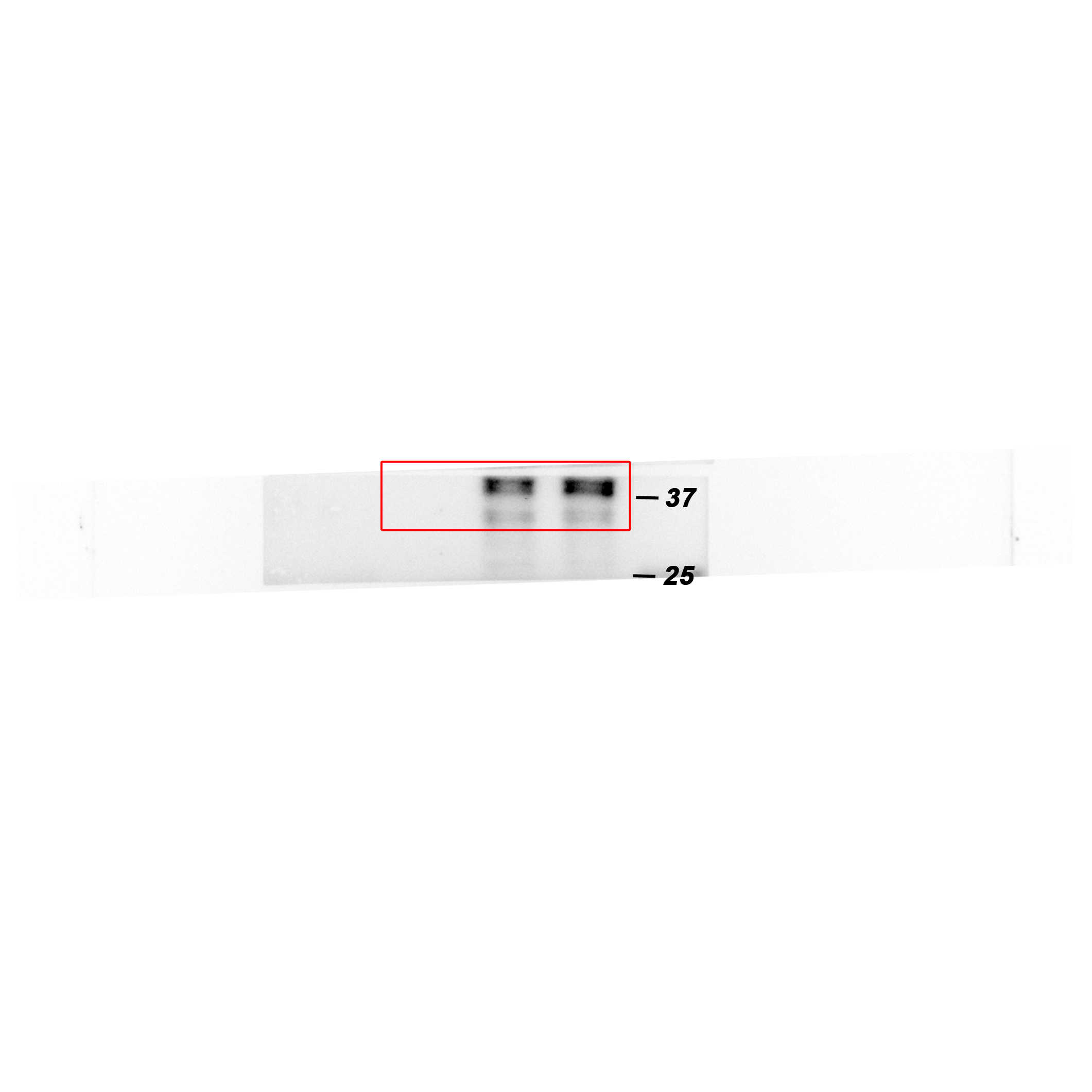

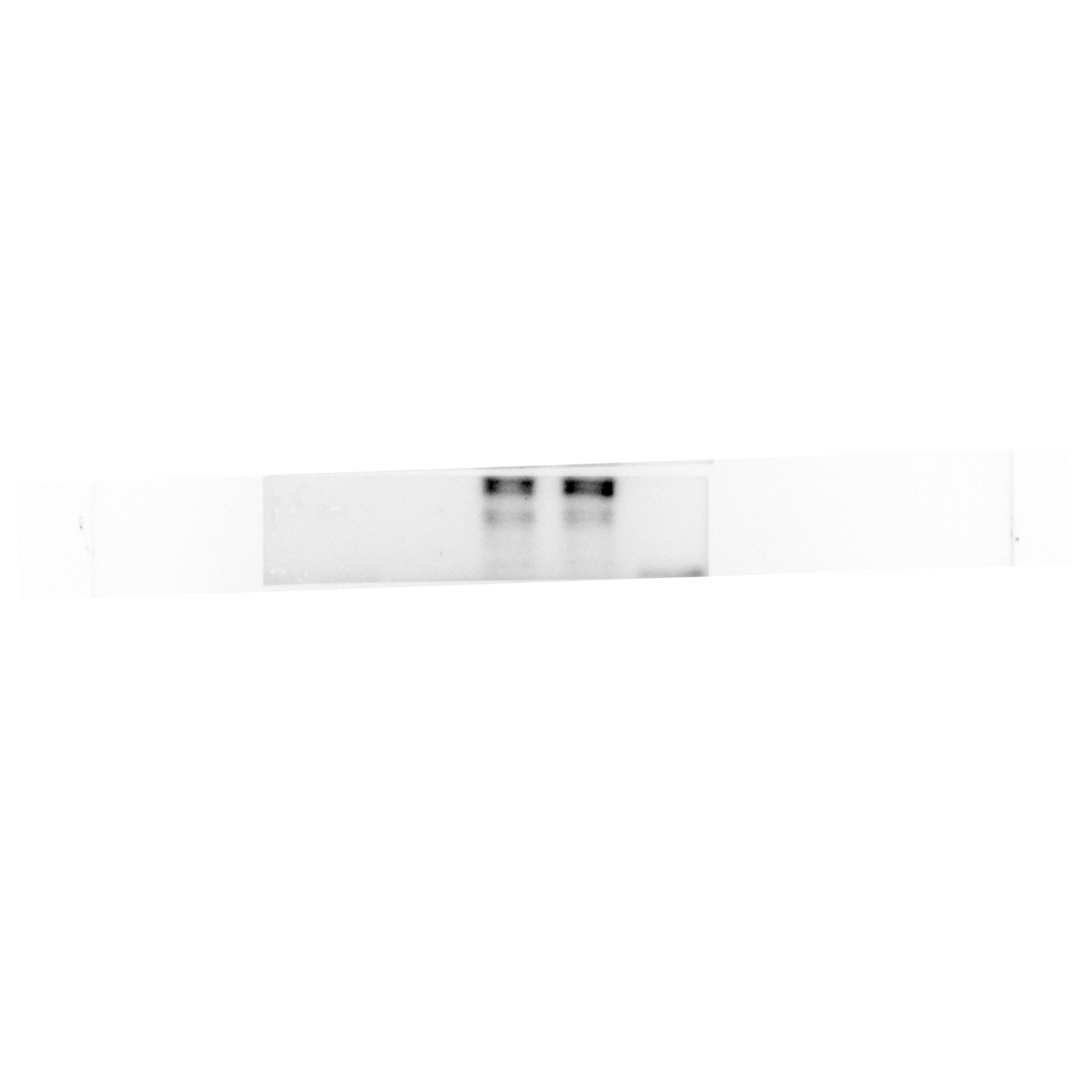
**

Western blot analysis of EpCAM protein levels in either vehicle or Pizotifen-treated MDA-MB-231 cells or E-cadherin positive cells in Pizotifen-treated MDA-MB-231 cells.

**Figure 5D_Keratin18 (KRT18)_source data**

**
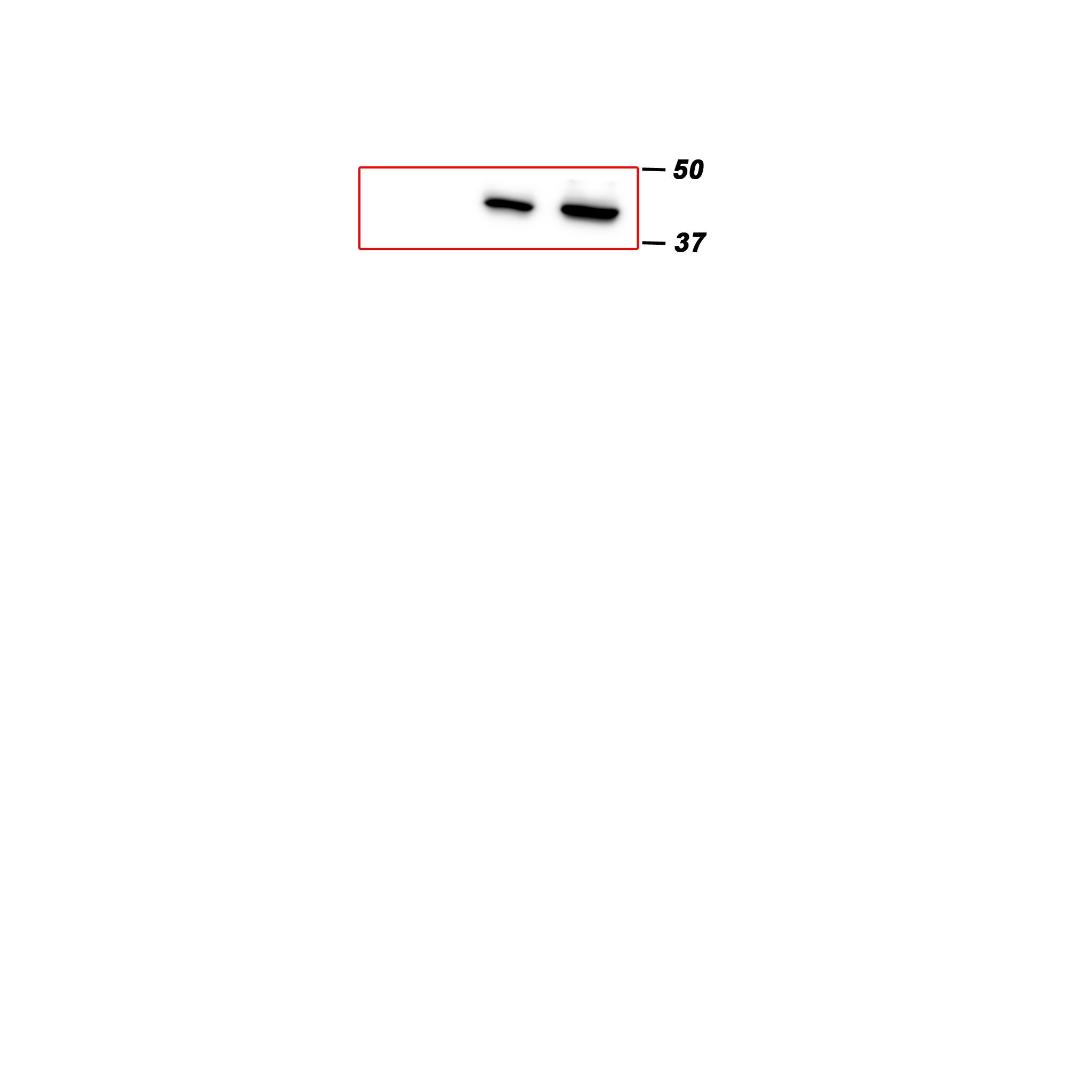

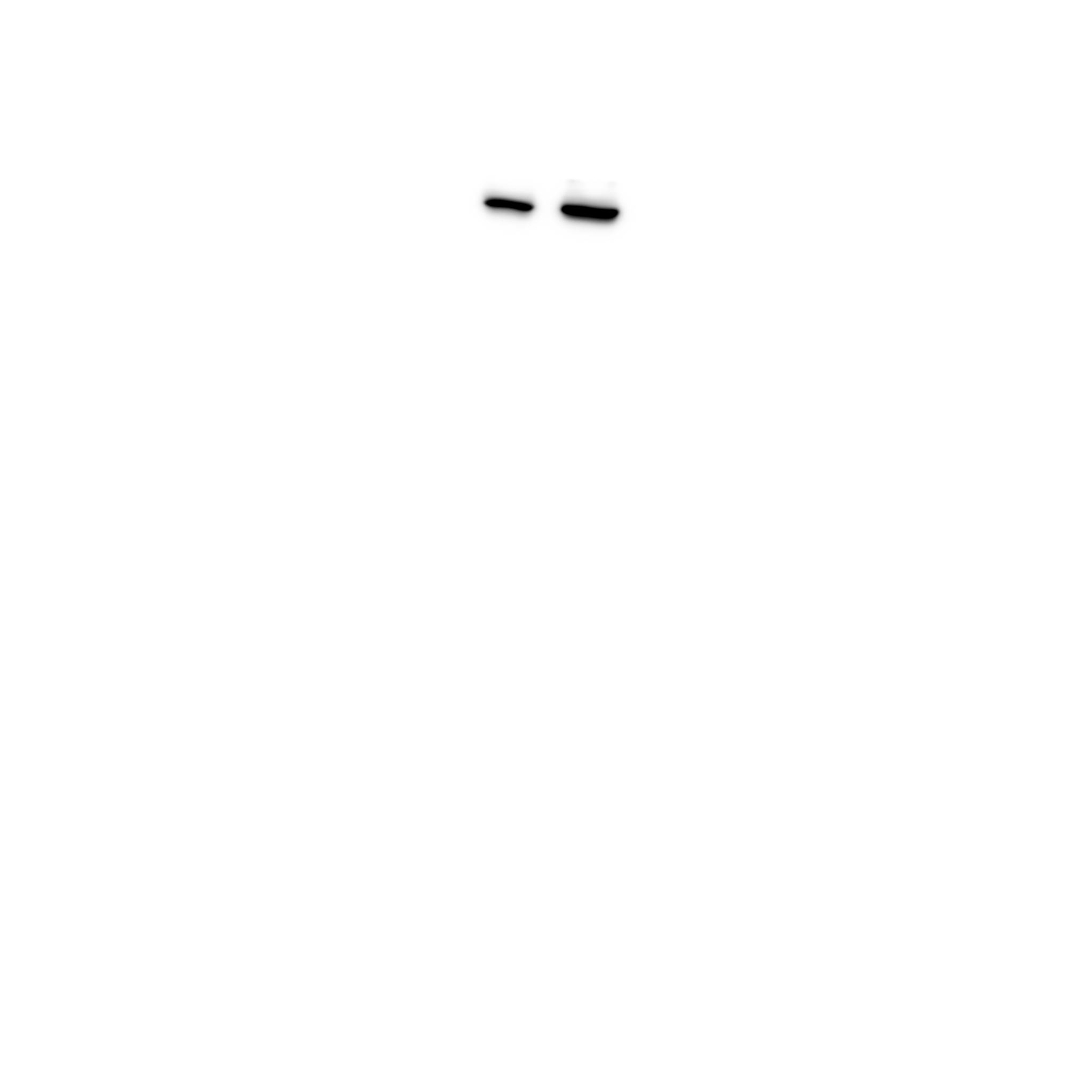
**

Western blot analysis of KRT18 protein levels in either vehicle or Pizotifen-treated MDA-MB-231 cells or E-cadherin positive cells in Pizotifen-treated MDA-MB-231 cells.

**Figure 5D_Keratin19 (KRT19)_source data**

**
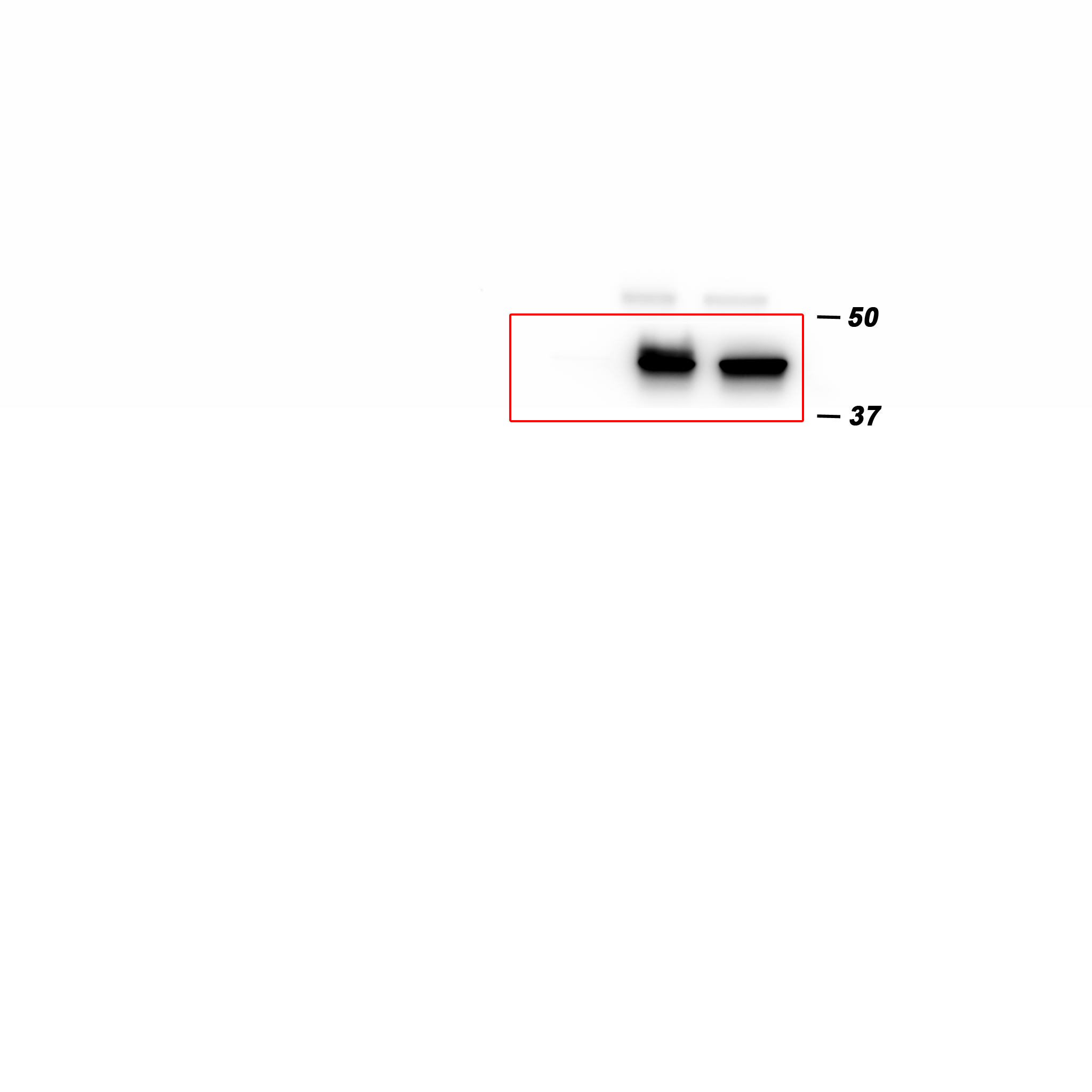

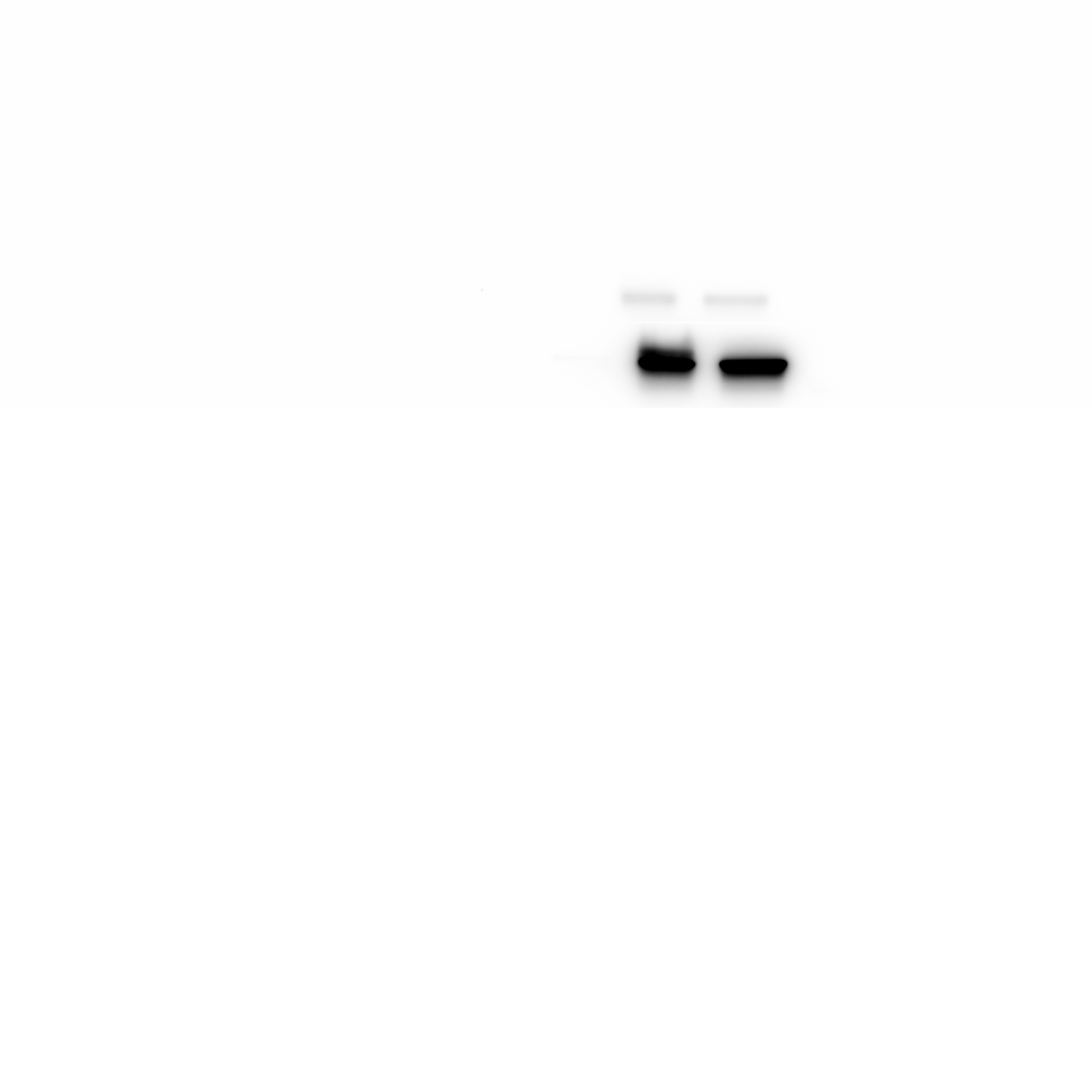
**

Western blot analysis of KRT19 protein levels in either vehicle or Pizotifen-treated MDA-MB-231 cells or E-cadherin positive cells in Pizotifen-treated MDA-MB-231 cells.

**Figure 5D_Vimentin_source data**

**
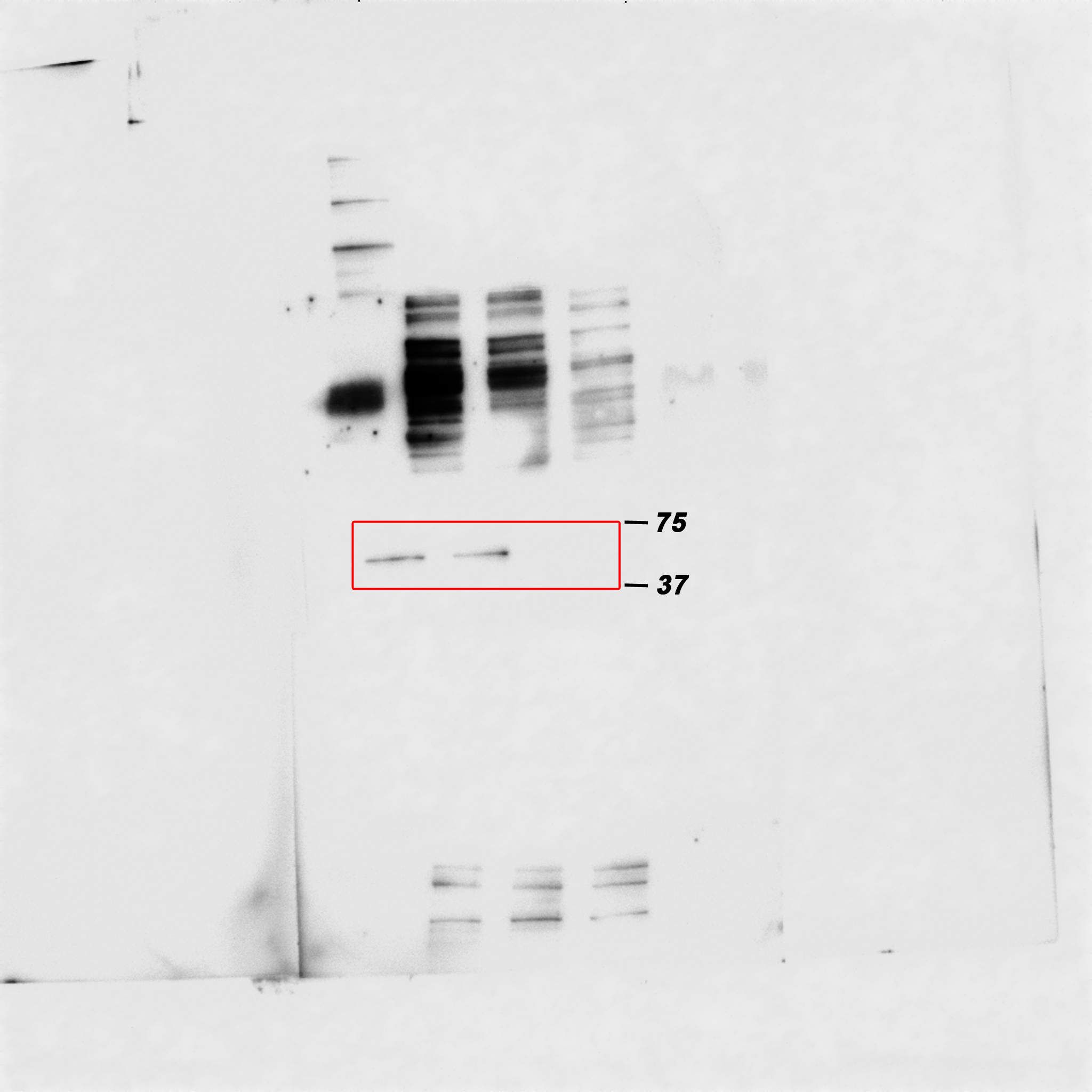

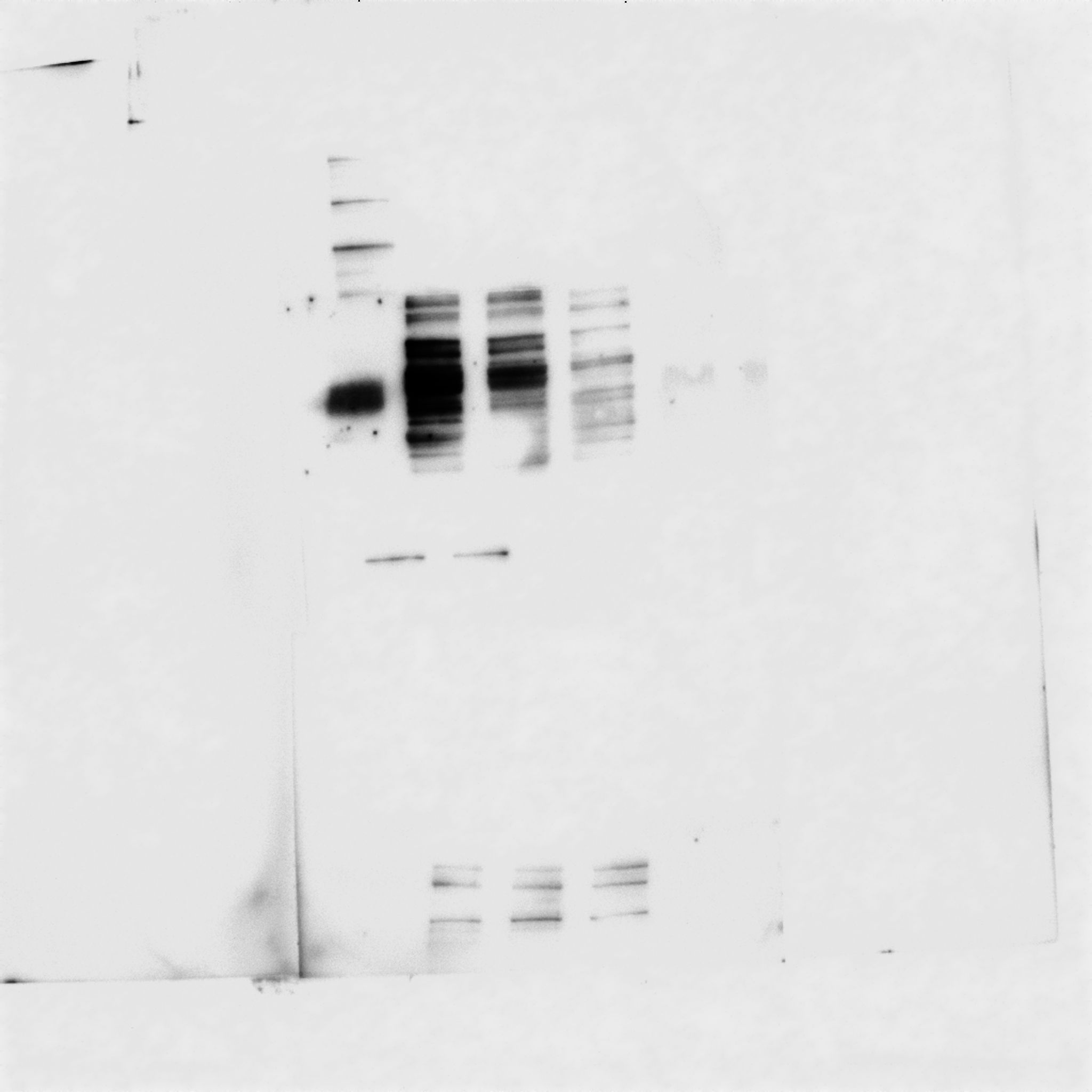
**

Western blot analysis of Vimentin protein levels in either vehicle or Pizotifen-treated MDA-MB-231 cells or E-cadherin positive cells in Pizotifen-treated MDA-MB-231 cells.

**Figure 5D_MMP1_source data

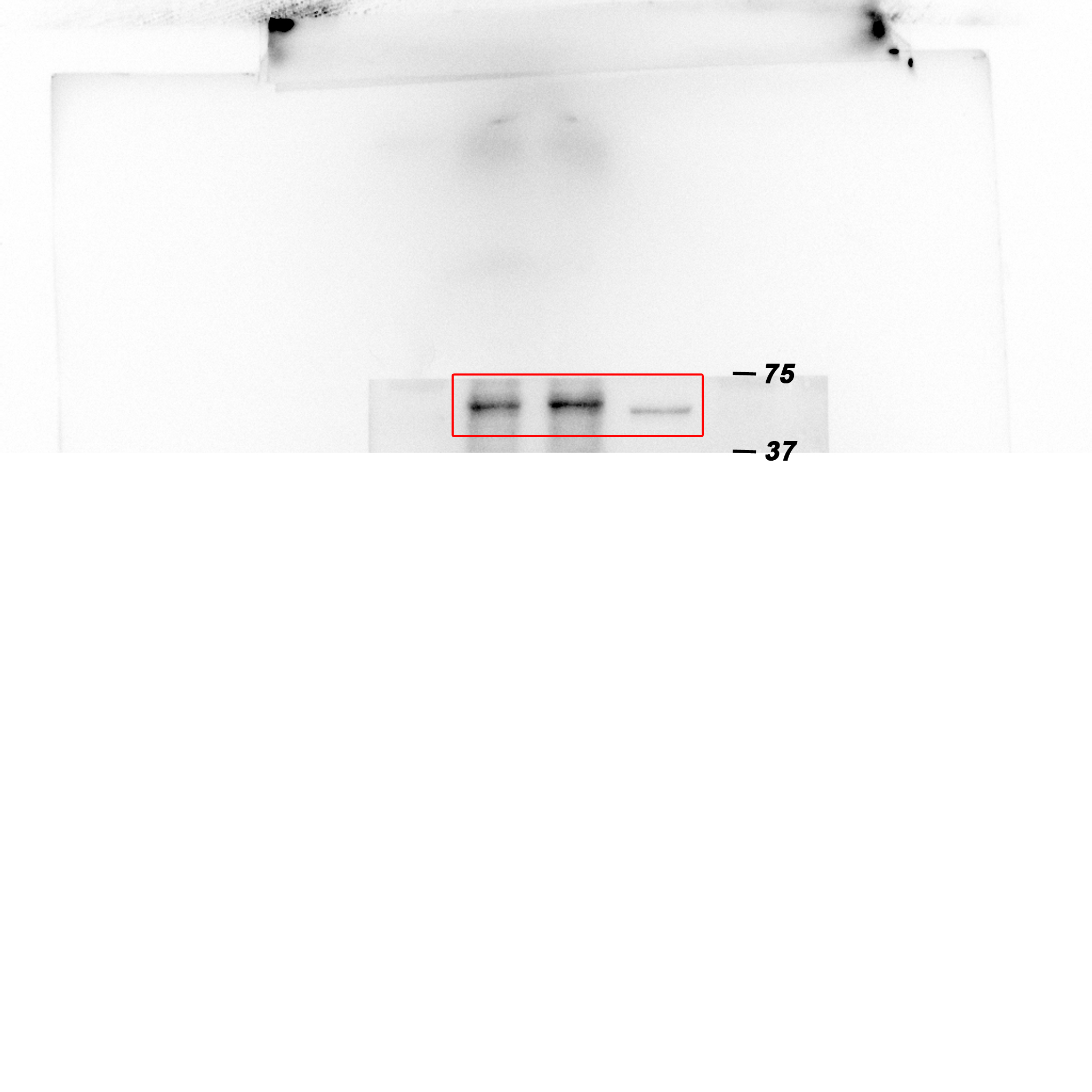

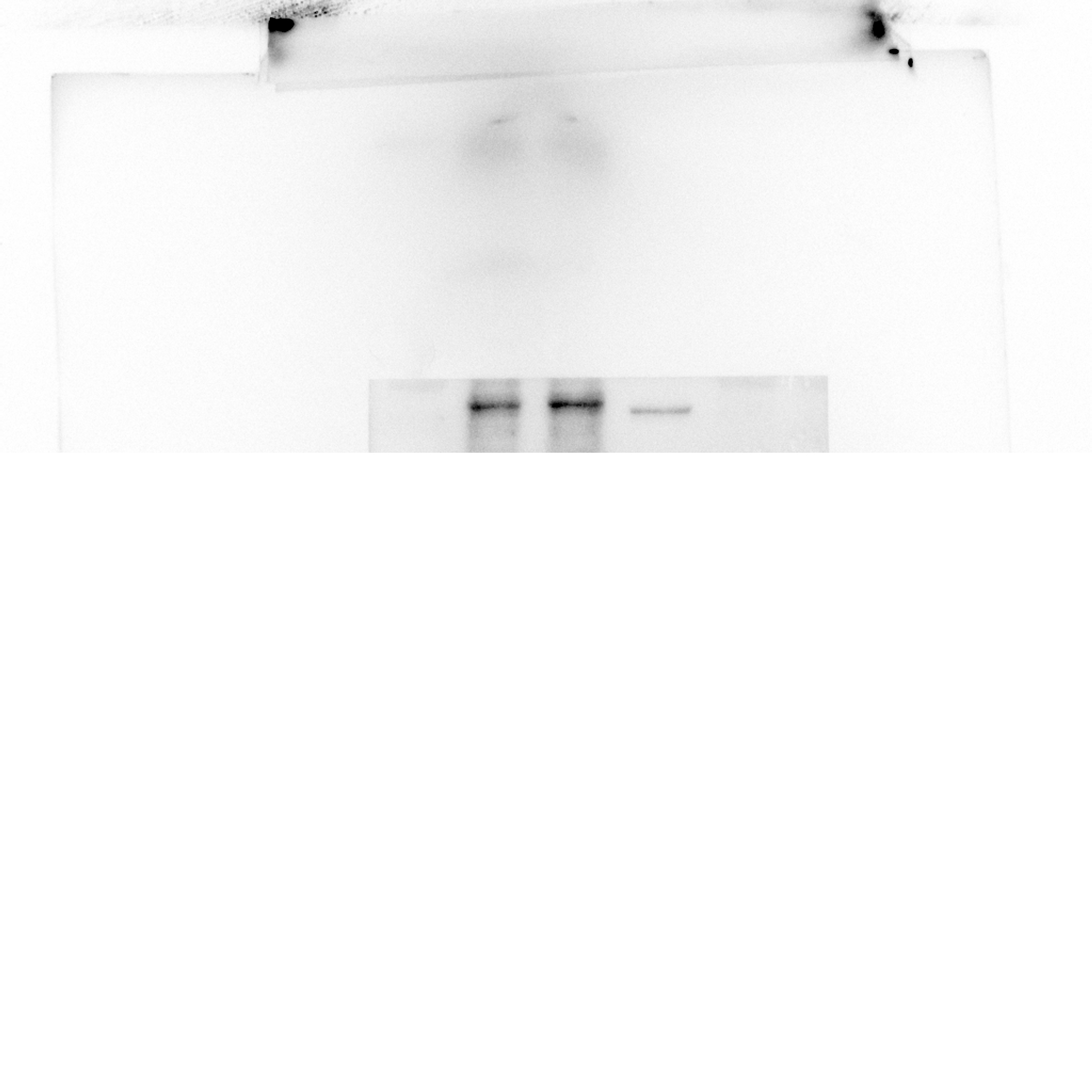
**

Western blot analysis of MMP1 protein levels in either vehicle or Pizotifen-treated MDA-MB-231 cells or E-cadherin positive cells in Pizotifen-treated MDA-MB-231 cells.

**Figure 5D_MMP3_source data**

**
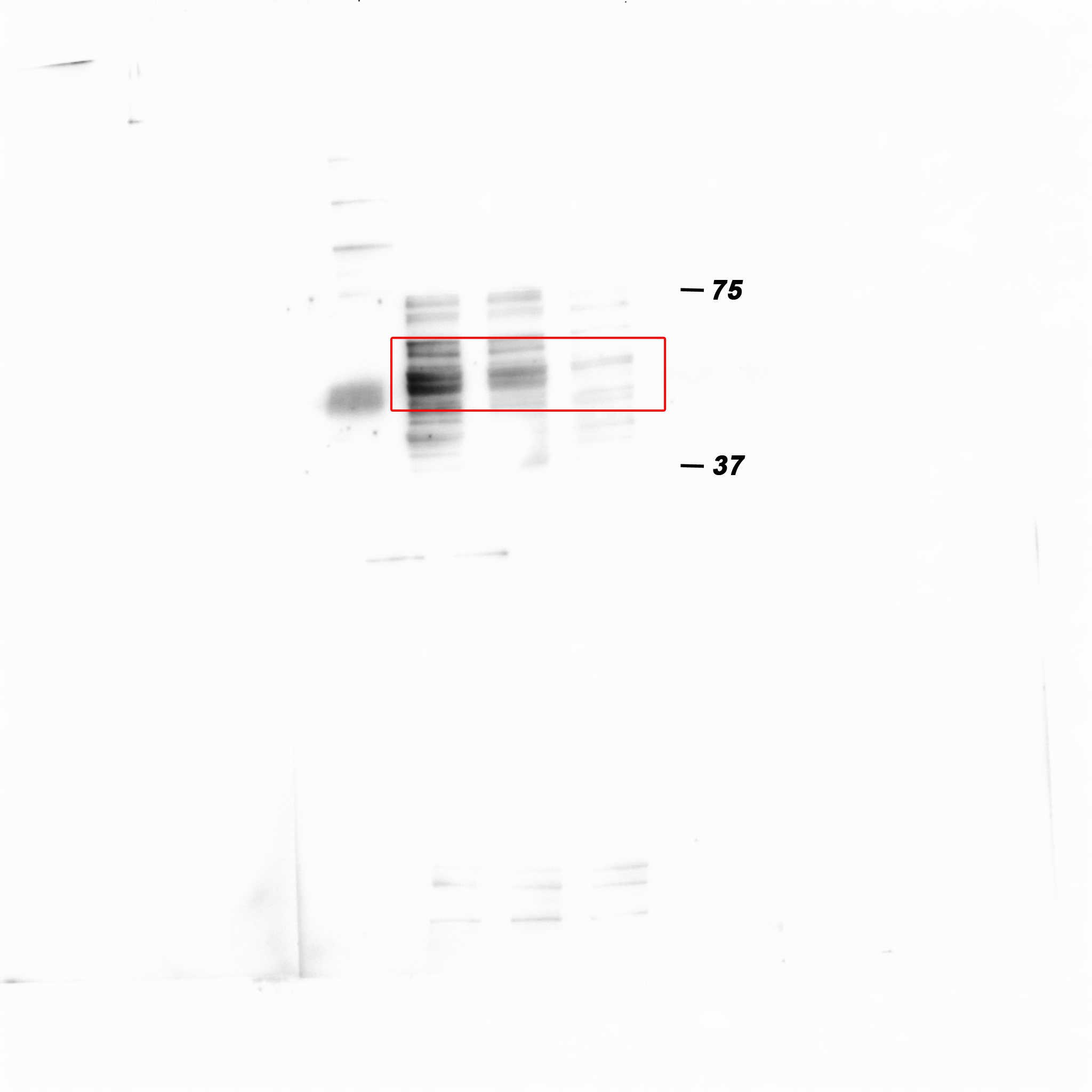

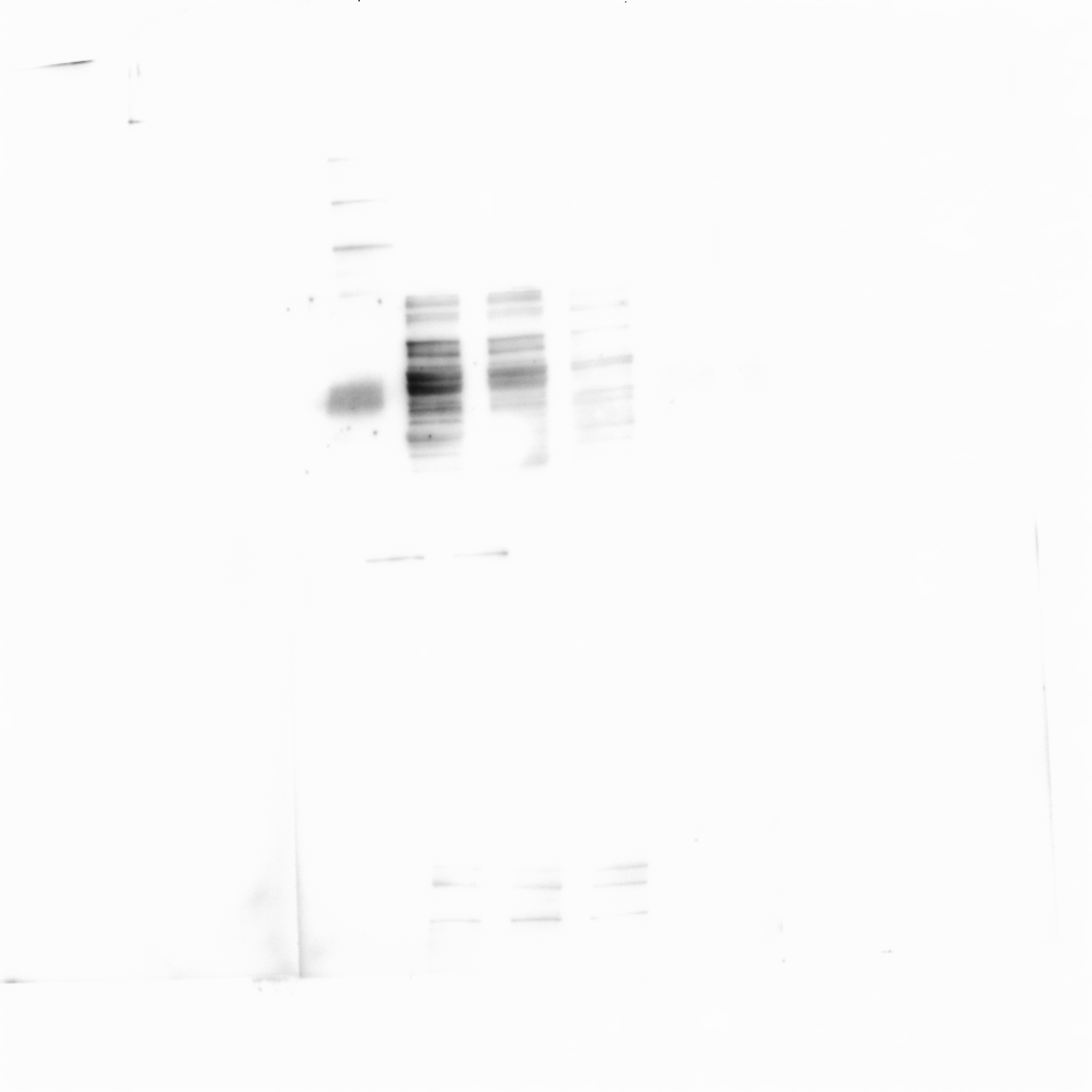
**

Western blot analysis of MMP3 protein levels in either vehicle or Pizotifen-treated MDA-MB-231 cells or E-cadherin positive cells in Pizotifen-treated MDA-MB-231 cells.

**Figure 5D_S100A4_source data**

**
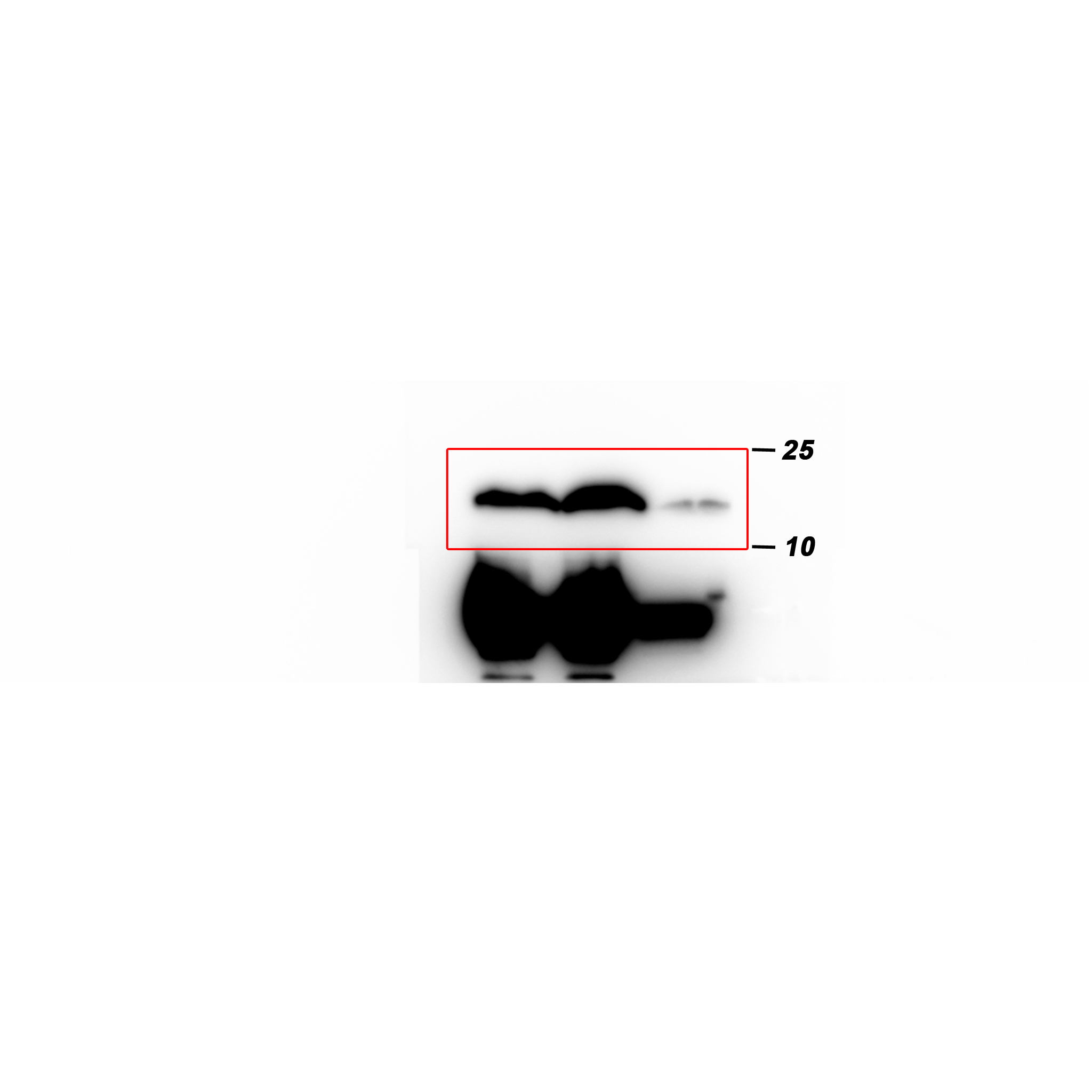

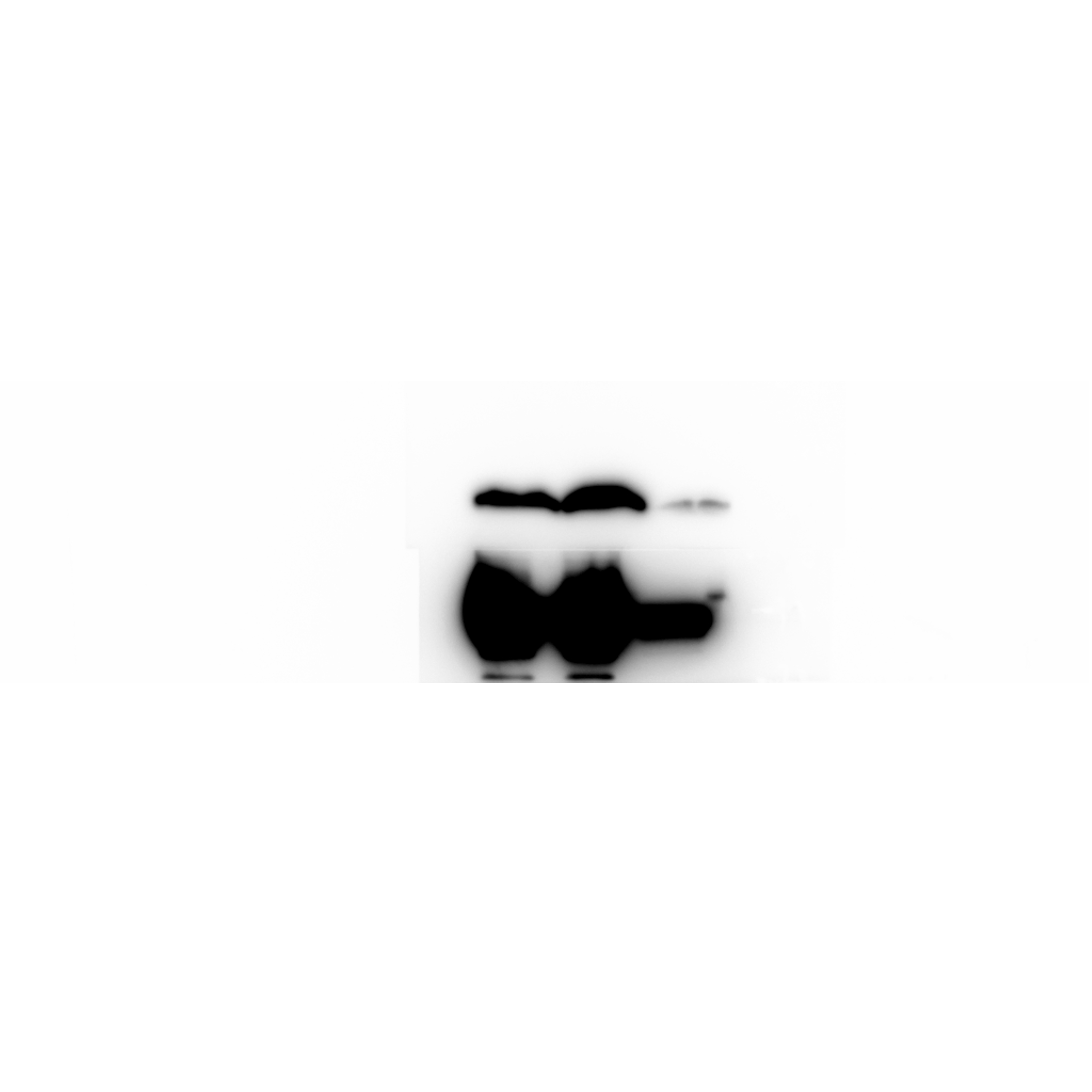
**

Western blot analysis of S100A4 protein levels in either vehicle or Pizotifen-treated MDA-MB-231 cells or E-cadherin positive cells in Pizotifen-treated MDA-MB-231 cells.

**Figure 5D_ZEB1_source data**

**
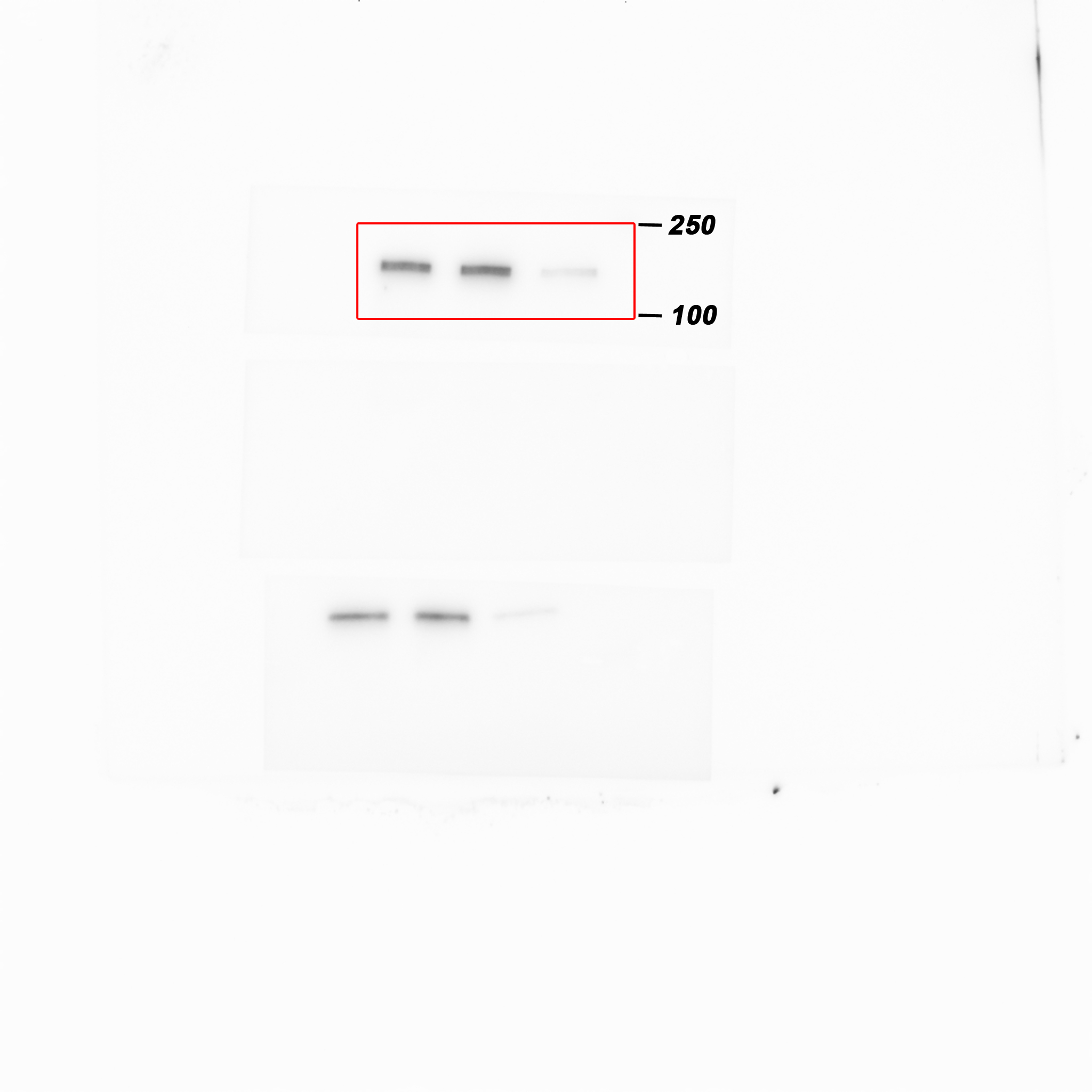

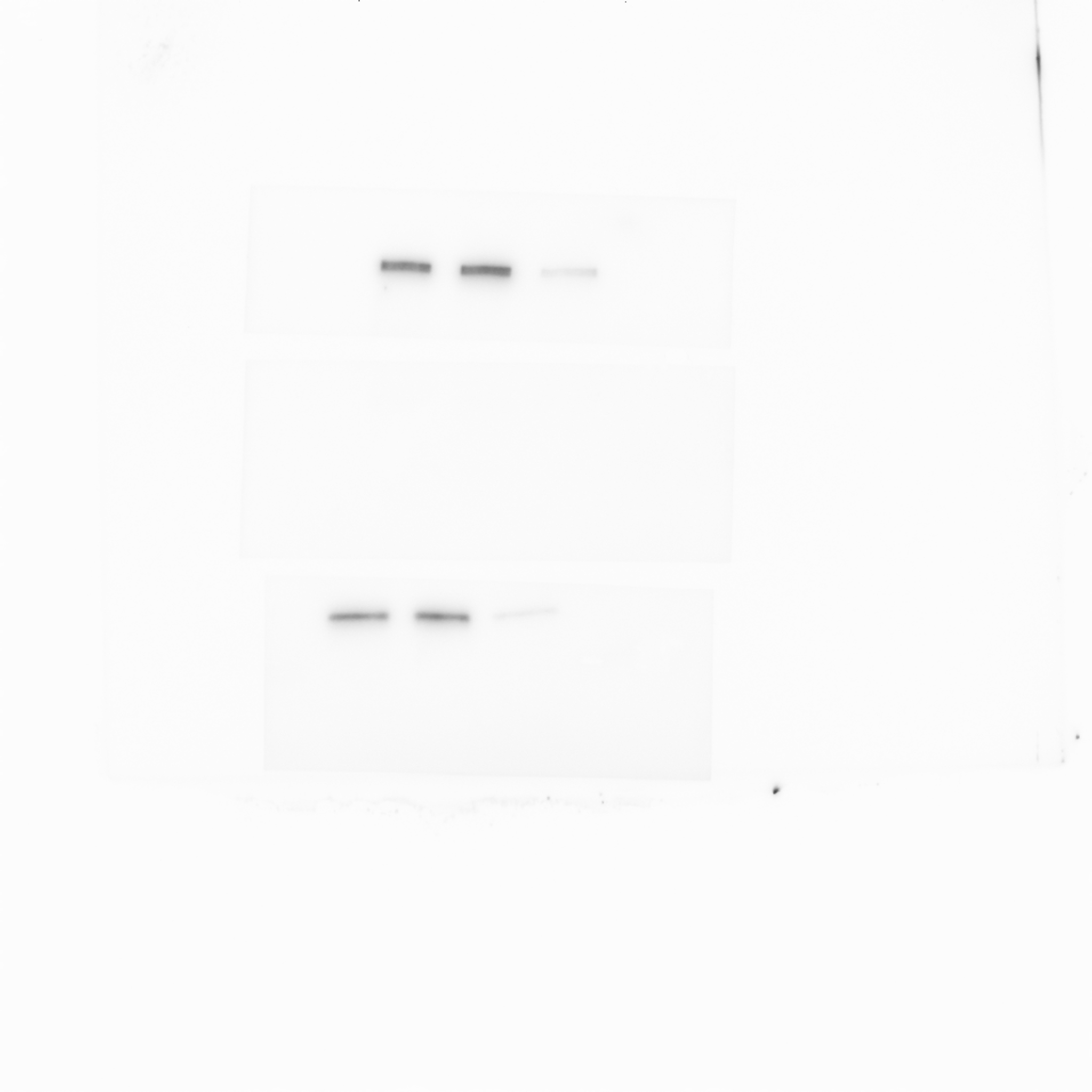
**

Western blot analysis of ZEB1 protein levels in either vehicle or Pizotifen-treated MDA-MB-231 cells or E-cadherin positive cells in Pizotifen-treated MDA-MB-231 cells.

**Figure 5D_GAPDH_source data**

**
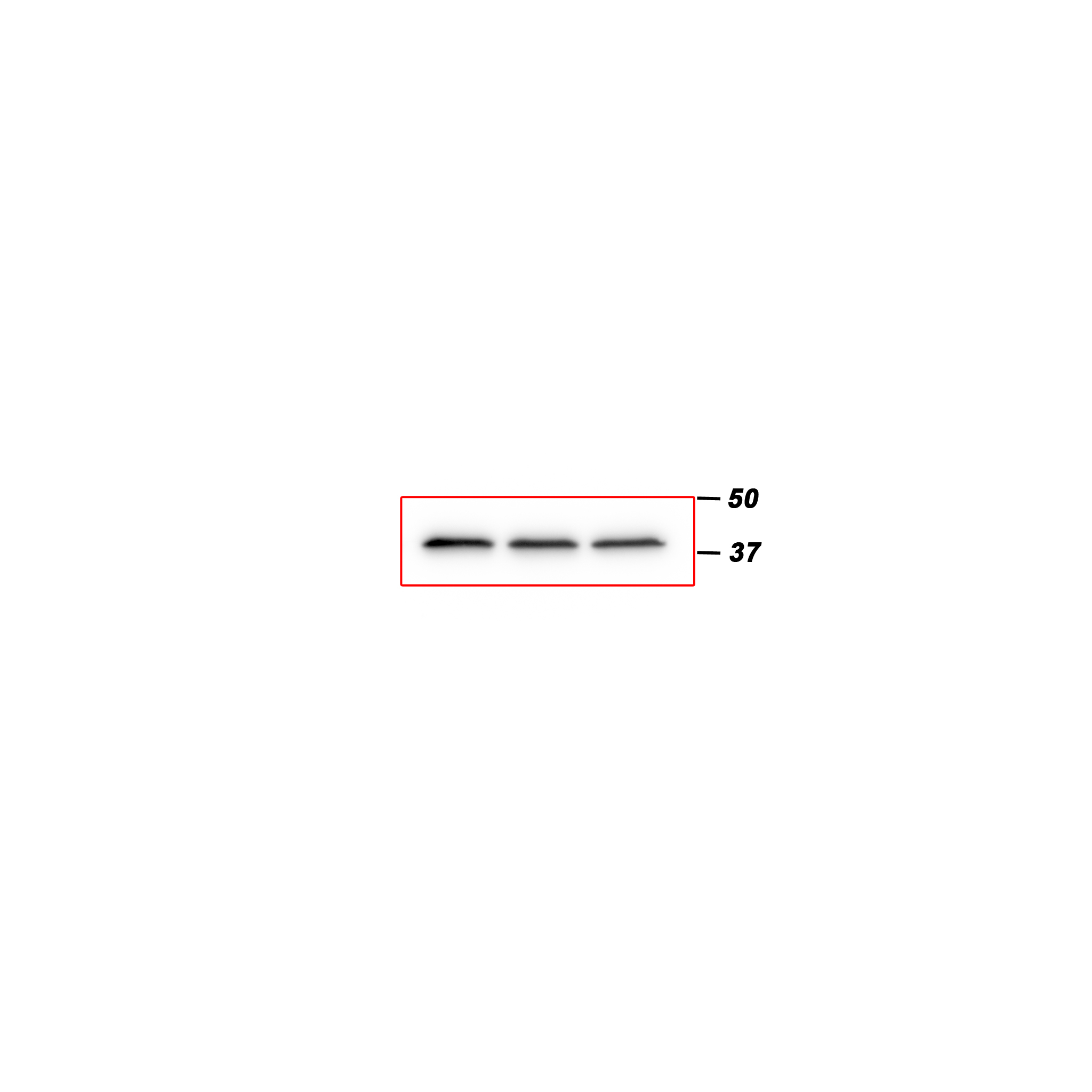

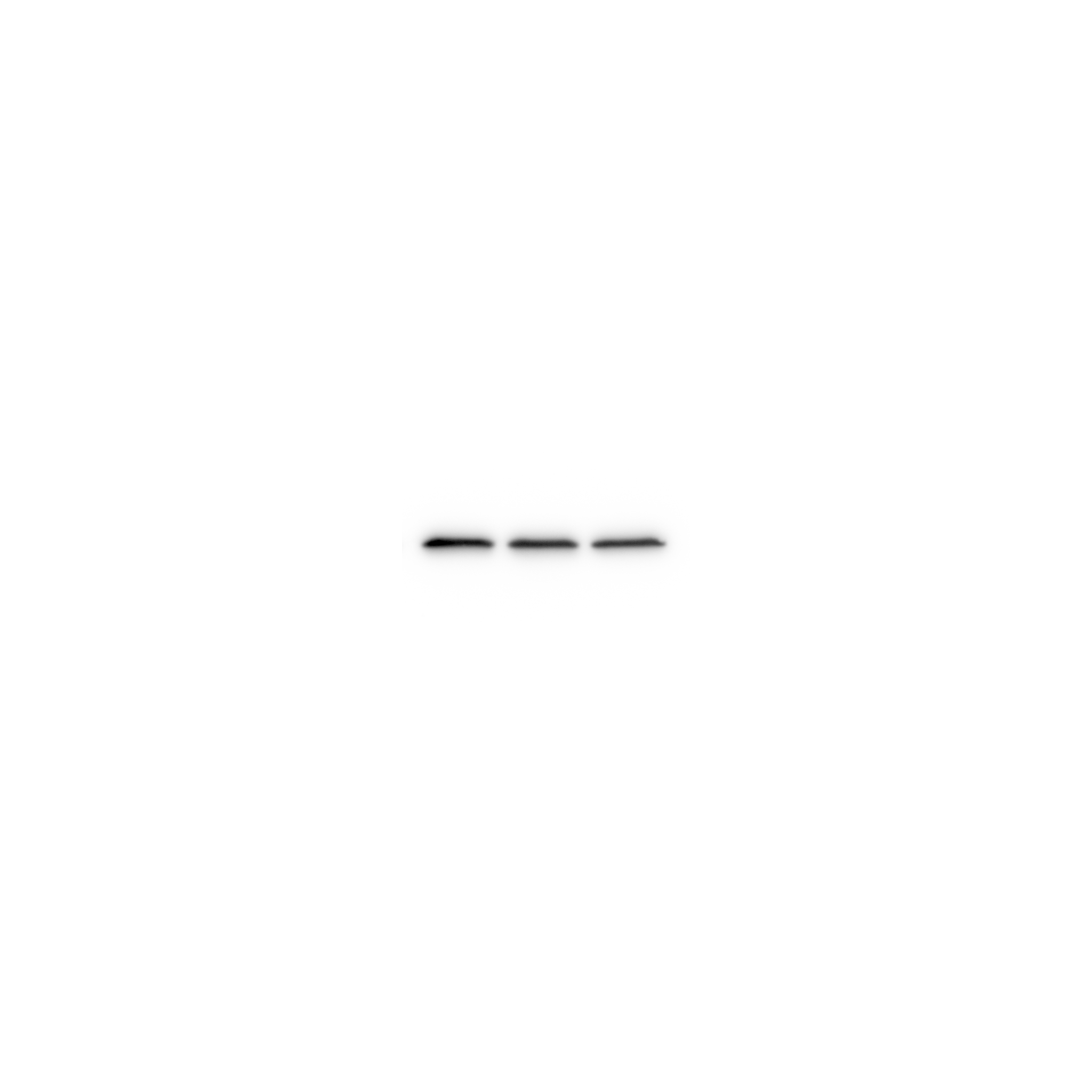
**

Western blot analysis of GAPDH protein levels in either vehicle or Pizotifen-treated MDA-MB-231 cells or E-cadherin positive cells in Pizotifen-treated MDA-MB-231 cells.

**Figure 5F_**β**-catenin in the nucleus_source data**

**
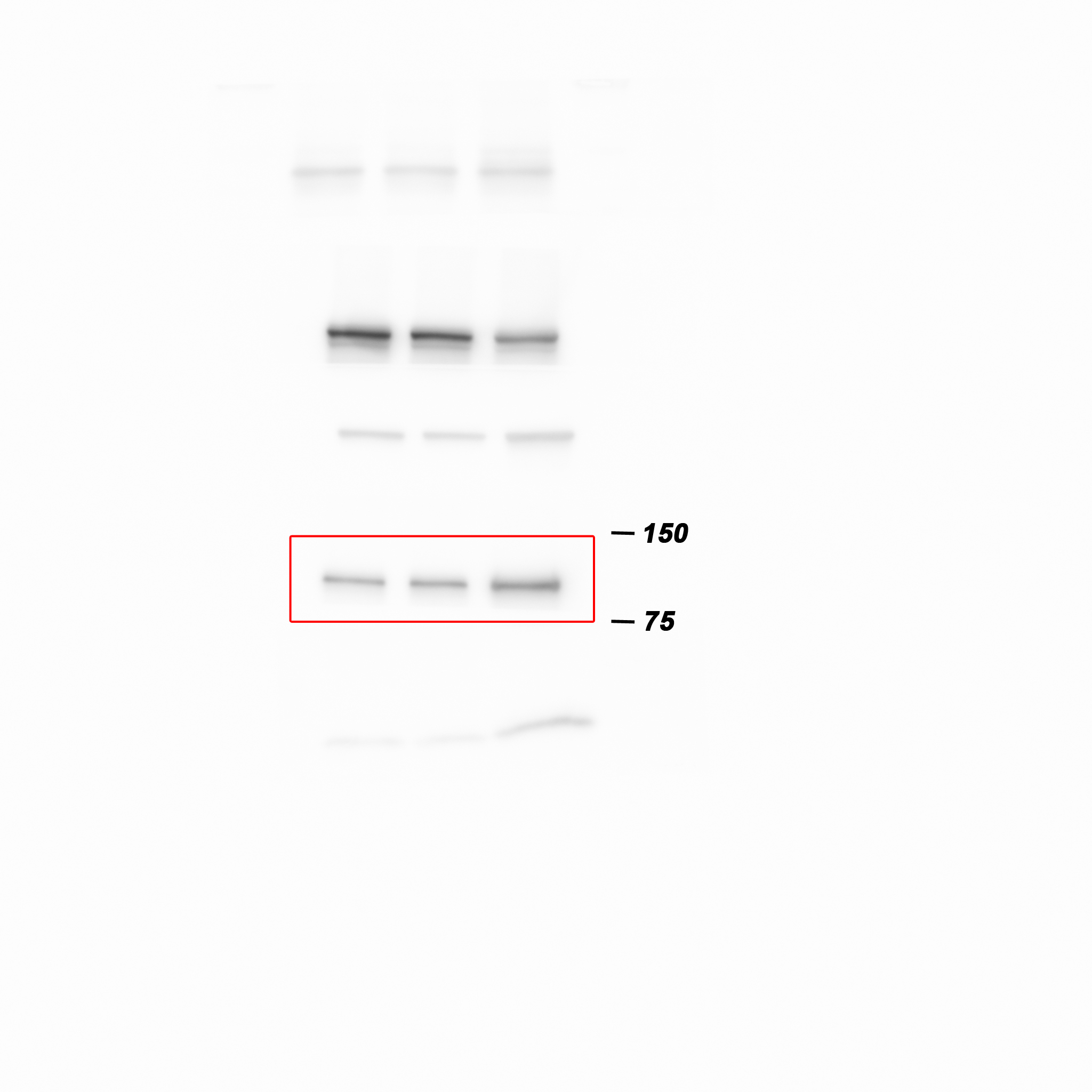

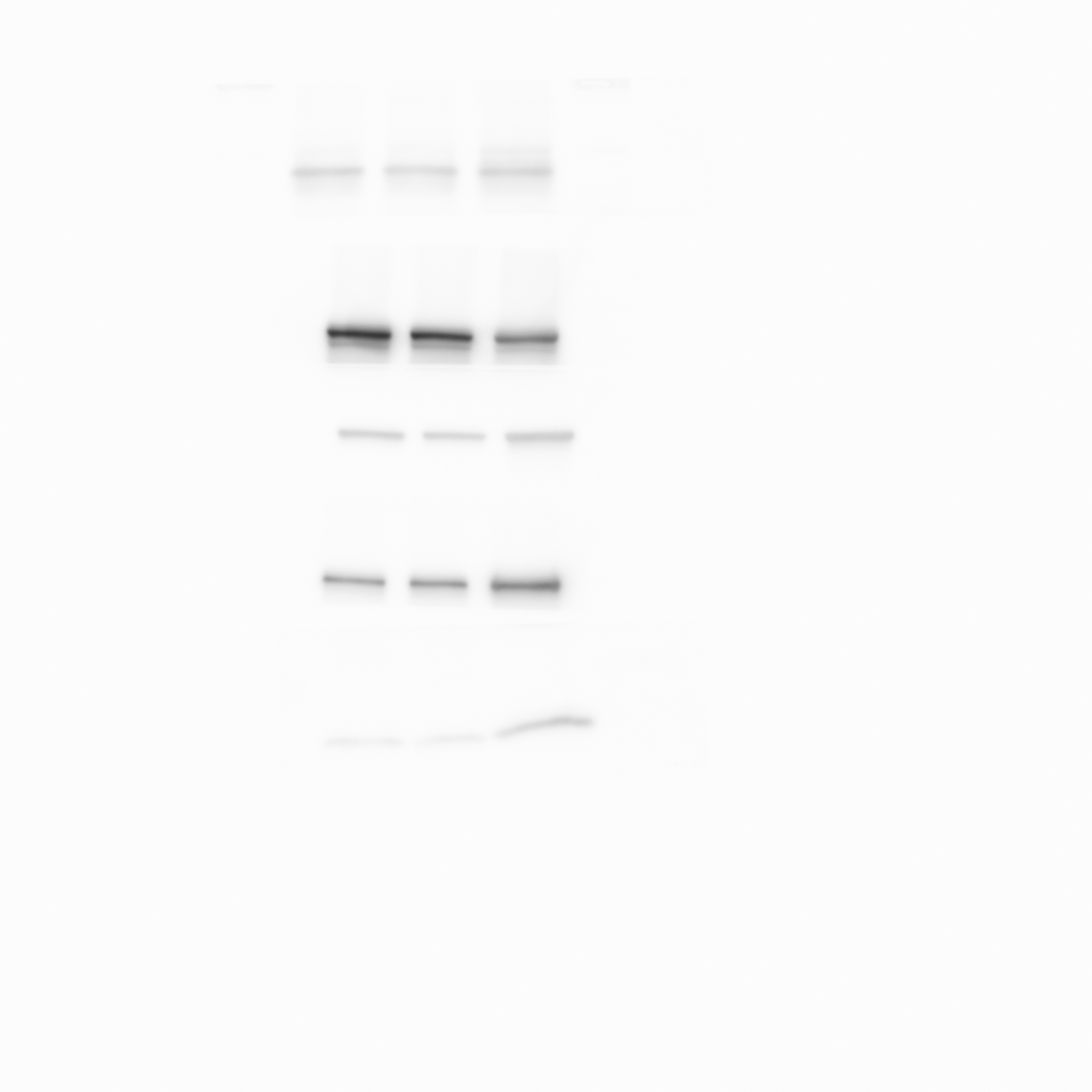
**

Western blot analysis of β-catenin protein levels in the nuclear of MCF7 cells expressing either the control vector or HTR2C.

**Figure 5F_Histone H3 in the nucleus _source data**

**
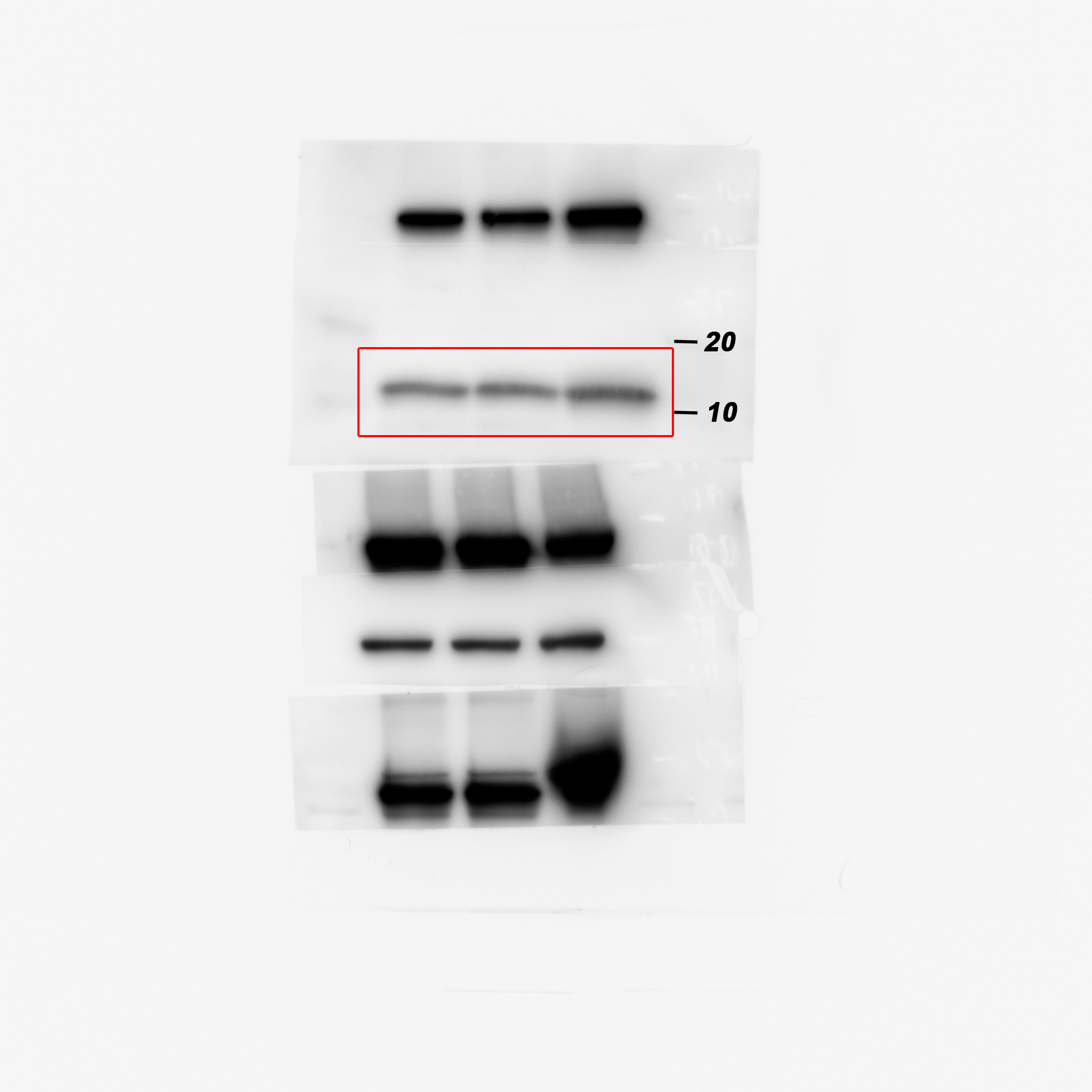

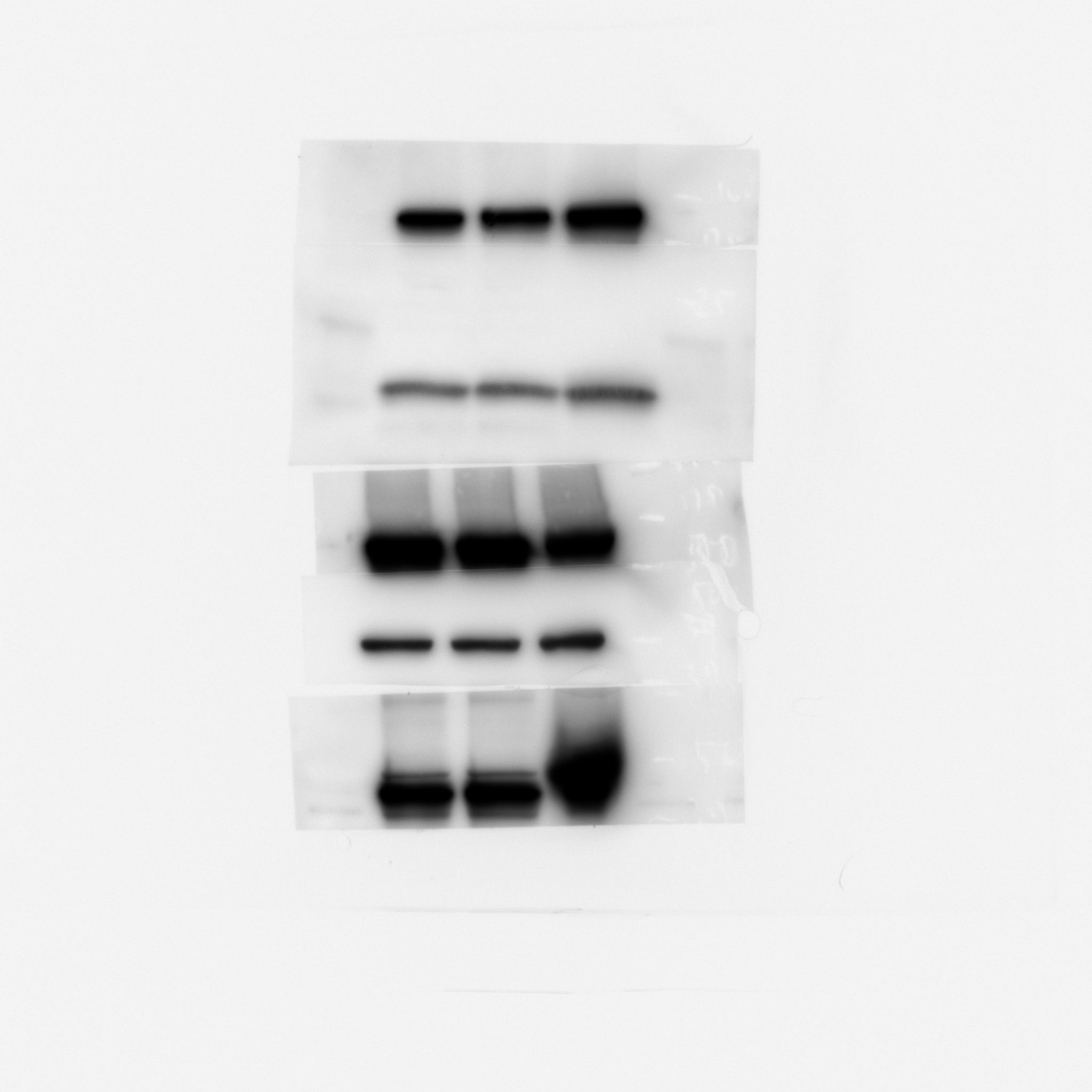
**

Western blot analysis of Histone H3 protein levels in the nuclear of MCF7 cells expressing either the control vector or HTR2C.

**Figure 5F_**β**-catenin in the cytoplasm_source data**

**
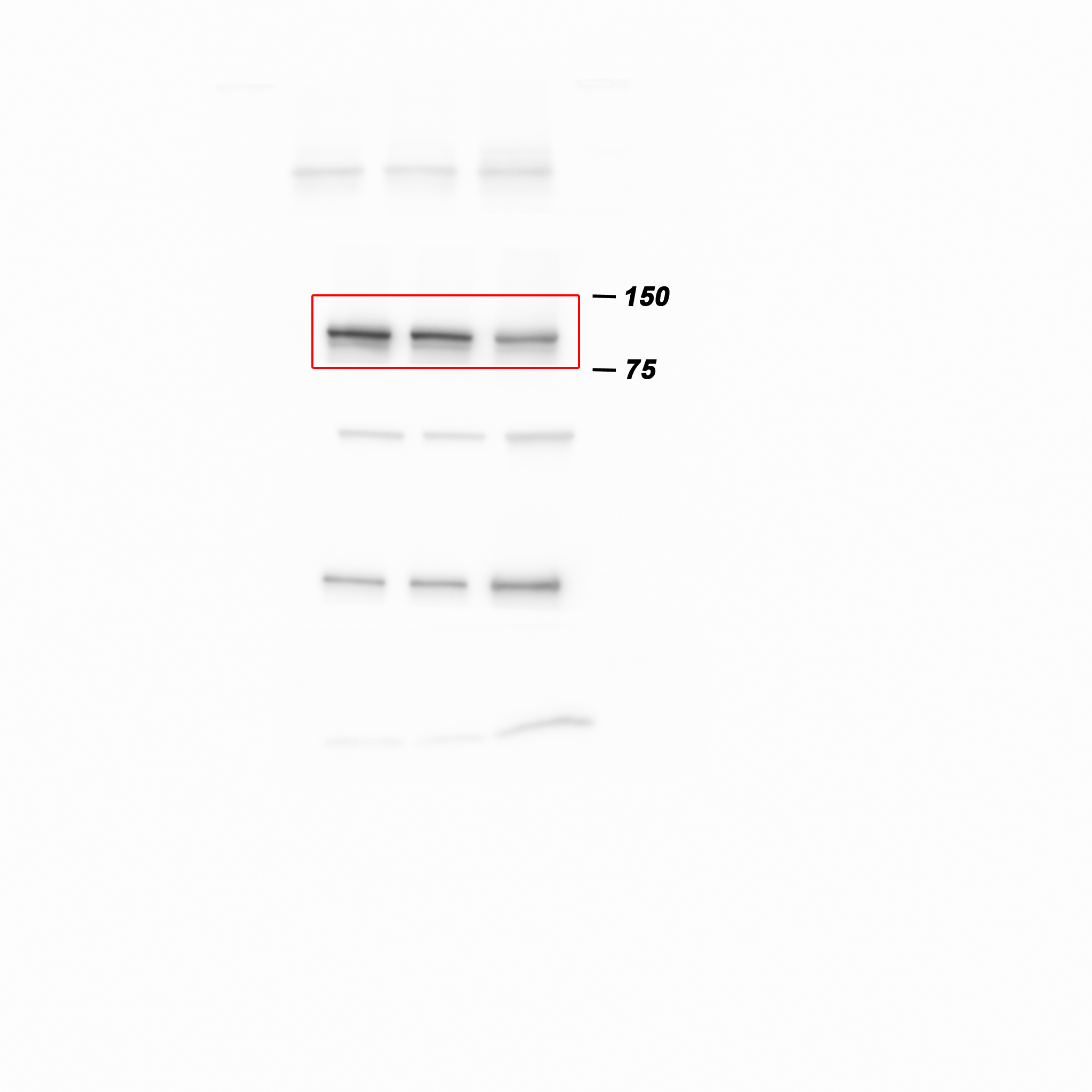

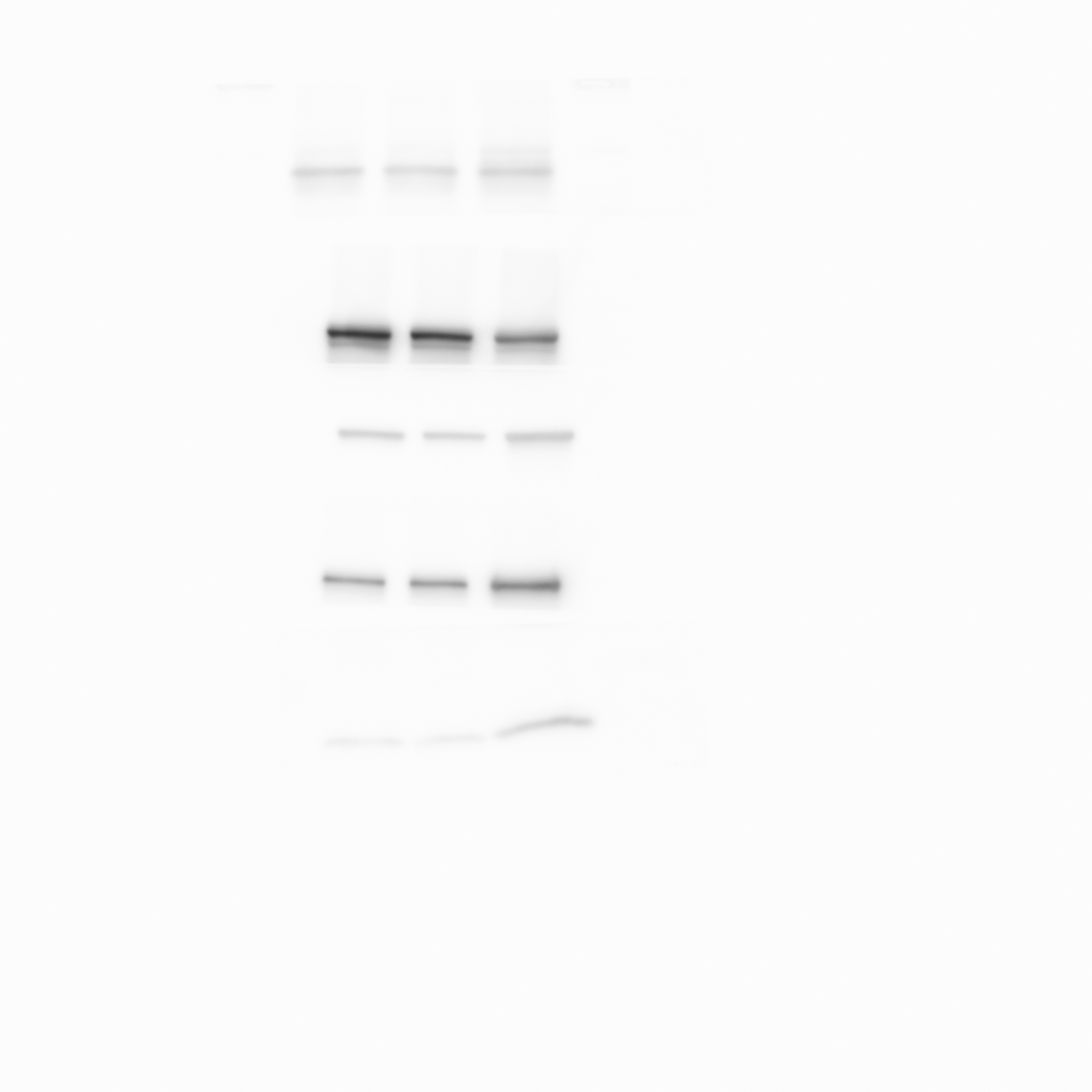
**

Western blot analysis of β-catenin protein levels in the cytoplasm of MCF7 cells expressing either the control vector or HTR2C.

**Figure 5F_**β**-tubulin in the cytoplasm_source data**

**
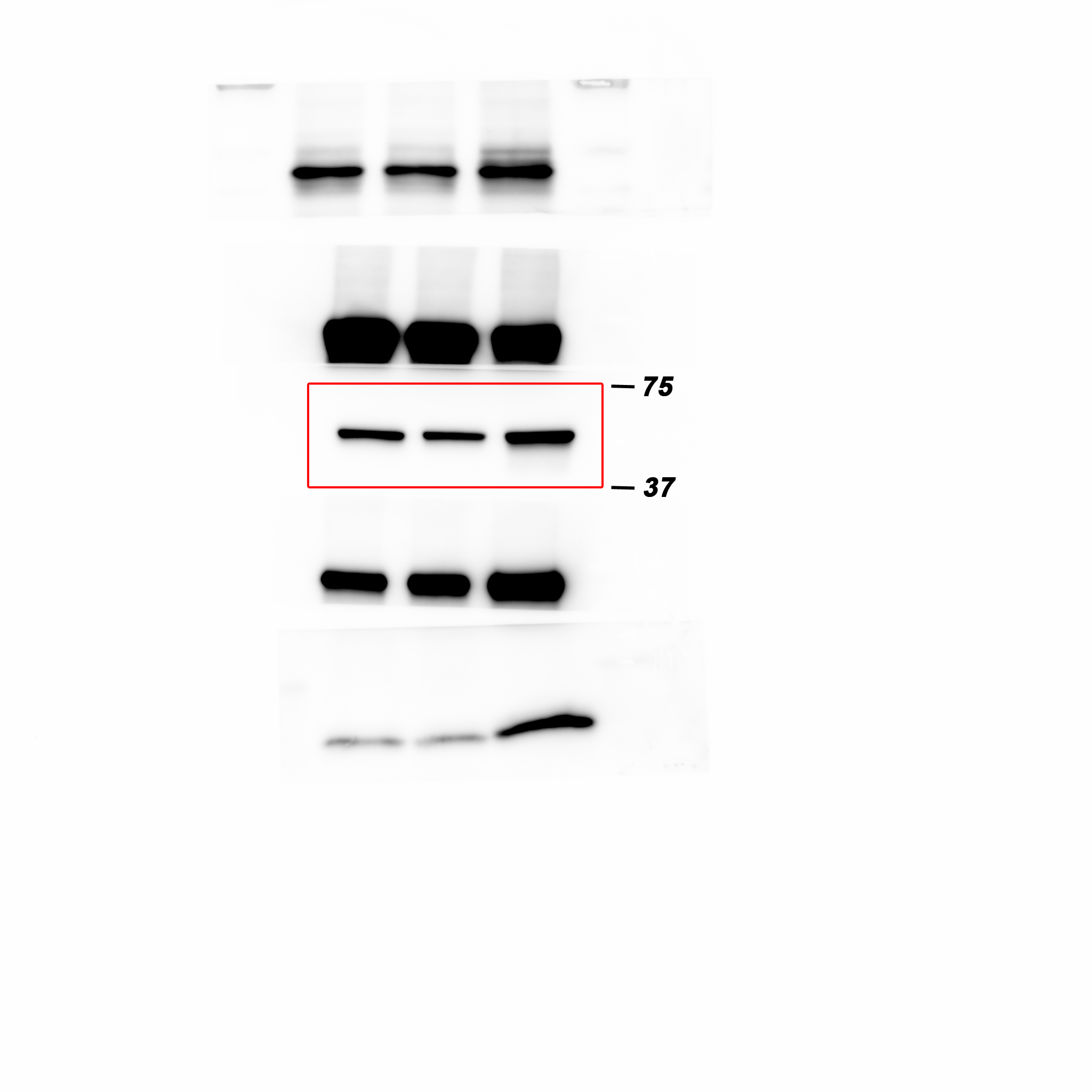

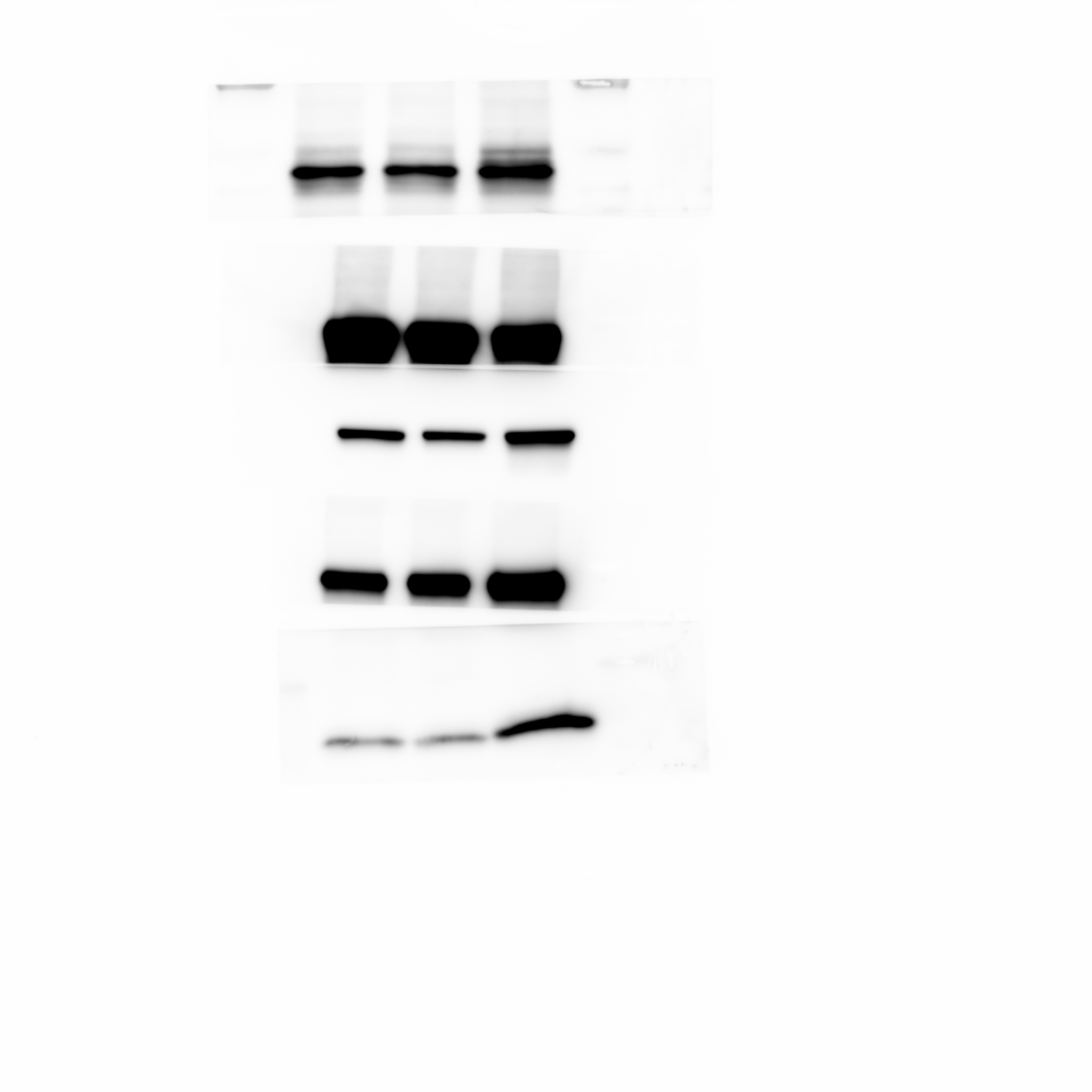
**

Western blot analysis of β-tubulin protein levels in the cytoplasm of MCF7 cells expressing either the control vector or HTR2C.

**Figure 5F_Phosphorylation of serine-9 in GSK3**β**_source data**

**
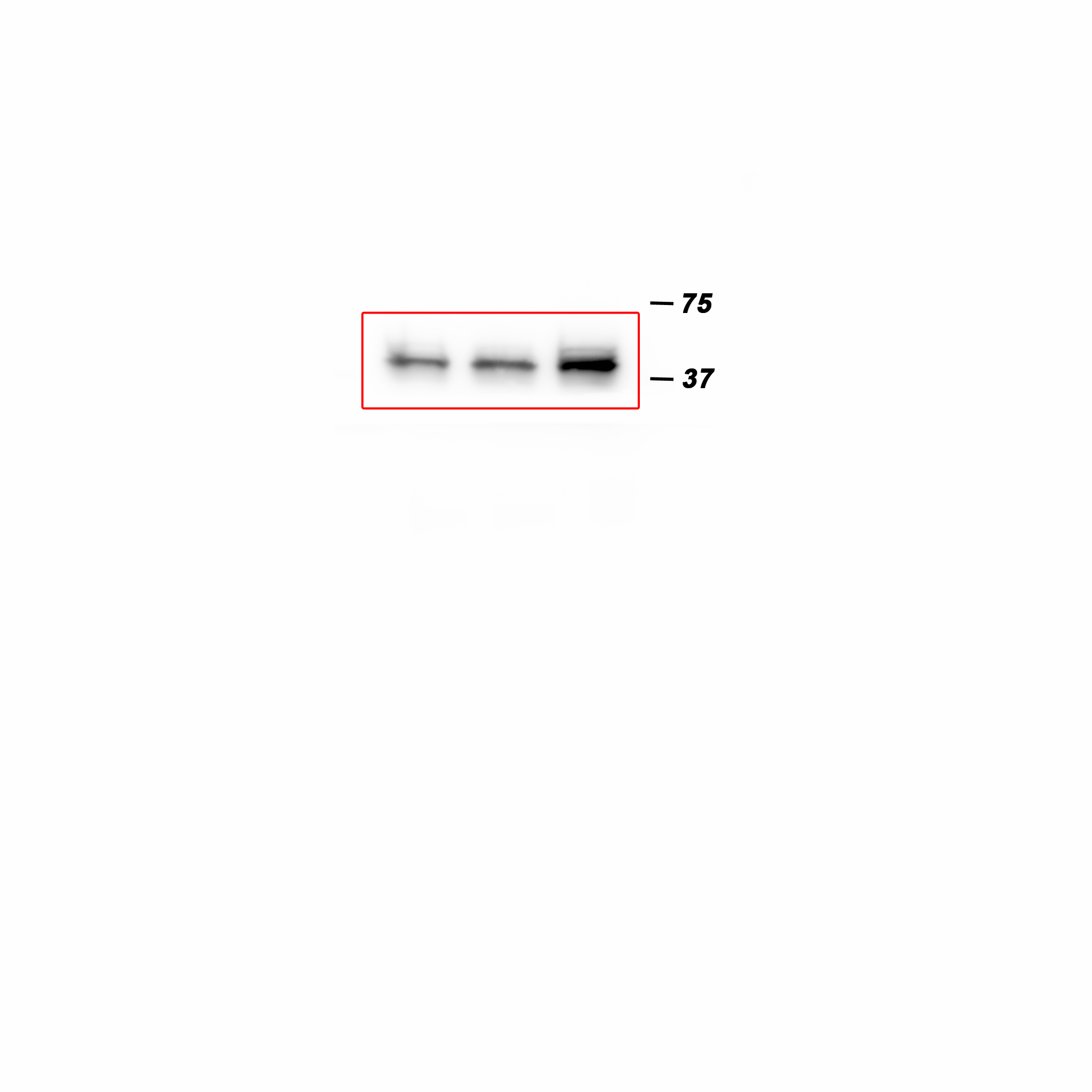

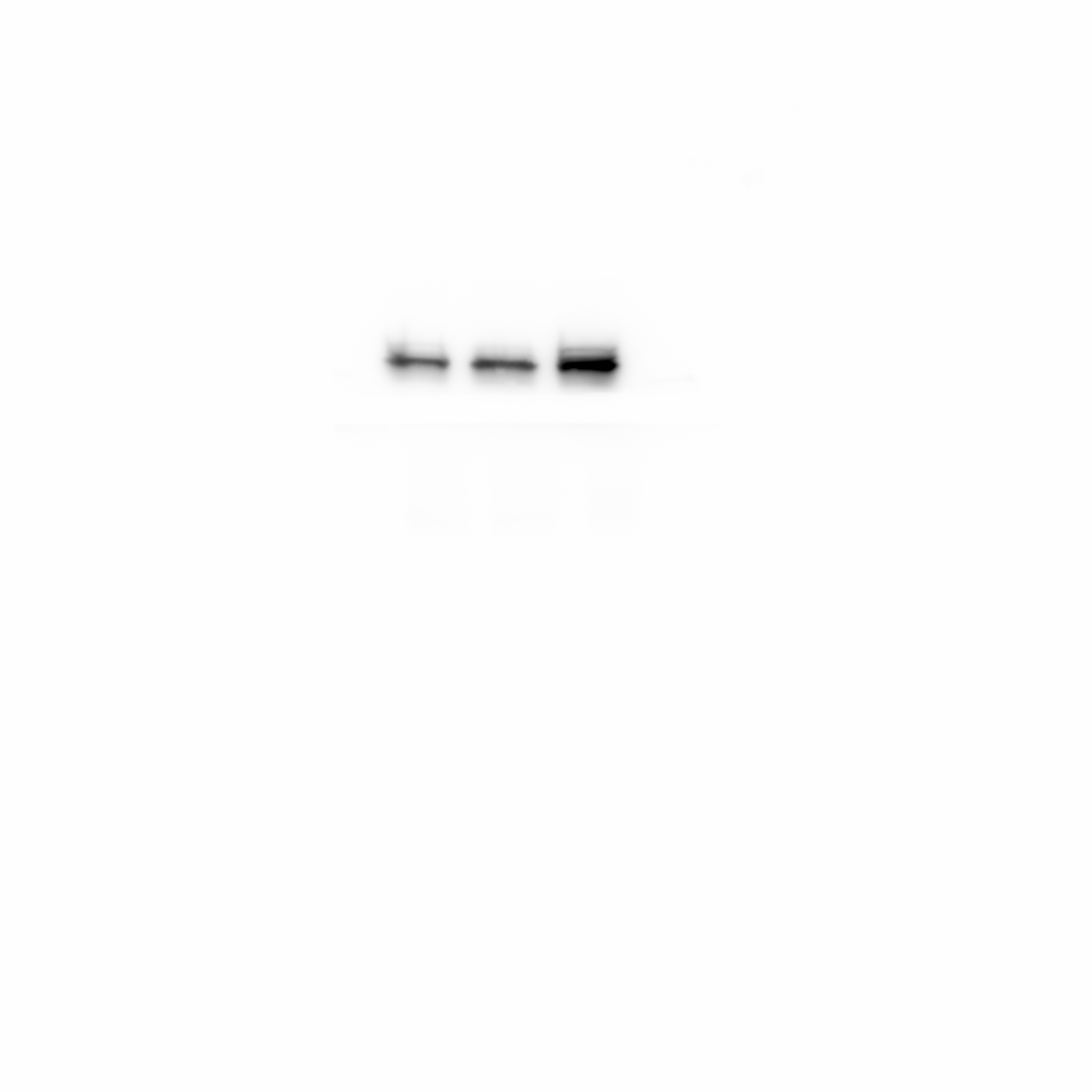
**

Western blot analysis of the protein levels of phosphorylation of serine-9 in GSK3β in whole cell lysate of MCF7 cells expressing either the control vector or HTR2C.

**Figure 5F_GSK3**β**_source data**

**
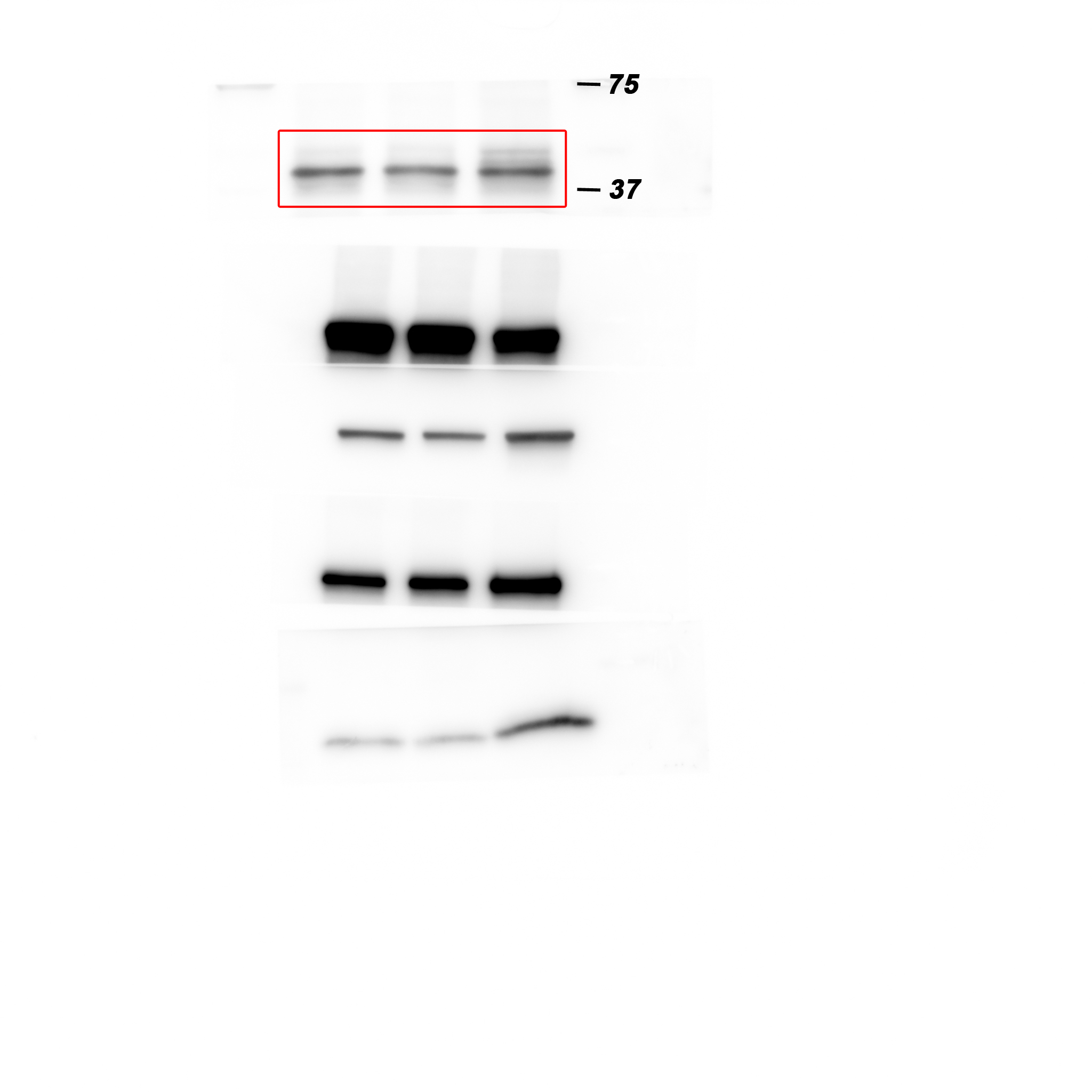

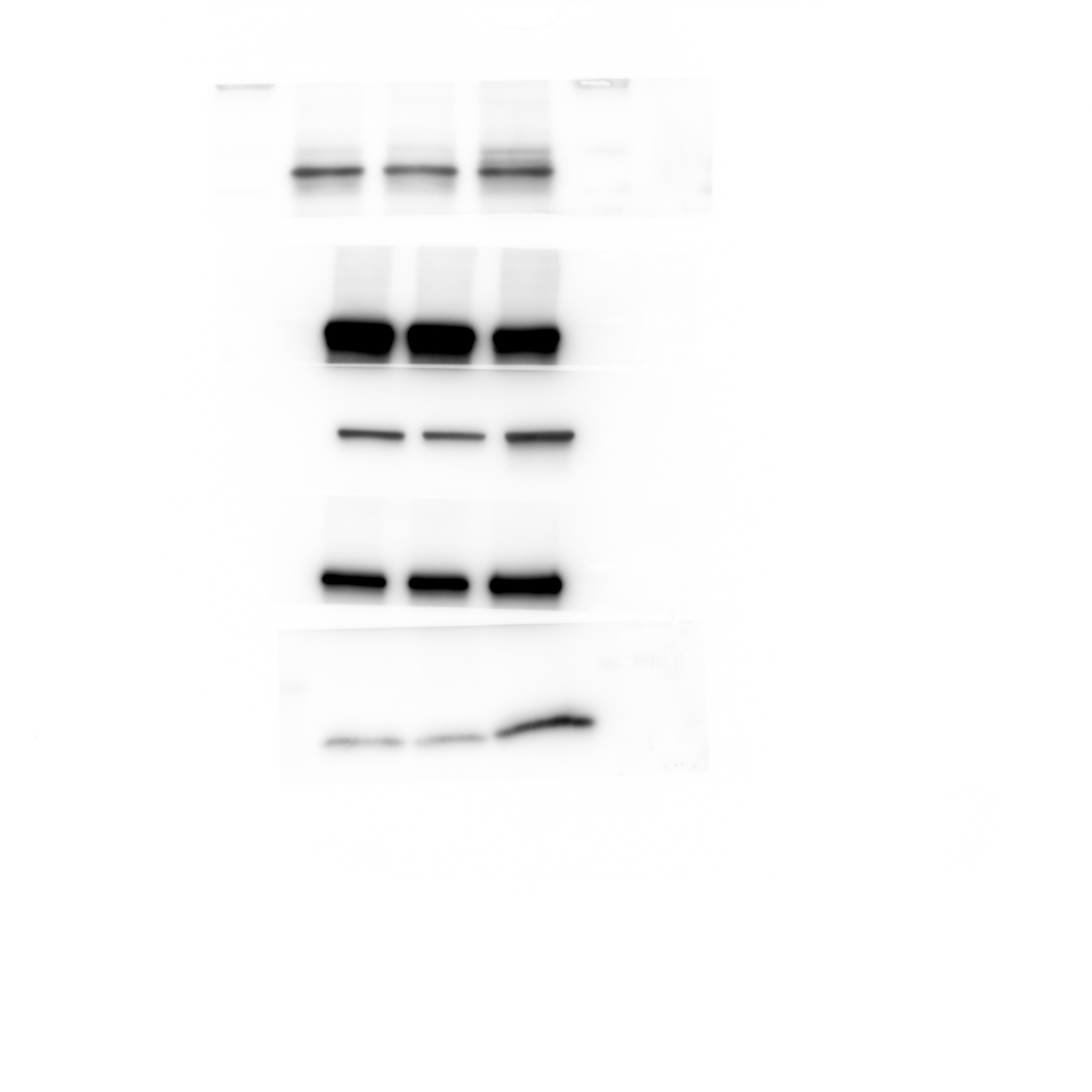
**

Western blot analysis of GSK3β protein levels in whole cell lysate of MCF7 cells expressing either the control vector or HTR2C.

**Figure 5F_GAPDH_source data**

Western blot analysis of GAPDH protein levels in whole cell lysate of MCF7 cells expressing either the control vector or HTR2C.

**Figure 5H_**β**-catenin in the nucleus_source data**

Western blot analysis of β-catenin protein levels in the nucleus of either vehicle or Pizotifen-treated MDA-MB-231 cells or E-cadherin positive cells in Pizotifen-treated MDA-MB-231 cells.

**Figure 5H_Histone H3 in the nucleus _source data**

Western blot analysis of Histone H3 protein levels in the nucleus of either vehicle or Pizotifen-treated MDA-MB-231 cells or E-cadherin positive cells in Pizotifen-treated MDA-MB-231 cells.

**Figure 5H_**β**-catenin in the cytoplasm_source data**

Western blot analysis of β-catenin protein levels in the cytoplasm of either vehicle or Pizotifen-treated MDA-MB-231 cells or E-cadherin positive cells in Pizotifen-treated MDA-MB-231 cells.

**Figure 5H_**β**-tubulin in the cytoplasm_source data**

Western blot analysis of β-tubulin protein levels in the cytoplasm of either vehicle or Pizotifen-treated MDA-MB-231 cells or E-cadherin positive cells in Pizotifen-treated MDA-MB-231 cells.

**Figure 5H_Phosphorylation of serine-9 in GSK3**β**_source data**

Western blot analysis of the protein levels of phosphorylation of serine-9 in GSK3β in whole cell lysate of either vehicle or Pizotifen-treated MDA-MB-231 cells or E-cadherin positive cells in Pizotifen-treated MDA-MB-231 cells.

**Figure 5H_GSK3**β**_source data**

Western blot analysis of GSK3β protein levels in whole cell lysate of either vehicle or Pizotifen-treated MDA-MB-231 cells or E-cadherin positive cells in Pizotifen-treated MDA-MB-231 cells.

**Figure 5H_GAPDH_source data**

Western blot analysis of GAPDH protein levels in whole cell lysate of either vehicle or Pizotifen-treated MDA-MB-231 cells or E-cadherin positive cells in Pizotifen-treated MDA-MB-231 cells.

**Figure 5-figure supplement 4_Snail (left) _source data**

Western blot analysis of Snail protein levels in MCF7 cells expressing either the control vector or HTR2C.

**Figure 5-figure supplement 4_ GAPDH in MCF7l (left) _source data**

Western blot analysis of GAPDH protein levels in MCF7 cells expressing either the control vector or HTR2C. GAPDH control was obtained in the same experiment from Figure 4C.

**Figure 5-figure supplement 4_Twist1 (left) _source data**

Western blot analysis of Twist1 protein levels in MCF7 cells expressing either the control vector or HTR2C.

**Figure 5-figure supplement 4_Twist1 (middle)**

Western blot analysis of Twist1 protein levels in either vehicle or Pizotifen-treated MDA-MB-231 cells.

**Figure 5-figure supplement 4_GAPDH (middle) _source data**

Western blot analysis of GAPDH protein levels in either vehicle or Pizotifen-treated MDA-MB-231 cells.

**Figure 5-figure supplement 4_Snail (right) _source data**

Western blot analysis of Snail protein levels in whole cell lysate of 4T1 primary tumors from either vehicle or Pizotifen-treated mice.

**Figure 5-figure supplement 4_Twist1 (right) _source data**

Western blot analysis of Twist1 protein levels in whole cell lysate of 4T1 primary tumors from either vehicle or Pizotifen-treated mice.

**Figure 5-figure supplement 4_ Luciferase (right) _source data**

Western blot analysis of Luciferase protein levels in whole cell lysate of 4T1 primary tumors from either vehicle or Pizotifen-treated mice. Luciferase control was obtained in the same experiment from Figure 5C.
