# Supplementary material for: A zebrafish embryo screen utilizing gastrulation identifies the HTR2C inhibitor pizotifen as a suppressor of EMT-mediated metastasis
Source: eLife. 2021 Dec 17;10:e70151. doi: 10.7554/eLife.70151 (PMC8824480; doi:10.7554/eLife.70151)
Supplement: Transparent reporting form [file elife-70151-transrepform1.docx]

***eLife’s* transparent reporting form**

We encourage authors to provide detailed information *within their submission* to facilitate the interpretation and replication of experiments. Authors can upload supporting documentation to indicate the use of appropriate reporting guidelines for health-related research (see [EQUATOR Network](http://www.equator-network.org/%20)), life science research (see the [BioSharing Information Resource](https://biosharing.org/)), or the [ARRIVE guidelines](http://www.plosbiology.org/article/info:doi/10.1371/journal.pbio.1000412) for reporting work involving animal research. Where applicable, authors should refer to any relevant reporting standards documents in this form.

If you have any questions, please consult our Journal Policies and/or contact us: [editorial@elifesciences.org](mailto:editorial@elifesciences.org).

**Sample-size estimation**

- You should state whether an appropriate sample size was computed when the study was being designed
- You should state the statistical method of sample size computation and any required assumptions
- If no explicit power analysis was used, you should describe how you decided what sample (replicate) size (number) to use

Please outline where this information can be found within the submission (e.g., sections or figure legends), or explain why this information doesn’t apply to your submission:

Figure 1_We used approximately twenty zebrafish embryos to evaluate whether one drug might interrupt epiboly of the embryos. The number needs to judge the effects.

Figure 2_ We injected human cancer cells into approximately *5*0 zebrafish and then the zebrafish were split into two group: vehicle-treated group and Pizotifen-treated group. The number needs to perform statistical analysis.

Figure 3_ We obtained data from 10 vehicle-treated mice and 10 Pizotifen-treated mice. The number needs to perform statistical analysis.

Figure 4_ We injected MCF7 cells expressing vector control into approximately 20 zebrafish, and also injected MCF7 cells expressing HTR2C into approximately 20 zebrafish. The number needs to perform statistical analysis.

**Replicates**

- You should report how often each experiment was performed
- You should include a definition of biological versus technical replication
- The data obtained should be provided and sufficient information should be provided to indicate the number of independent biological and/or technical replicates
- If you encountered any outliers, you should describe how these were handled
- Criteria for exclusion/inclusion of data should be clearly stated
- High-throughput sequence data should be uploaded before submission, with a private link for reviewers provided (these are available from both GEO and ArrayExpress)

Please outline where this information can be found within the submission (e.g., sections or figure legends), or explain why this information doesn’t apply to your submission:

Figure 1B_we confirmed whether positive hit drugs could interrupt zebrafish epiboly progression.

Figure 2A-E_Each experiment was performed at least twice.

Figure 3A-E_We obtained data from 10 vehicle-treated mice and 10 Pizotifen-treated mice.

Figure 3F-G_ We obtain data from 10 mice that were inoculated with 4T1-12B cells expressing shRNA targeting for either LacZ (n=5) or HTR2C (n=5)

Figure 4A-E_Each experiment was performed twice.

Figure 5A-H_Each experiment was performed twice.

**Statistical reporting**

- Statistical analysis methods should be described and justified
- Raw data should be presented in figures whenever informative to do so (typically when N per group is less than 10)
- For each experiment, you should identify the statistical tests used, exact values of N, definitions of center, methods of multiple test correction, and dispersion and precision measures (e.g., mean, median, SD, SEM, confidence intervals; and, for the major substantive results, a measure of effect size (e.g., Pearson's r, Cohen's d)
- Report exact p-values wherever possible alongside the summary statistics and 95% confidence intervals. These should be reported for all key questions and not only when the p-value is less than 0.05.

Please outline where this information can be found within the submission (e.g., sections or figure legends), or explain why this information doesn’t apply to your submission:

All of statistical analyses in this study were performed by student "T" test.

Raw data of Figure 2D is showed in Table S3.

Raw data of Figure S4B is showed in Table S4.

Raw data of Figure 2E is showed in Table S5.

Raw data of Figure 4E is showed in Table S6.

GSEA source data 1 of gene expression of Figure S1 is attached as GSEA source data 1.

GSEA source data 2 of enriched pathways Figure S1 is attached as GSEA source data 2.

(For large datasets, or papers with a very large number of statistical tests, you may upload a single table file with tests, Ns, etc., with reference to sections in the manuscript.)

**Group allocation**

- Indicate how samples were allocated into experimental groups (in the case of clinical studies, please specify allocation to treatment method); if randomization was used, please also state if restricted randomization was applied
- Indicate if masking was used during group allocation, data collection and/or data analysis

Please outline where this information can be found within the submission (e.g., sections or figure legends), or explain why this information doesn’t apply to your submission:

Figure 1_To adjust development stage of zebrafish embryos, zebrafish embryos at two cell stage were collected at 20 mins after their fertilization. And then approximately 20 zebrafish embryos were allocated into each well of 24-well plate.

Figure 2D_*Tg(kdrl:eGFP)* zebrafish at 2 dpf were inoculated with mCherry-labelled human cancer cells lines and randomly assigned to two groups. One group was maintained in the presence of pizotifen-containing E3 medium and the other group was maintained in vehicle containing E3 medium.

Figure 3_on day two post inoculation, 20 of a female BALB/c mice were randomly assigned to two groups and one group received once daily intraperitoneal injections of 10mg/kg Pizotifen while the other group received a vehicle injection.

**Additional data files (“source data”)**

- We encourage you to upload relevant additional data files, such as numerical data that are represented as a graph in a figure, or as a summary table
- Where provided, these should be in the most useful format, and they can be uploaded as “Source data” files linked to a main figure or table
- Include model definition files including the full list of parameters used
- Include code used for data analysis (e.g., R, MatLab)
- Avoid stating that data files are “available upon request”

Please indicate the figures or tables for which source data files have been provided:

Figure 2A, Figure 4C, Figure 5C, Figure 5D, Figure 5F, Figure 5H, Figure S1, Figure S2A, Figure S3B, Figure S4B.
